# XIAP Stabilizes DDRGK1 to Promote ER‐Phagy and Protects Against Noise‐Induced Hearing Loss

**DOI:** 10.1002/advs.202511217

**Published:** 2026-01-26

**Authors:** Lin Yan, Yuhua Zhang, Jiawei Du, Yongjun Zhu, Wei Cao, Yongjie Wei, Han Wu, Shiyu Qiu, Shiyi Pan, Lian Chen, Pingping Liang, Renjie Chai, Jianming Yang, Qiaojun Fang

**Affiliations:** ^1^ Department of Otolaryngology‐Head and Neck Surgery The Second Affiliated Hospital of Anhui Medical University Hefei China; ^2^ School of Life Sciences Anhui Medical University Hefei China; ^3^ State Key Laboratory of Digital Medical Engineering Department of Otolaryngology Head and Neck Surgery Zhongda Hospital School of Life Sciences and Technology School of Medicine Advanced Institute For Life and Health Jiangsu Province High‐Tech Key Laboratory for Bio‐Medical Research Southeast University Nanjing China; ^4^ Co‐Innovation Center of Neuroregeneration Nantong University Nantong China; ^5^ Department of Neurology School of Life Science Beijing Institute of Technology Aerospace Center Hospital Beijing China; ^6^ Department of Otolaryngology Head and Neck Surgery Sichuan Provincial People's Hospital University of Electronic Science and Technology of China Chengdu China; ^7^ Southeast University Shenzhen Research Institute Shenzhen China

**Keywords:** DDRGK1, ER‐phagy, hair cells, noise‐induced hearing loss, XIAP

## Abstract

Noise‐induced hearing loss (NIHL) is a common cause of acquired sensorineural hearing loss. Excessive endoplasmic reticulum (ER) stress‐induced apoptosis of cochlear hair cells contributes to NIHL. ER autophagy (ER‐phagy) is a critical pathway for maintaining ER homeostasis and cell survival. DDRGK1 (DDRGK domain containing 1) is a crucial receptor in ER‐phagy, essential for the removal of injured ER components. This work investigates the involvement of DDRGK1‐mediated ER‐phagy in NIHL, which has remained unclear. ER‐phagy flux is inhibited in HEI‐OC1 cells treated with hydrogen peroxide. Noise exposure reduces XIAP (X‐linked inhibitor of apoptosis protein) and DDRGK1 protein levels in these cells. Moreover, XIAP binds to DDRGK1, increasing the stability of DDRGK1 and activating ER‐phagy. Notably, in noise‐exposed CBA/CaJ mice, gastrodin, a traditional Chinese medicine ingredient, reduces noise‐induced loss of cochlear hair cells, ribbon synaptic damage, and hearing loss by promoting XIAP expression, thereby increasing DDRGK1 protein levels and activating ER‐phagy. These findings highlight XIAP‐DDRGK1‐mediated ER‐phagy as a novel therapeutic target for NIHL treatment.

## Graphical abstract

Mechanism of GAS‐mediated protection against noise‐induced hearing loss (NIHL). Noise exposure activates the ATF4/eIF2α axis, downregulating XIAP and promoting DDRGK1 degradation, thereby inhibiting ER‐phagy and leading to hair cell (HC) death. GAS treatment rescues XIAP and DDRGK1 expression, reactivating ER‐phagy to mitigate HC loss, synaptic damage, and hearing impairment. Created with BioRender.com.

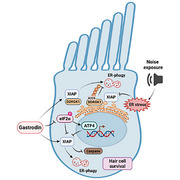

## 1 Introduction

Noise‐induced hearing loss (NIHL), predominantly caused by extended exposure to work or recreational noise, is among the most prevalent types of acquired sensorineural hearing loss, accounting for approximately one‐third of the global cases of hearing loss [1]. Mild‐to‐moderate noise exposure can cause a temporary threshold shift, wherein damage to hair cells (HCs) and auditory nerve fibers is reversible. In contrast, prolonged or intense noise exposure can lead to permanent loss of sensory HCs in the inner ear and disruption of ribbon synaptic connections in inner HCs, resulting in irreversible hearing loss, also known as a permanent threshold shift [2]. According to the World Health Organization, adolescents and young adults are particularly susceptible to NIHL, with an estimated 1 billion individuals aged 12–35 years being at risk [3]. Despite the significant impact of NIHL, no Food and Drug Administration‐approved drug has been used to treat it.

X‐linked inhibitor of apoptosis protein (XIAP) is a key regulator of multiple cell death and inflammatory pathways, controlling cell growth and apoptosis by interacting with and inhibiting various caspase proteins [4]. XIAP is critical for regulating cellular processes, including apoptosis, autophagy, and cell growth [5]. As a classic antiapoptotic protein and an essential E3 ubiquitin ligase, XIAP contributes to carcinogenesis by regulating the stability of various proteins [6]. It stabilizes PARP1 (poly (ADP‐ribose) polymerase 1) by regulating its ubiquitination, thereby promoting pancreatic cancer progression [7]. Previous studies have indicated that XIAP overexpression in mice, owing to its antiapoptotic effects, considerably reduces HC damage caused by noise, neomycin, and cisplatin [8, 9]. XIAP deficiency is associated with defects in autophagy, and this protein plays an important role in promoting autophagy during hemophagocytic lymph histiocytosis [10]. XIAP promotes the survival of cochlear HCs and provides hearing protection via antiapoptotic pathways. However, few studies have investigated other functions of XIAP in NIHL.

Endoplasmic reticulum (ER) autophagy (ER‐phagy) is a major stress response mechanism mediated via ER receptors (e.g., FAM134B, RTN3L, CCPG1, SEC62, and DK5RAP3 (DDRGK1) [11], through which excess ER fragments and abnormal resident proteins within the ER are transported to lysosomes for rapid elimination, allowing maintenance of ER size and homeostasis [12]. This self‐digestion process is not only essential for cellular survival, adaptation to nutritional stress, and defense against bacterial and viral infections [13], but also serves as a critical modulator of the ER stress response. ER‐phagy participates in several key physiological processes, including metabolism, immune response, inflammation, and cell proliferation. Consequently, dysfunction of ER‐phagy is closely linked to the development of numerous diseases [14, 15]. DDRGK1–UFL1‐mediated ER‐phagy alleviates ER stress and reduces apoptosis in renal tubular epithelial cells [16]. Conversely, FAM134B receptor‐mediated ER‐phagy exacerbates cisplatin‐induced ER stress, thereby promoting apoptosis of cochlear HCs, as previously observed in cisplatin‐induced ototoxicity [17]. While ER‐phagy confers protection under moderate or prolonged stress, under conditions of excessive stress, it can contribute to cell death by enhancing the degradation of essential ER components, thereby amplifying ER stress [13]. ER stress contributes to the apoptosis of cochlear HCs in NIHL [18]. However, the specific roles and mechanisms through which ER stress and ER‐phagy influence noise‐induced damage remain unclear.

Gastrodin (GAS), the principal bioactive component of *Gastrodia elata* Blume, exhibits significant neuroprotective effects and has shown potential in the treatment and management of various neurological conditions, including neuropathic pain, Alzheimer's disease, and epilepsy [19, 20]. GAS exerts anti‐inflammatory, antiapoptotic, and antioxidative effects primarily through the PI3K/AKT‐Sirt3 signaling pathway [21]. Furthermore, GAS can alleviate diabetic encephalopathy by reducing ER stress [22]. Prior research has demonstrated that GAS triggers autophagy through the AMPK‐Foxo1‐TFEB pathway in foam cells [23]. However, whether GAS treatment regulates ER‐phagy in NIHL has not yet been investigated.

While XIAP is known to promote the survival of cochlear HCs via antiapoptotic pathways [24] and to modulate the sensitivity to ER stress‐ induced cell death [25, 26], its potential involvement in regulating organelle‐specific autophagy, particularly ER‐phagy, remains unexplored. This pathway may represent an additional cytoprotective effect that extends beyond caspase inhibition. This work aimed to clarify the link between XIAP and ER‐phagy, as well as to examine the function and mechanism of XIAP in HC degeneration during NIHL. To this end, we employed hydrogen peroxide (H_2_O_2_)‐induced impairment of HEI‐OC1 cells and noise‐exposed mouse models. We observed an accumulation of reactive oxygen species (ROS) and greater apoptosis in cochlear HCs, a decrease in ER‐phagy activity, and enhanced ER stress in these models. Furthermore, XIAP and DDRGK1 were downregulated under certain conditions. Mechanistically, XIAP could interact with and stabilize the expression of DDRGK1, promoting ER‐phagy. GAS treatment activated the expression of XIAP and DDRGK1 as well as the ER‐phagy pathway, thereby reducing ER stress and apoptosis. Consequently, the modulation of XIAP‐DDRGK1‐mediated ER‐phagy may represent a therapeutic target for averting the degeneration of HCs in NIHL.

## 2 Results

### 2.1 H_2_O_2_‐Induced Apoptosis and Oxidative Stress in HEI‐OC1 Cells

**FIGURE 1:**
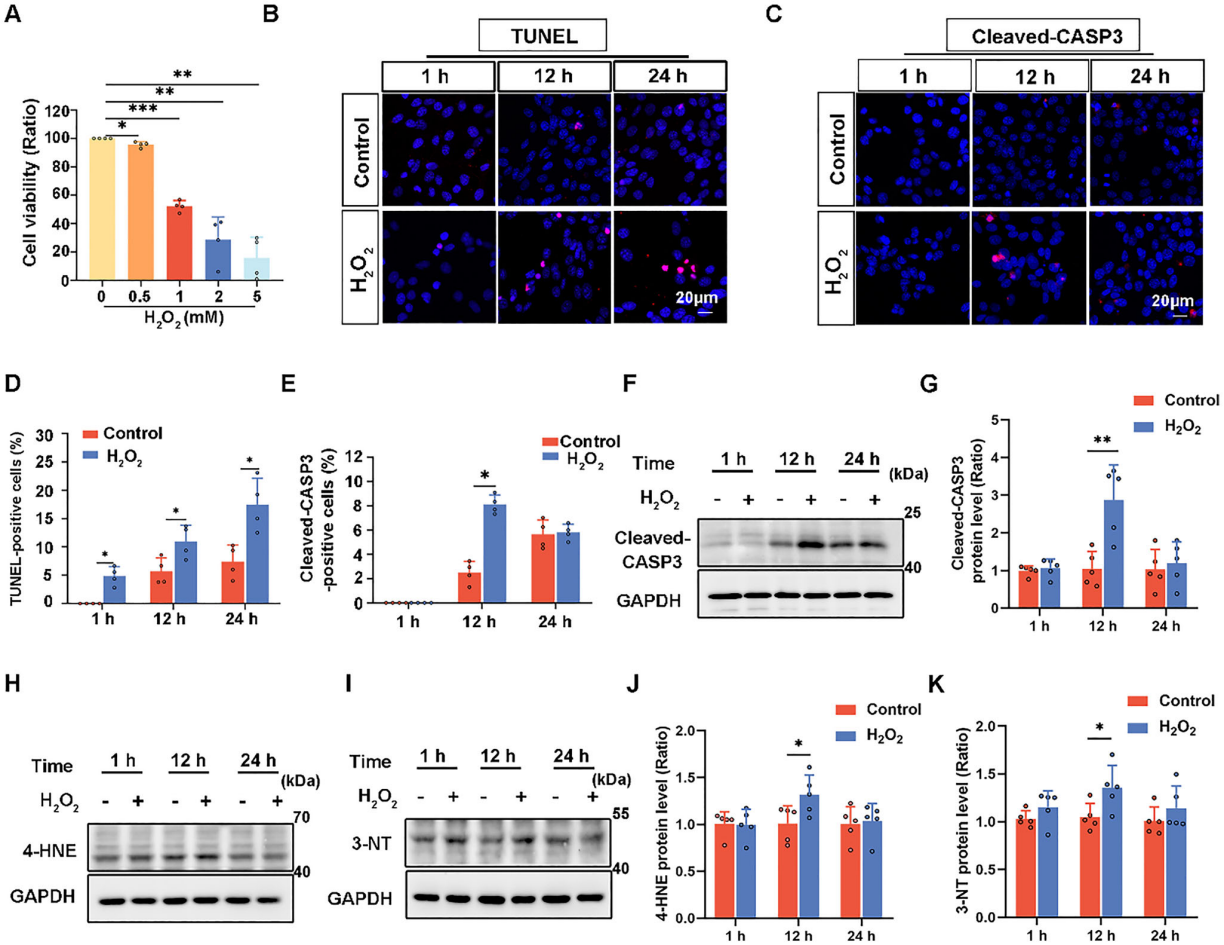
H_2_O_2_ treatment damages HEI‐OC1 cells. (A) Cell viability analysis of HEI‐OC1 cells treated with various concentrations of H_2_O_2_ for 1 h, followed by 24 h of starvation. *n* = 4. (B) Immunofluorescence staining of HEI‐OC1 cells treated with 1 mM H_2_O_2_ for 1, 12, and 24 h using TUNEL Apoptosis Detection Kit. (C) Immunofluorescence staining of HEI‐OC1 cells treated with 1 mM H_2_O_2_ for 1, 12, and 24 h using an antibody against cleaved‐CASP3. (D) Quantification of TUNEL‐positive cells in B. *n* = 4. (E) Quantification of cleaved‐CAPS3‐positive cells in C. *n* = 4. (F) Western blot analysis showing the protein levels of cleaved‐CASP3 in HEI‐OC1 cells treated with 1 mM H_2_O_2_ for different time durations. (G) Quantification of cleaved‐CAPS3 protein levels in F. *n* = 5. (H and I) Western blot analysis showing 4‐HNE and 3‐NT levels in HEI‐OC1 cells treated with 1 mM H_2_O_2_ for different time durations. (J and K) Quantification analysis of western blot bands in H and I, respectively. *n* = 5. Data are expressed as the mean ± standard deviation (SD). Statistical analysis was performed using the Student's t‐test. Statistical significance is indicated as follows: ^*^ *p* < 0.05, ^**^ *p* < 0.01, ^***^ *p* < 0.001.

Excess ROS is believed to significantly contribute to noise‐induced cellular damage [27]. H_2_O_2_, a common ROS and crucial signaling molecule, is frequently employed to investigate the effects of oxidative stress on various physiological processes [28]. Therefore, we treated HEI‐OC1 cells with H_2_O_2_ to simulate noise‐induced oxidative stress. HEI‐OC1 cells were exposed to various concentrations of H_2_O_2_ for 24 h, and their viability was evaluated using the cell counting kit‐8 (CCK‐8). Treatment with 1 mM H_2_O_2_ reduced the cell viability by 50% (Figure 1A). Therefore, we selected 1 mM H_2_O_2_ for subsequent experiments. After treating HEI‐OC1 cells with 1 mM H_2_O_2_, immunofluorescence staining for TUNEL assays and cleaved‐caspase 3 (Cleaved‐CASP3) were conducted to assess apoptosis at 1, 12, and 24 h intervals. A significant increase in both TUNEL‐ and cleaved‐CASP3‐positive cells was observed after 12 h of H_2_O_2_ treatment, when compared with that in the untreated controls (Figure 1B–E). Additionally, cleaved‐CASP3 levels were markedly elevated (Figure 1F,G). Annexin V/PI flow cytometry assay further revealed a significant increase in apoptotic cell populations after 12 h of H_2_O_2_ treatment (Figure S1A,B). Western blot analysis indicated a substantial increase in oxidative stress markers 4‐hydroxynonenal (4‐HNE) and 3‐nitrotyrosine (3‐NT) following 12 h of H_2_O_2_ treatment (Figure 1H–K). These findings indicate that treatment with 1 mM H_2_O_2_ for 12 h induced substantial apoptosis and markedly increased oxidative stress levels in HEI‐OC1 cells.

### 2.2 ER‐Phagy Level Is Decreased in the H_2_O_2_‐Treated HEI‐OC1 Cell Model of Noise Injury

**FIGURE 2:**
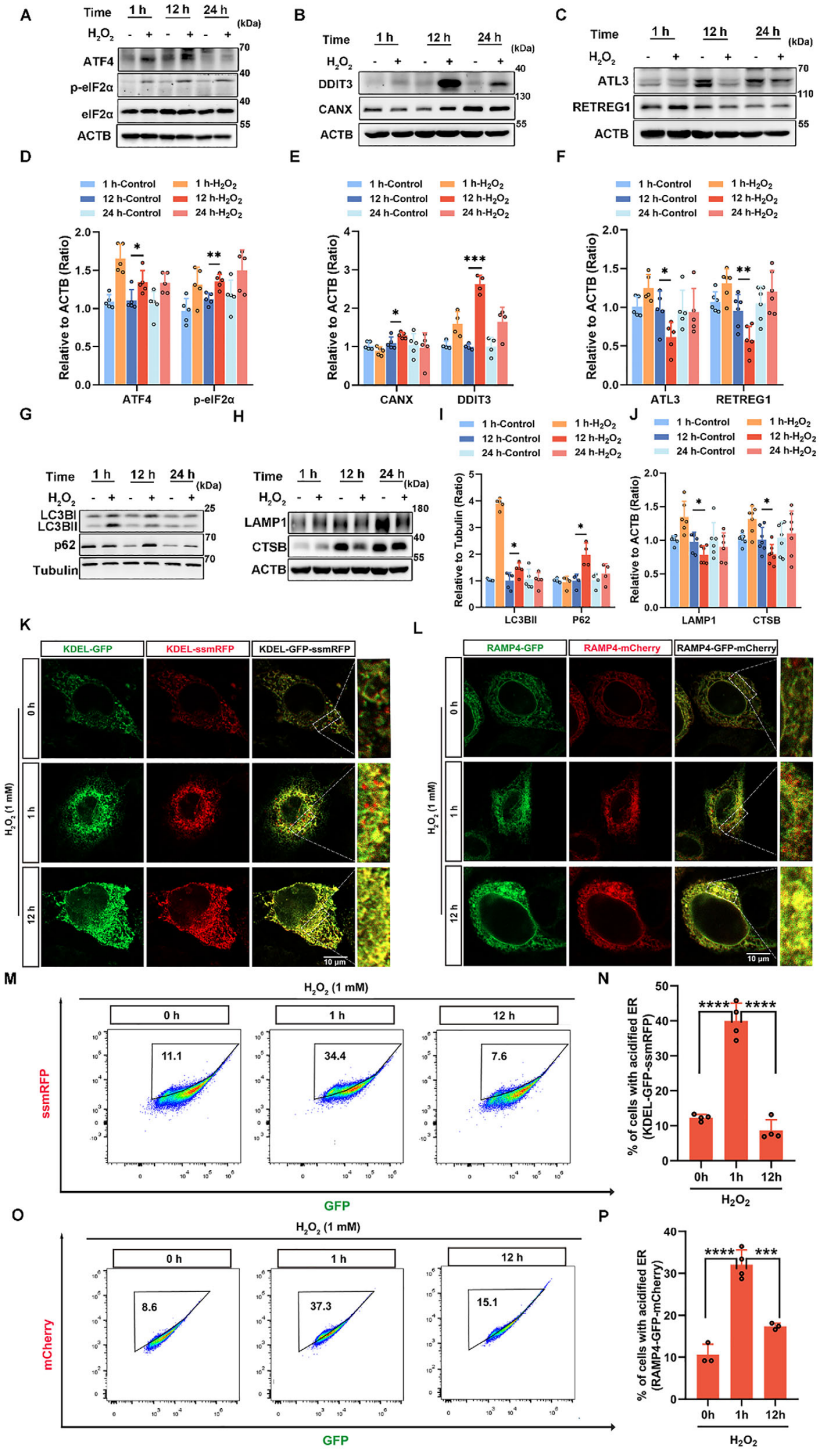
ER‐phagy in HEI‐OC1 cells treated with H_2_O_2_. (A) Western blots showing ATF4, eIF2α, and p‐eIF2α levels in HEI‐OC1 cells treated with 1 mM H_2_O_2_ for different time durations. (B) Western blot showing changes in the levels of DDIT3 and CANX in HEI‐OC1 cells treated with 1 mM H_2_O_2_ for different time durations. (C) Western blot depicting the changes in the expression of RETREG1and ATL3 in HEI‐OC1 cells treated with 1 mM H_2_O_2_ for different time durations. (D–F) Quantification of western blot bands in A‐C. *n* ≥ 4. (G) Western blot showing changes in the expression of LC3B and p62 in HEI‐OC1 cells treated with 1 mM H_2_O_2_ for different time durations. (H) Western blot showing changes in the expression of LAMP1 and CTSB in HEI‐OC1 cells treated with 1 mM H_2_O_2_ for different time durations. (I and J) Quantification of western blot bands in G and H. *n *≥ 4. (K) HEI‐OC1 cells expressing the ER‐phagy reporter (KDEL‐GFP‐ssmRFP) were fixed and observed via fluorescence microscopy at different time points after 1 mM H_2_O_2_ treatment. Scale bars represent 10 µm. (L) HEI‐OC1 cells expressing the ER‐phagy reporter, RAMP4‐GFP‐mCherry, were fixed and observed via fluorescence microscopy at different time points after 1 mM H_2_O_2_ treatment. Scale bars represent 10 µm. (M) HEI‐OC1 cells expressing the ER‐phagy reporter (KDEL‐GFP‐ssmRFP) were detected using flow cytometry at different time points after 1 mM H_2_O_2_ treatment. Cells within the acidified ER gate were identified as undergoing ER‐phagy. (N) Percentage of cells with a more acidified ER, quantified using flow cytometry data in M. *n* = 4. (O) HEI‐OC1 cells expressing the ER‐phagy reporter (RAMP4‐GFP‐mCherry) were detected using flow cytometry at different time points after 1 mM H_2_O_2_ treatment. Cells within the acidified ER gate experience ER‐phagy. (P) Percentage of cells with a more acidic ER, quantified using flow cytometry data in O. *n* = 4. Data are expressed as the mean ± SD. Statistical analyses were performed using the Student's *t*‐test or one‐way ANOVA, as appropriate. Statistical significance is indicated as follows: ^*^ *p* < 0.05, ^**^ *p* < 0.01, ^***^ *p* < 0.001, ^****^ *p* < 0.0001.

ROS induce ER stress, with ER‐phagy acting both as a trigger and a consequence of ER stress responses [29, 30]. To ascertain the function of ER‐phagy in noise‐induced cellular injury, we treated HEI‐OC1 cells with H_2_O_2_ for various time durations. Initially, we observed changes in ER stress that were closely related to ER‐phagy. The expression levels of ATF4 (activating transcription factor 4) and phosphorylated eIF2α (p‐eIF2α) increased over time compared with those in controls (Figure 2A,D), with DDIT3/CHOP (DNA damage inducible transcript 3)—an essential signaling factor in ER stress—showing peak expression after 12 h of H_2_O_2_ treatment (Figure 2B,E). These results indicated that H_2_O_2_ treatment led to a sustained increase in ER stress. We noted a short‐term decrease in the protein expression of calnexin (CANX), an ER membrane marker. However, after prolonged H_2_O_2_ treatment, the CANX levels significantly increased after 12 h (Figure 2B,E). Conversely, ER‐phagy receptors RETREG1/FAM134B (reticulophagy regulator 1) and ATL3 (atlastin GTPase 3) exhibited an early increase in protein levels, followed by a marked decrease at 12 h (Figure 2C,F). These results indicated increased ER accumulation, reflecting impaired ER clearance due to reduced ER‐phagy. To further explore ER‐phagy, we observed changes in the expression of SQSTM1/p62 (sequestosome 1) and MAP1LC3B/LC3B (microtubule‐associated protein 1 light chain 3β), as well as in lysosomal function. At the early stage (1 h post‐H_2_O_2_ treatment), the levels of LC3B‐II, LAMP1, and CTSB were increased (Figure 2G,I), indicating an initial activation of the autophagic process. However, at later stages (12 h and 24 h post‐H_2_O_2_ treatment), we observed a concurrent accumulation of LC3B‐II and p62, coupled with a decrease in LAMP1 and CTSB levels, suggesting a subsequent impairment of autolysosomal degradation (Figure 2H,J). To monitor ER‐phagy flux, we transfected cells with KEDL‐GFP‐ssmRFP and RAMP4‐GFP‐mCherry lentivirus, generating a stable HEI‐OC1 ER‐phagy reporter cell line [31, 32]. These reporters appear yellow in the ER matrix but become red when the reporter is transported to lysosomes via ER‐phagy, as a result of GFP quenching in the acidic environment. RFP/mCherry orphan intensities significantly increased after 1 h H_2_O_2_ treatment but reduced after 12 h H_2_O_2_ treatment (Figure 2K,L). We then quantified the fraction of cells undergoing ER‐phagy using flow cytometry. A notable increase was observed after 1 h H_2_O_2_ treatment, followed by a decrease after 12 h H_2_O_2_ treatment (Figure 2M–P). These results indicated a reduction in the lysosomal degradation capacity, potentially leading to impaired ER‐phagy flux and increased ER stress. Additionally, short‐term increases in ER stress appeared to initially promote ER‐phagy, supporting the maintenance of ER size and homeostasis. However, with sustained and intense stress (noise exposure), ER‐phagy was impaired, particularly at the later stages of autophagy, which led to ER expansion and dysfunction, ultimately causing severe and irreversible cellular damage.

### 2.3 eIF2α Suppresses XIAP Translation and ATF4 Facilitates XIAP Degradation Through Autophagy in H_2_O_2_‐Treated HEI‐OC1 Cells

**FIGURE 3:**
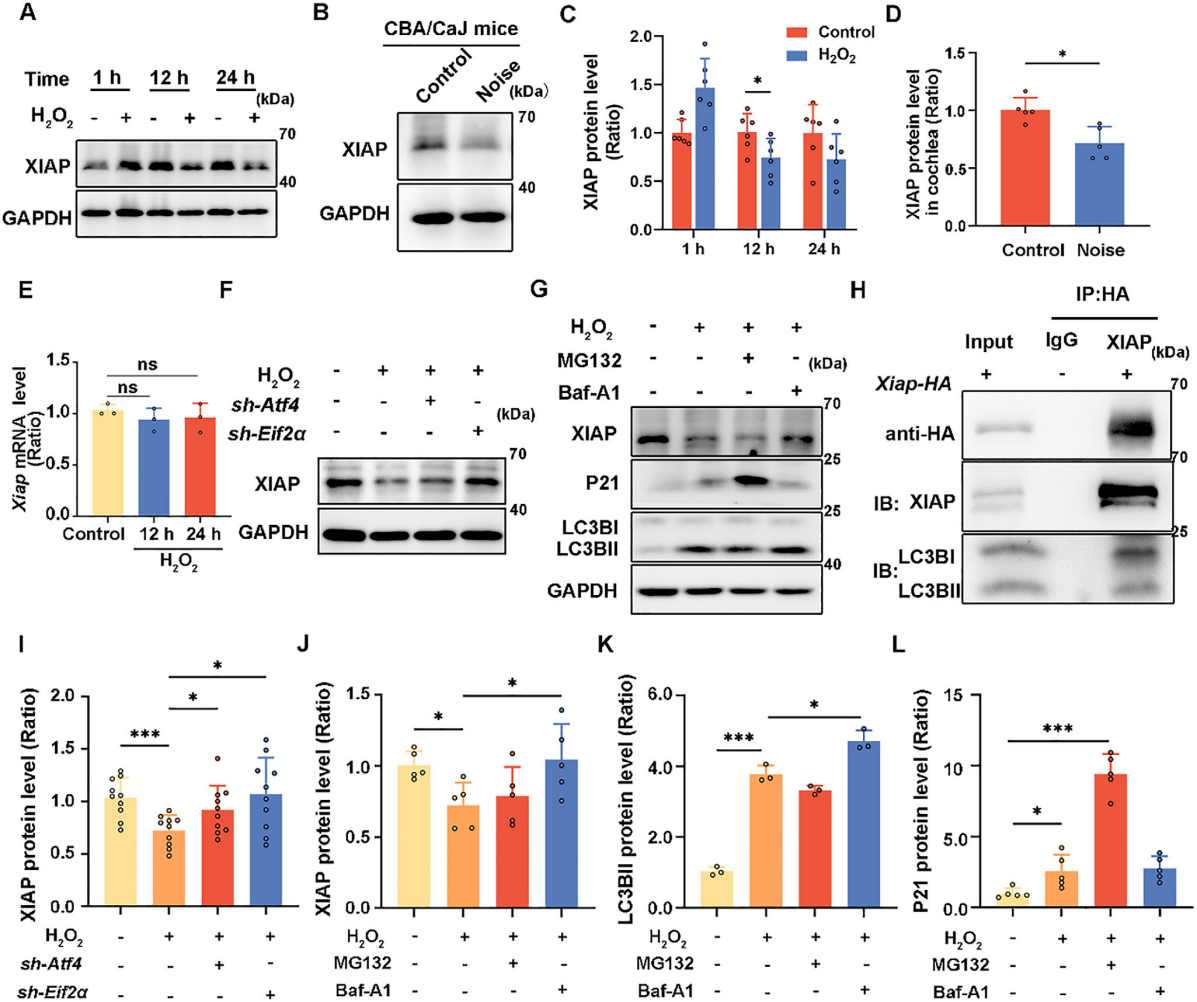
ER stress downregulates XIAP at the protein level but not at the *Xiap* mRNA level. (A) Western blot showing XIAP levels in HEI‐OC1 cells treated with 1 mM H_2_O_2_ for different time durations. (B) Western blot showing XIAP levels in the cochlea following 2 h of 110 dB white noise treatment. (C and D) Quantification of western blot bands in A and B. *n* ≥ 5. (E) *Xiap* mRNA expression in HEI‐OC1 cells treated with 1 mM H_2_O_2_ for different time durations. (F) Western blot showing changes in XIAP levels in HEI‐OC1 cells knocked down for ATF4 or eIF2α and treated with 1 mM H_2_O_2_. (G) Western blot showing changes in XIAP levels following treatment of HEI‐OC1 cells with 1 mM H_2_O_2_ and MG132 (10 µM for 6 h) or bafilomycin A1 (Baf‐A1; 100 nM for 6 h). p21 was used as a positive control for MG132. (H) Western blot showing the interaction of XIAP with LC3B. HEI‐OC1 cells were transfected with *Xiap*‐HA plasmids, and cell lysates were incubated with HA antibody. (I) Quantification of western blots bands in F, *n* = 10. (J–L) Quantification of western blots in G, *n* ≥ 3. Data are expressed as the mean ± SD. Statistical analyses were performed using the Student's *t*‐test or one‐way ANOVA, as appropriate. Statistical significance is indicated as follows: ns: no significance, ^*^ *p* < 0.05, ^***^ *p* < 0.001.

As reported previously, XIAP prevents noise‐induced damage to HCs damage through apoptosis inhibition [24]. eIF2α and ATF4 regulate XIAP at the translational and posttranslational level. We found that the ATF4 and p‐eIF2α expression increased significantly. A significant decrease in XIAP expression was also detected following 12 h treatment with 1 mM H_2_O_2_ (Figure 3A,C). Similarly, CBA/CaJ mice exposed to 110 dB white noise for 2 h exhibited a marked decrease in XIAP protein levels in the cochleae after one day (Figure 3B,D). Moreover, *Xiap* mRNA levels showed no obvious change after H_2_O_2_ treatment for 12 and 24 h, relative to the control levels (Figure 3E). These findings indicated that H_2_O_2_ induced ATF4 and p‐eIF2α upregulation, which promotes XIAP degradation and inhibits its translation, while having no effect on the transcriptional activity of *Xiap*. To confirm this, we knocked down ATF4 and eIF2α using Atf4‐ and eIF2α‐specific shRNAs (Figure S1C–F). The degradation of XIAP was compromised after ATF4 or p‐eIF2α knockdown (Figure 3F,I). To further investigate the pathway of XIAP degradation, MG132 and Bafilomycin A1 were employed to block the ubiquitin‐proteasome pathway and autophagy, respectively. No XIAP degradation was noted in H_2_O_2_‐exposed HEI‐OC1 cells pretreated with Bafilomycin A1. Co‐IP also demonstrated that XIAP interacted with LC3 and was degraded via autophagy (Figure 3G,H,J–L).

### 2.4 XIAP Mitigates Noise‐induced Cell Damage and Oxidative Stress via Activation of ER‐phagy

**FIGURE 4:**
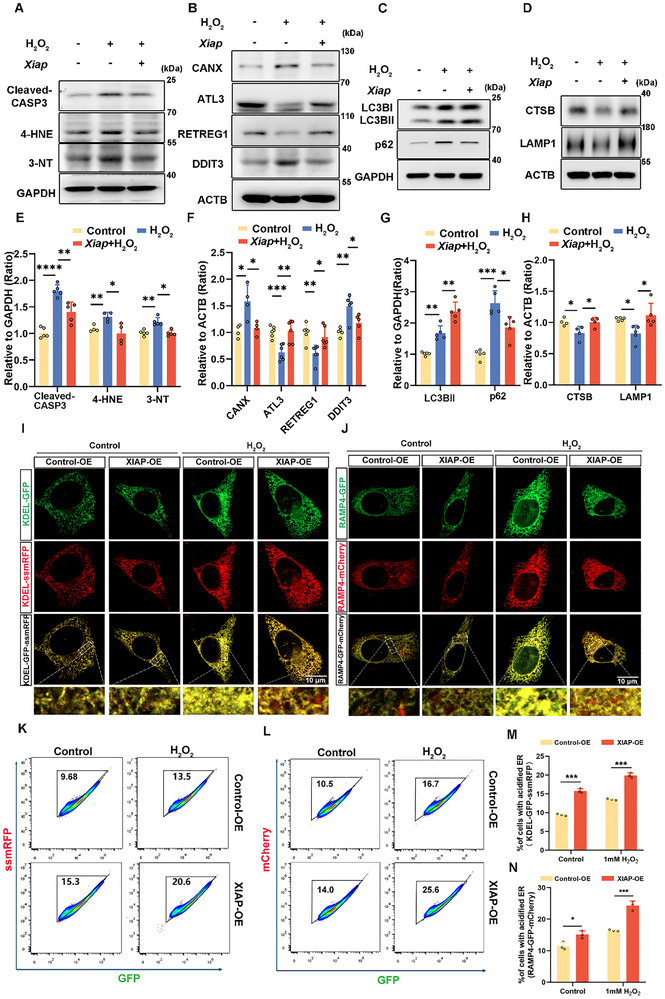
Overexpression of XIAP attenuates apoptosis and oxidative stress in HEI‐OC1 cells by promoting ER‐phagy. (A) Western blot showing changes in the levels of cleaved CASP3, 4‐HNE, and 3‐NT in HEI‐OC1 cells treated with 1 mM H_2_O_2_ for 12 h, with or without prior transfection with the *Xiap*‐HA plasmid for 24 h. (B) Western blot showing the expression levels of CANX, ATL3, RETREG1, and DDIT3 in HEI‐OC1 cells treated with 1 mM H_2_O_2_ for 12 h, with or without prior transfection with the *Xiap*‐HA plasmids. (C and D) Western blot showing alterations in LC3B, p62, CTSB, and LAMP1 levels in HEI‐OC1 cells treated with 1 mM H_2_O_2_, with or without prior transfection with the *Xiap*‐HA plasmid. (E–H) Quantification of western blots bands in A‐D. *n* ≥ 4. (I) Cellular localization of ER‐autophagic flux in HEI‐OC1 cells treated with 1 mM H_2_O_2_ for 12 h, with or without prior transfection with the *Xiap*‐HA plasmid, via fluorescence microscopy using the dual‐reporter (KDEL‐GFP‐ssmRFP). Scale bar represents 10 µm. (J) Cellular localization of ER‐autophagic flux in HEI‐OC1 cells treated with 1 mM H_2_O_2_ for 12 h, with or without prior transfection with the *Xiap*‐HA plasmid, via fluorescence microscopy of the ER‐phagy reporter, RAMP4‐GFP‐mCherry. Scale bars represent 10 µm. (K) Flow cytometry‐based quantitation of ER‐phagic activity in HEI‐OC1 cells expressing KDEL‐GFP‐ssmRFP treated with 1 mM H_2_O_2_ for 12 h, with or without prior transfection with the *Xiap*‐HA plasmid. Cells exhibiting fluorescence within the acidified ER gate were identified as undergoing active ER‐phagy. (L) Flow cytometry‐based quantitation of ER‐phagic activity in HEI‐OC1 cells expressing RAMP4‐GFP‐mCherry treated with 1 mM H_2_O_2_ for 12 h, with or without prior transfection with the *Xiap*‐HA plasmid. Cells exhibiting fluorescence within the acidified ER gate were identified as actively undergoing ER‐phagy. (M) Percentage of cells with an acidified ER, quantified using flow cytometry data in K. *n* = 3. (N) Percentage of cells with an acidified ER, quantified using flow cytometry data in L. *n* = 3. Data are expressed as the mean ± SD. Statistical analyses were performed using the Student's *t*‐test or one‐way ANOVA, as appropriate. Statistical significance is indicated as follows: ^*^ *p* < 0.05, ^**^ *p* < 0.01, ^***^ *p* < 0.001, ^****^ *p* < 0.0001.

To further investigate the role of XIAP in H_2_O_2_‐injured HEI‐OC1 cells, we overexpressed XIAP using an *Xiap*‐HA plasmid and silenced it using *Xiap*‐specific siRNA (Figure S1G–J). Overexpression of XIAP effectively reduced the H_2_O_2_‐induced levels of cleaved CASP3 (Figure 4A,E), and concurrently attenuated apoptosis in HEI‐OC1 cells following H_2_O_2_ treatment (Figure S1K–N). The 3‐NT and 4‐HNE levels were also decreased (Figure 4A). XIAP overexpression reduced the apoptosis and oxidative damage caused by H_2_O_2_ exposure. To clarify the involvement of XIAP in ER‐phagy in H_2_O_2_‐treated HEI‐OC1 cells, we analyzed the expression of ER‐associated proteins and ER‐phagy receptors following XIAP alteration. Upon XIAP overexpression, a notable reduction in the levels of the ER membrane marker CANX was observed along with an increase in the levels of ER‐phagy receptors (Figure 4B,F). Moreover, the levels of ER stress marker DDIT3 were restored (Figure 4B,F). To confirm whether the ER‐phagy flux is affected by the modulation of XIAP expression, we assessed LC3BII, p62, CTSB, and LAMP1 protein levels in H_2_O_2_‐treated HEI‐OC1 cells. Upon XIAP overexpression, LC3BII levels were significantly increased, whereas those of p62 were notably decreased (Figure 4C,G), indicating enhanced autophagic flux. Additionally, lysosomal proteins CTSB and LAMP1 were upregulated (Figure 4D,H), indicating improved lysosomal function and enhanced ER‐phagy.

To confirm the role of XIAP in ER‐phagy, we employed the reporter cell lines. The percentage of cells with an acidic ER after H_2_O_2_ treatment increased significantly under XIAP overexpression (Figure 4I–N). Conversely, knockdown of XIAP in H_2_O_2_‐treated HEI‐OC1 cells led to a further increase in the levels of cleaved‐CASP3 (Figure S2A,E), protein accumulation in the ER, elevated levels of ER stress markers, and impaired autophagy (Figure S2B–D,F–H). These findings indicate that XIAP is essential for the activation of ER‐phagy, which in turn maintains ER homeostasis under noise‐induced stress in HEI‐OC1 cells.

### 2.5 XIAP Promotes ER‐Phagy by Maintaining the Stability of DDRGK1

**FIGURE 5:**
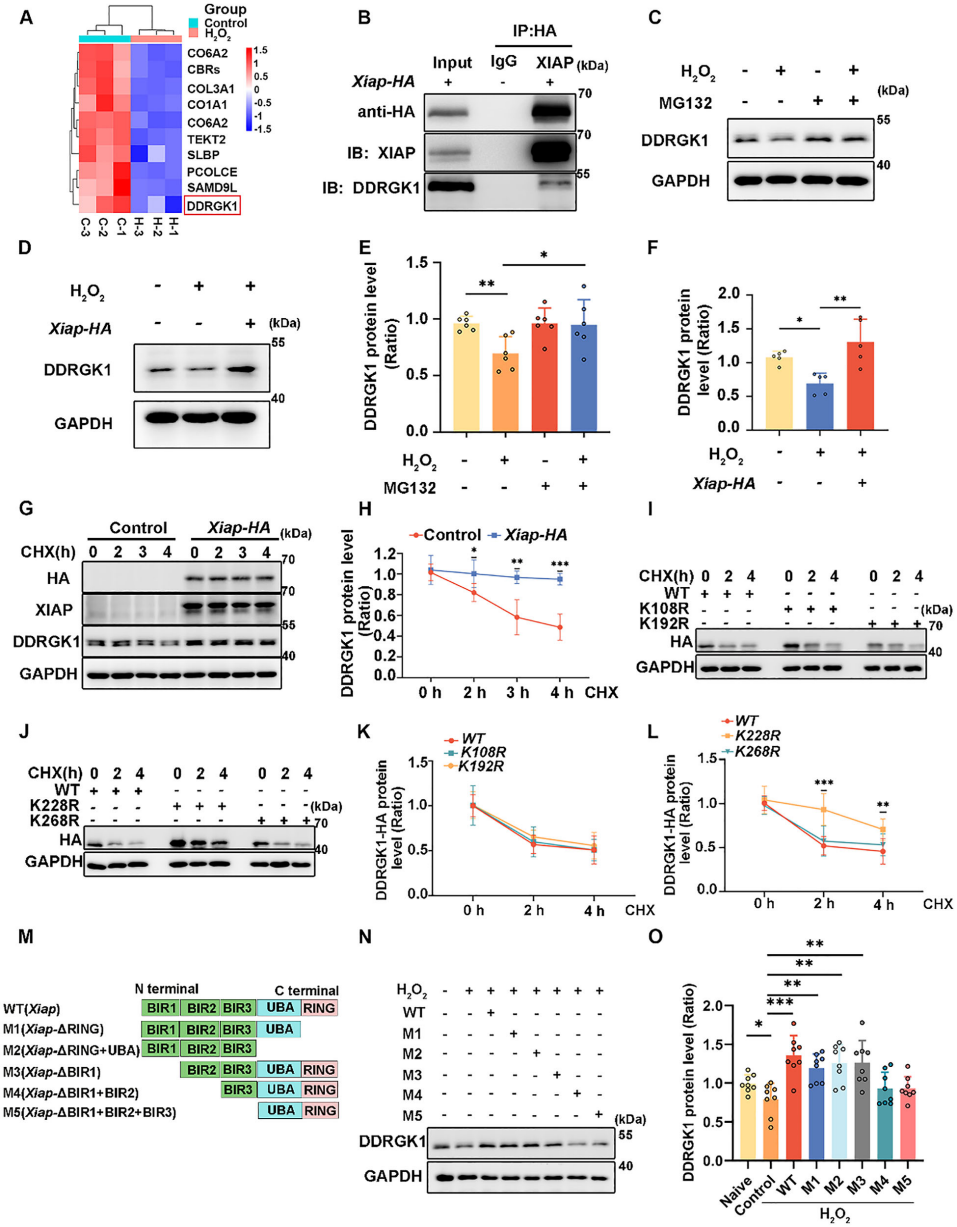
XIAP stabilizes the expression of DDRGK1 protein. (A) Heat map showing differentially expressed proteins, between the 1 mM H_2_O_2_ treatment and control treatment groups of HEI‐OC1 cells, interacting with XIAP. *Xiap*‐HA plasmids were used to transfect HEI‐OC1 cells. (B) Co‐IP analysis indicating the interaction between XIAP and DDRGK1. (C) Western blot showing DDRGK1 expression in HEI‐OC1 cells after 6 h treatment with 10 µM MG132, with or without 1 mM H_2_O_2_. (D) Western blot showing DDRGK1 levels in HEI‐OC1 cells transfected with *Xiap‐HA* plasmids, with or without 1 mM H_2_O_2_ treatment. (E) Quantification of western blot bands in (C). *n* = 6. (F) Quantification of western blot bands in D. *n* = 5. (G) Western blots for evaluating DDRGK1 stability in *Xiap*‐HA transfected HEI‐OC1 cells were treated with cycloheximide (CHX; 10 µg mL^−1^) for various durations. (H) Quantification of DDRGK1 levels in G. *n* = 5. (I and J) Western blots of proteins from HEI‐OC1 cells transfected with *Xiap*‐HA wild type (WT) or mutant plasmids (K108R, K192R, K228R, and K268R), followed by CHX treatment for specified time periods to assess DDRGK1 stability. (K and L) Quantification of DDRGK1‐HA levels in I and J. *n* = 7. (M) Diagram of the XIAP wild type and truncated mutant XIAP protein. (N) Western blot showing the expression of DDRGK1 in cells expressing XIAP mutants treated with 1 mM H_2_O_2_ for 12 h. (O) Quantification of western blot bands in (N), *n* = 8. The data are presented as the mean ± SD, Statistical analyses were performed using the Student's *t*‐test or one‐way ANOVA, as appropriate. Statistical significance is denoted as follows: ^*^ *p* < 0.05, ^**^ *p* < 0.01, ^***^ *p* < 0.001.

Although XIAP is widely recognized for its role in antiapoptotic and autophagy pathways, few studies have explored its involvement in ER‐phagy [10, 33]. To clarify the specific mechanism through which XIAP regulates ER‐phagy, we performed immunoprecipitation(IP)‐mass spectrometry analysis to identify differentially expressed proteins between the control and H_2_O_2_‐treated HEI‐OC1 cells following XIAP overexpression. DDRGK1, an ER‐phagy receptor regulating lysosomal function and maintaining ER homeostasis under stress conditions, was downregulated in the cluster heat map analysis (Figure 5A) [13, 34]. We confirmed the interaction between XIAP and DDRGK1 through co‐immunoprecipitation (Co‐IP) assays (Figure 5B). Moreover, a decrease in DDRGK1 levels was observed in HEI‐OC1 cells after 12 h of H_2_O_2_ treatment (Figure S3A,D). The decrease in DDRGK1 levels was counteracted via administration of proteasome inhibitor MG132 (Figure 5C,E), which indicated that H_2_O_2_‐induced DDRGK1 degradation occurred via the proteasome.

We speculated that XIAP regulates DDRGK1 expression and protects it from degradation. To test this hypothesis, we overexpressed XIAP in HEI‐OC1 cells, which resulted in an upregulation of endogenous DDRGK1 (Figure 5D,F, and Figure S3B,E). Conversely, transfection with XIAP‐targeting siRNA decreased endogenous DDRGK1 levels (Figure S3C,E). To prove that XIAP affects DDRGK1 stability, XIAP‐overexpressing HEI‐OC1 cells were treated with cycloheximide. Western blot analysis indicated that XIAP overexpression prolonged the half‐life of DDRGK1 and directly reduced H_2_O_2_‐induced ubiquitination of DDRGK1 (Figure 5G,H and Figure S3F), suggesting that XIAP inhibits the degradation of DDRGK1 via the ubiquitin‐proteasome pathway. To identify potential sites for XIAP‐mediated stabilization of DDRGK1, we conducted an online database search (BioGRID; https://thebiogrid.org/) and identified K108, K192, K228, and K268 as potential sites on DDRGK1. Indeed, cycloheximide treatment of HEI‐OC1 cells overexpressing the K228R mutant of DDRGK1 confirmed an extended half‐life (Figure 5I–L), and the overexpression of this mutant significantly reduced H_2_O_2_‐induced ubiquitination of DDRGK1(Figure S3G), suggesting that K228 is indeed a key site for the ubiquitination‐mediated degradation of DDRGK1. Moreover, to determine the contribution of XIAP domains to DDRGK1 stability, we generated several XIAP truncations (Figure 5M). The absence of the BIR2 domain in both the M4 and M5 truncations resulted in lower DDRGK1 protein levels (Figure 5N,O, and Figure S3H–I). Furthermore, deletion of the BIR2 domain abolished the interaction between XIAP and DDRGK1(Figure S3J), suggesting that the BIR2 domain of XIAP mediates the regulation of DDRGK1 stability. These findings underscore the importance of the BIR2 domain in modulating protein stability in the XIAP‐DDRGK1 interaction.

**FIGURE 6:**
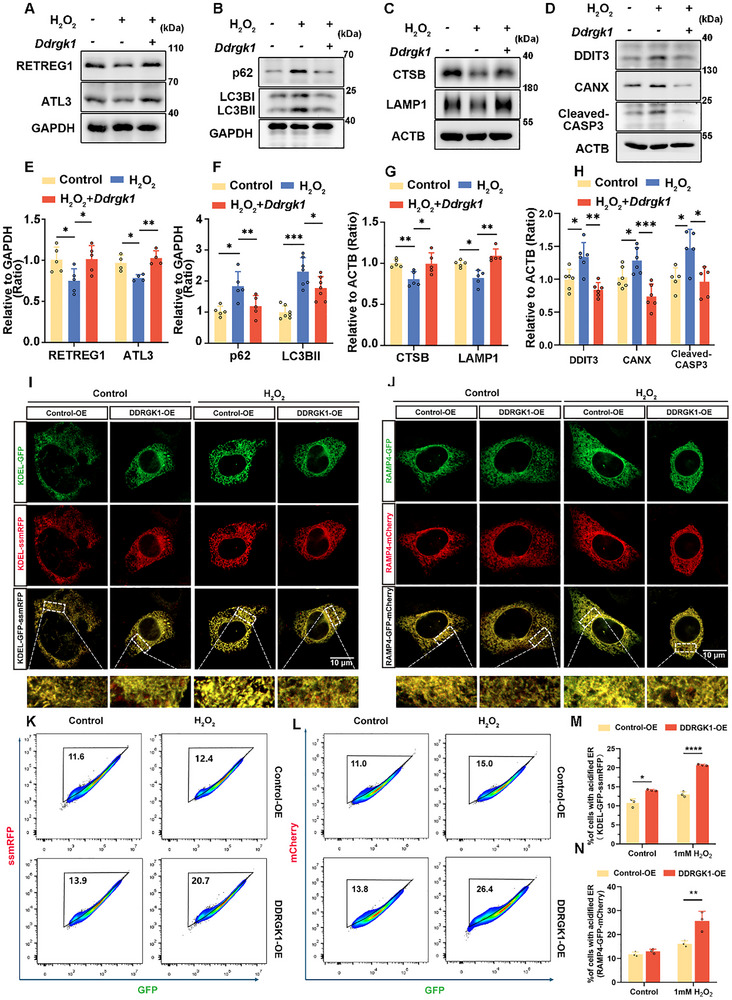
Overexpression of DDRGK1 attenuates apoptosis and oxidative stress in HEI‐OC1 cells by promoting ER‐phagy. (A) Western blot illustrating changes in the levels of ATL3 and RETREG1 in HEI‐OC1 cells treated with 1 mM H_2_O_2_ for 12 h, with or without prior transfection with a *Ddrgk1* overexpression plasmid for 24 h. (B and C) Western blot showing changes in the levels of LC3B, p62, CTSB, and LAMP1 in HEI‐OC1 cells treated with 1 mM H_2_O_2_, with or without prior transfection with the *Ddrgk1* overexpression plasmid. (D) Western blot showing the expression levels of DDIT3, CANX, and cleaved CASP3 in HEI‐OC1 cells treated with 1 mM H_2_O_2_, with or without prior transfection with the *Ddrgk1* overexpression plasmid. (E–H) Quantification of western blots bands in A‐D. *n* ≥ 4. (I) Cellular localization of ER‐autophagic flux in HEI‐OC1 cells treated with 1 mM H_2_O_2_ for 12 h, with or without transient overexpression of DDRGK1, via fluorescence microscopy of the dual‐reporter, KDEL‐GFP‐ssmRFP. Scale bar represents 10 µm. (J) Cellular localization of ER‐autophagic flux in HEI‐OC1 cells treated with 1 mM H_2_O_2_ for 12 h, with or without prior overexpression of DDRGK1, via fluorescence microscopy of the ER‐phagy dual‐reporter, RAMP4‐GFP‐mCherry. Scale bars represent 10 µm. (K) Flow cytometry‐based quantitation of ER‐phagic activity in HEI‐OC1 cells expressing KDEL‐GFP‐ssmRFP, treated with 1 mM H_2_O_2_ for 12 h, with or without DDRGK1 overexpression. Cells exhibiting fluorescence within the acidified ER gate were identified as undergoing ER‐phagy. (L) Flow cytometry‐based quantitation of ER‐phagic activity in HEI‐OC1 cells expressing RAMP4‐GFP‐mCherry, treated with 1 mM H_2_O_2_ for 12 h, with or without DDRGK1 overexpression. Cells exhibiting fluorescence within the acidified ER gate were identified as undergoing ER‐phagy. (M) Percentage of cells with an acidified ER, quantified using flow cytometry data in K. *n *= 3. (N) Percentage of cells with an acidified ER, quantified using flow cytometry data in L. *n* = 3. Data are expressed as the mean ± SD. Statistical analyses were performed using the Student's t‐test or one‐way ANOVA, as appropriate. Statistical significance is indicated as follows: ^*^ *p* < 0.05, ^**^ *p* < 0.01, ^***^ *p* < 0.001, ^****^ *p* < 0.0001.

Finally, to determine the role of DDRGK1‐mediated ER‐phagy in mitigating noise‐induced damage of HCs, we treated DDRGK1‐overexpressing HEI‐OC1 cells with H_2_O_2_ (Figure S3K–L). Western blotting revealed that DDRGK1 upregulation enhances the ER‐phagy activity, as evident from changes in the expression of key markers (Figure 6A–C,E–G) and a concurrent reduction in the levels of ER‐stress indicators (Figure 6D,H). Additionally, cleaved‐CASP3 levels were significantly reduced (Figure 6D,H). Furthermore, fluorescence microscopy and flow cytometry analyses of the reporter line showed greater ER acidification under DDRGK1 overexpression (Figure 6I–N). These results indicated that DDRGK1 plays a protective role in attenuating ER stress and apoptosis by promoting ER‐phagy under oxidative stress. Taken together, our findings suggest that XIAP regulates ER‐phagy by modulating the expression of DDRGK1.

### 2.6 GAS Treatment Protects Cochlea HCs From Noise‐Induced Damage via Upregulating XIAP Expression and ER‐Phagy

In animal models subjected to middle cerebral artery occlusion, GAS promotes the recovery of nerve function by upregulating XIAP expression [35]. To investigate whether GAS prevents H_2_O_2_‐induced damage by stimulating the restoration of ER‐phagy, we first measured HEI‐OC1 cell viability after GAS treatment. We observed significant cytotoxicity after treatment with 500 µM GAS (Figure S4A). Additionally, a marked increase in XIAP levels was noted following treatment with 50 and 100 µM GAS (Figure S4B,C). Accordingly, we hypothesized that GAS treatment upregulates XIAP expression, which subsequently activates ER‐phagy and restores ER homeostasis. To evaluate the effects of GAS treatment on autophagic flux and apoptosis after H_2_O_2_ exposure, LC3BII, p62, and cleaved CASP3 levels in HEI‐OC1 cells were evaluated. GAS treatment resulted in higher LC3BII levels, whereas p62 and cleaved CASP3 levels were reduced (Figure S4D,G), indicating that GAS significantly enhanced H_2_O_2_‐induced autophagic flux and apoptosis. Moreover, the levels of ER membrane proteins were decreased, and those of ER‐phagy receptors increased significantly after GAS treatment (Figure S4E,H). These data revealed that ER‐phagy was restored following GAS treatment. Finally, western blot results also showed that ER stress was significantly alleviated after GAS treatment (Figure S4F,I). Collectively, our data indicate that GAS facilitates the survival of HCs by boosting ER‐phagy via the stimulation of XIAP expression.

**FIGURE 7:**
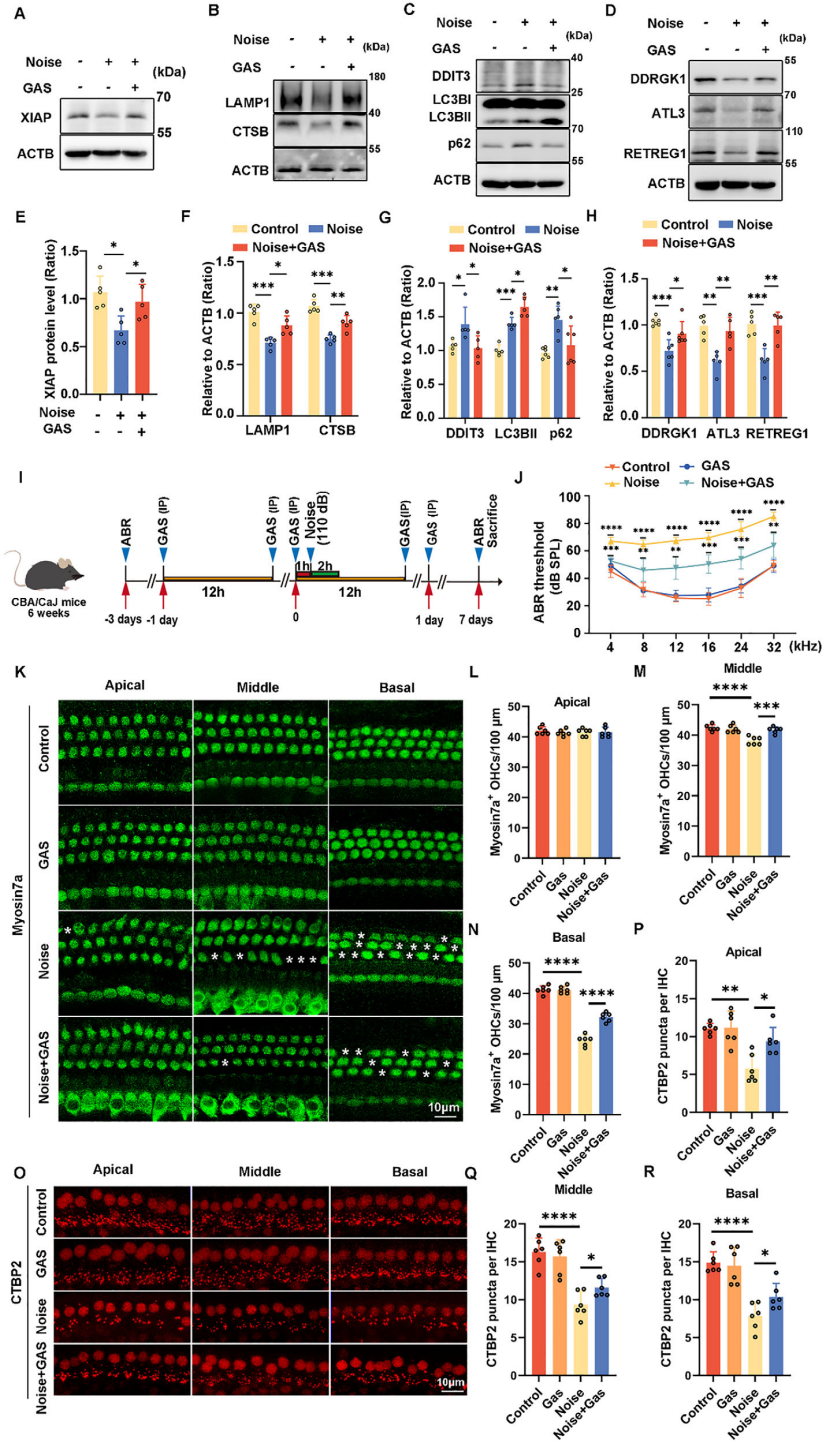
GAS treatment mitigates NIHL, loss of HCs, and synapse damage by enhancing XIAP expression, which activates ER‐phagy. (A) Western blot showing changes in the expression of XIAP in the cochlea after mice were treated with GAS and subjected to noise exposure. (B) Western blots showing changes in the expression of LAMP1 and CTSB in the cochlea. (C) Western blots showing changes in the expression of DDIT3, LC3B, and p62 in HCs. (D) Western blots showing changes in the expression of DDRGK1, ATL3, and RETREG1 in the cochlea. (E–H) Quantification of western blot bands in (A–D). *n ≥ *4. (I) Schematic representation of the experimental setup: six‐week‐old CBA/CaJ mice exposed to 110 dB noise were pretreated with 100 mg kg^−1^ GAS. (J). Auditory brainstem response (ABR) thresholds measured across different treatment groups, indicating hearing preservation in GAS‐pretreated mice. *n* = 6. (K) Immunofluorescence images of myosin7a (Myo7a)‐labeled HCs in the apical, middle, and basal turn of cochlea. (L–N) Quantification of Myo7a‐positive outer HCs (OHCs) from the apical, middle, and basal cochlear region. *n* = 6. (O) Immunofluorescence staining with CTBP2 puncta of the ribbon synapse. (P–R) Quantification of CTBP2 puncta from the apical, middle, and basal turn of the cochlea in (O). *n* = 6. Data are expressed as the mean ± SD. Statistical analyses were performed using the Student's *t*‐test or one‐way ANOVA, as appropriate. Statistical significance is indicated as follows: ^*^ *p* < 0.05, ^**^ *p* < 0.01, ^***^ *p* < 0.001, ^****^ *p* < 0.0001.

We observed that XIAP protected HEI‐OC1 cells from H_2_O_2_‐induced damage by activating ER‐phagy. Consequently, the protective effects of GAS in HCs were associated with XIAP‐mediated regulation of the ER‐phagy pathway. Thus, we further investigated the effects of GAS on protection against hearing loss. To confirm whether GAS activates ER‐phagy through the XIAP‐DDRGK1 axis to protect against NIHL, the cochleae of mice were extracted after noise exposure. Western blotting revealed a substantial increase in XIAP levels and ER‐phagy activity after GAS treatment (Figure 7A–H). Analysis of the ABR revealed a significant reduction in auditory thresholds in the GAS‐pretreated group in comparison with that in the noise exposure‐only group (Figure 7I,J), indicating a protective effect of GAS on hearing function. Analysis using immunofluorescence of myosin7a revealed a lesser loss of HCs in the cochlear middle and basal regions of mice that received GAS pretreatment (Figure 7K–N).

Our findings indicate that NIHL affects the entire frequency of the ABR; however, the damage is localized to the outer HCs in the middle and basal regions of the cochlea. To further investigate this, we assessed synaptic integrity using CTBP2 immunofluorescence. Noise exposure led to a reduction in C‐terminal binding protein 2 (CTBP2) puncta throughout the cochlear region. However, in the group pretreated with GAS, CTBP2 puncta were significantly preserved (Figure 7O–R). These findings highlight the protective role of GAS in maintaining the integrity of cochlear synapses during noise‐induced damage. These results indicated that GAS may play a role in enhancing ER‐phagy through the XIAP‐DDRGK1 axis, potentially contributing to its protective effects against noise‐induced loss of HCs, synapse damage, and hearing loss.

## 3 Discussion

Loss of the ability to regenerate auditory HCs in adult mammals is a critical cause of irreversible damage following noise‐induced HC injury, resulting in hearing loss [36]. The main causes of NIHL include mechanical damage due to noise and excessive oxidative stress [37]. Oxidative stress impairs the functionality of redox‐sensitive organelles, including the mitochondria and ER [38]. This results in the accumulation of misfolded proteins that trigger the ER stress response. ER‐phagy is an important pathway for degrading unfolded or misfolded proteins that are not readily degraded via the unfolded protein response pathway, while also facilitating the restoration of ER morphology [39]. Although activation of ER‐phagy has been reported in cisplatin‐induced hearing loss, the precise mechanism underlying this process remains inadequately understood [17]. This study was aimed at examining the protective effects of ER‐phagy on HCs and the underlying mechanisms related to NIHL.

Our study demonstrates that XIAP‐mediated restoration of ER‐phagy mitigates H_2_O_2_‐induced hair cell damage. This mechanism may have broader relevance for the stability of key structural complexes in HCs, such as those compromised in hereditary hearing loss. For instance, several proteins associated with the Usher syndrome (e.g., harmonin and cadherin‐23) and the tip‐link complex (e.g., protocadherin‐15) are integral to the integrity of the stereocilia bundle. The pathological role of Cdh23 mutations in hearing loss was first established by Han et al. [40], who demonstrated that the erl mutation (recently identified as the cdh23^S47P^ missense mutation within the extracellular domain [41] triggers apoptosis of inner ear HCs, with apoptosis inhibition conferring hearing protection in mutant mice [40]. Notably, the cdh23^S47P/erl^ mutation exhibits only minor structural alterations in the PCDH15‐CDHR23 complex interface without disrupting its folding or heterophilic binding capacity in vitro [41]. This is consistent with the observation that cdh23^erl/erl^ mice exhibit normal hair cell tip‐link structure and complete hearing function before 1 month of age. The delayed onset of progressive hearing loss and hair cell degeneration (commencing at ∼1 month and culminating in deafness by 3 months) cannot therefore, be attributed to a primary structural defect in the tip‐link complex. Instead, Hu et al. first identified persistent ER stress as the upstream molecular driver of this apoptotic cascade [42]. Subsequently, Hu et al. demonstrated that targeted ER stress inhibition using salubrinal (an ER stress suppressor) [42], tauroursodeoxycholic acid (TUDCA, suppressing ER stress‐mediated caspase‐3 activation) [43], or 4‐phenylbutyrate (4‐PBA, a chemical chaperone downregulating ER stress markers) [41] effectively rescued the survival of HCs in cdh23^erl/erl^ mice. Collectively, this study established that the primary pathology in cdh23^S47P/erl^‐associated hearing loss is ER stress‐induced apoptosis, not structural failure of the tip link, validating ER stress modulation as a key otoprotective strategy.

We investigated the role of ER‐phagy in NIHL and elucidated how XIAP restores ER homeostasis by modulating ER‐phagy, thereby potentially mitigating NIHL. Initially, we observed increased protein levels of ER stress markers, including ATF4, p‐eIF2α, and DDIT3, which indicated activation of ER stress, following 12 h of H_2_O_2_ treatment (Figure 2A,B,D,E). Additionally, expression of the ER membrane marker CANX increased, whereas that of the ER‐phagy receptors RETREG1 and ATL3 decreased (Figure 2B,C,E,F), indicating the accumulation of damaged ER and impairment of ER‐phagy. Subsequently, we noted an elevation in LC3BII and p62 levels alongside decreased lysosomal function (Figure 2G–J). These findings imply that noise exposure leads to a reduction in the ER‐phagy flux, resulting in compromised downstream processes associated with ER‐phagy and inhibition of damaged ER degradation. Following 12 h H_2_O_2_ treatment, XIAP expression was diminished (Figure 3A,C), which may contribute to the obstruction of ER‐phagy flux. Upon XIAP overexpression, we observed reduced ER stress levels and enhanced removal of damaged ER. Moreover, ER‐phagy flux was increased, resulting in a reduction in apoptosis (Figure 4). Collectively, these data suggest that XIAP may mitigate ER stress‐induced damage by enhancing ER‐phagy. This indicates a potential regulatory role for XIAP in modulating ER‐phagy in noise‐induced damage models.

We noted a decrease in XIAP levels in the NIHL models (Figure 3A–D), although *Xiap* mRNA levels remained unchanged after 12 h (Figure 3E). The mechanism through which noise exposure decreases XIAP levels warrants further investigation. Previous studies have indicated that ER stress can suppress XIAP expression via eIF2α, which downregulates XIAP synthesis and promotes its degradation through ATF4 [25]. A substantial elevation in eIF2α and ATF4 levels was confirmed (Figure 2A,D). Moreover, knockdown of *Atf4* and *Eif2α* restored XIAP expression in H_2_O_2_‐injured HEI‐OC1 cells (Figure 3F,I), suggesting that eIF2α and ATF4 are crucial in regulating XIAP levels. Additionally, we found that XIAP undergoes degradation via the autophagy pathway, as evidenced through its interaction with LC3B (Figure 3G,H,J–L).

DDRGK1, an ER‐phagy receptor, plays a crucial role in regulating ER‐phagy. Our data revealed a notable decrease in DDRGK1 levels following H_2_O_2_ exposure (Figure S3A,B). XIAP was found to interact with DDRGK1, and modulation of XIAP affected DDRGK1 levels (Figure 5B,D,F and Figure S3C–E), suggesting a regulatory relationship whereby XIAP modulates DDRGK1 expression. Further experiments confirmed that XIAP enhanced DDRGK1 stability through the BIR2 domain (Figure 5M–O and Figure S3H–J). However, no difference was observed between the effects of M4 and M5 truncations (Figure 5M–O), suggesting that BIR3 domain of XIAP does not influence the regulation of DDRGK1 stability. Thus, we believe that the BIR2 domain of XIAP may be more critical for DDRGK1 regulation. An important question arising from this study concerns how the XIAP/DDRGK1 axis interfaces with the canonical ER‐phagy machinery. DDRGK1 is known to recruit the UFL1 ligase complex to facilitate the ufmylation of ER‐resident proteins, which can serve as a signal for ER‐phagy receptor recruitment and autophagosome formation around ER domains [44]. Our finding that XIAP stabilizes DDRGK1 suggests a model whereby XIAP ensures the sufficient levels of DDRGK1 required for efficient UFL1 complex assembly and subsequent ER‐phagy initiation. It is plausible that DDRGK1 stabilization does not operate in isolation but may act synergistically with or regulate key ER‐phagy receptors such as FAM134B [45, 46]. For instance, XIAP‐mediated stabilization of DDRGK1 could potentially enhance the ufmylation and activity of other ER‐phagy receptors or core components, thereby coordinating a multi‐faceted ER‐phagy response. This proposed crosstalk significantly enhances the novelty of our model, suggesting that XIAP does not merely influence a linear pathway but acts as a central integrator that fine‐tunes the entire ER‐phagy network. Furthermore, by simultaneously inhibiting apoptosis via caspase suppression and promoting ER‐phagy through DDRGK1 stabilization, XIAP implements a dual cytoprotective strategy. This positions XIAP as a critical sentinel at the crossroads of ER quality control and apoptotic signaling, a role that has not been previously elucidated. Our study, therefore, provides a novel conceptual framework for understanding how cells coordinate organelle‐specific autophagy with survival pathways to mitigate stress‐induced damage.

Our data demonstrate that XIAP, through its BIR2 domain, interacts with DDRGK1 and reduces its polyubiquitination, thereby shielding it from proteasomal degradation. As a well‐characterized E3 ubiquitin ligase, [47, 48], XIAP may exert this stabilizing effect by competing with other E3 ligases for DDRGK1 binding. Future studies are needed to identify the specific E3 ligases responsible for DDRGK1 degradation under stress conditions and to delineate the precise nature of the ubiquitin code modulated by XIAP.

GAS, the primary bioactive component of *Gastrodia elata* Blume, has been demonstrated to exert significant therapeutic effects on the brain and nervous system. It is particularly effective in treating neurodegenerative diseases, emotional disorders, and cognitive impairments via the activation of autophagy, consistent with its reported effects in other neurological models [49]. Furthermore, GAS enhances perioperative cardiac protection by facilitating mitophagy through the PINK1/Parkin pathway, hence mitigating myocardial ischemia‐reperfusion injury [50]. GAS exerts protective effects against oxidative stress‐induced injury by promoting autophagy and phagocytosis via the PPARα‐TFEB/CD36 signaling pathway [51]. Although GAS demonstrates potential advantages in the management of acute sensorineural hearing loss, the detailed molecular mechanisms remain unclear [52]. Our findings indicate that GAS protects cochlear HCs by enhancing the expression of XIAP‐DDRGK1 and restoring ER‐phagy, which collectively reduce apoptosis and ER‐stress in cochlear HCs and offer protection against NIHL (Figure 7, and Figure S4), providing strong evidence endorsing the prospective therapeutic utilization of GAS for auditory protection.

## 4 Conclusions

**FIGURE 8:**
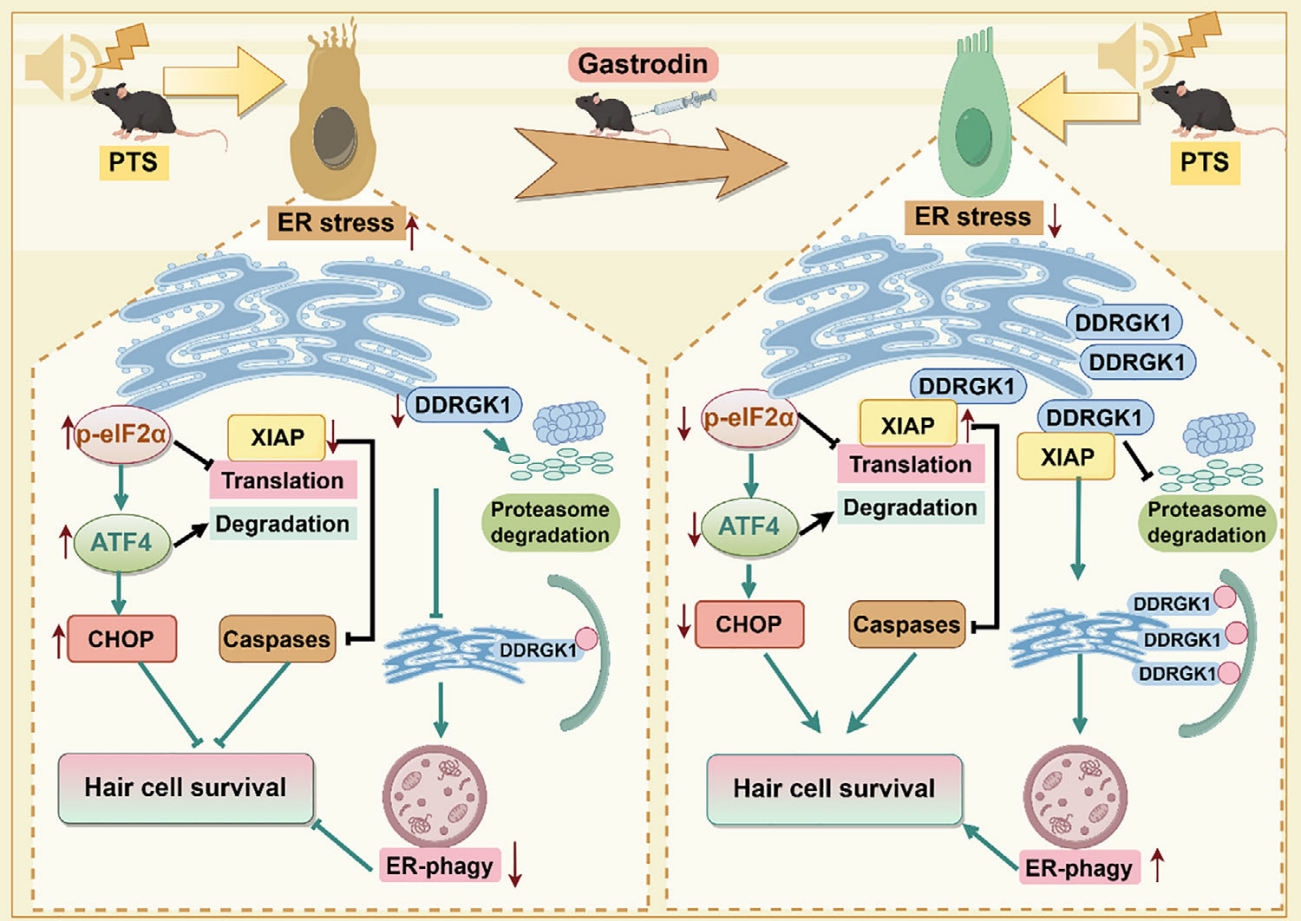
Mechanisms underlying the effect of XIAP on DDRGK1‐mediated ER‐phagy in NIHL.

We have demonstrated that DDRGK1‐ER‐phagy plays a role in noise‐induced death of HCs. We have also shown that noise exposure inhibits ER‐phagy by downregulating XIAP via the activation of ATF4 and eIF2α, which in turn promotes DDRGK1 degradation. GAS treatment restores XIAP and DDRGK1 expression, activating ER‐phagy and thereby mitigating noise‐induced loss of HCs, synaptic damage, and hearing loss caused by noise (Figure 8). Our findings provide novel therapeutic targets and a theoretical basis for the treatment of NIHL.

## 5 Experimental Section

### 5.1 Animals and Ethical Statement

Four‐week‐old CBA/CaJ mice were obtained from Beijing SPF Biotechnology Co., Ltd. Prior to the experiments, mice were acclimatized for one week in an enclosed space with a controlled temperature (22 ± 1°C) and a 12 h light/dark cycle and provided unrestricted access to water and food. Thereafter, the mice were randomly assigned to either the control or simulated noise‐exposure group. All experimental protocols were approved by the Institute of Health and Medicine, Hefei Comprehensive National Science Centre (IHM‐AP‐2024‐032), and measures were implemented to reduce the number of animals utilized and to mitigate suffering.

### 5.2 Noise Exposure

Six‐week‐old male mice were individually kept in stainless‐steel wire cages (approximate volume: 9 cm^3^) for the study. The noise‐exposed group was subjected to white noise at 110 dB for 2 h to produce a permanent threshold shift. The noise exposure chamber was equipped with JBL 2450H speakers, amplified by a Crown Audio XLS 202D amplifier, and controlled via a Tascam TEAC American CD‐200 CD player. Noise was generated using Adobe Audition 3, and sound levels were verified with a Quest Technologies 1200 sound level meter at multiple chamber locations to ensure field uniformity and stability, both before and after exposure. The control mice were kept in the chamber for 2 h without exposure to auditory stimuli [53]. All ABR measurements were performed by a single experimenter, and threshold values were determined by specialists blinded to the treatment conditions.

### 5.3 Auditory Brainstem Response Measurement

ABR were assessed in both ears of anesthetized mice before and one week following noise exposure. Mice were anesthetized via an IP injection of pelltobarbitalum natricum (100 mg/kg) and positioned in acoustically and electrically shielded compartments (Acoustic Systems). Body temperature was observed and regulated at approximately 35°C using a heating pad. Subcutaneous electrodes were placed on the skull apex (active), below the left ear mastoid (reference), and below the right ear mastoid (ground). The ABR recordings were obtained at frequencies of 4, 8, 12, 16, 24, and 32 kHz.The Tucker–Davis Technologies System III hardware, in conjunction with the SigGen/Biosig software, was employed to present the stimulation (a 15 ms tone burst with a 1 ms rise–fall duration) and capture the responses. An average of 1,024 responses per stimulus level was collected. ABR wave I was employed to ascertain the threshold at each frequency, computed by decrementing the intensity in 10 dB intervals, followed by 5 dB changes near the threshold, until no structured response was observed. The threshold was defined as the midpoint between the minimum stimulus level eliciting a detectable reaction and the maximum level that does not provoke a response. All ABR measurements were performed by a single experimenter, and threshold values were determined by specialists blinded to the treatment conditions.

### 5.4 Animal Procedure

In vivo experiments were performed using a mouse model of NIHL. Adult CBA/CaJ mice were randomly assigned to one of three groups: control group, noise exposure group, or noise exposure combined with GAS treatment group. GAS was administered via intraperitoneal injection at a dose of 100 mg kg^−1^ every 12 h for two doses, with an additional dose delivered 1 h prior to noise exposure. Mice in both the noise exposure and GAS treatment groups were exposed to continuous broadband noise at 110 dB SPL for 2 h. In the treatment group, GAS administration was repeated at 12 and 24 h postnoise exposure. Auditory function was assessed by measuring ABR thresholds 3 days before noise exposure and 7 days after exposure. Subsequently, cochlear tissues were collected for histological and biochemical analyses.

### 5.5 Immunofluorescence of the Cochlea

The temporal bone was removed and perfused topically with 4% paraformaldehyde (Solarbio, P1110) in PBS solution (pH 7.4) and stored overnight in this fixative at 4°C. Following a 4–6 h decalcification process in 4% EDTA sodium solution (pH 7.4, adjusted with HCl) at 20°C, the cochlea was segmented into three regions—apical, middle, and basal. To facilitate sample preparation, Cell‐Tak (Corning, 354240) was applied to 8 mm circular cover glasses (CITOTEST, 80344–0820). The samples were permeabilized with PBST for 15 min, followed by blocking with 10% donkey serum (Solarbio, SL050) for 1 h. The samples were subsequently treated overnight at 4°C with primary antibodies, and DAPI (Solarbio, C0060) was used for nuclear staining. After three 5‐min washes in PBST, the samples were incubated with secondary antibodies at room temperature for 1 h. Following a minimum of three final rinses with PBST, all immunolabeled samples were mounted onto circular cover slides using 8 µL of sealer (Beyotime, P0131), sandwiched with another circular cover slide, and placed on a microscope slide. The edges were secured with nail polish. Immunofluorescence images were captured using a ×63 magnification lens on a Zeiss LSM 900 microscope.

### 5.6 Cell Culture, Cell Transfection, and Drug Treatment

HEI‐OC1 cells were grown in DMEM supplemented with 10% FBS (EallBio, 03. U16001DC) and 50 µg mL^−1^ ampicillin (Sigma‐Aldrich, A0166) at 37°C in a 5% CO_2_ environment. The cells were dislodged from the culture vessel with 0.25% trypsin‐EDTA (Thermo Fisher Scientific, 25 200 054). At 60–70% confluence, the cells were transfected with the plasmids or siRNA using Opti‐MEM (Gibco, 31,985,062) and Lipofectamine 2000 transfection reagent (Invitrogen, 11,668,027). All the plasmids and siRNA sequences utilized in this work were listed in the Supporting Information (Table S1). Following transfection, the HEI‐OC1 cells were cultured in FBS‐free medium containing 1 mM H_2_O_2_ (RETOUCH, RTQ03‐8‐22‐0723‐10) for 12 h and then collected for western blot analysis. Additionally, the cells were seeded in 6‐well culture plates treated with 100 µM GAS for 24 h after they achieved 70% confluence. The control group received an equivalent amount of dimethyl sulfoxide. Subsequently, each group was treated with 1 mM H_2_O_2_ and GAS for 12 h.

### 5.7 Immunofluorescence and Flow Cytometry of HEI‐OC1 Cells

HBLV‐ KDEL‐GFP‐ssmRFP and HBLV‐ RAMP4‐GFP‐mCherry lentivirus were purchased from HanBio Technology (Shanghai, China). HEI‐OC1 cells were infected with these lentiviruses to establish stably expressing cell lines. Following induction of oxidative stress with 1 mM H_2_O_2_, ER‐autophagic flux was assessed at different time points by monitoring the KDEL‐GFP‐ssmRFP or RAMP4‐GFP‐mCherry. For the rescue study, HEI‐OC1 cells were transiently pre‐transfected to overexpress XIAP or DDRGK1 for 24 h, followed by exposure to 1 mM H_2_O_2_ for 12 h. Cells were either fixed and observed under a fluorescence microscope (ZEISS, LSM 980) or trypsinized, resuspended in complete medium without fixation, and immediately assessed via flow cytometry (Beckman, CytoFLEX S). The yellow puncta represented ER‐autophagosomes, and the red only puncta represented ER‐autolysosomes (acidified ER). The extent of ER autophagy activity was quantitatively assessed by determining the proportion of cells exhibiting red‐shifted fluorescence within the acidified ER gate.

### 5.8 Cell Vitality Assay

HEI‐OC1 cells were inoculated into a 96‐well plate. After the cells attached to the well surface, they were subjected to a H_2_O_2_ concentration gradient. Subsequently, the cells were incubated with the CCK‐8 reagent (Beyotime, C0042) for 1 h. The absorbance was recorded at 450 nm using a Thermo Fisher Scientific Varioskan LUX multimode microplate reader.

### 5.9 RNA Isolation and RT‐qPCR

Cells from HEI‐OC1 cells were cultured in a Petri dish for RT‐qPCR analysis. Total RNA was isolated using the TRIzol reagent (Life Technologies, 15 596‐018). Reverse transcription was performed using the RevertAid First Strand cDNA Synthesis Kit (Thermo Fisher Scientific, K1622). The primers used for RT‐qPCR are listed in the Table S1. An Applied Biosystems CFX96 real‐time PCR system (Bio‐Rad, Hercules, USA) was used to conduct RT‐qPCR, following the method described in a previous report [53]. The mRNA levels were normalized against *Gapdh* levels, and relative gene expression was calculated using the comparative cycle threshold method (ΔΔCt) [54].

### 5.10 Western Blotting and Reagents

The lysis of HEI‐OC1 cells were lysed in RIPA buffer (Beyotime, P0013B) containing a protease inhibitor cocktail (Roche, 04,693,132,001). Protein concentrations were determined using the Pierce BCA Protein Assay Kit (Beyotime, P0009) according to the manufacturer's protocol. Proteins were separated by electrophoresing on a sodium dodecyl sulfate‐polyacrylamide gel and then transferred onto polyvinylidene difluoride membranes (Millipore, IPVH00010). The membranes were blocked with a buffer containing 5% skimmed milk powder and incubated overnight with the primary antibody at 4°C. They were subsequently incubated with the secondary antibody, which was applied for 1 h at 25°C. The detection and analysis of the blotted proteins were carried out using a chemiluminescence detection system and the ImageJ software.

### 5.11 Antibodies

The following antibodies were used in this study: Anti‐myosin7a (Proteus Bioscience, 25–6790); GAPDH (Proteintech, 60004‐1‐Ig); α‐tubulin (Proteintech, 66031‐1‐Ig); anti‐p‐eIF2α (Cell Signaling Technology, 9721); anti‐eIF2α (Cell Signaling Technology, 9722); anti‐CHOP/DDIT3 (Cell Signaling Technology, 5554); anti‐calnexin (Abcam, ab22595); anti‐ATL3 (Proteintech, 16921‐1‐AP); anti‐DDRGK1 (Proteintech, 21445‐1‐AP); anti‐LC3B (Cell Signaling Technology, 2775); anti‐ATF4 (Proteintech, 60035‐1‐Ig); anti‐SQSTM1/p62 (Cell Signaling Technology, 5114); anti‐LAMP2 (Invitrogen, PA1‐655); anti‐LAMP1 (Abcam, ab208943); anti‐CTSB (Cell Signaling Technology, 31718); 4‐HNE (Abcam, ab46545); 3‐NT (Sigma‐Aldrich, N0409); cleaved caspase‐3 (Cell Signaling Technology, 9661); β‐actin (Proteintech, 20536‐1‐AP); anti‐XIAP antibody (Proteintech, 66800‐1‐Ig); anti‐FAM134B/RETREG1 (Proteintech, 21537‐1‐AP); Alexa Fluor 488‐conjugated Donkey Anti‐Rabbit IgG (Invitrogen, A‐21206); Alexa Fluor 488‐conjugated Donkey Anti‐Mouse IgG (Invitrogen, R37114); Alexa Fluor 555‐conjugated donkey anti‐rabbit IgG (Invitrogen, A‐31572); Alexa Fluor 555‐conjugated donkey anti ‐mouse IgG (Invitrogen, A‐31570); mouse anti‐CTBP2 IgG1 (BD Biosciences, 612044); Alexa Fluor 568‐conjugated Goat Anti‐Mouse IgG1 (Invitrogen, A21124).

### 5.12 Statistical Analysis

All quantitative experiments were performed with at least three independent replicates (*n* ≥ 3). Data are presented as the mean ± standard deviation (SD). No specific data transformation was applied, and no outliers were excluded from the analyses. The sample size ‘n’ for each experiment, representing the number of independent biological replicates, is specified in the corresponding figure legends. Statistical analyses were performed using the GraphPad Prism software (version 8.0). To assess significant differences between two groups, an unpaired, two‐tailed Student's *t*‐test was used. For comparisons across multiple groups, one‐way analysis of variance (ANOVA) was applied, followed by Tukey's posthoc test for multiple comparisons. A *p*‐value < 0.05 was considered to indicate a statistically significant difference. All statistical tests were two‐sided, and the assumptions of the tests (e.g., normal distribution for ANOVA) were verified.

## Author Contributions

L. Y., Y. Z., J. D., and Y. Z. contributed equally as first authors to this work. R.J., J. Y., P. L., and Q. F. conceptualized, supervised, and designed this project. L. Y., Y. Z., J. D., and Y. Z. performed project administration, investigation, data curation, and writing – original draft. W. C., Y. W., and H. W. helped review and editing the draft. H. W., S.Q., S. P., and L. C. executed some data curation and cell experiments.

## Conflicts of Interest

The authors declare no conflicts of interest.

## Supporting information




**Supporting File 1**: advs73976‐sup‐0001‐SuppMat.docx.

**Supporting File 2**: advs73976‐sup‐0002‐SuppMat.zip.

## Data Availability

The data that support the findings of this study are available from the corresponding author upon reasonable request.
