# Supplementary material for: XIAP Stabilizes DDRGK1 to Promote ER‐Phagy and Protects Against Noise‐Induced Hearing Loss
Source: Adv Sci (Weinh). 2026 Jan 26;13(18):e11217. doi: 10.1002/advs.202511217 (PMC13042907; doi:10.1002/advs.202511217)
Supplement: Supplementary file 1 — Supporting File 1: advs73976‐sup‐0001‐SuppMat.docx. [file ADVS-13-e11217-s001.docx]

**Supporting Information**

**XIAP Stabilizes DDRGK1 to Promote ER-phagy and Protects Against Noise-Induced Hearing Loss**

Lin Yan^#^, Yuhua Zhang^#^, Jiawei Du^#^, Yongjun Zhu^#^, Wei Cao, Yongjie Wei, Han Wu, Shiyu Qiu, Shiyi Pan, Lian Chen, Pingping Liang*, Renjie Chai*, Jianming Yang*, Qiaojun Fang*

**Materials and Methods**

*Plasmids*

*Xiap*-HA, *Ddrgk1*-HA (WT), *Ddrgk1*-HA (K108R), *Ddrgk1*-HA (K192R), *Ddrgk1*-HA (K228R), and *Ddrgk1*-HA (K268R) were synthesized by Beijing Tsingke Biotech Co., Ltd. *Xiap*-*Flag* (WT) was generated by inserting the XIAP fragment into pAAV- CAG-3×Flag vector, *Xiap*-Flag–ΔRING (delete 1297-1491nt, M1), *Xiap*-Flag -ΔRING+UBA (delete 1105-1491nt, M2), *Xiap*-Flag –ΔBIR1 (delete 4-279nt, M3), *Xiap*-Flag -ΔBIR1+BIR2 (delete 4-690nt, M4), and *Xiap*-Flag -ΔBIR1+BIR2+BIR3 (delete 4-987nt, M5) were generated by per-forming overlap extension of PCR-mediated deletions on *Xiap*-Flag.

**Supplementary Figures**


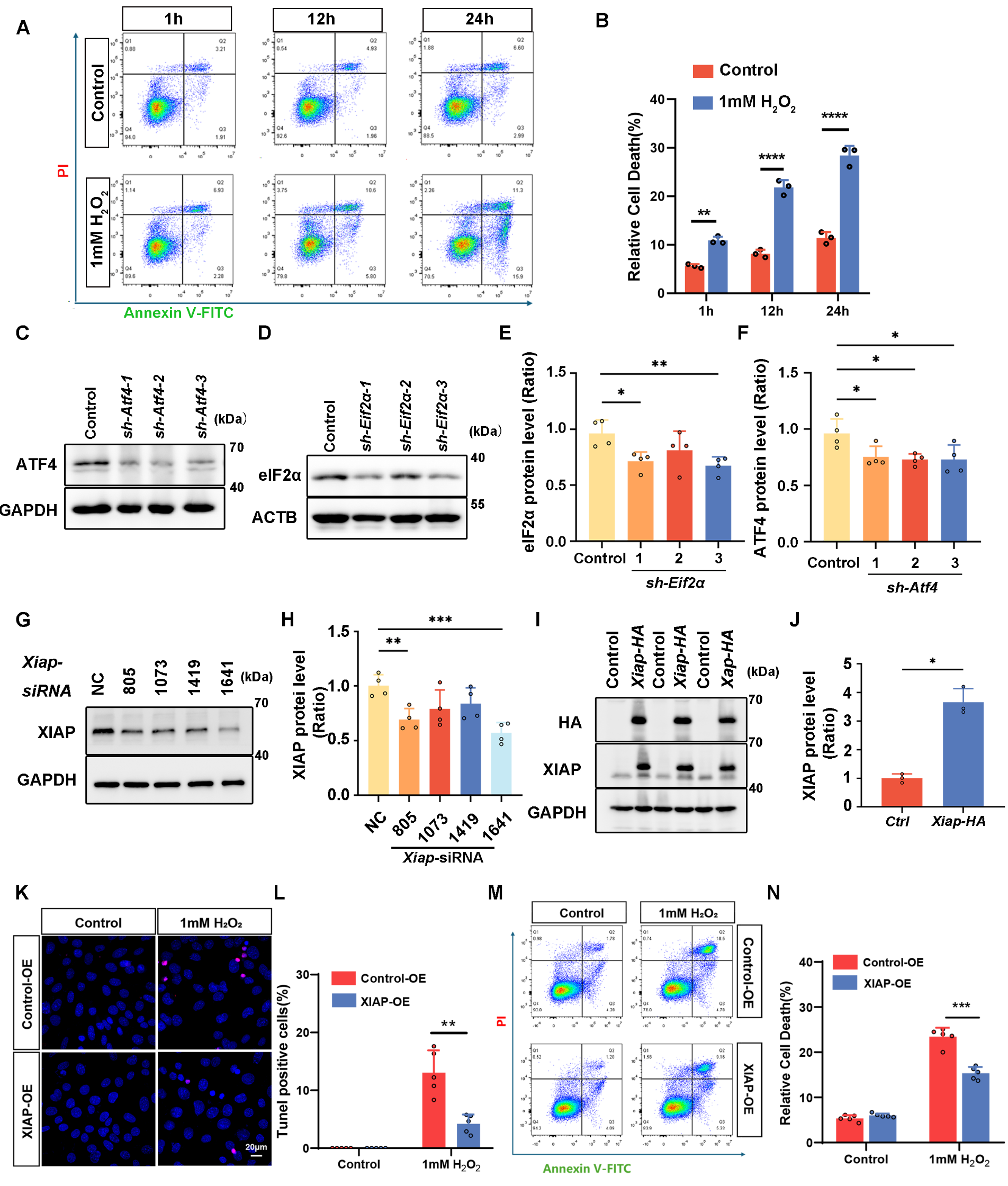


Figure S1. Transfection efficiency of RNA interference and overexpression constructs. A, B) Annexin V/PI flow cytometry dot plots and quantification of apoptotic cell populations after 1 h, 12 h and 24 h of H_2_O_2_ treatment. *n* = 3. C, D) Western blot showing the efficiency of different *Atf4*- and *Eif2α*-specific shRNA transfection experiments. HEI-OC1 cells were transfected with *Atf4*- and *Eif2α*-shRNA for 72 h. E, F) Quantification of western blots in C and D. *n* = 4. G) Western blot showing the efficiency of different *Xiap siRNA* transfection experiments. HEI-OC1 cells were transfected with *Xiap* siRNAs for 72 h. H) Quantification of western blot in G. *n* = 4. I) Western blot showing transfection efficiency of *Xiap-HA* overexpression in HEI-OC1 cells using XIAP and HA antibodies. HEI-OC1 cells were transfected with *Xiap-HA* plasmids for 72 h. J) Quantification of XIAP levels in I. *n* = 3. K, L) Immunofluorescence staining and quantitative analysis of HEI-OC1 cells following transfection with *Xiap-HA* or control plasmids for 72 h, followed by treatment with 1 mM H_2_O_2_ for 12 h, were performed using the TUNEL Apoptosis Detection Kit. *n* = 5. M, N) Annexin V/PI flow cytometry dot plots and quantification of apoptotic cell populations were analyzed after transfection with Xiap-HA or control plasmids for 72 hours, followed by 12 hours of H_2_O_2_ treatment. *n* = 5. Data are expressed as the mean ± SD. Statistical analyses were performed using the Student’s t-test or one-way ANOVA, as appropriate. Statistical significance is indicated as follow: **p* < 0.05, ***p* < 0.01, ****p* < 0.001.


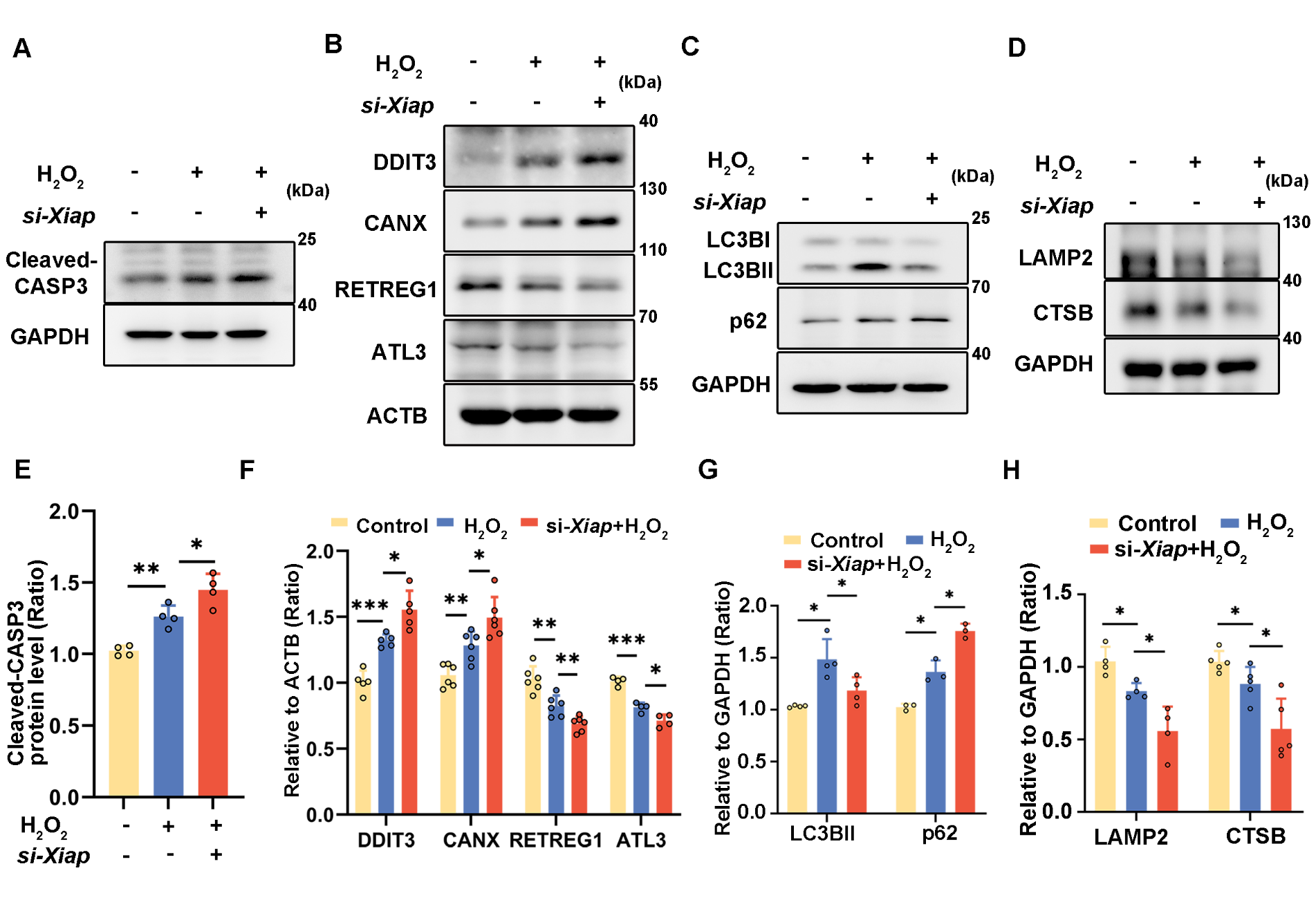


Figure S2. Knockdown of XIAP in H_2_O_2_-treated HEI-OC1 cells led to A) Changes in cleaved-CASP3 levels after 12 h of 1 mM H_2_O_2_ treatment in XIAP knockdown HEI-OC1 cells. B) Western blot showing the levels of DDIT3, CANX, RETREG1, and ATL3 under the same conditions as in A. C-D) Western blot showing the levels of LC3BII, p62, LAMP2, and CTSB after H_2_O_2_ treatment in HEI-OC1 cells knocked down for XIAP. E-H) Quantification of western blot bands in A-D. *n* ≥ 4. Data are expressed as the mean ± SD. Statistical analyses were performed using the Student’s t-test or one-way ANOVA, as appropriate. Statistical significance is indicated as follows: **p* < 0.05, ***p* < 0.01, ****p* < 0.01.


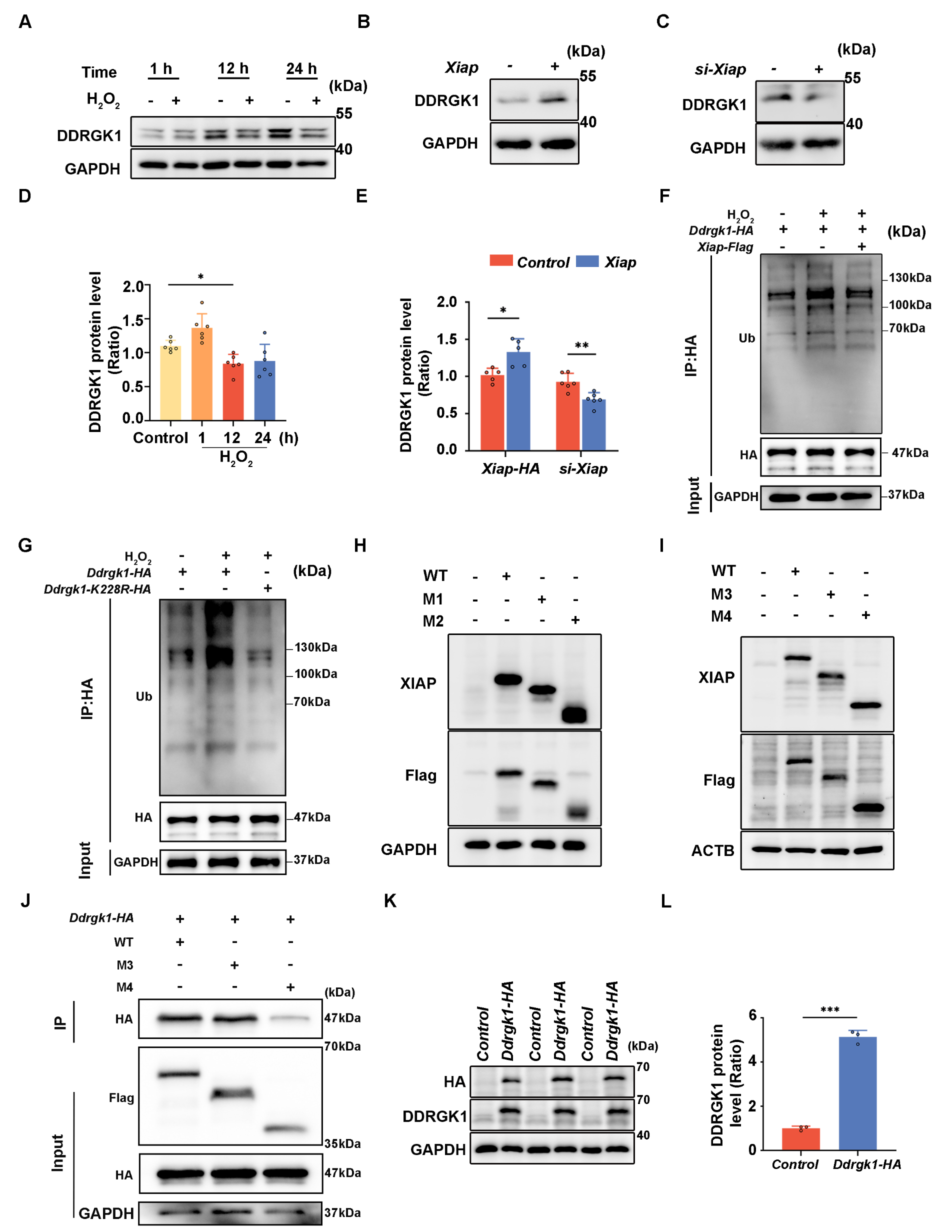


Figure S3. Levels of XIAP and DDRGK1 under different conditions. A) Western blot showing changes in the levels of DDRGK1 following treatment with 1 mM H_2_O_2_ at various time points. B) Western blot showing DDRGK1 levels in HEI-OC1 cells transfected with the *Xiap-HA* plasmid. C) Western blot showing DDRGK1 levels in HEI-OC1 cells transfected with *Xiap* siRNA. D) Quantification of western blot bands in A. *n* = 6. E) Quantification of western blots results in B and C. *n* ≥ 5. F) HEI-OC1 cells were transfected with *Ddrgk1-HA* and *Xiap-Flag* overexpression plasmids for 48 hours, followed by treatment with H_2_O_2_ for 12 hours, after which the ubiquitination level of DDRGK1 was assessed by immunoprecipitation using HA Tags. G) HEI-OC1 cells were transfected with *Ddrgk1-HA* or *Ddrgk1-K228R-HA* mutant overexpression plasmids for 48 hours, followed by treatment with H_2_O_2_ for 12 hours, after which the ubiquitination level of DDRGK1 was assessed by immunoprecipitation using HA Tags.

H, I) Western blot showing truncated XIAP expression in HEI-OC1 cells. J) HEI-OC1 cells were transfected with truncated XIAP and *Ddrgk1-HA* overexpression plasmids for 48 hours, after which the level of DDRGK1 was evaluated by co-immunoprecipitation using Flag Tags. K) Western blot showing the transfection efficiency of DDRGK1 expression using HA and DDRGK1 antibodies. HEI-OC1 cells were transfected with *Ddrgk1-HA* plasmids for 72 h. L) Quantification of DDRGK1 bands in J. *n* = 3. Data are expressed as the mean ± SD. Statistical analyses were performed using the Student’s t-test or one-way ANOVA, as appropriate. Statistical significance is indicated as follows: **p* < 0.05, ***p* < 0.01, ****p* < 0.01.


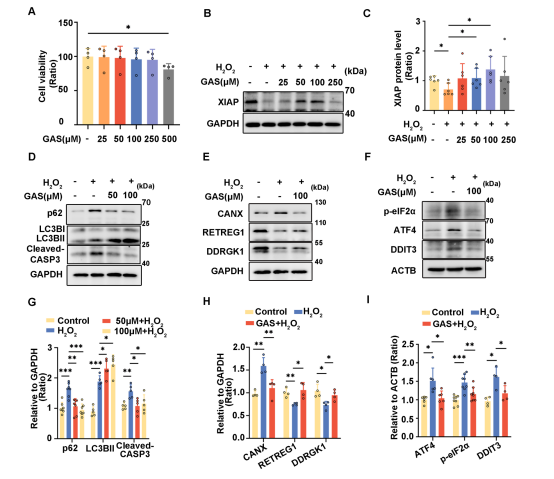


Figure S4. GAS treatment promotes the survival of HEI-OC1 cells by activating ER-phagy. A) Cell viability assay showing the effects of various GAS concentrations on HEI-OC1 cells treated for 36 h. *n* = 4. B) Western blot showing changes in the levels of XIAP after pretreatment with different concentrations of GAS for 24 h, followed by treatment with 1 mM H_2_O_2_ for 12 h. C) Quantification of western blots in B. *n* = 5. D) Western blot illustrating changes in the levels of p62, LC3B, and cleaved CASP3 in HEI-OC1 cells pretreated with GAS for 24 h, followed by co-treatment with 1 mM H_2_O_2_ and GAS (50 or 100 μM) for 12 h. E) Western blot showing changes in the levels of CANX, RETREG1, and DDRGK1 under 1 mM H_2_O_2_ and 100 μM GAS treatments. F) Western blot showing the expression levels of p-eIF2α, ATF4, and DDIT3 in HEI-OC1 cells treated with 1 mM H_2_O_2_ and 100 μM GAS for 12 h. G-I) Quantification of western blots in D-F. n ≥4. Data are expressed as the mean ± SD. Statistical analyses were performed using the Student’s t-test or one-way ANOVA, as appropriate. Statistical significance is indicated as follows: **p* < 0.05, ***p* < 0.01, ****p* < 0.001.

**Table S1. PCR primers, siRNA, and shRNA sequences used in this study**

| **Targets** | **Sequences (5’-3’)** |
| --- | --- |
| *Xiap-*Forward | CGAGCTGGGTTTCTTTATACCG |
| *Xiap-*Reverse | GCAATTTGGGGATATTCTCCTGT |
| *Gapdh-*Forward | AGG TCG GTG TGA ACG GAT TTG |
| *Gapdh-* Reverse | TGTAGACCATGTAGTTGAGGTCA |
| SiRNA-Negative Control-Forward | UUCUCCGAACGUGUCACGUTT |
| SiRNA-Negative Control- Reverse | ACGUGACACGUUCGGAGAATT |
| *Xiap*-siRNA 805 Forward | CUGGACAGGUUGUAGAUAUTT |
| *Xiap*-siRNA 805 Reverse | AUAUCUACAACCUGUCCAGTT |
| *Xiap*-siRNA 1419 Forward | GAGGAAUCUUUGGGAAGAATT |
| *Xiap*-siRNA 1419 Reverse | UUCUUCCCAAAGAUUCCUCTT |
| *Xiap*-siRNA 1641 Forward | GAGGAUGAGUCAAGUCAAATT |
| *Xiap*-siRNA 1641 Reverse | UUUGACUUGACUCAUCCUCTT |
| *Xiap*-siRNA 1073 Forward | UCGAACAUUAACGUUCCGGTT |
| *Xiap*-siRNA 1073 Reverse | UCGAACAUUAACGUUCCGGTT |
| *Atf4*-shRNA1 Forward | CCAGAGCATTCCTTTAGTTTA |
| *Atf4***-**shRNA1 Reverse | TAAACTAAAGGAATGCTCTGG |
| *Atf4*-shRNA2 Forward | CGGACAAAGATACCTTCGAGT |
| *Atf4***-**shRNA2 Reverse | ACTCGAAGGTATCTTTGTCCG |
| *Atf4*-shRNA3 Forward | CTAGGTCTCTTAGATGACTAT |
| *Atf4***-**shRNA3 Reverse | ATAGTCATCTAAGAGACCTAG |
| *Eif2α*-shRNA1 Forward | GCCTCTTTATTCAAATGGAAT |
| *Eif2α***-**shRNA1 Reverse | ATTCCATTTGAATAAAGAGGC |
| *Eif2α*-shRNA2 Forward | GCCACTTTGAACTTCGGTATA |
| *Eif2α***-**shRNA2 Reverse | TATACCGAAGTTCAAAGTGGC |
| *Eif2α*-shRNA3 Forward | CCTCCACATGACAGAAGGTTT |
| *Eif2α***-**shRNA3 Reverse | AAACCTTCTGTCATGTGGAGG |
