# Supplementary figures and images for: XIAP Stabilizes DDRGK1 to Promote ER‐Phagy and Protects Against Noise‐Induced Hearing Loss (part 2 of 2)
Source: Adv Sci (Weinh). 2026 Jan 26;13(18):e11217. doi: 10.1002/advs.202511217 (PMC13042907; doi:10.1002/advs.202511217)

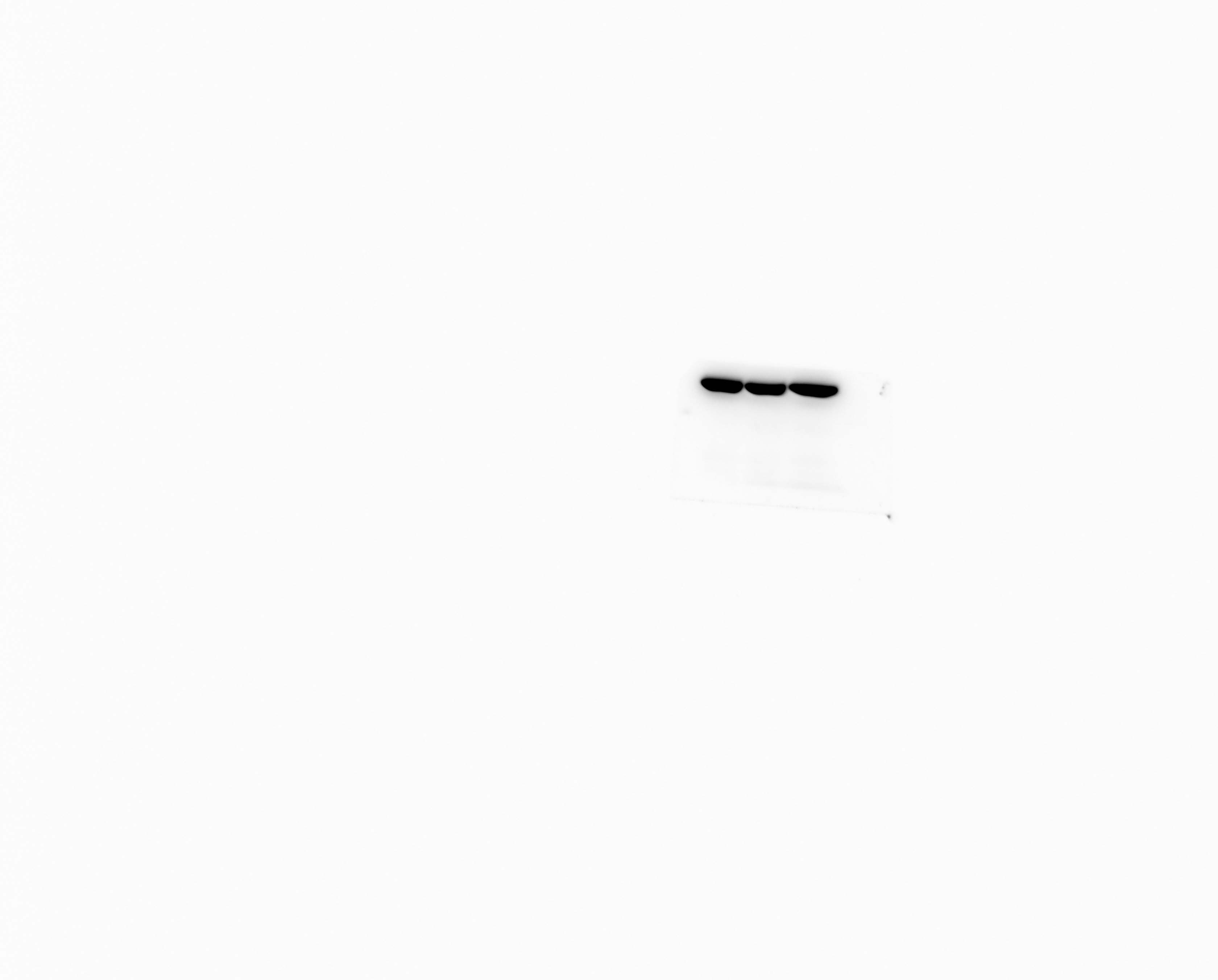

Supplement: Supplementary file 2 — Supporting File 2: advs73976‐sup‐0002‐SuppMat.zip. [file ADVS-13-e11217-s002.zip › WB#U4ee3#U8868#U56fe/xiap#U539f#U59cb#U6570#U636ewb1-JPEG/ACTIN_8 canx oex.jpg]

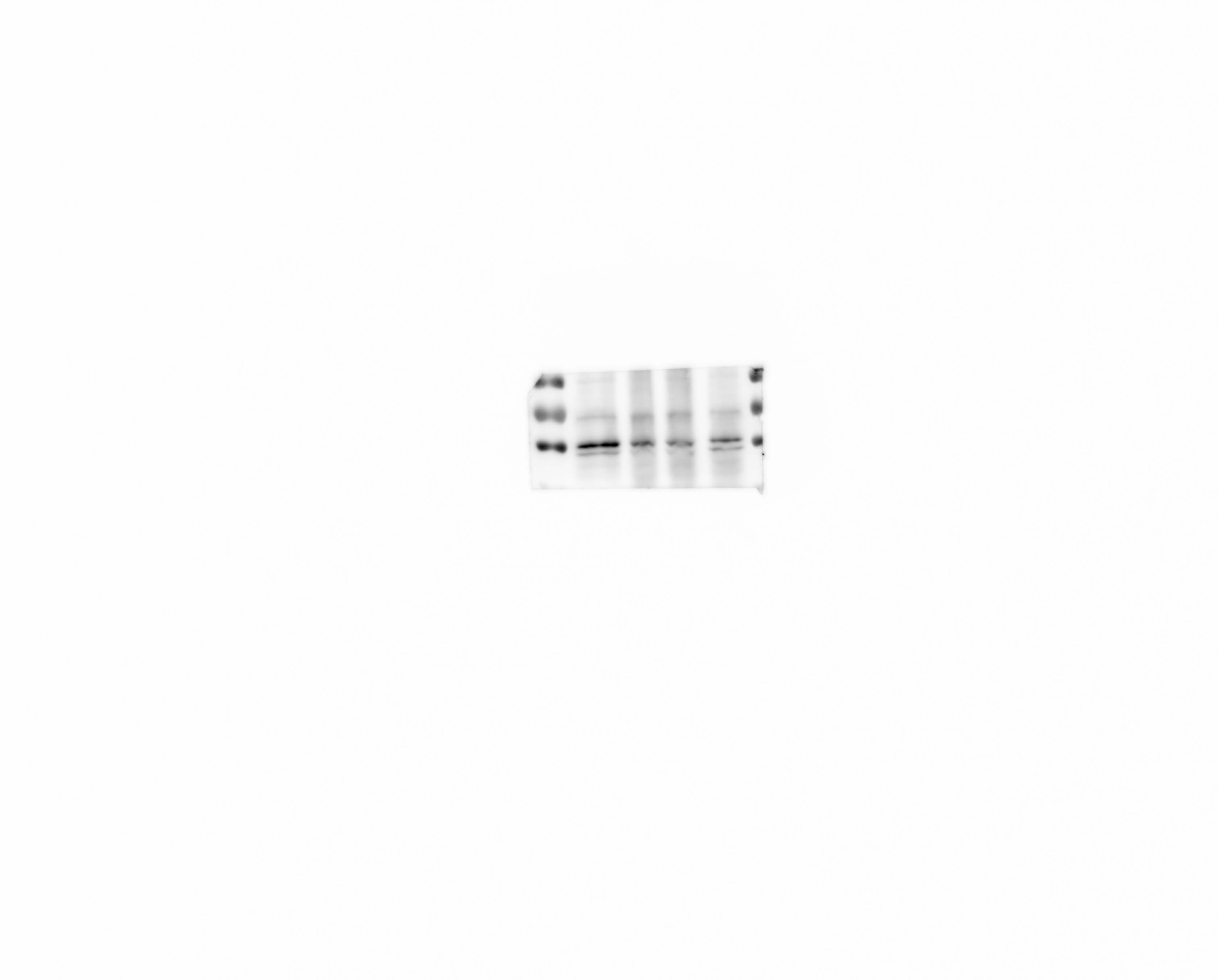

Supplement: Supplementary file 2 — Supporting File 2: advs73976‐sup‐0002‐SuppMat.zip. [file ADVS-13-e11217-s002.zip › WB#U4ee3#U8868#U56fe/xiap#U539f#U59cb#U6570#U636ewb1-JPEG/ATF4_5 shatf4#U6548#U7387.jpg]

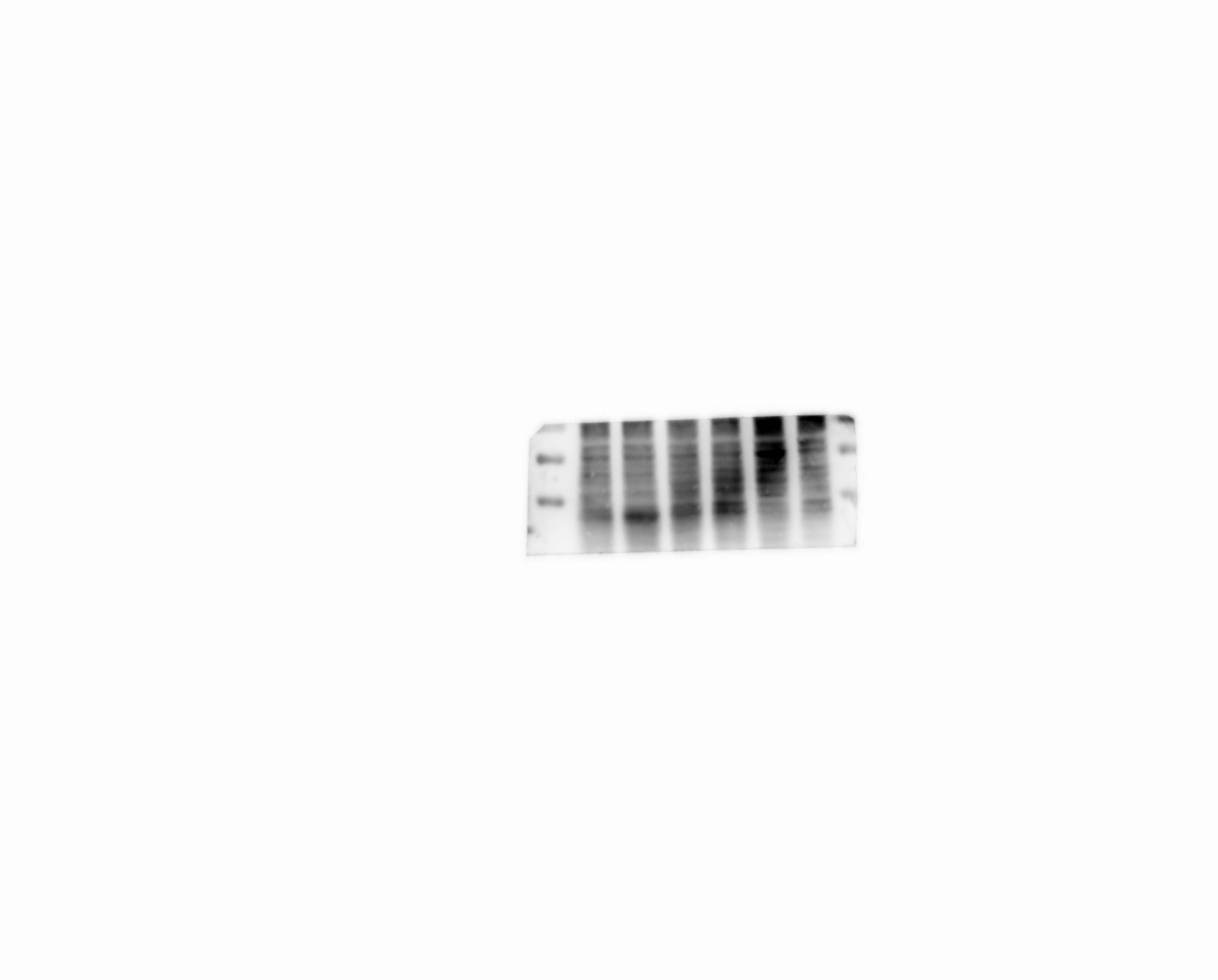

Supplement: Supplementary file 2 — Supporting File 2: advs73976‐sup‐0002‐SuppMat.zip. [file ADVS-13-e11217-s002.zip › WB#U4ee3#U8868#U56fe/xiap#U539f#U59cb#U6570#U636ewb1-JPEG/ATF4_8 DB.jpg]

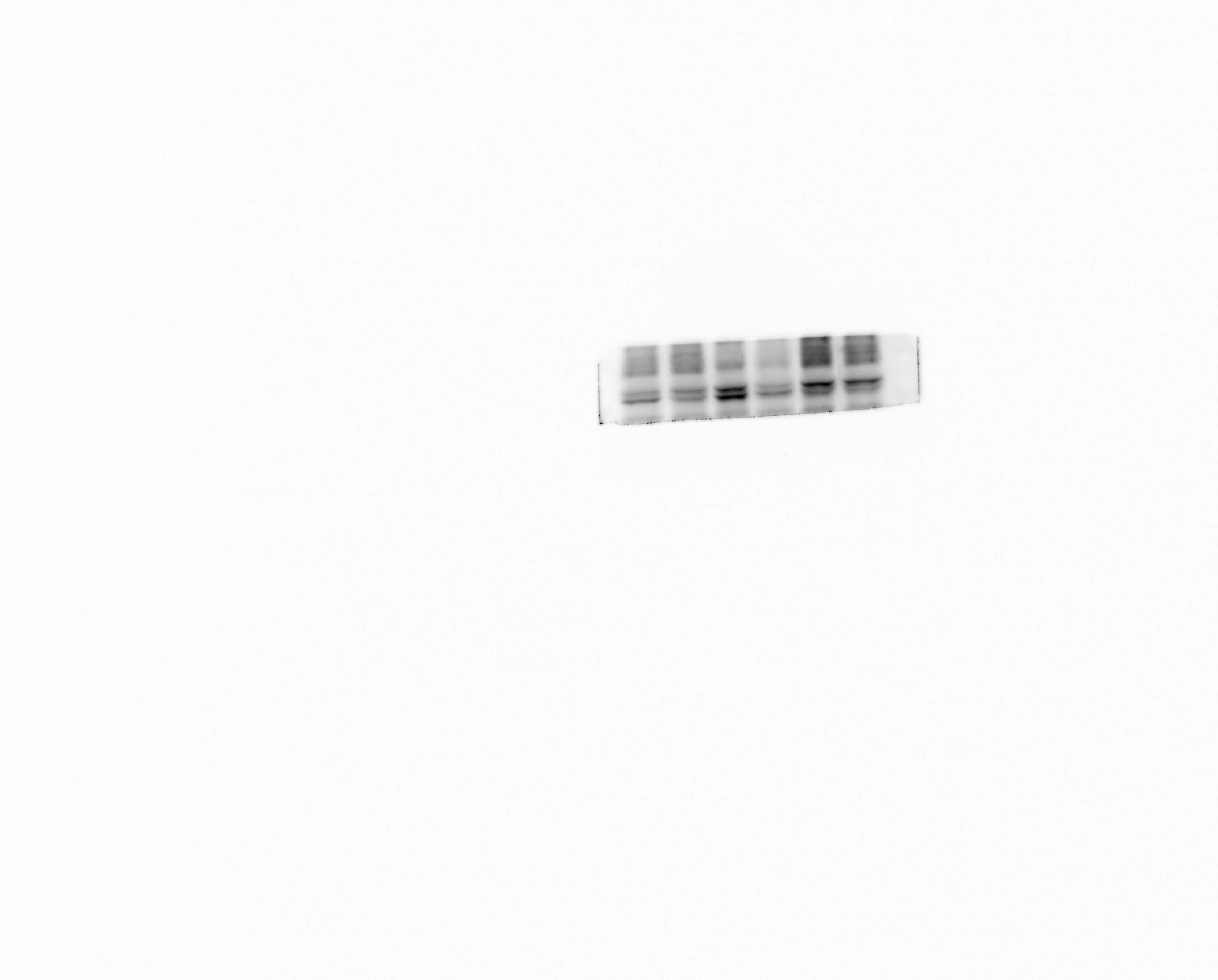

Supplement: Supplementary file 2 — Supporting File 2: advs73976‐sup‐0002‐SuppMat.zip. [file ADVS-13-e11217-s002.zip › WB#U4ee3#U8868#U56fe/xiap#U539f#U59cb#U6570#U636ewb1-JPEG/ATL3-_6.jpg]

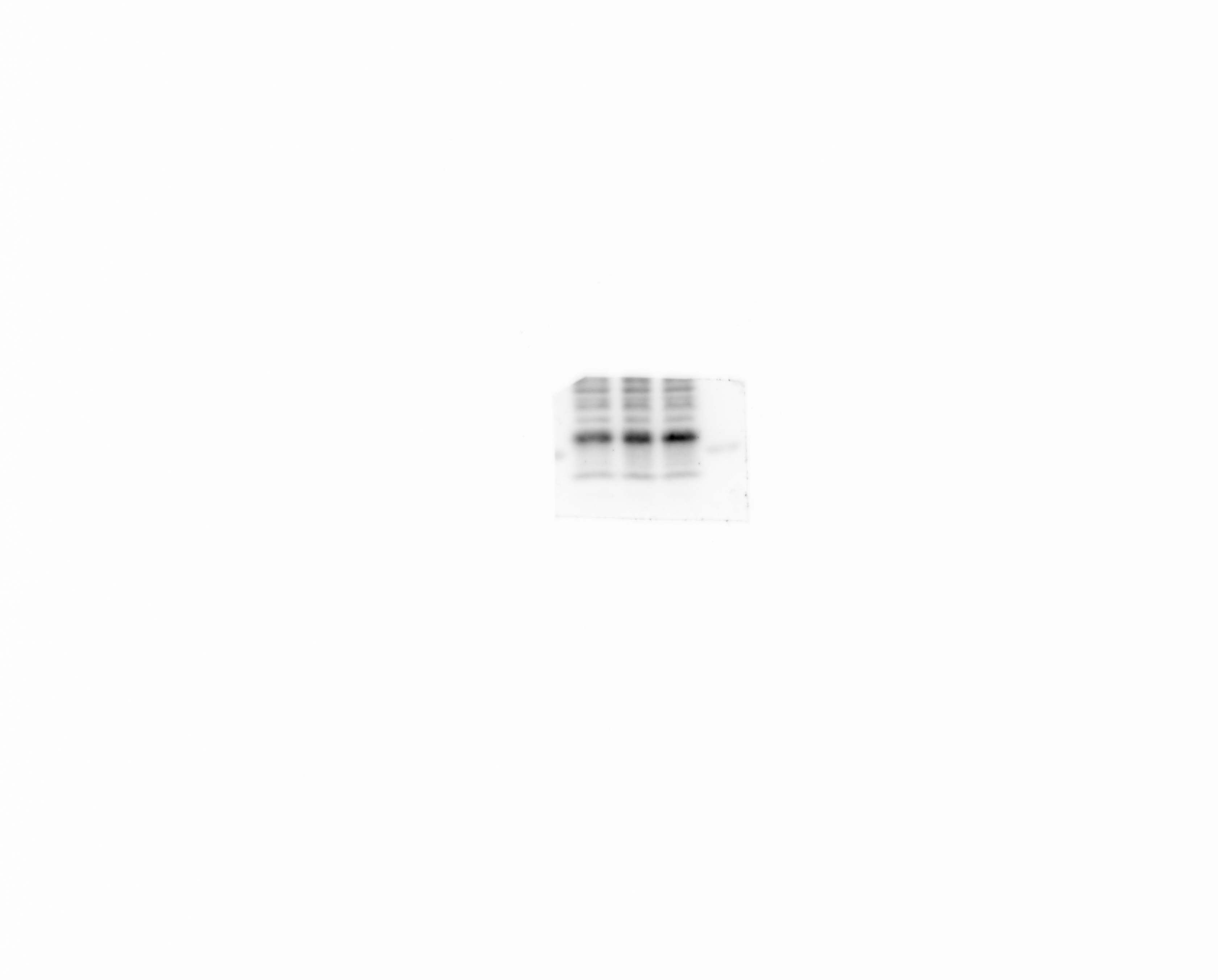

Supplement: Supplementary file 2 — Supporting File 2: advs73976‐sup‐0002‐SuppMat.zip. [file ADVS-13-e11217-s002.zip › WB#U4ee3#U8868#U56fe/xiap#U539f#U59cb#U6570#U636ewb1-JPEG/c-casps3 4s_8 six.jpg]

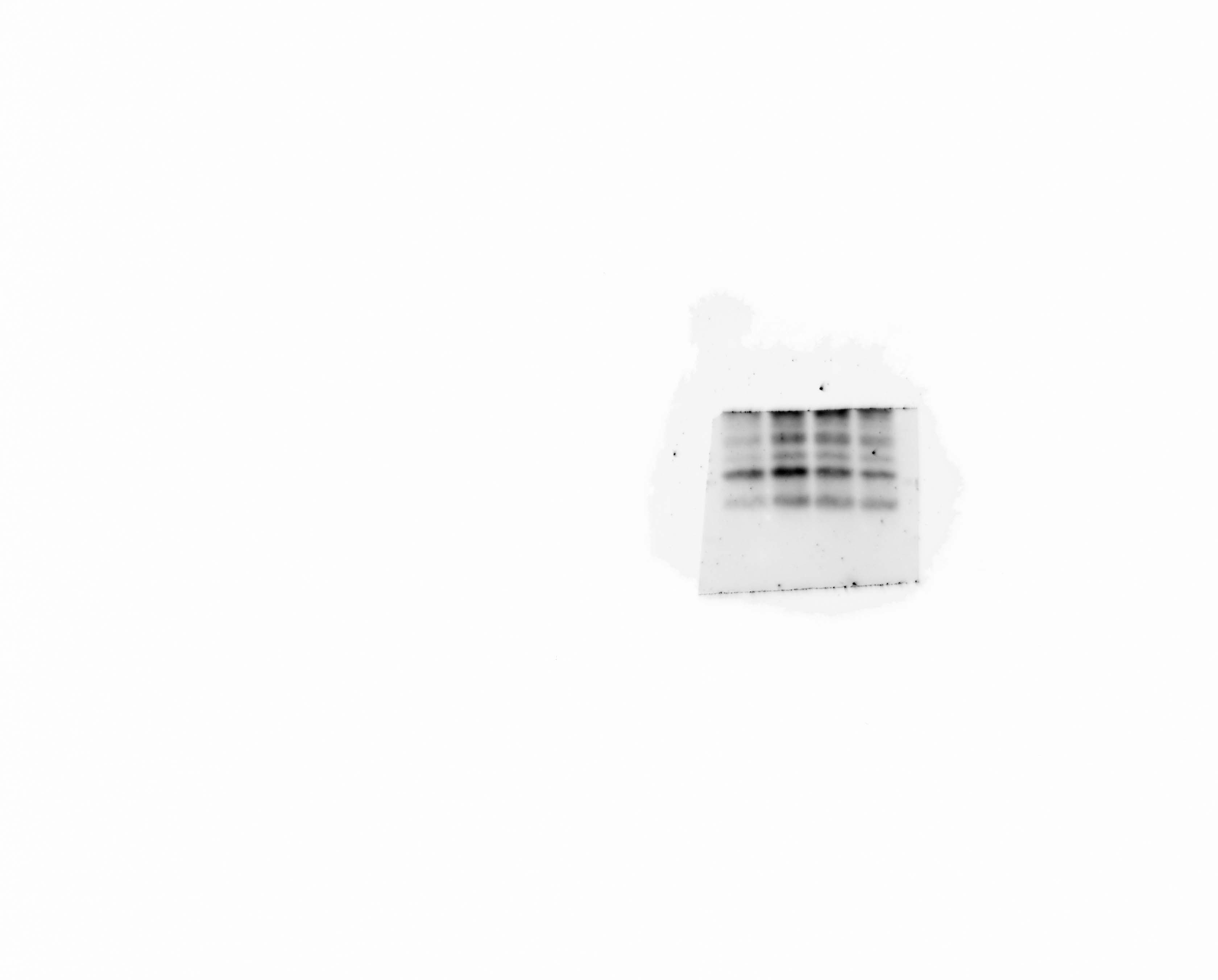

Supplement: Supplementary file 2 — Supporting File 2: advs73976‐sup‐0002‐SuppMat.zip. [file ADVS-13-e11217-s002.zip › WB#U4ee3#U8868#U56fe/xiap#U539f#U59cb#U6570#U636ewb1-JPEG/c-casps3-_3 #U4ee3#U8868 gas.jpg]

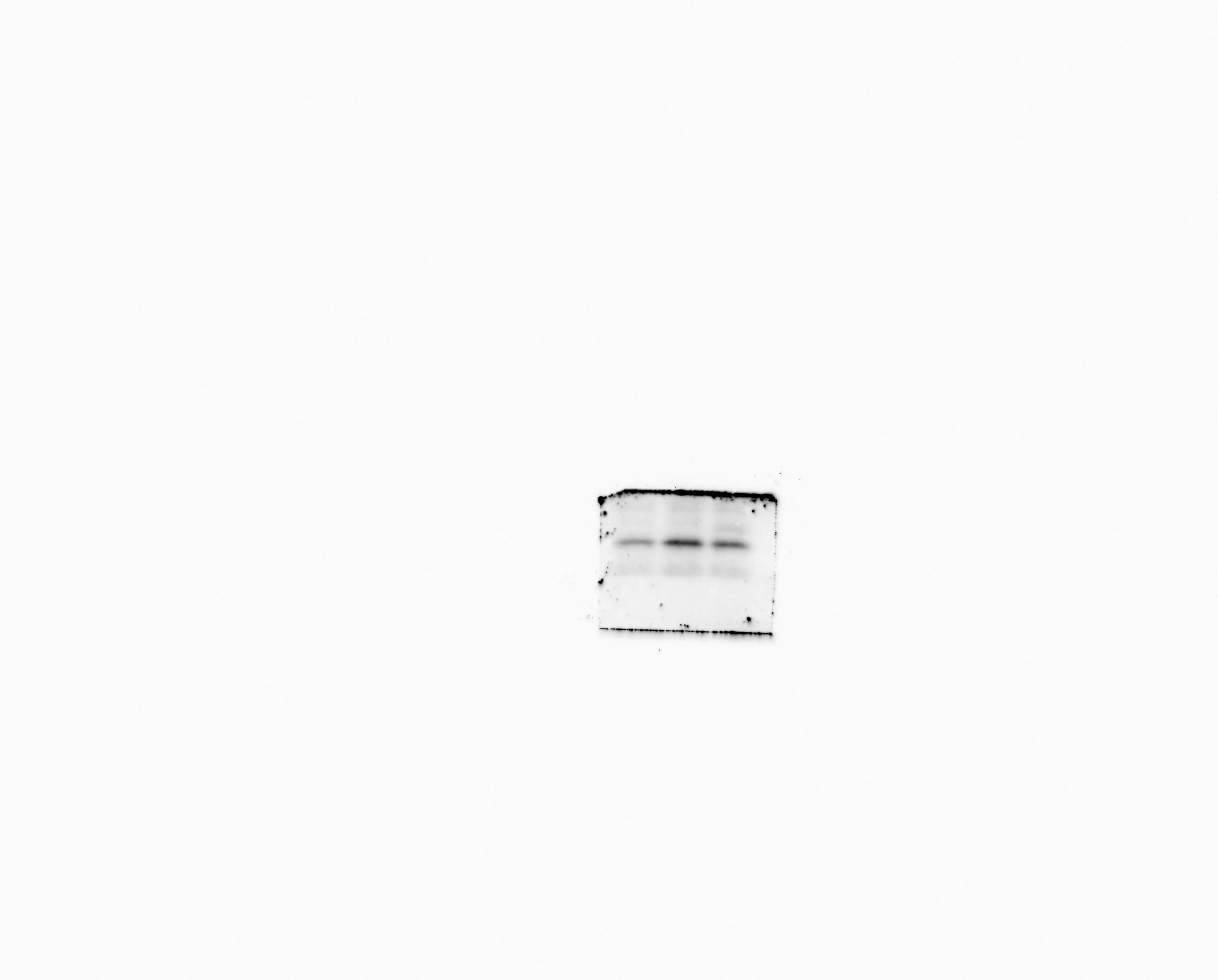

Supplement: Supplementary file 2 — Supporting File 2: advs73976‐sup‐0002‐SuppMat.zip. [file ADVS-13-e11217-s002.zip › WB#U4ee3#U8868#U56fe/xiap#U539f#U59cb#U6570#U636ewb1-JPEG/c-casps3_9 oex.jpg]

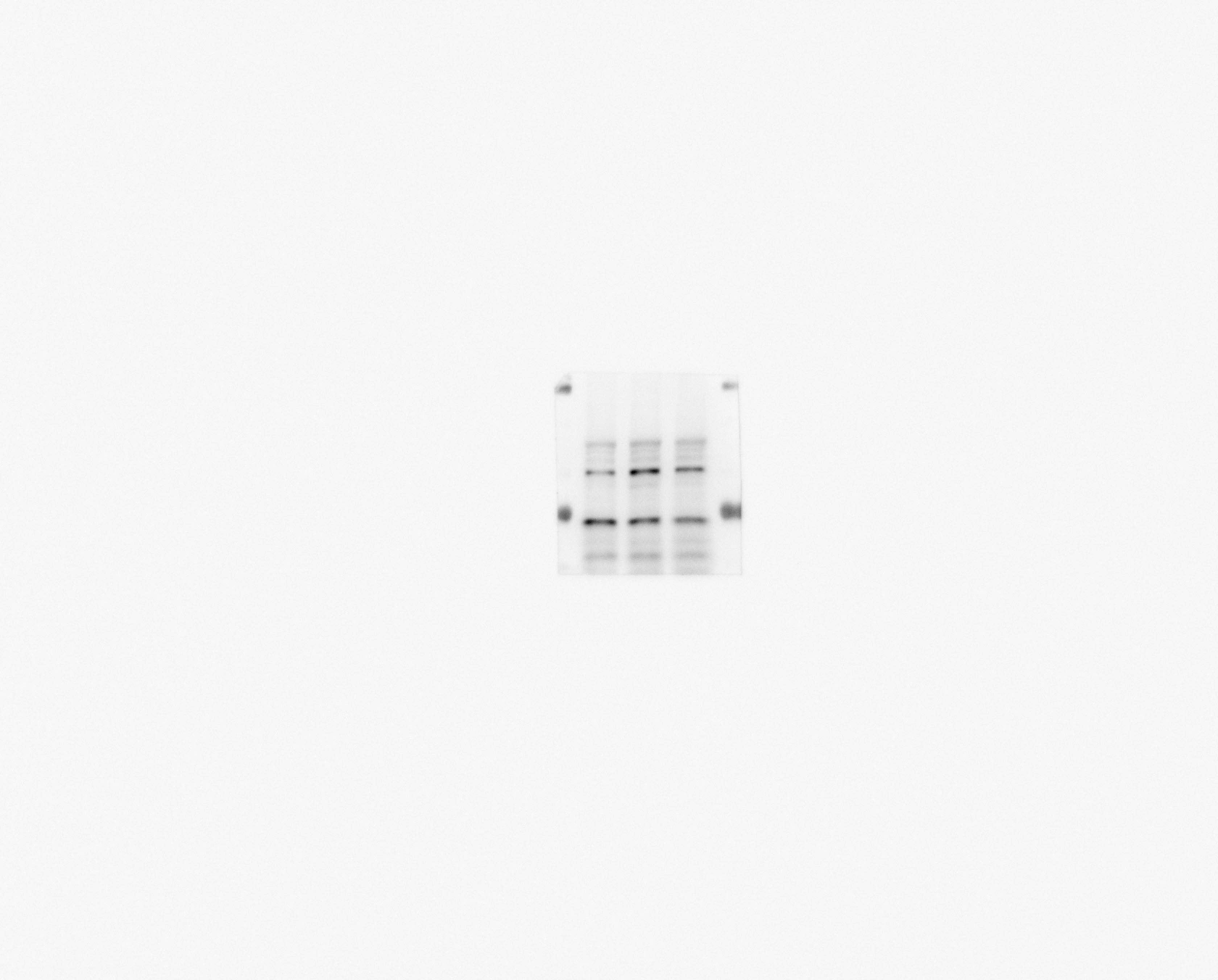

Supplement: Supplementary file 2 — Supporting File 2: advs73976‐sup‐0002‐SuppMat.zip. [file ADVS-13-e11217-s002.zip › WB#U4ee3#U8868#U56fe/xiap#U539f#U59cb#U6570#U636ewb1-JPEG/CANX_1 oex.jpg]

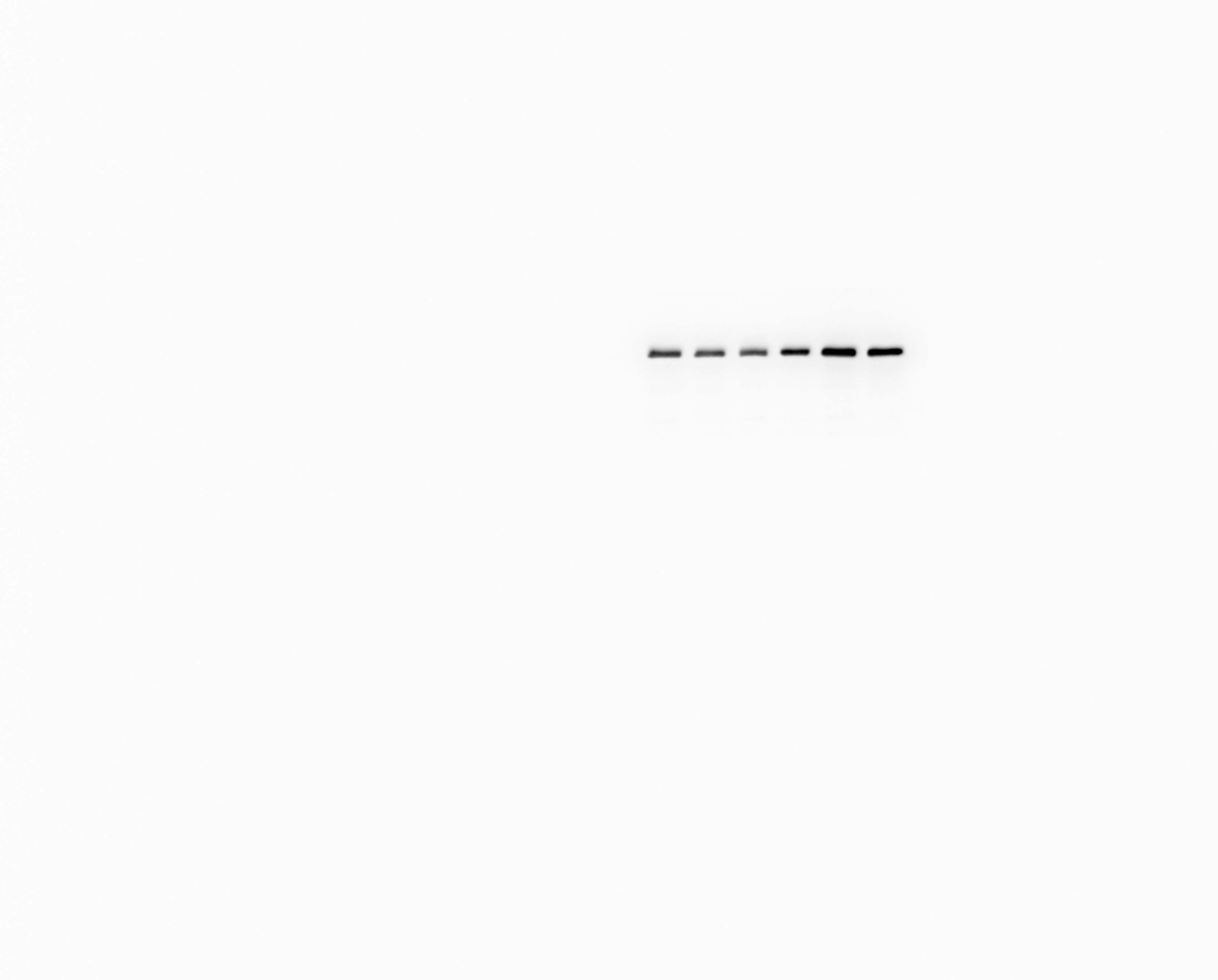

Supplement: Supplementary file 2 — Supporting File 2: advs73976‐sup‐0002‐SuppMat.zip. [file ADVS-13-e11217-s002.zip › WB#U4ee3#U8868#U56fe/xiap#U539f#U59cb#U6570#U636ewb1-JPEG/CANX_8.jpg]

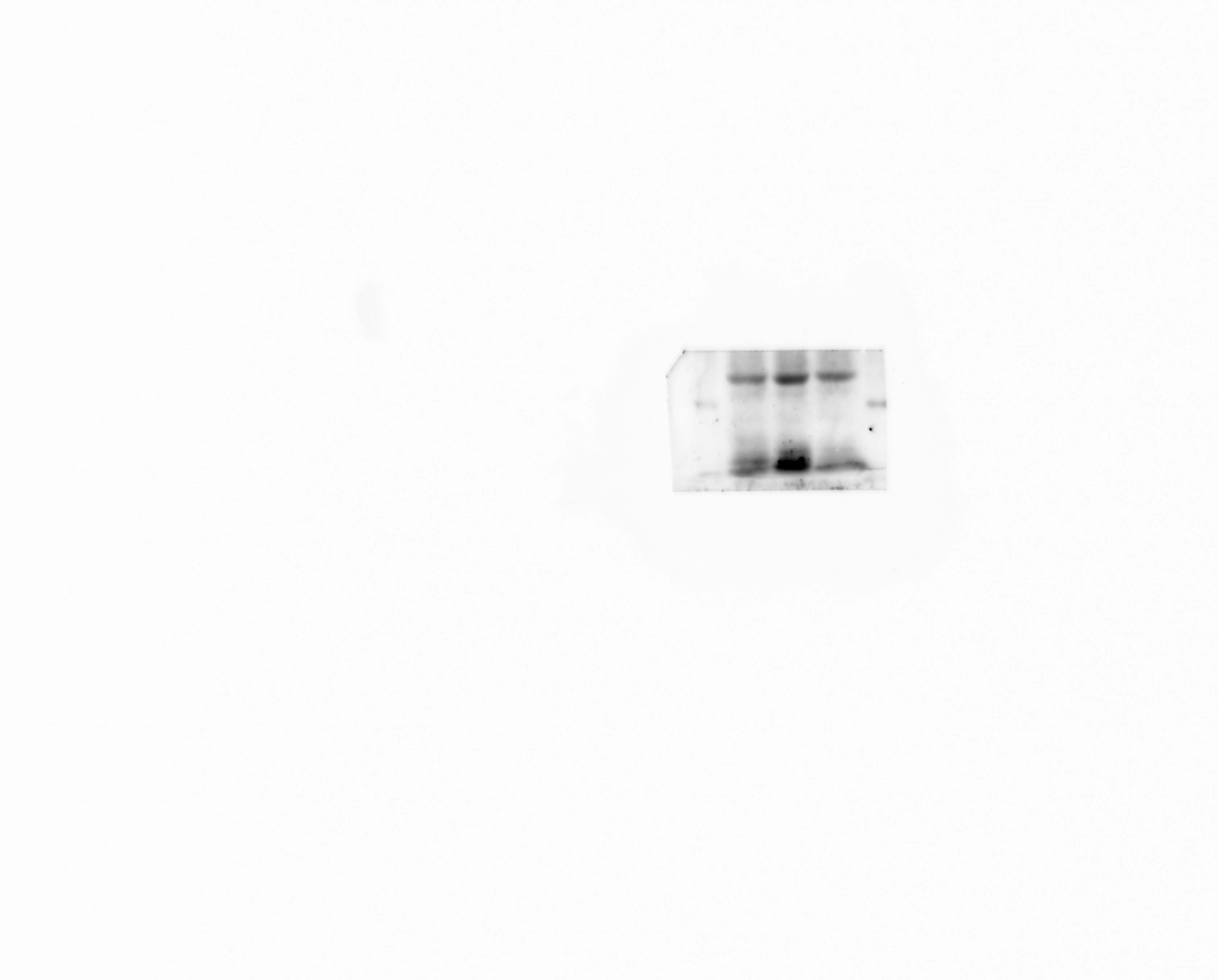

Supplement: Supplementary file 2 — Supporting File 2: advs73976‐sup‐0002‐SuppMat.zip. [file ADVS-13-e11217-s002.zip › WB#U4ee3#U8868#U56fe/xiap#U539f#U59cb#U6570#U636ewb1-JPEG/CHOP_6 oex.jpg]

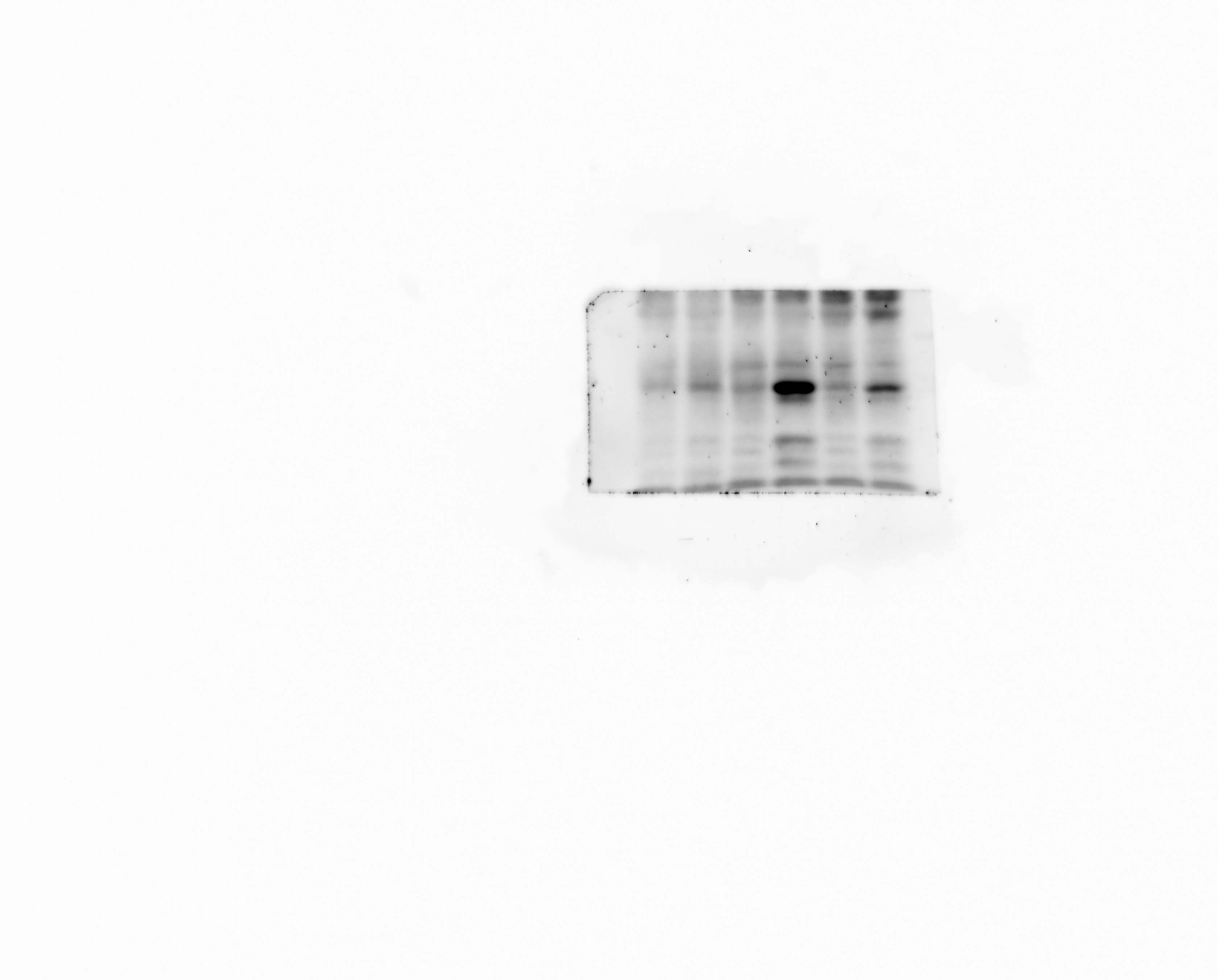

Supplement: Supplementary file 2 — Supporting File 2: advs73976‐sup‐0002‐SuppMat.zip. [file ADVS-13-e11217-s002.zip › WB#U4ee3#U8868#U56fe/xiap#U539f#U59cb#U6570#U636ewb1-JPEG/chop_6.jpg]

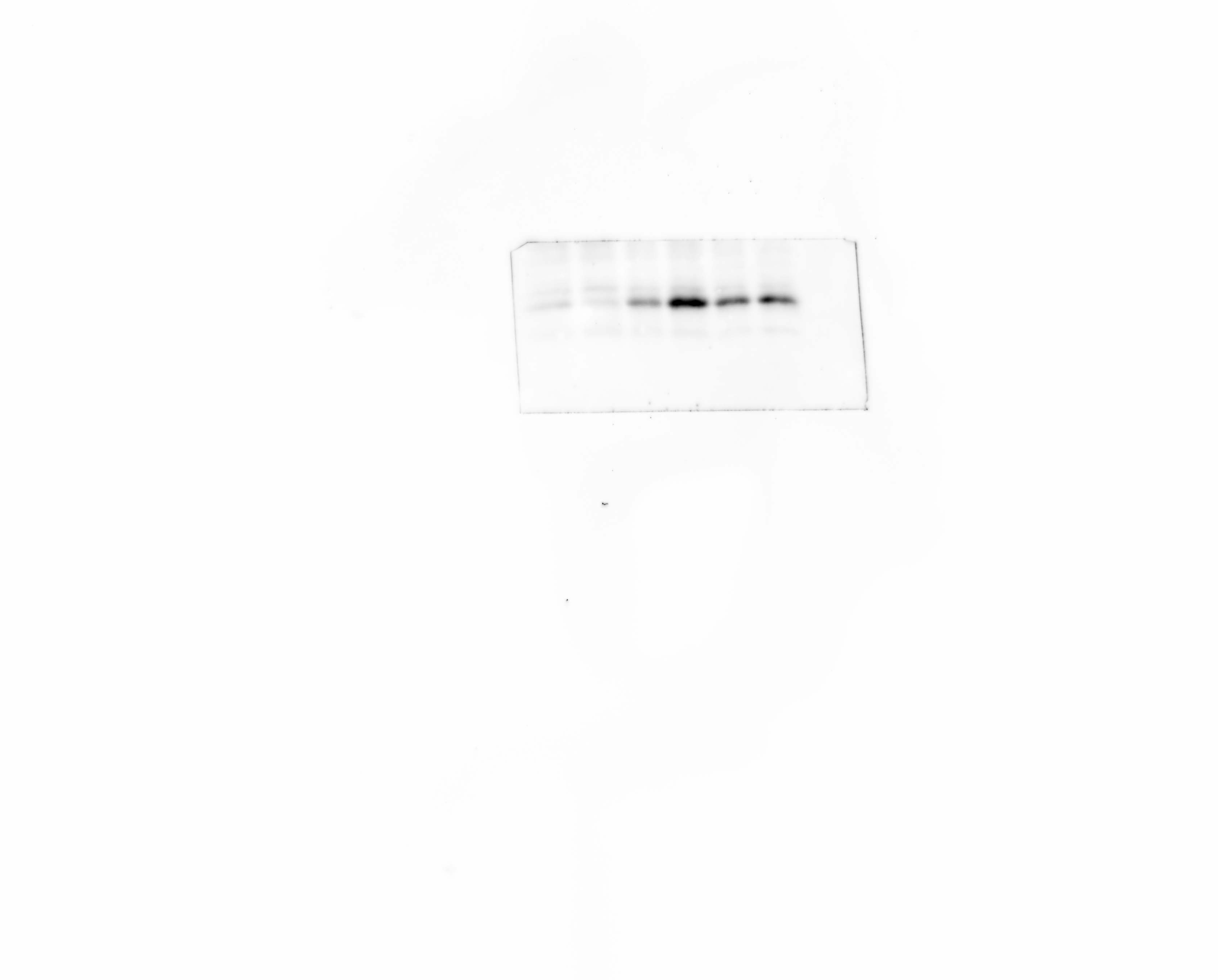

Supplement: Supplementary file 2 — Supporting File 2: advs73976‐sup‐0002‐SuppMat.zip. [file ADVS-13-e11217-s002.zip › WB#U4ee3#U8868#U56fe/xiap#U539f#U59cb#U6570#U636ewb1-JPEG/cleaved-casp3_5#U4ee3#U8868.jpg]

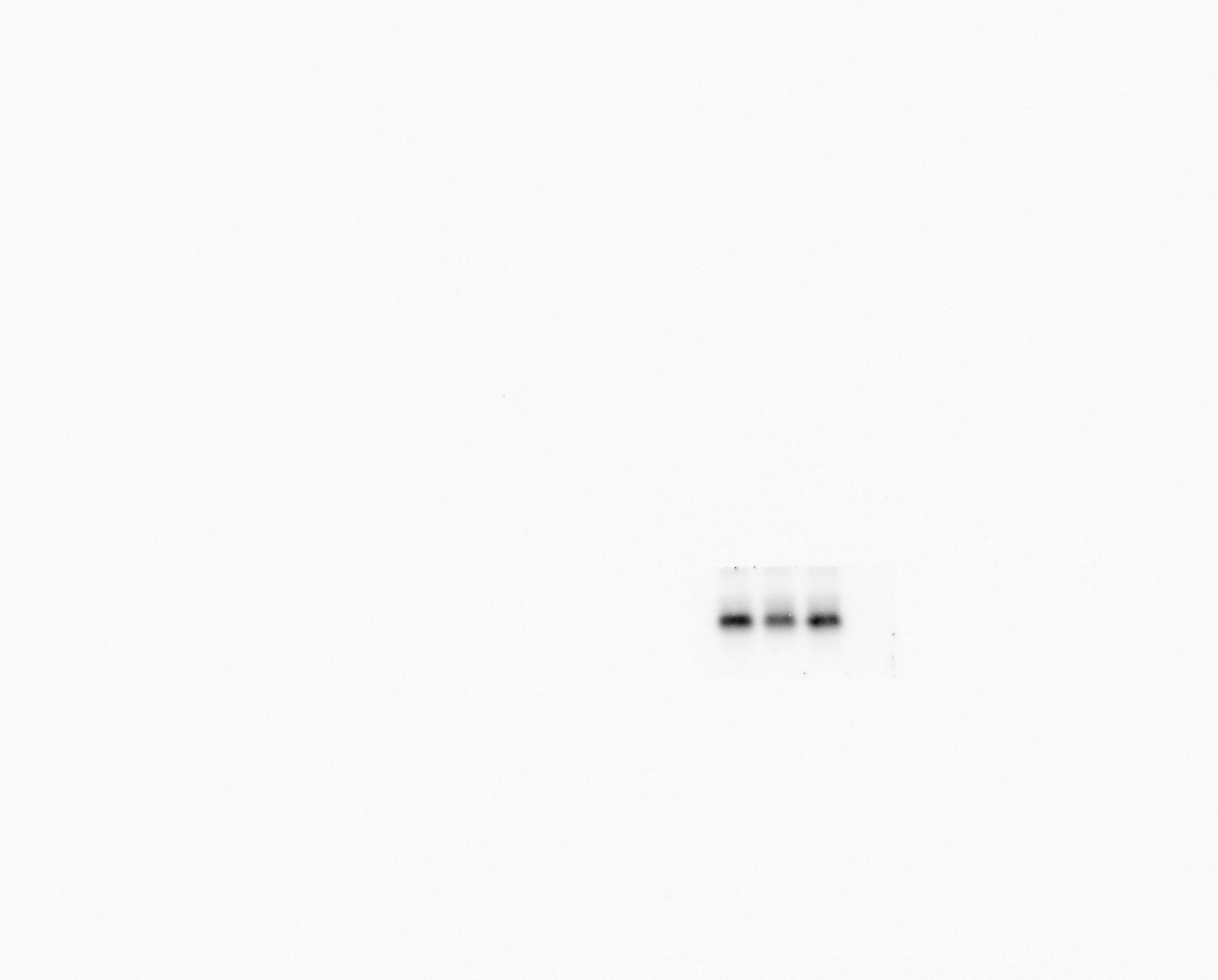

Supplement: Supplementary file 2 — Supporting File 2: advs73976‐sup‐0002‐SuppMat.zip. [file ADVS-13-e11217-s002.zip › WB#U4ee3#U8868#U56fe/xiap#U539f#U59cb#U6570#U636ewb1-JPEG/ctsb_1 oex.jpg]

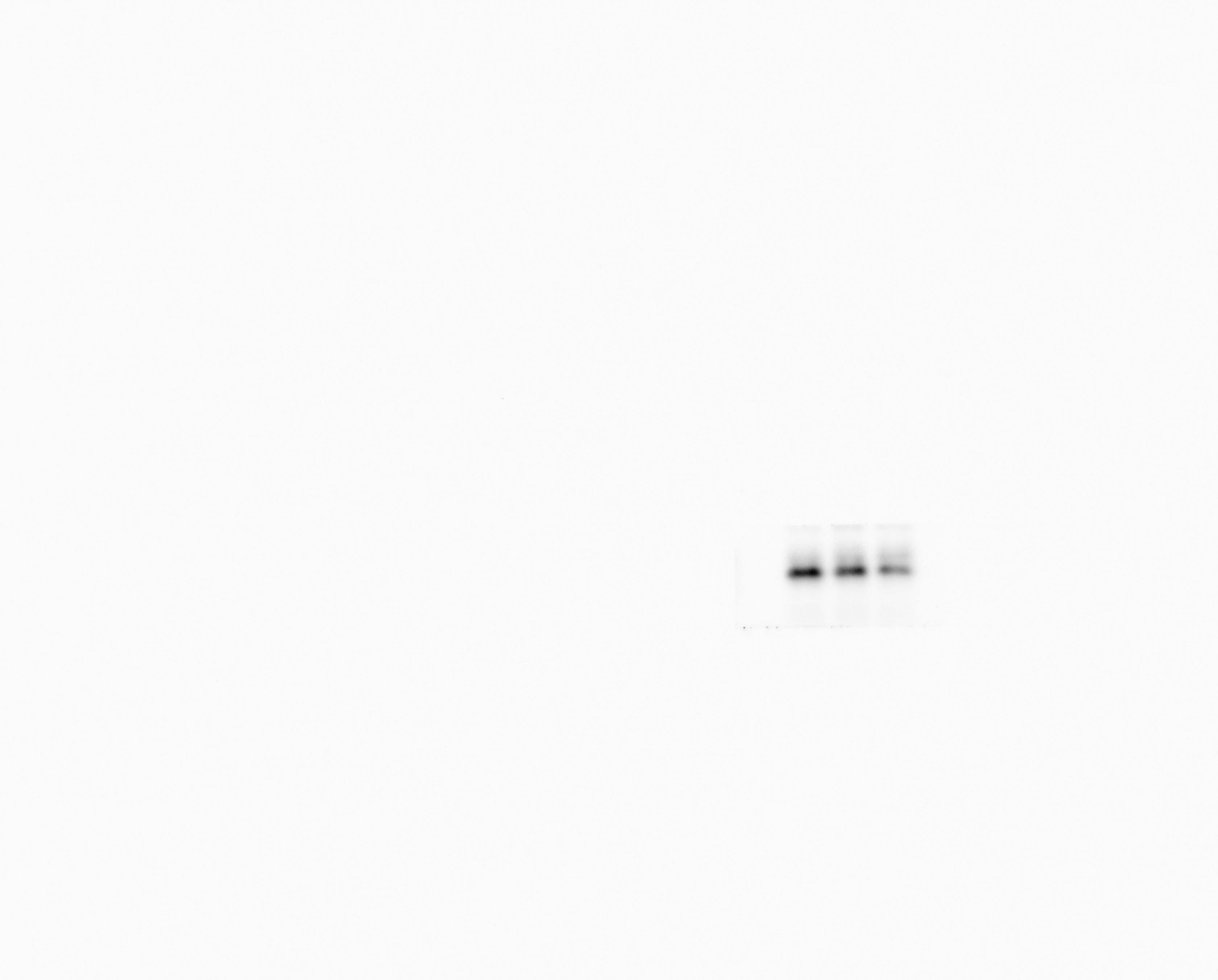

Supplement: Supplementary file 2 — Supporting File 2: advs73976‐sup‐0002‐SuppMat.zip. [file ADVS-13-e11217-s002.zip › WB#U4ee3#U8868#U56fe/xiap#U539f#U59cb#U6570#U636ewb1-JPEG/ctsb_1 six.jpg]

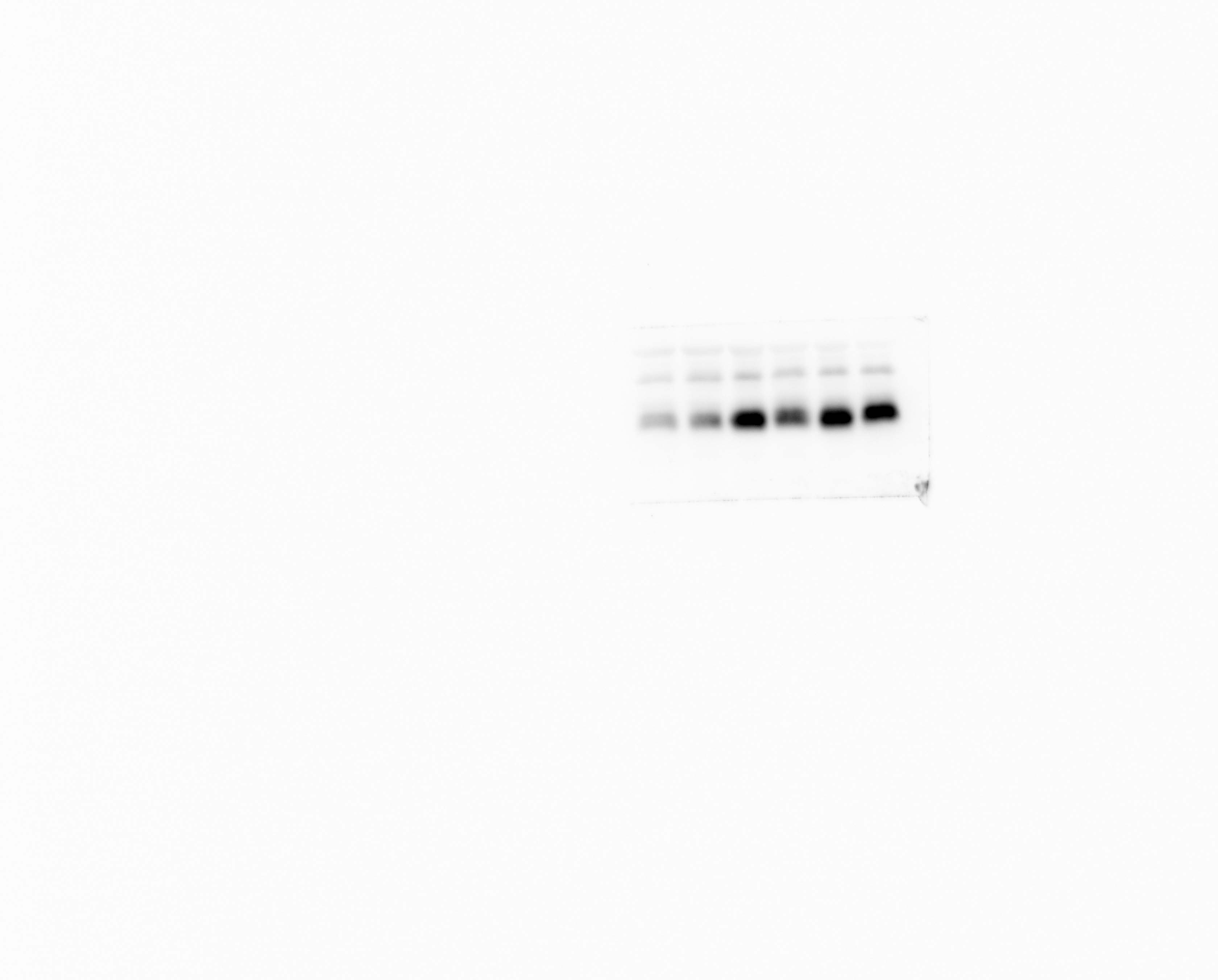

Supplement: Supplementary file 2 — Supporting File 2: advs73976‐sup‐0002‐SuppMat.zip. [file ADVS-13-e11217-s002.zip › WB#U4ee3#U8868#U56fe/xiap#U539f#U59cb#U6570#U636ewb1-JPEG/CTSB_7.jpg]

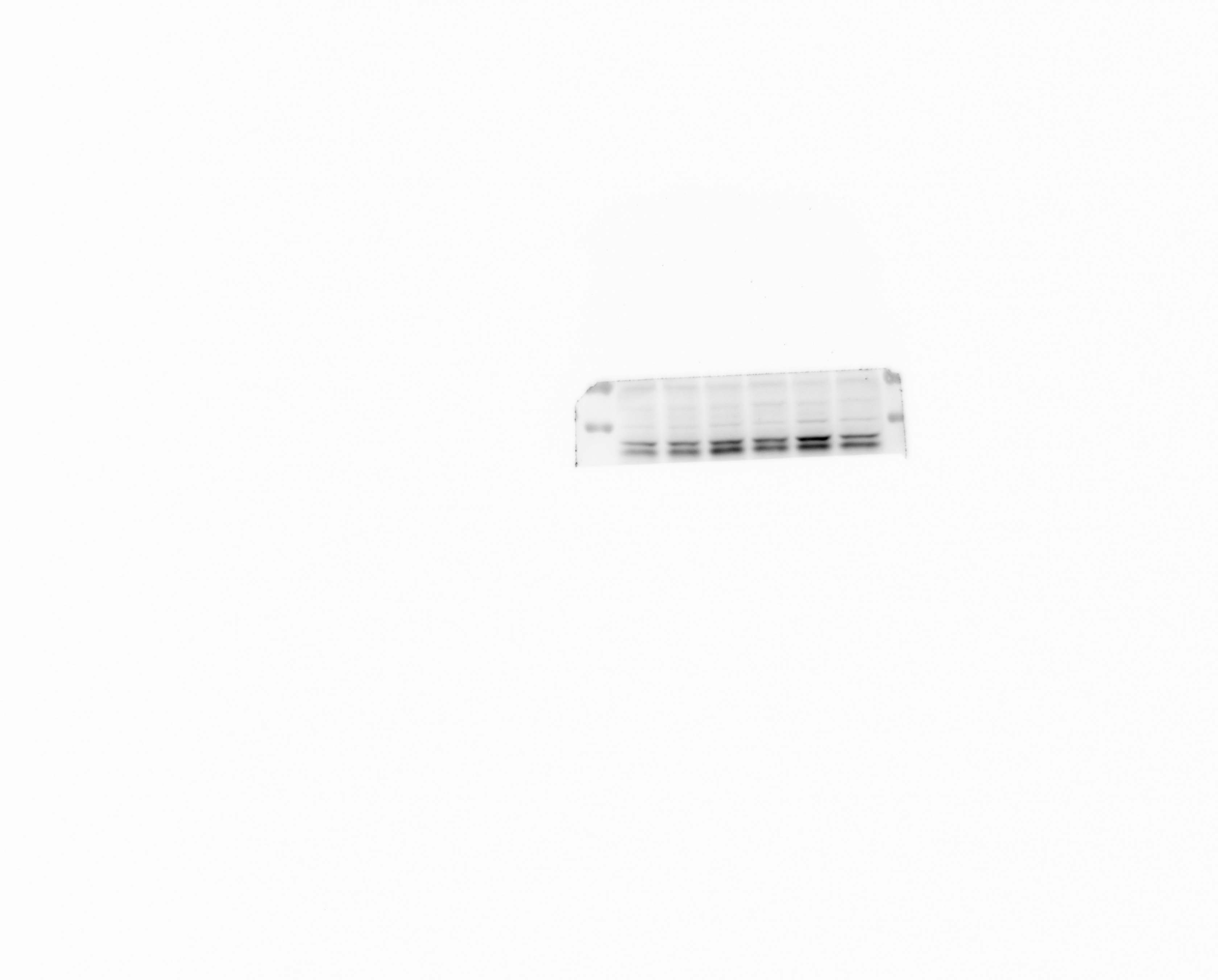

Supplement: Supplementary file 2 — Supporting File 2: advs73976‐sup‐0002‐SuppMat.zip. [file ADVS-13-e11217-s002.zip › WB#U4ee3#U8868#U56fe/xiap#U539f#U59cb#U6570#U636ewb1-JPEG/ddrgk1cut_6 #U4ee3#U8868.jpg]

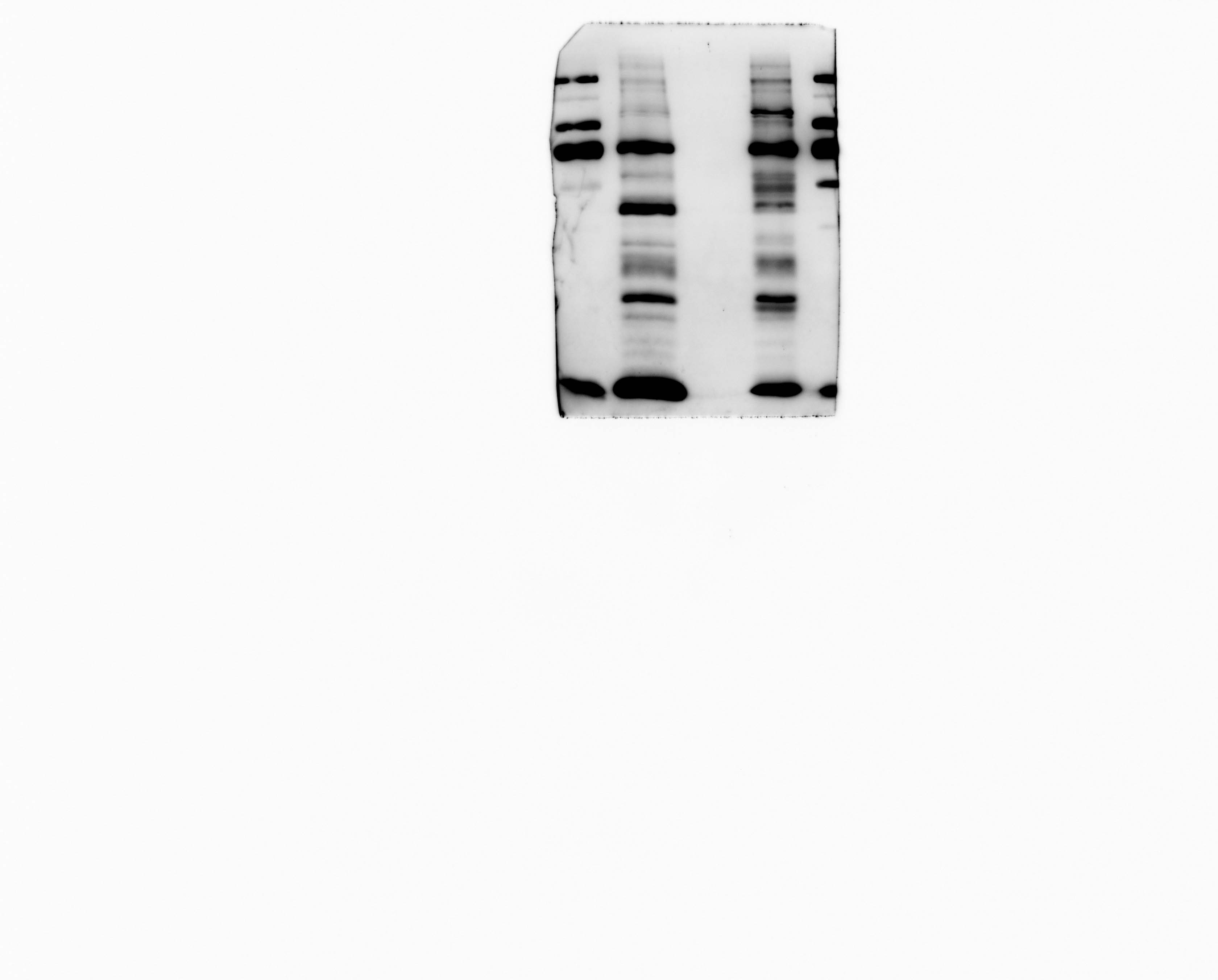

Supplement: Supplementary file 2 — Supporting File 2: advs73976‐sup‐0002‐SuppMat.zip. [file ADVS-13-e11217-s002.zip › WB#U4ee3#U8868#U56fe/xiap#U539f#U59cb#U6570#U636ewb1-JPEG/ddrgk1_10 ip.jpg]

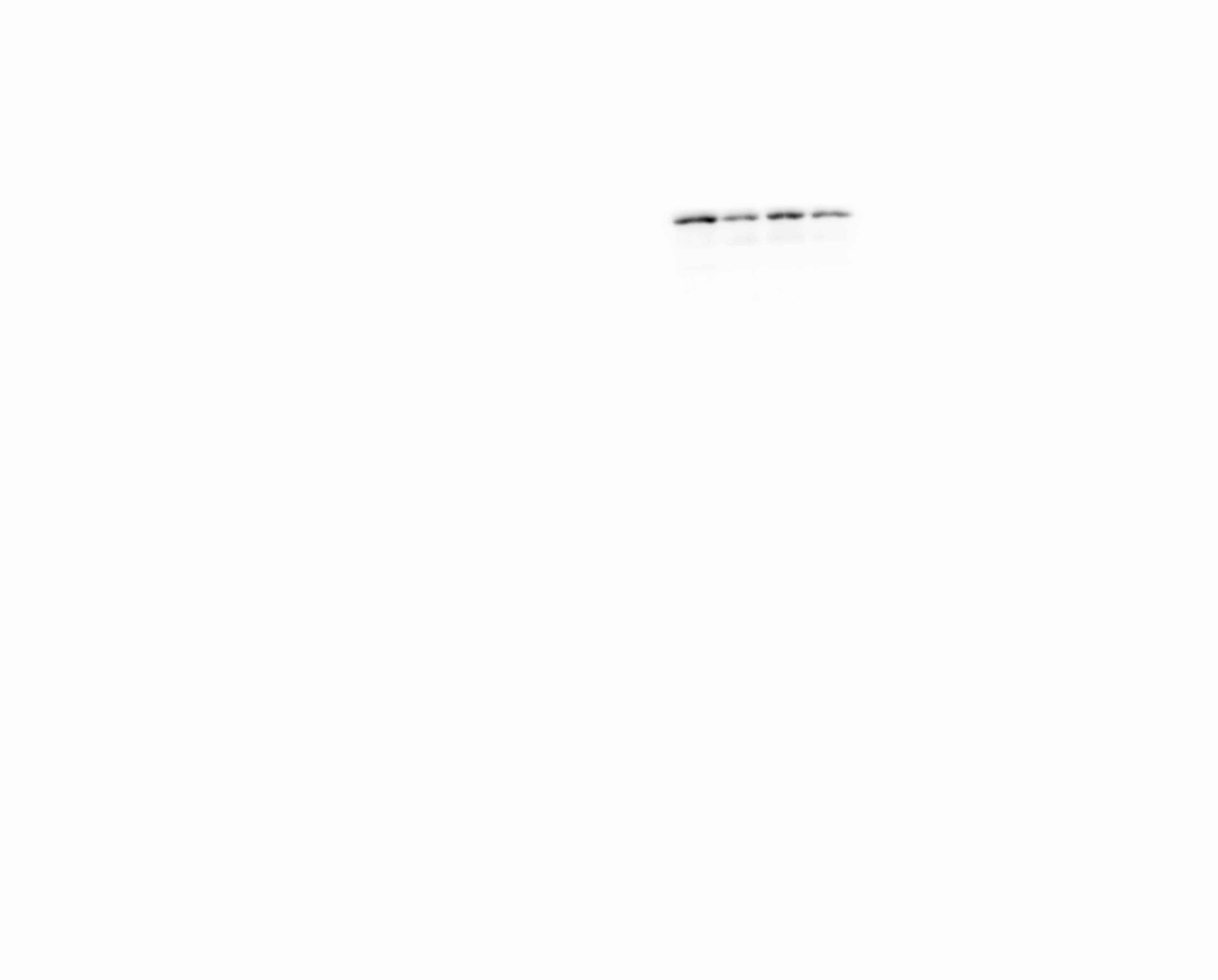

Supplement: Supplementary file 2 — Supporting File 2: advs73976‐sup‐0002‐SuppMat.zip. [file ADVS-13-e11217-s002.zip › WB#U4ee3#U8868#U56fe/xiap#U539f#U59cb#U6570#U636ewb1-JPEG/eif2a_4 #U4ee3#U8868 sheif#U6548#U7387.jpg]

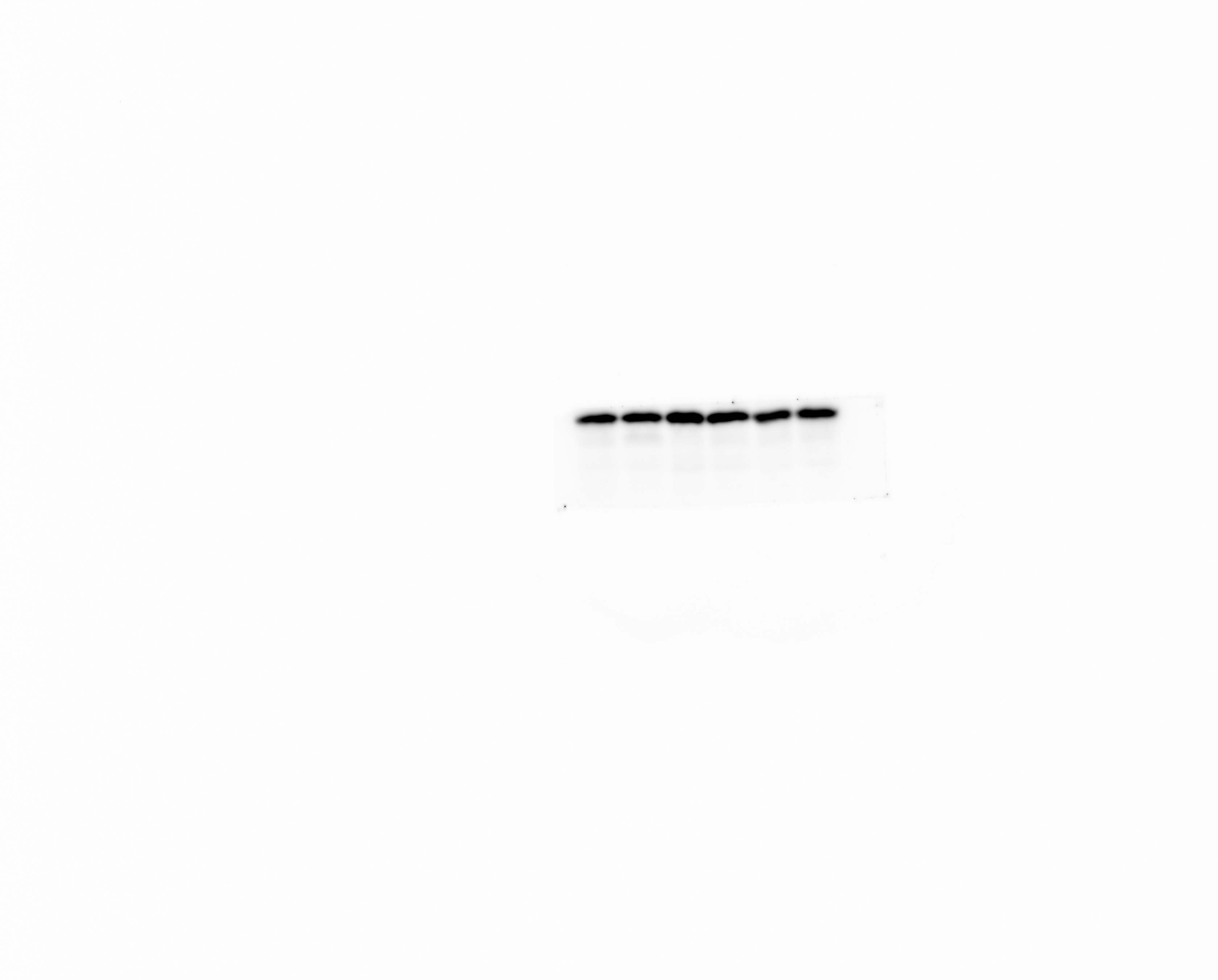

Supplement: Supplementary file 2 — Supporting File 2: advs73976‐sup‐0002‐SuppMat.zip. [file ADVS-13-e11217-s002.zip › WB#U4ee3#U8868#U56fe/xiap#U539f#U59cb#U6570#U636ewb1-JPEG/EIF2A_7.jpg]

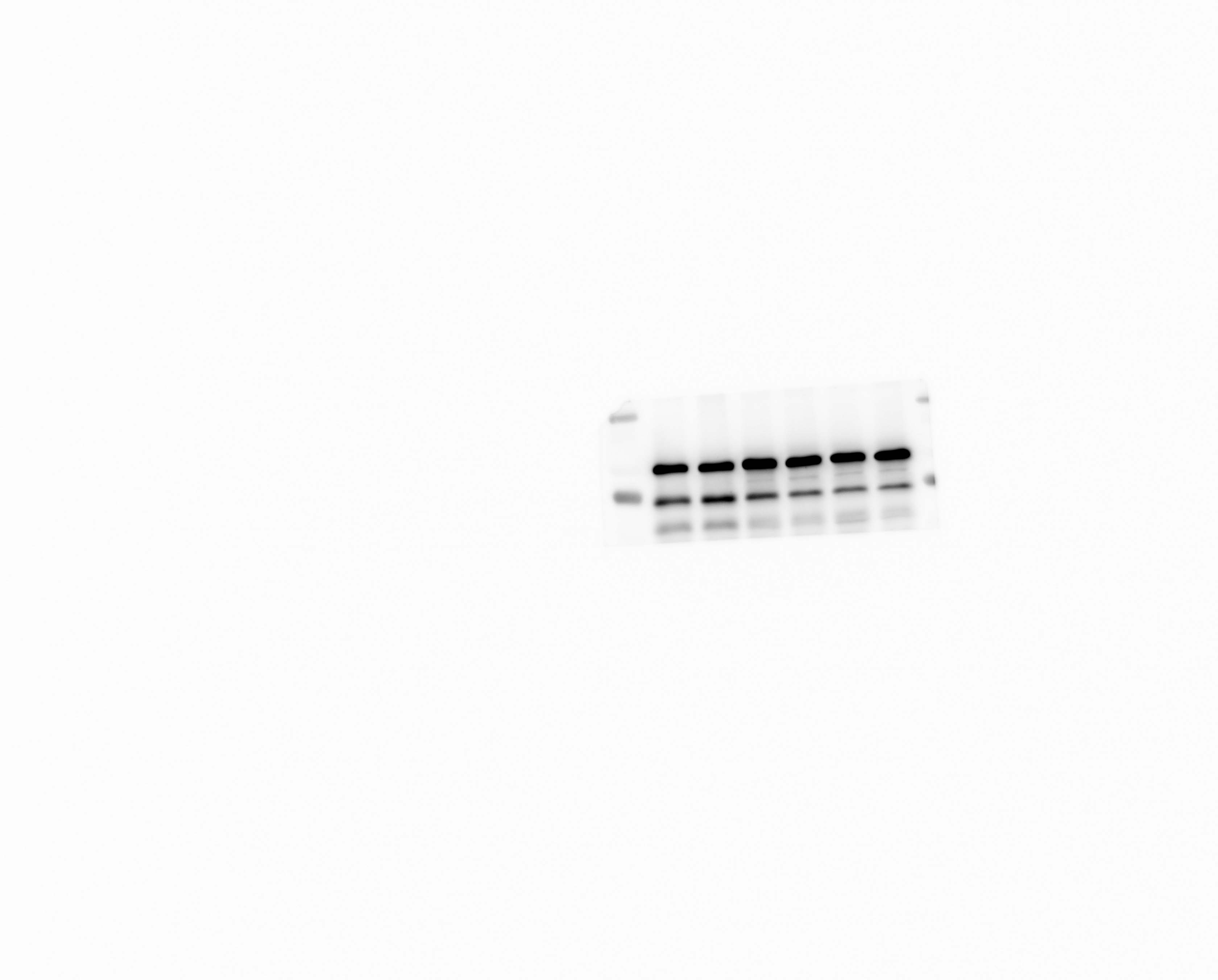

Supplement: Supplementary file 2 — Supporting File 2: advs73976‐sup‐0002‐SuppMat.zip. [file ADVS-13-e11217-s002.zip › WB#U4ee3#U8868#U56fe/xiap#U539f#U59cb#U6570#U636ewb1-JPEG/FAM134B_6#U4ee3#U8868.jpg]

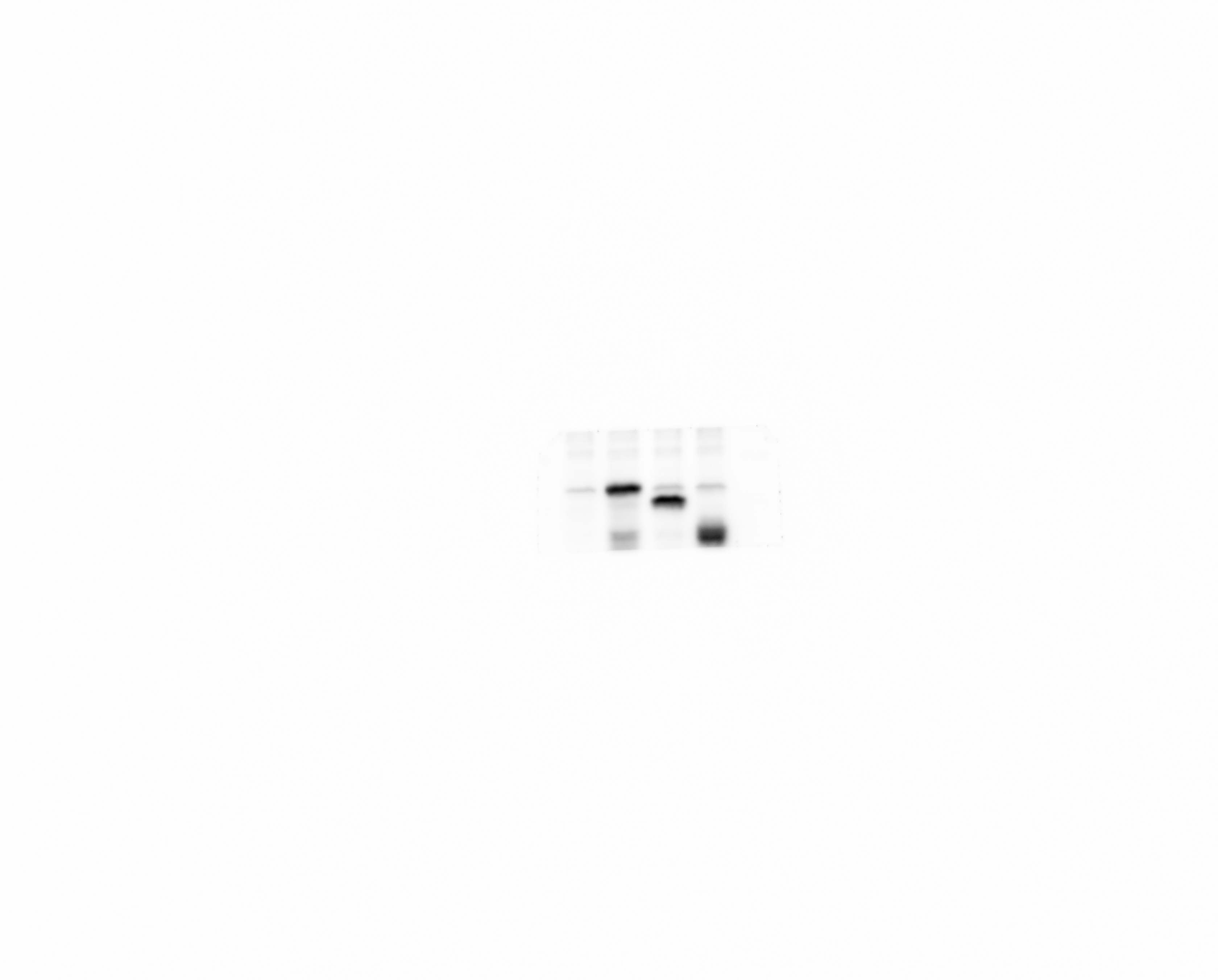

Supplement: Supplementary file 2 — Supporting File 2: advs73976‐sup‐0002‐SuppMat.zip. [file ADVS-13-e11217-s002.zip › WB#U4ee3#U8868#U56fe/xiap#U539f#U59cb#U6570#U636ewb1-JPEG/flag 4 db m12.jpg]

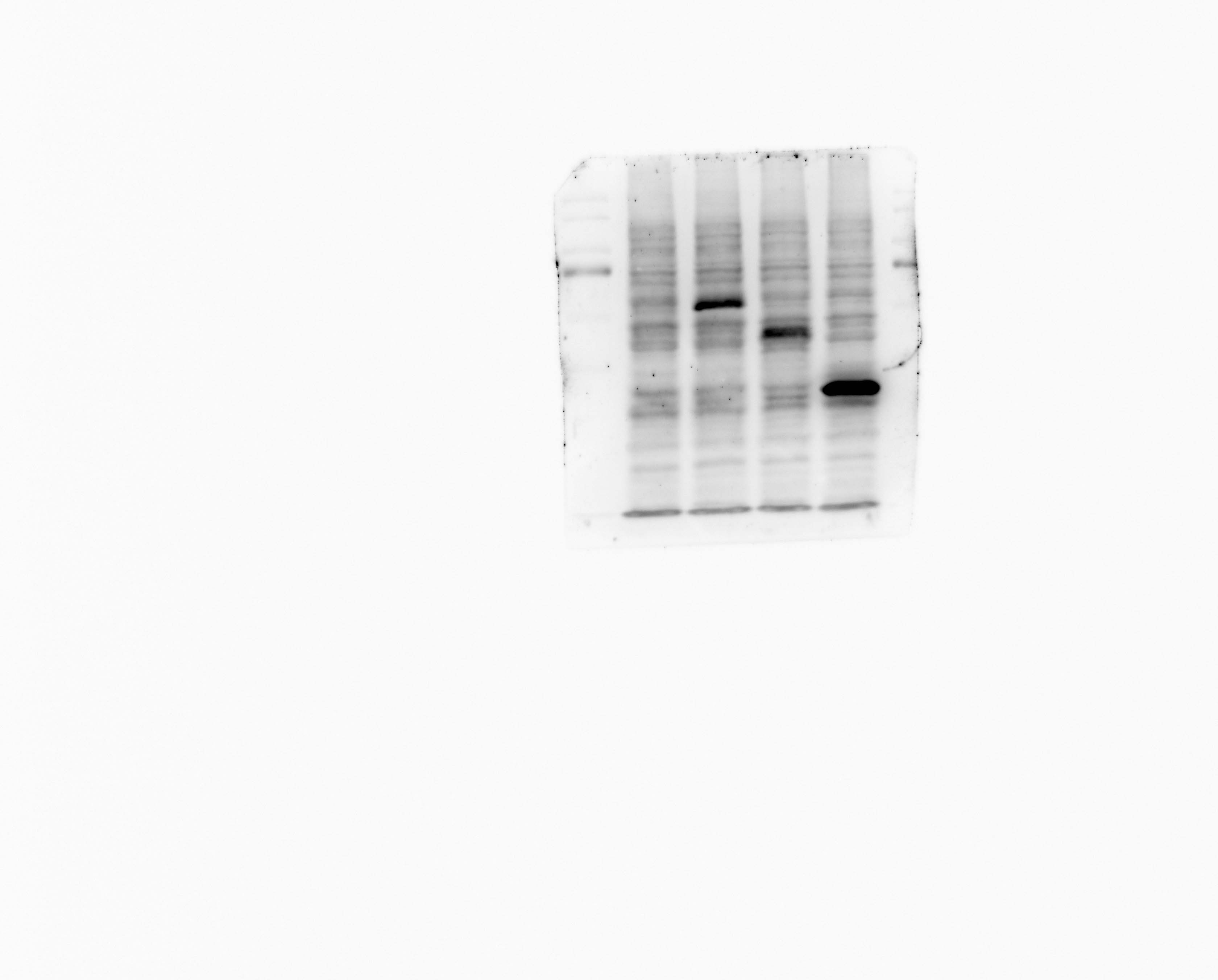

Supplement: Supplementary file 2 — Supporting File 2: advs73976‐sup‐0002‐SuppMat.zip. [file ADVS-13-e11217-s002.zip › WB#U4ee3#U8868#U56fe/xiap#U539f#U59cb#U6570#U636ewb1-JPEG/FLAG1_13 db m34.jpg]

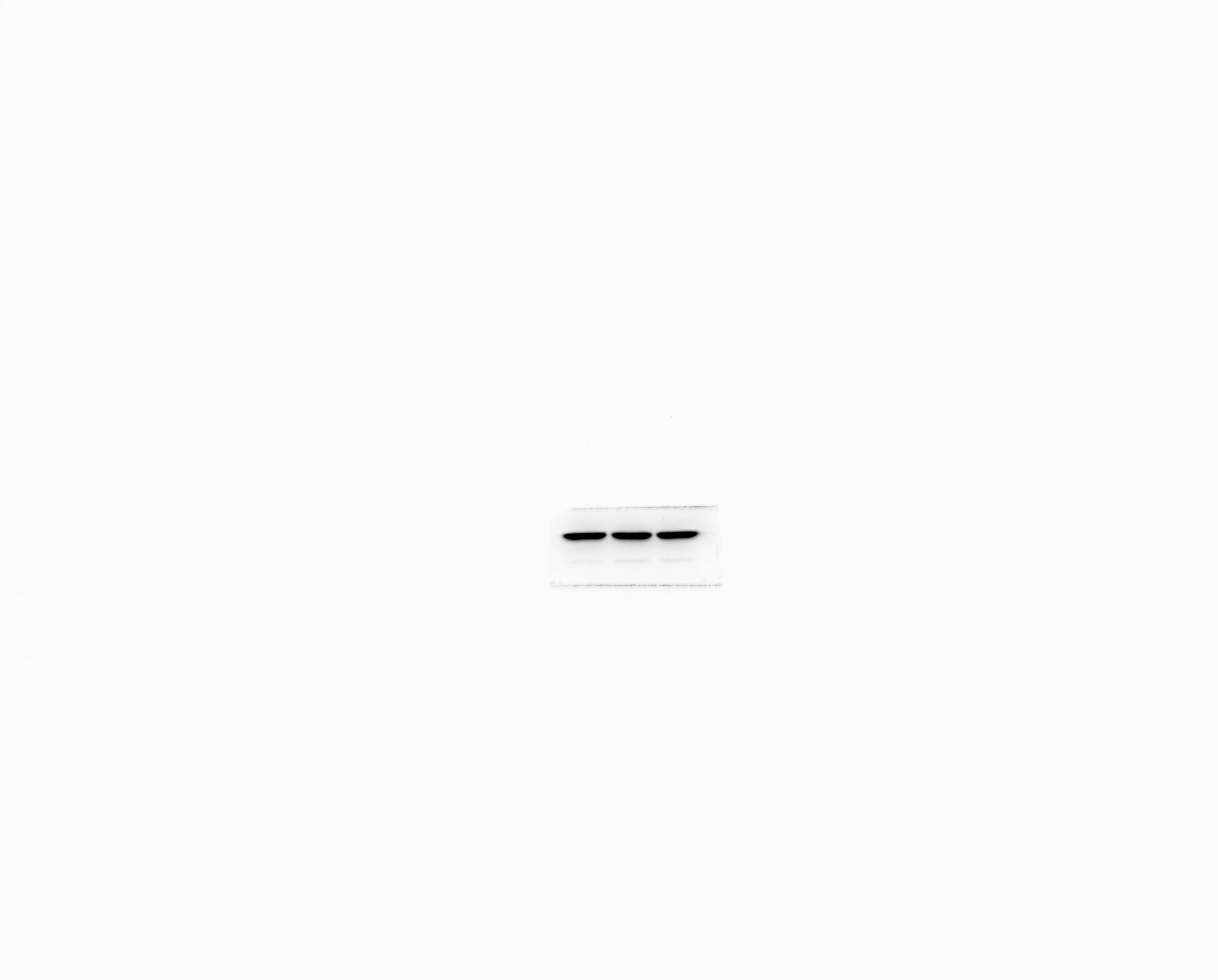

Supplement: Supplementary file 2 — Supporting File 2: advs73976‐sup‐0002‐SuppMat.zip. [file ADVS-13-e11217-s002.zip › WB#U4ee3#U8868#U56fe/xiap#U539f#U59cb#U6570#U636ewb1-JPEG/gap-_7 c-casps3 oex.jpg]

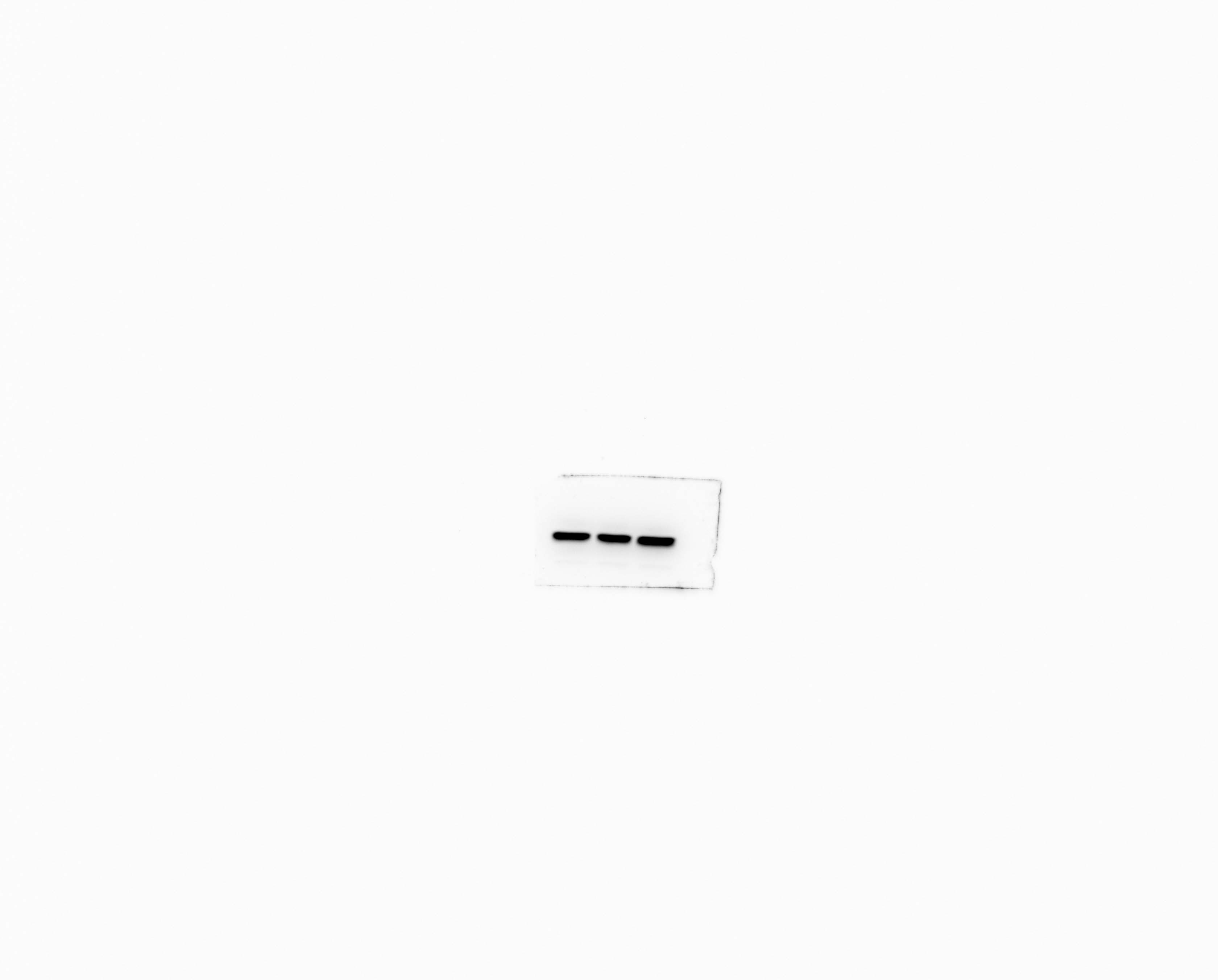

Supplement: Supplementary file 2 — Supporting File 2: advs73976‐sup‐0002‐SuppMat.zip. [file ADVS-13-e11217-s002.zip › WB#U4ee3#U8868#U56fe/xiap#U539f#U59cb#U6570#U636ewb1-JPEG/gap-_7#U4ee3#U8868 p62 oex.jpg]

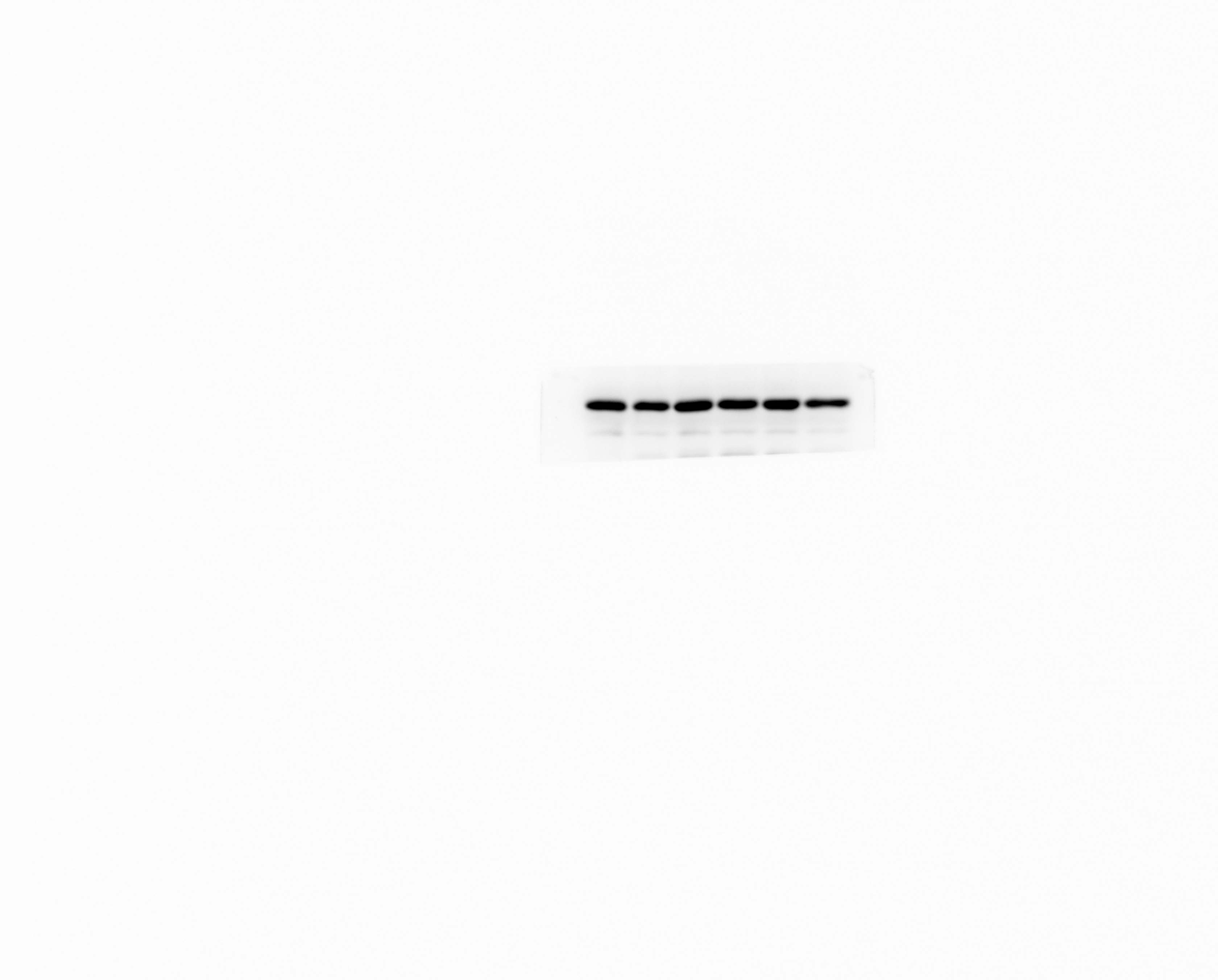

Supplement: Supplementary file 2 — Supporting File 2: advs73976‐sup‐0002‐SuppMat.zip. [file ADVS-13-e11217-s002.zip › WB#U4ee3#U8868#U56fe/xiap#U539f#U59cb#U6570#U636ewb1-JPEG/gapcut_6 DK.jpg]

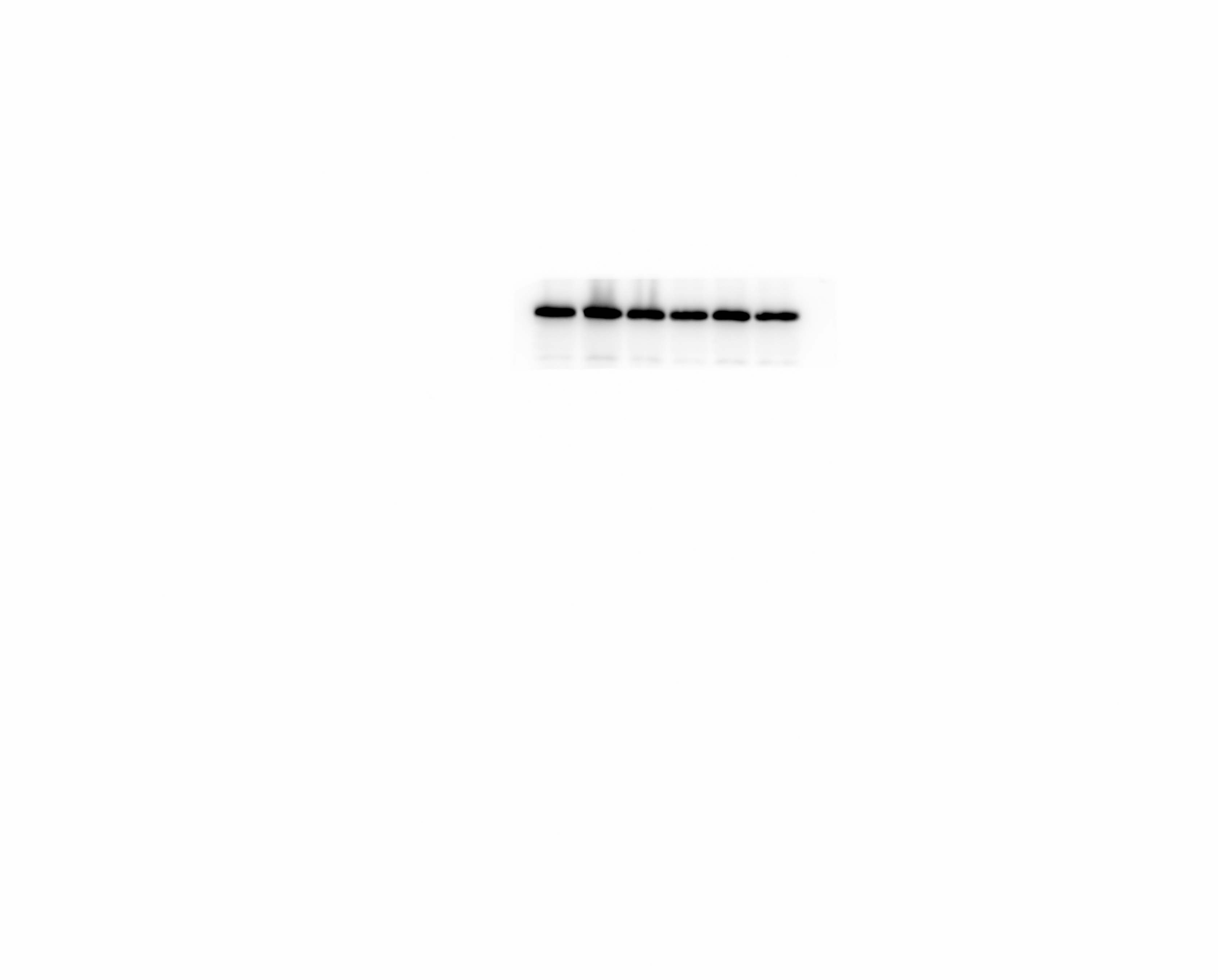

Supplement: Supplementary file 2 — Supporting File 2: advs73976‐sup‐0002‐SuppMat.zip. [file ADVS-13-e11217-s002.zip › WB#U4ee3#U8868#U56fe/xiap#U539f#U59cb#U6570#U636ewb1-JPEG/GAPDH-_6-#U4ee3#U8868 xiap.jpg]

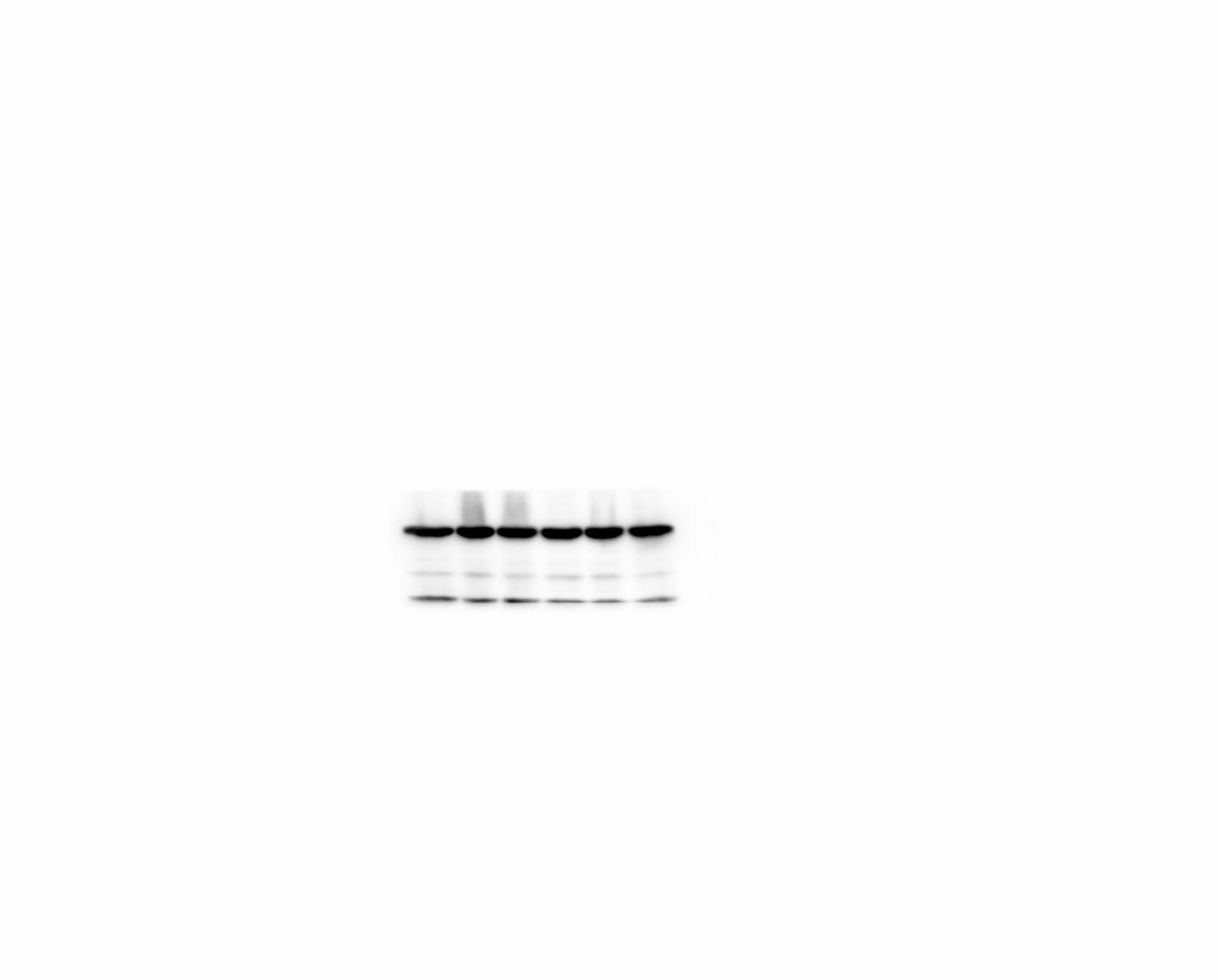

Supplement: Supplementary file 2 — Supporting File 2: advs73976‐sup‐0002‐SuppMat.zip. [file ADVS-13-e11217-s002.zip › WB#U4ee3#U8868#U56fe/xiap#U539f#U59cb#U6570#U636ewb1-JPEG/GAPDH_4#U4ee3#U8868 xiap oex.jpg]

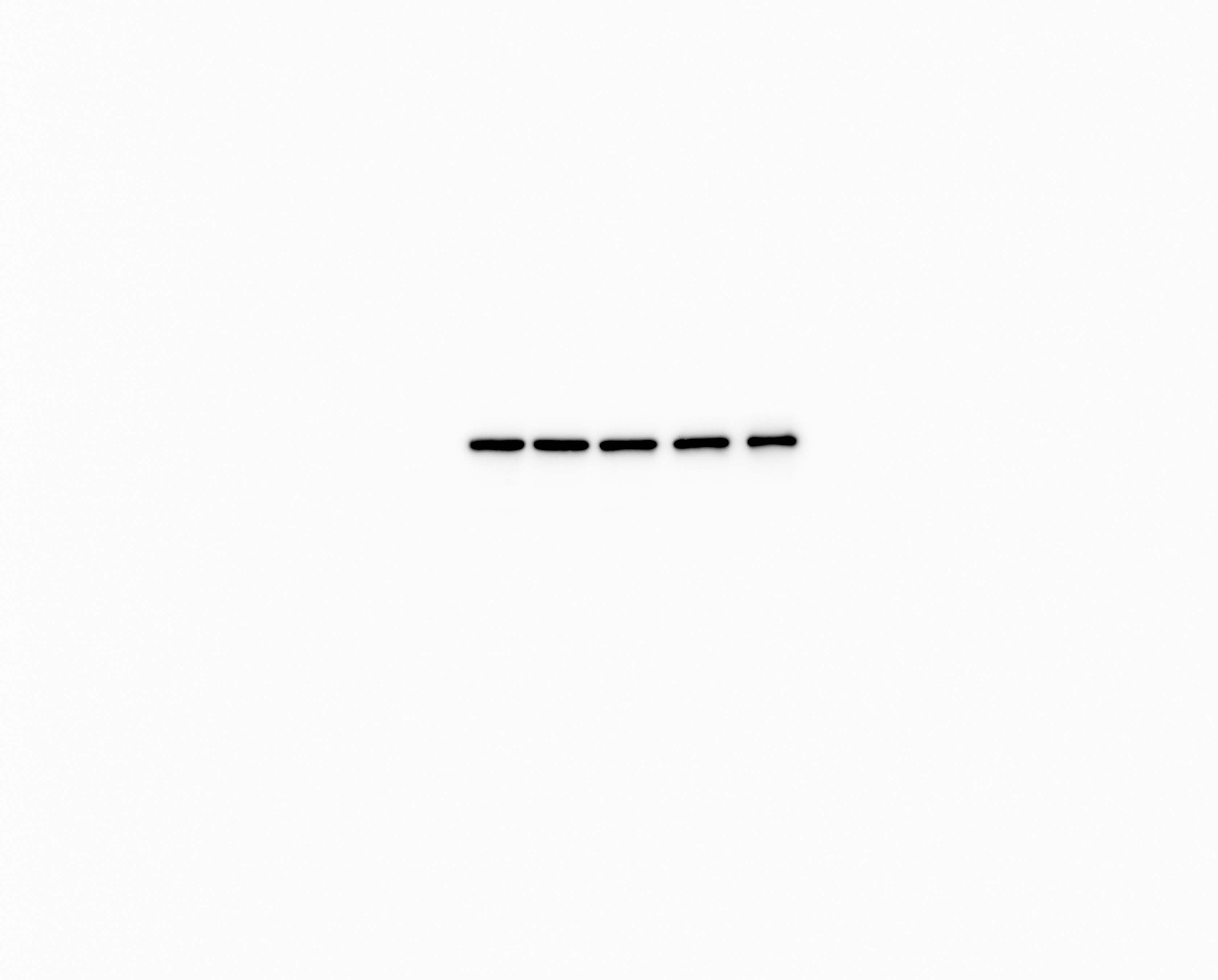

Supplement: Supplementary file 2 — Supporting File 2: advs73976‐sup‐0002‐SuppMat.zip. [file ADVS-13-e11217-s002.zip › WB#U4ee3#U8868#U56fe/xiap#U539f#U59cb#U6570#U636ewb1-JPEG/gapdh_6 six.jpg]

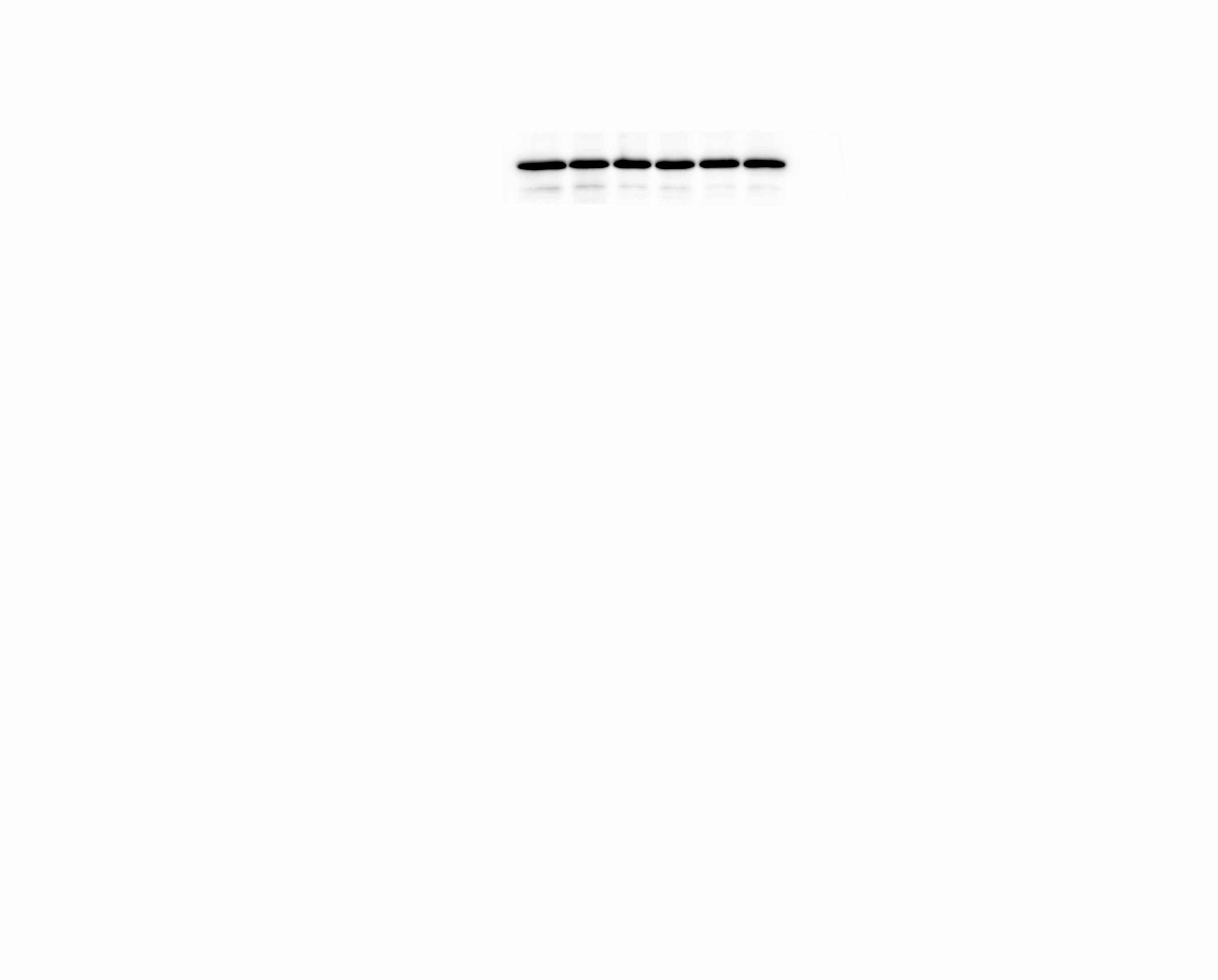

Supplement: Supplementary file 2 — Supporting File 2: advs73976‐sup‐0002‐SuppMat.zip. [file ADVS-13-e11217-s002.zip › WB#U4ee3#U8868#U56fe/xiap#U539f#U59cb#U6570#U636ewb1-JPEG/GAPDH_7#U4ee3c-casps3.jpg]

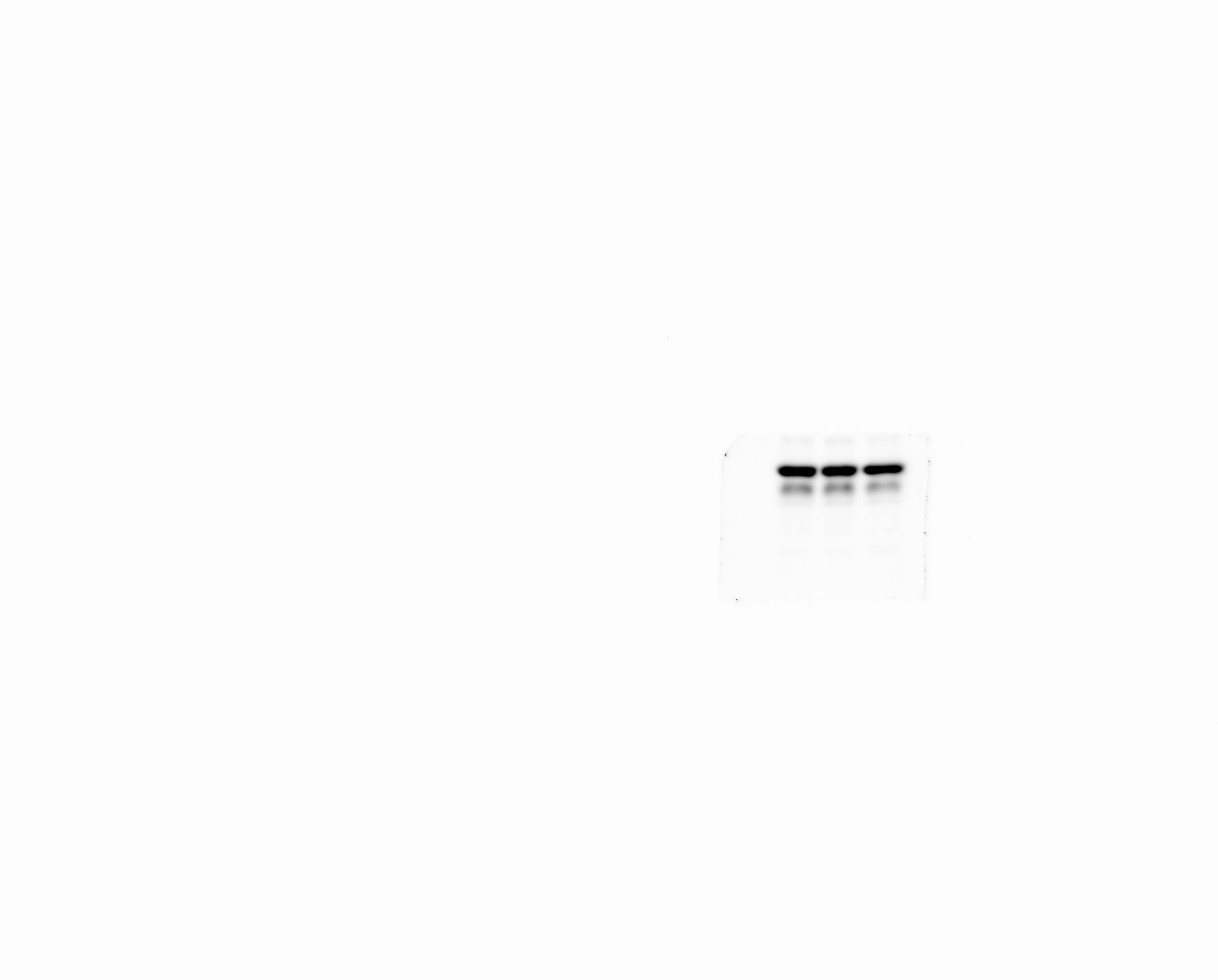

Supplement: Supplementary file 2 — Supporting File 2: advs73976‐sup‐0002‐SuppMat.zip. [file ADVS-13-e11217-s002.zip › WB#U4ee3#U8868#U56fe/xiap#U539f#U59cb#U6570#U636ewb1-JPEG/GAP_10 -lamp2 six.jpg]

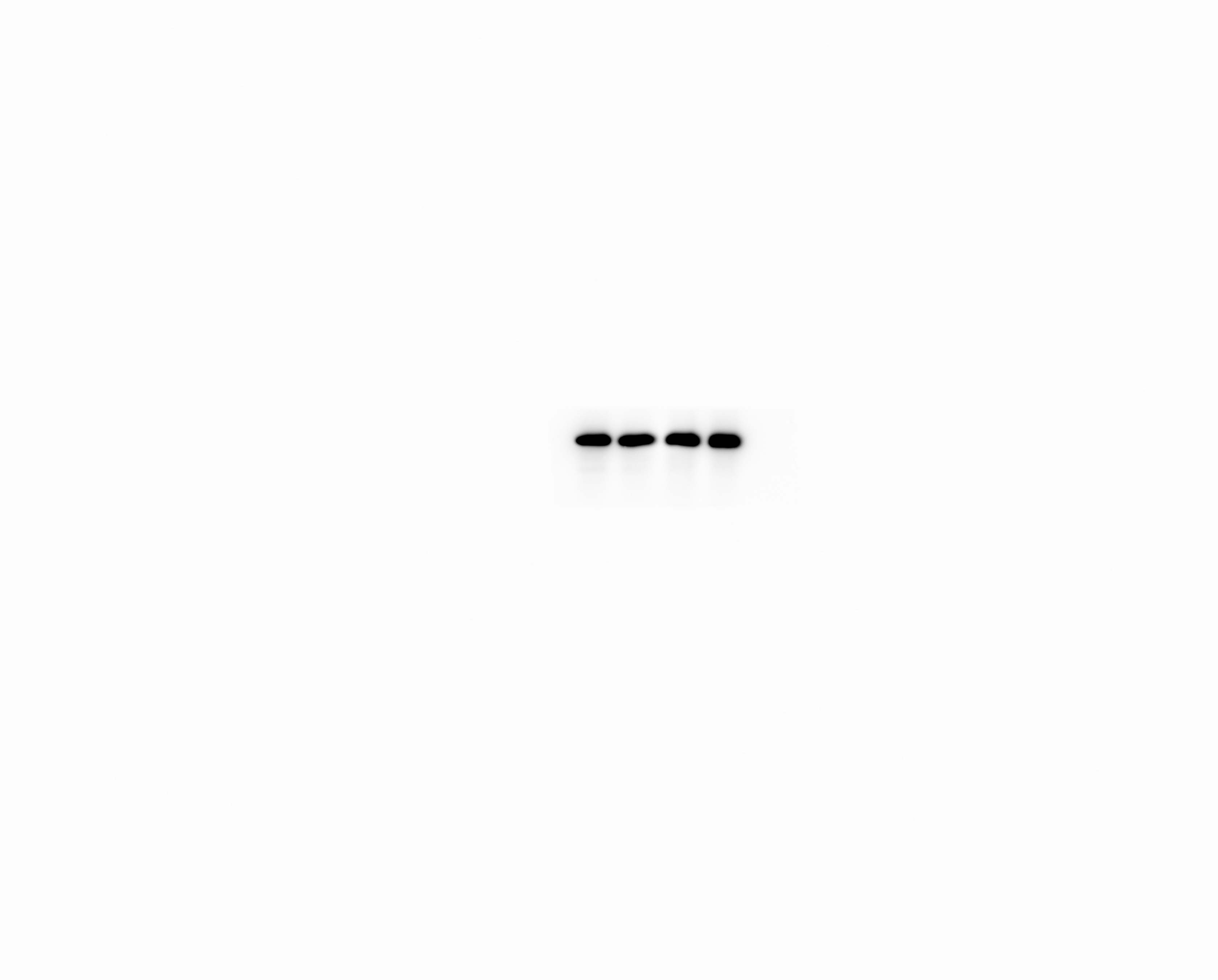

Supplement: Supplementary file 2 — Supporting File 2: advs73976‐sup‐0002‐SuppMat.zip. [file ADVS-13-e11217-s002.zip › WB#U4ee3#U8868#U56fe/xiap#U539f#U59cb#U6570#U636ewb1-JPEG/gap_10 db m12.jpg]

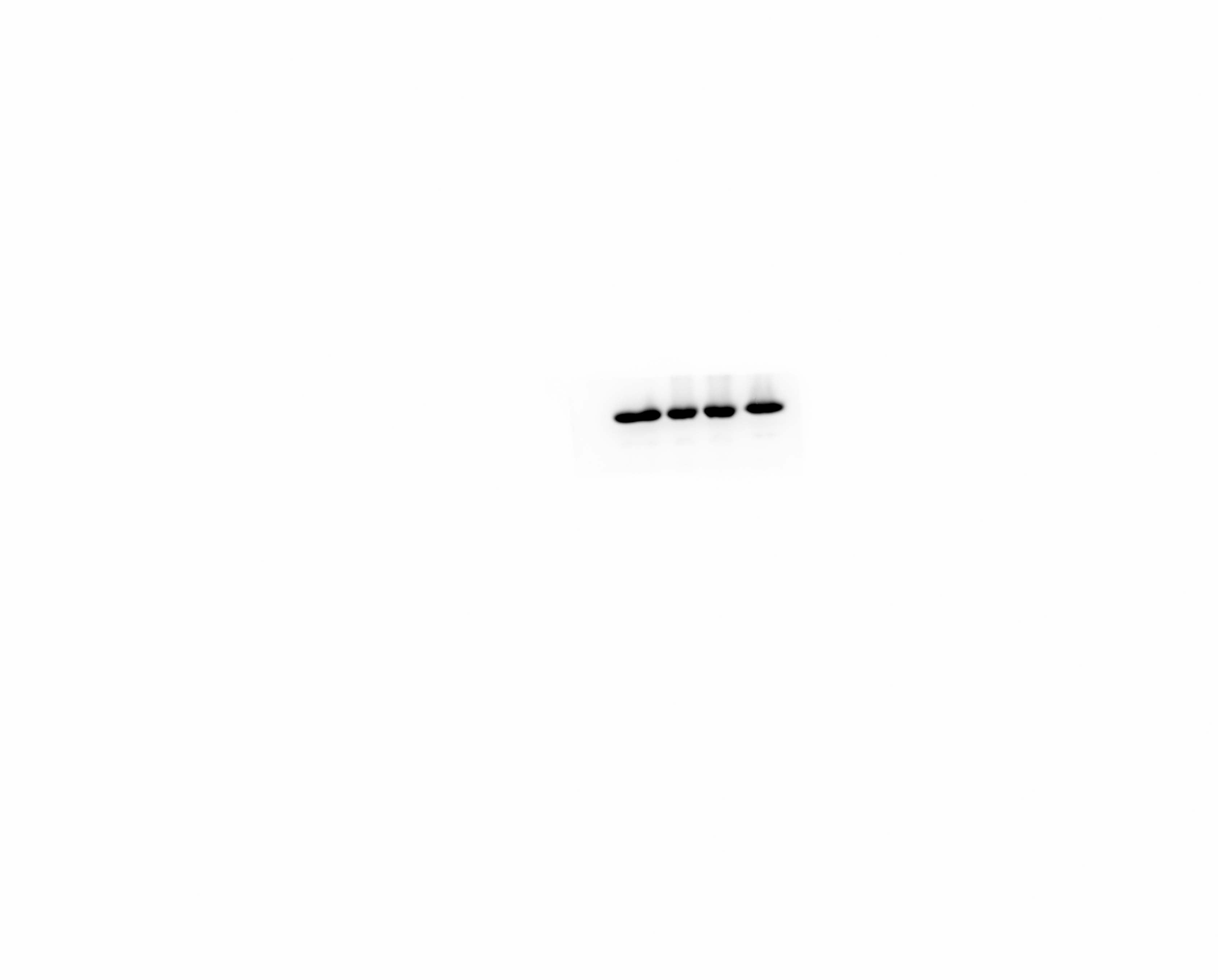

Supplement: Supplementary file 2 — Supporting File 2: advs73976‐sup‐0002‐SuppMat.zip. [file ADVS-13-e11217-s002.zip › WB#U4ee3#U8868#U56fe/xiap#U539f#U59cb#U6570#U636ewb1-JPEG/GAP_4 shatf4#U6548#U7387.jpg]

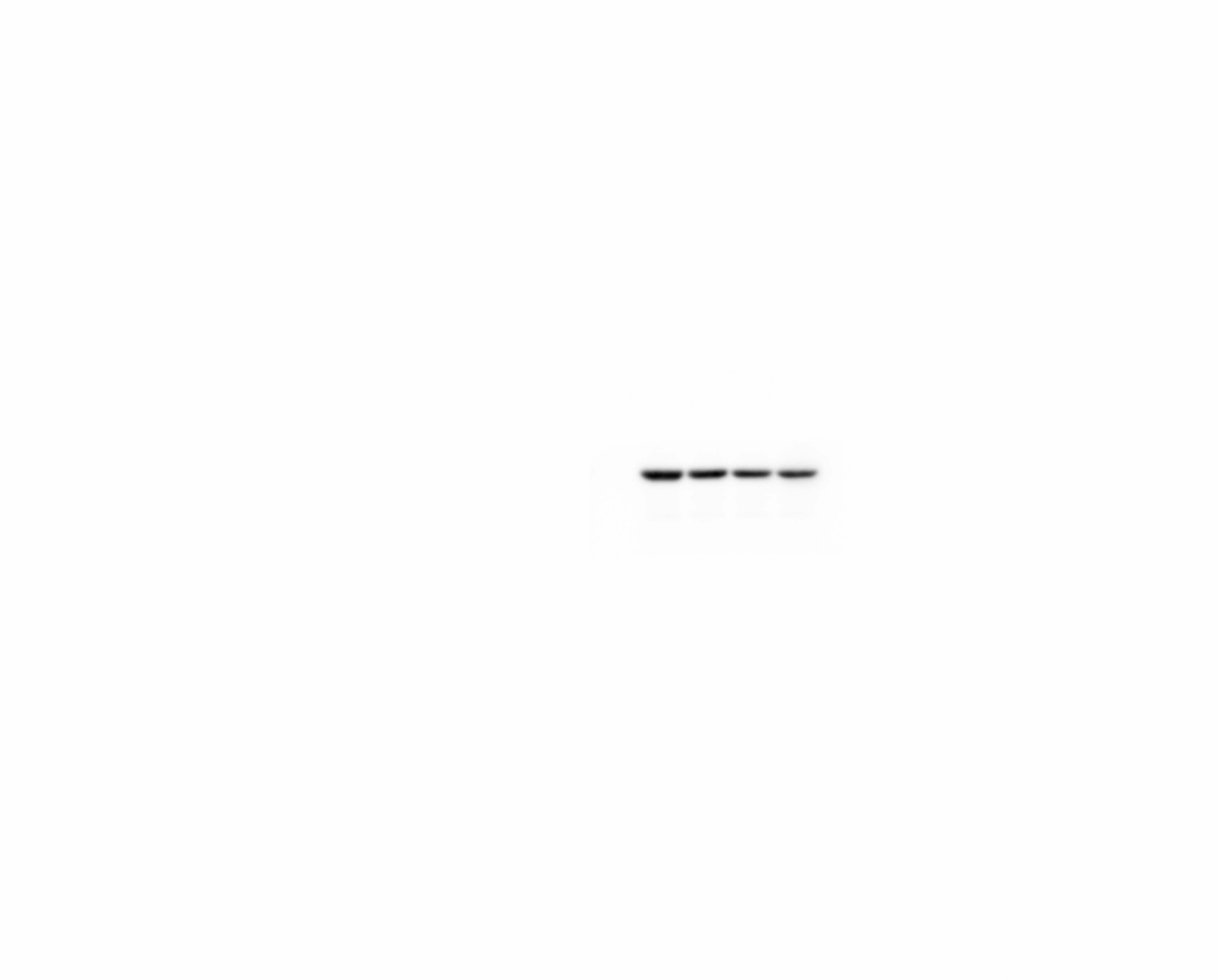

Supplement: Supplementary file 2 — Supporting File 2: advs73976‐sup‐0002‐SuppMat.zip. [file ADVS-13-e11217-s002.zip › WB#U4ee3#U8868#U56fe/xiap#U539f#U59cb#U6570#U636ewb1-JPEG/GAP_4#U4ee3#U8868 xiap mg12.jpg]

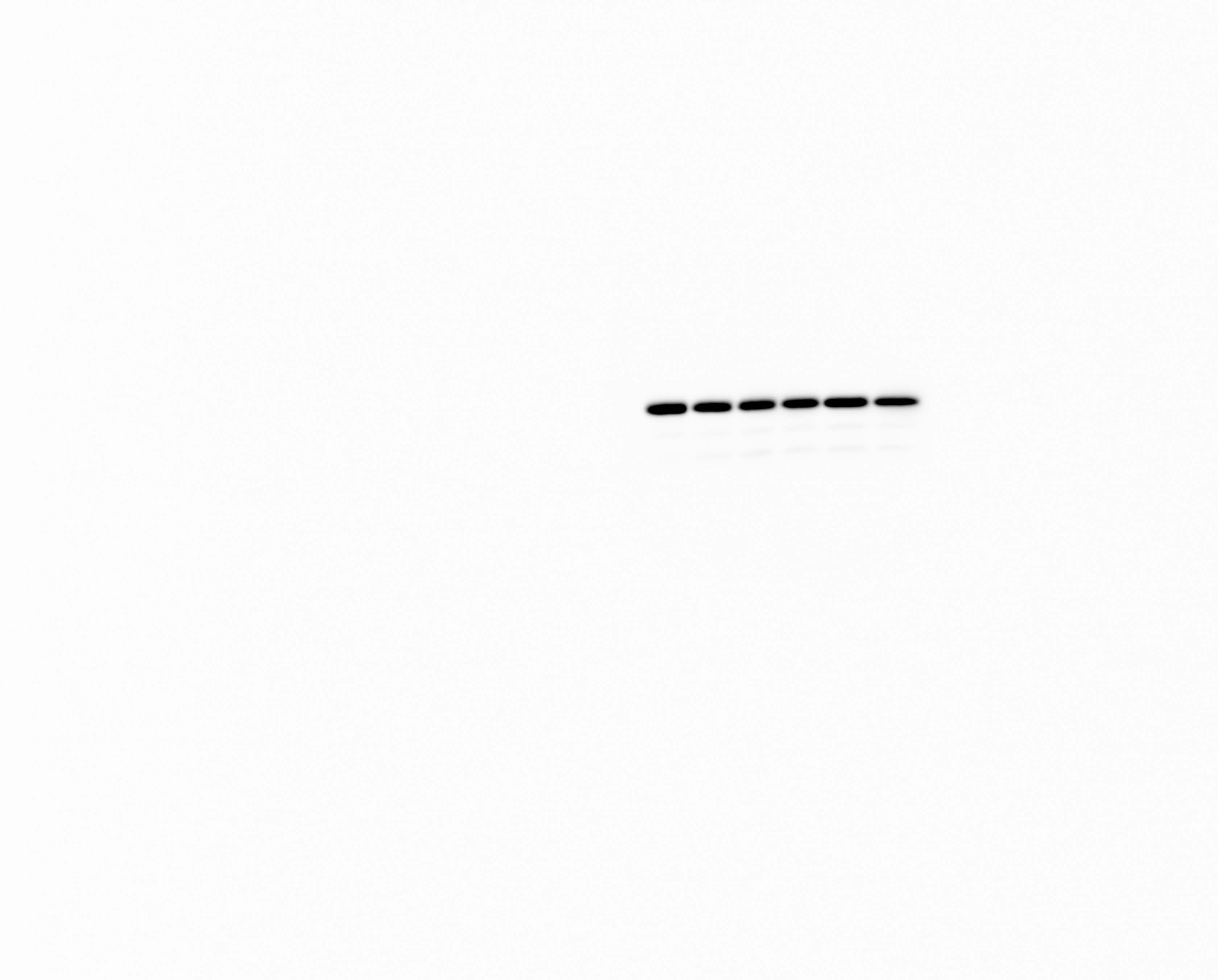

Supplement: Supplementary file 2 — Supporting File 2: advs73976‐sup‐0002‐SuppMat.zip. [file ADVS-13-e11217-s002.zip › WB#U4ee3#U8868#U56fe/xiap#U539f#U59cb#U6570#U636ewb1-JPEG/gap_6 xiap.jpg]

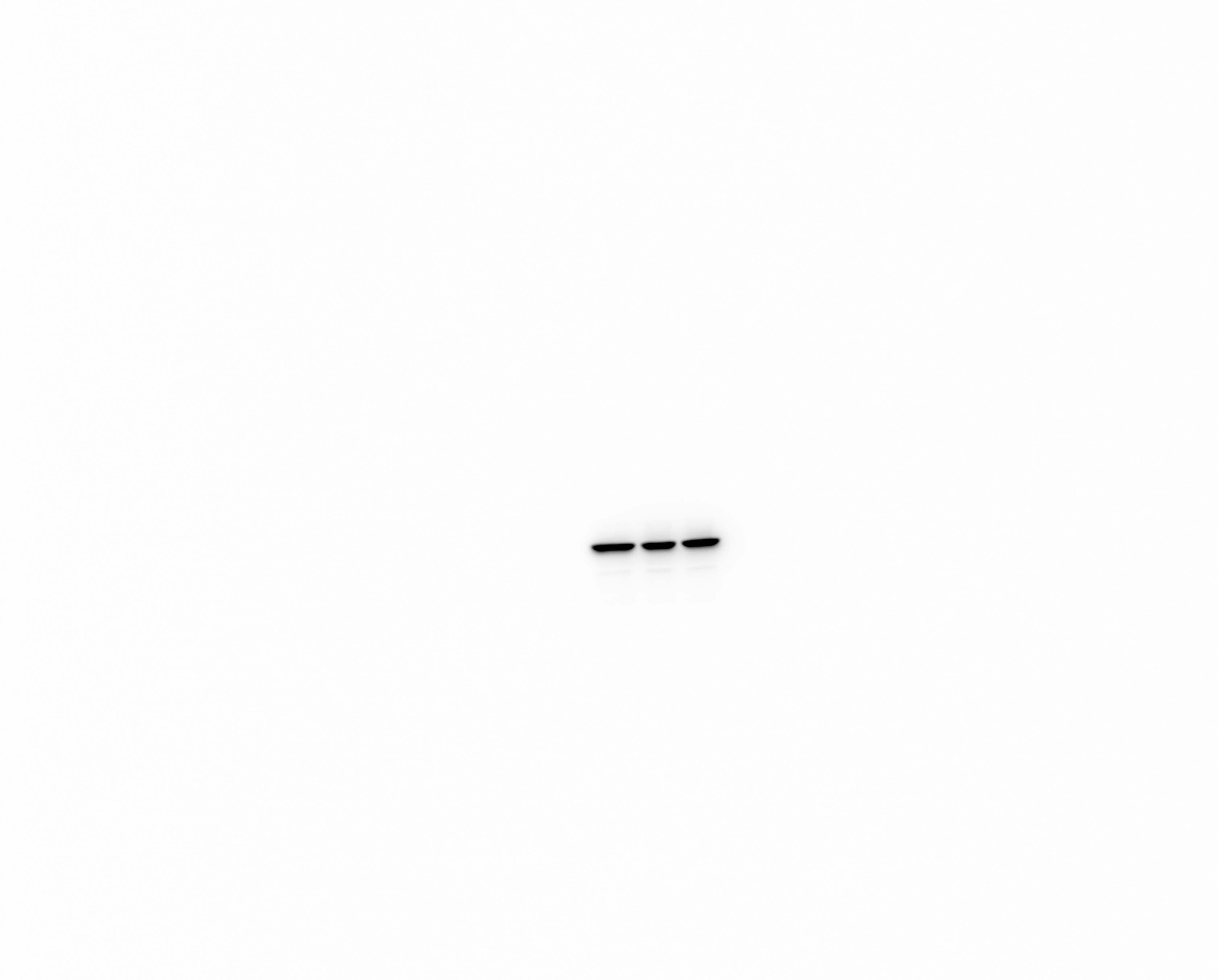

Supplement: Supplementary file 2 — Supporting File 2: advs73976‐sup‐0002‐SuppMat.zip. [file ADVS-13-e11217-s002.zip › WB#U4ee3#U8868#U56fe/xiap#U539f#U59cb#U6570#U636ewb1-JPEG/gap_6#U4ee3#U8868 SIXC-CASPS3.jpg]

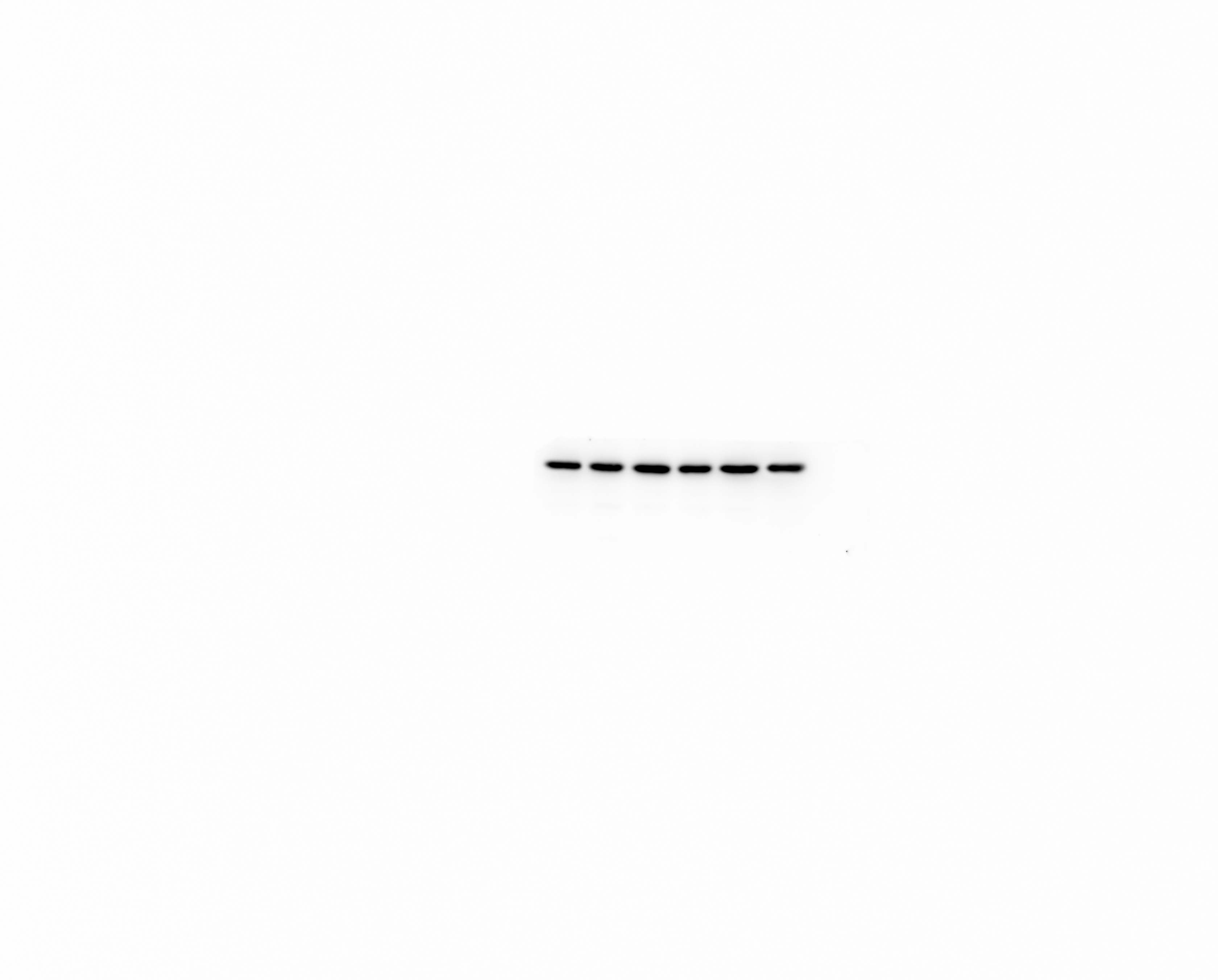

Supplement: Supplementary file 2 — Supporting File 2: advs73976‐sup‐0002‐SuppMat.zip. [file ADVS-13-e11217-s002.zip › WB#U4ee3#U8868#U56fe/xiap#U539f#U59cb#U6570#U636ewb1-JPEG/gap_6#U4ee3#U8868-3nt.jpg]

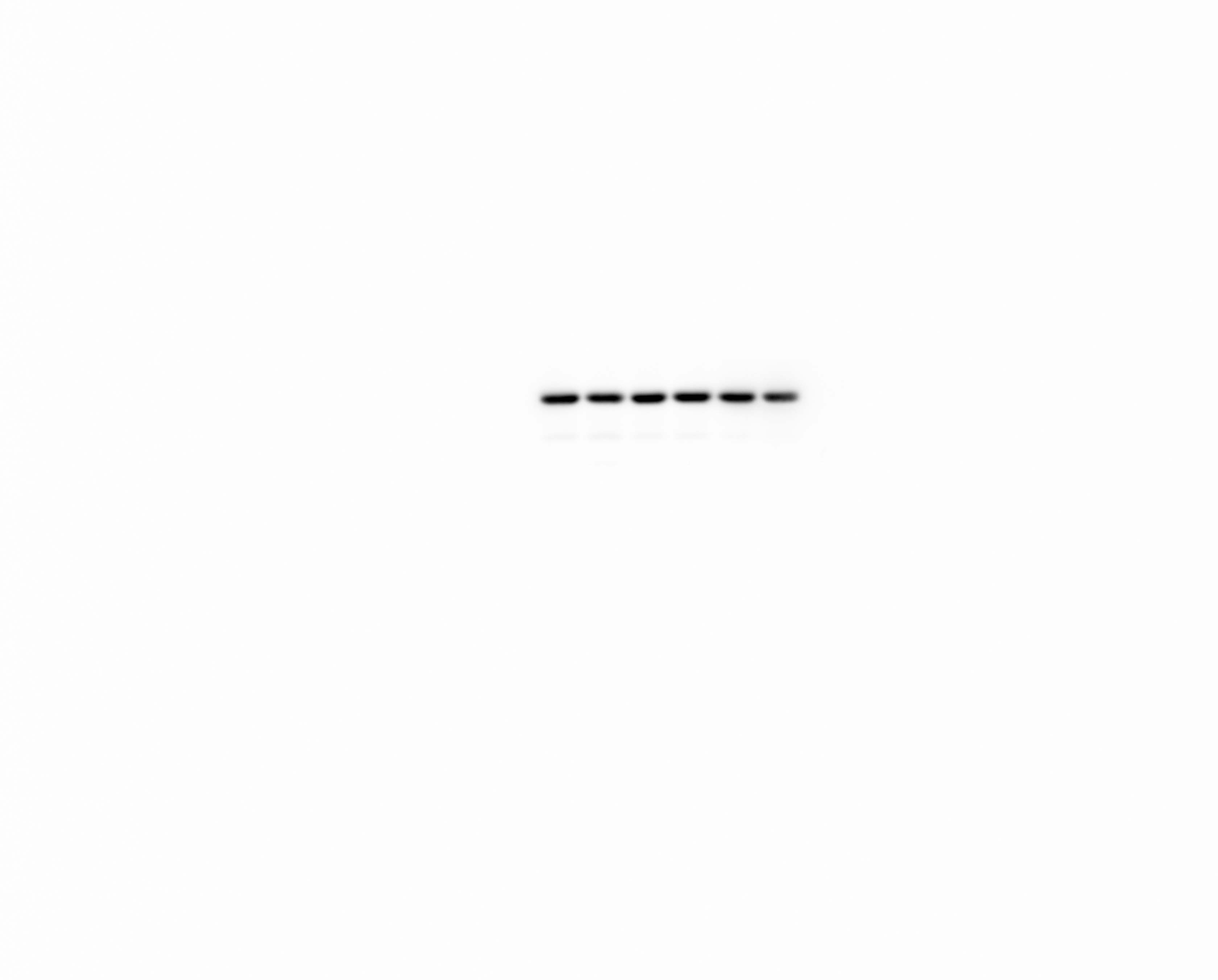

Supplement: Supplementary file 2 — Supporting File 2: advs73976‐sup‐0002‐SuppMat.zip. [file ADVS-13-e11217-s002.zip › WB#U4ee3#U8868#U56fe/xiap#U539f#U59cb#U6570#U636ewb1-JPEG/GAP_7#U4ee3#U8868-4hne.jpg]

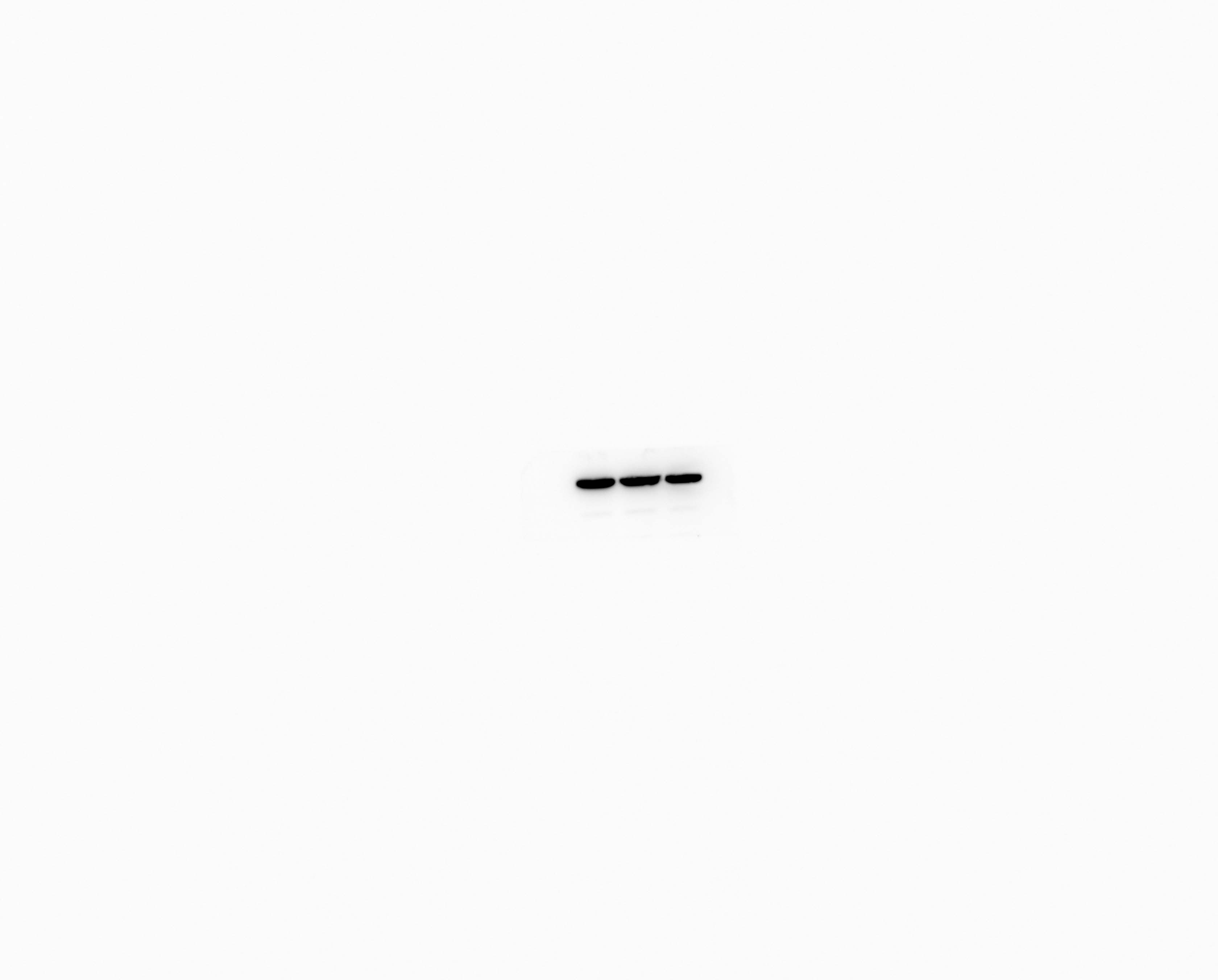

Supplement: Supplementary file 2 — Supporting File 2: advs73976‐sup‐0002‐SuppMat.zip. [file ADVS-13-e11217-s002.zip › WB#U4ee3#U8868#U56fe/xiap#U539f#U59cb#U6570#U636ewb1-JPEG/GAP_8#U4ee3#U8868 -lc3 six.jpg]

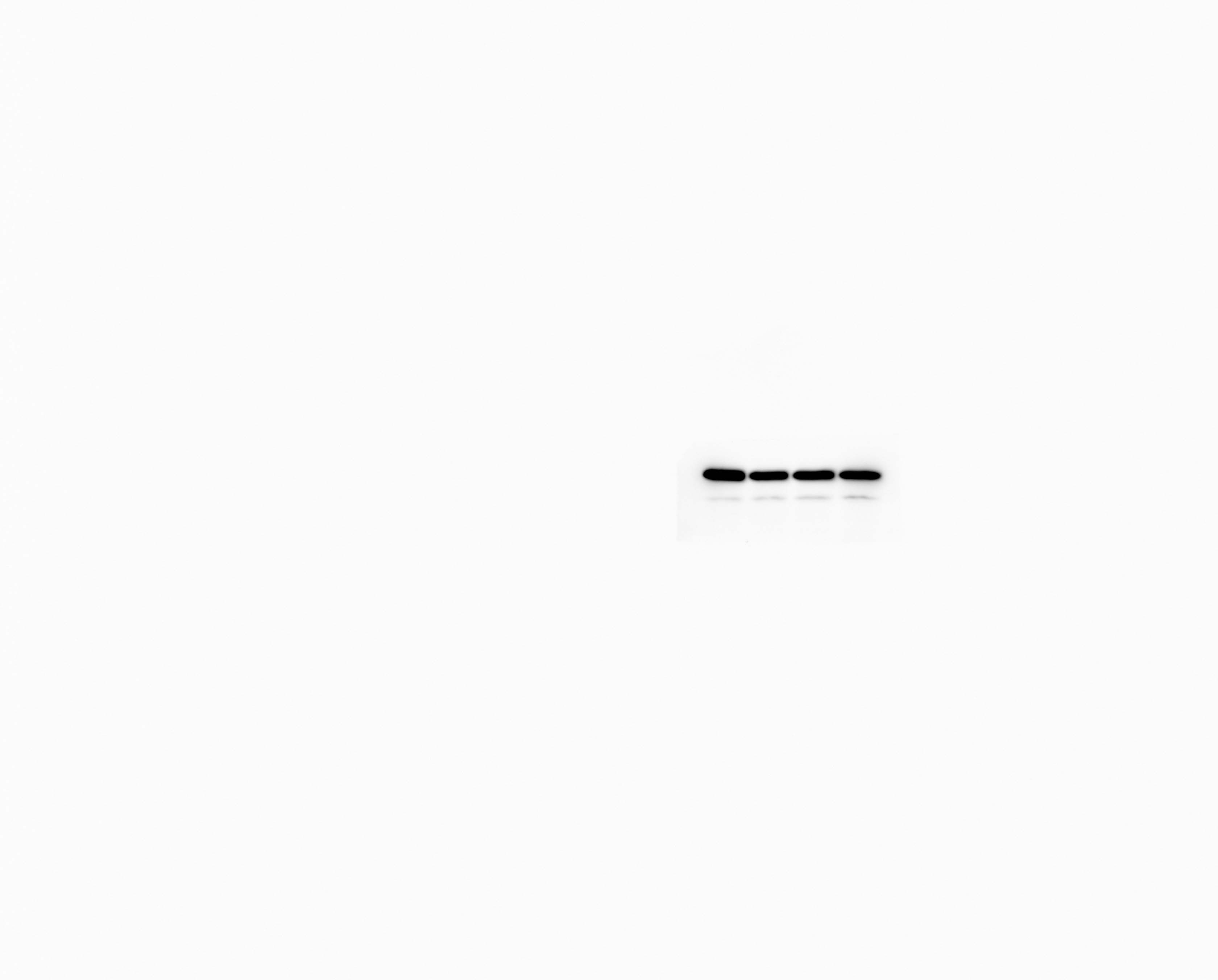

Supplement: Supplementary file 2 — Supporting File 2: advs73976‐sup‐0002‐SuppMat.zip. [file ADVS-13-e11217-s002.zip › WB#U4ee3#U8868#U56fe/xiap#U539f#U59cb#U6570#U636ewb1-JPEG/gap_9 xiap shatf4.jpg]

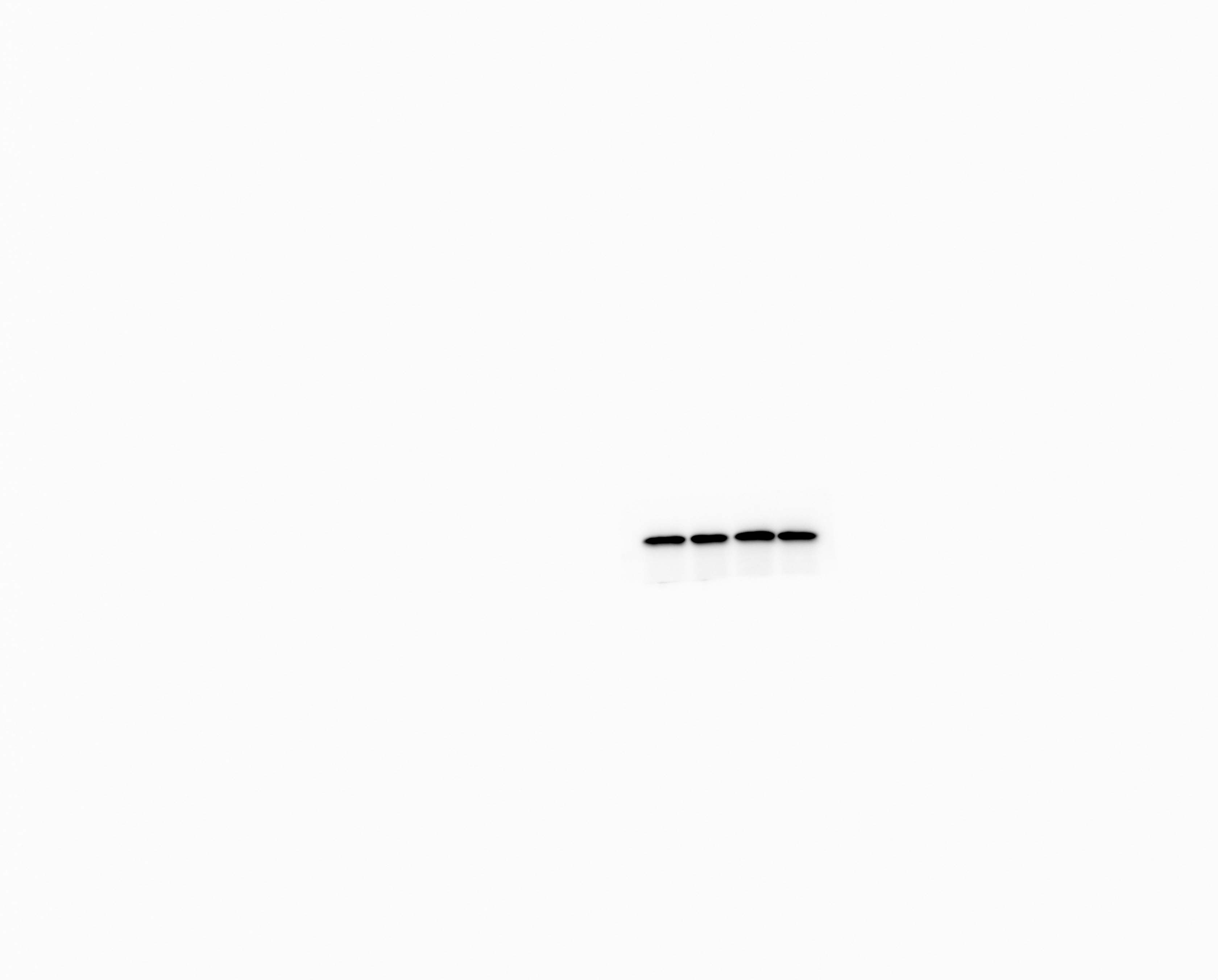

Supplement: Supplementary file 2 — Supporting File 2: advs73976‐sup‐0002‐SuppMat.zip. [file ADVS-13-e11217-s002.zip › WB#U4ee3#U8868#U56fe/xiap#U539f#U59cb#U6570#U636ewb1-JPEG/gap_9 #U4ee3#U8868 c-casps3.jpg]

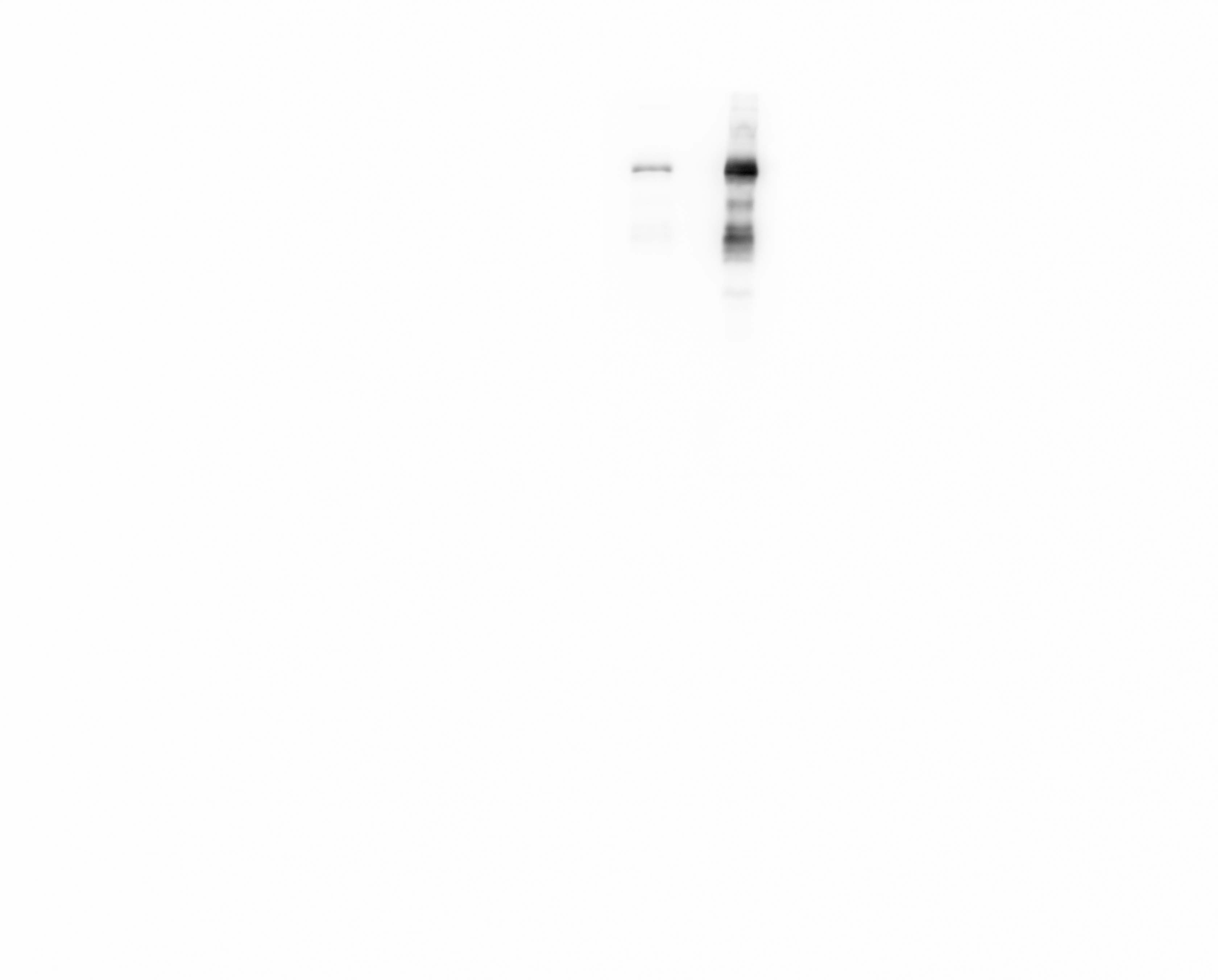

Supplement: Supplementary file 2 — Supporting File 2: advs73976‐sup‐0002‐SuppMat.zip. [file ADVS-13-e11217-s002.zip › WB#U4ee3#U8868#U56fe/xiap#U539f#U59cb#U6570#U636ewb1-JPEG/HA_2db ip lc3.jpg]

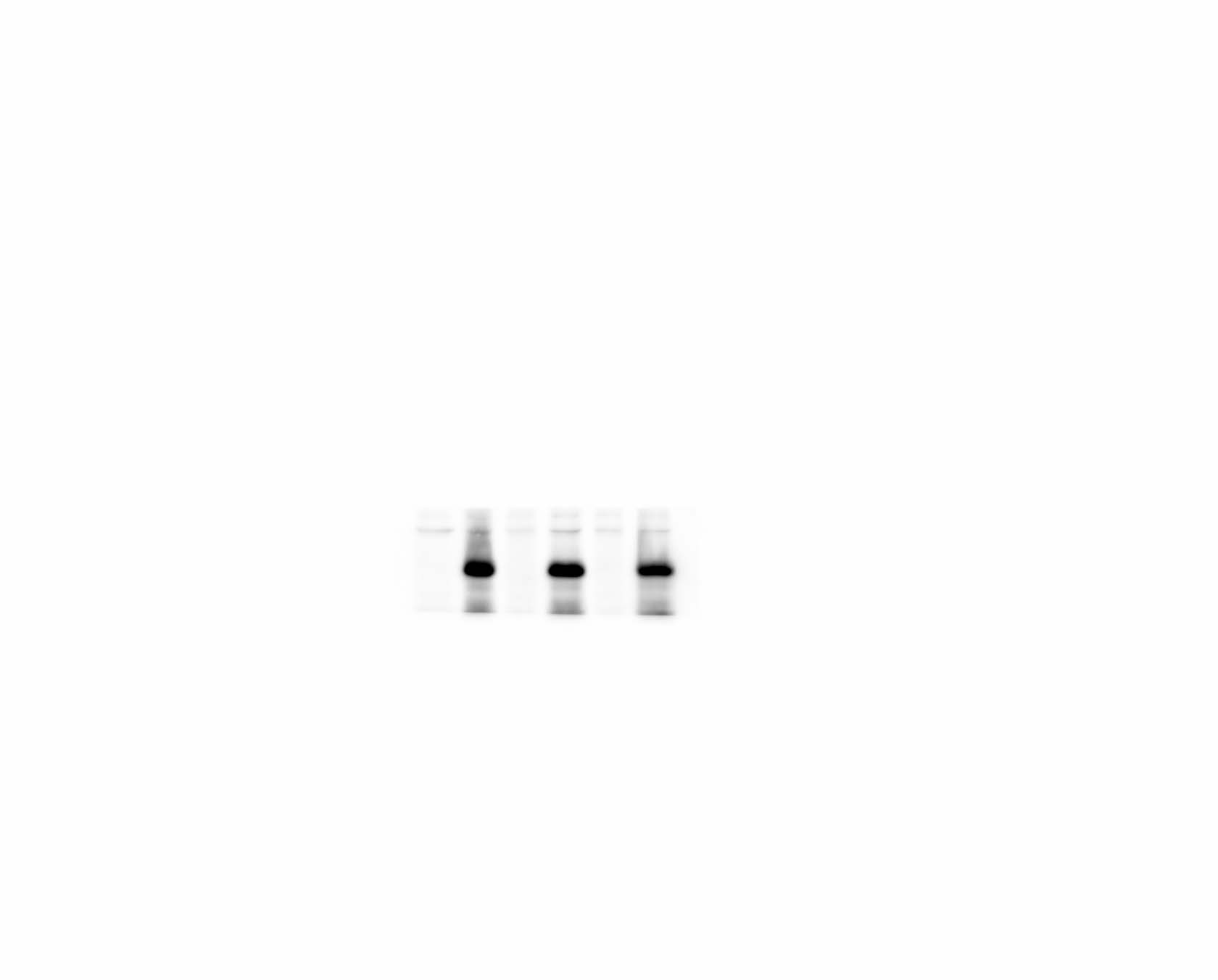

Supplement: Supplementary file 2 — Supporting File 2: advs73976‐sup‐0002‐SuppMat.zip. [file ADVS-13-e11217-s002.zip › WB#U4ee3#U8868#U56fe/xiap#U539f#U59cb#U6570#U636ewb1-JPEG/HA_5#U4ee3#U8868.jpg]

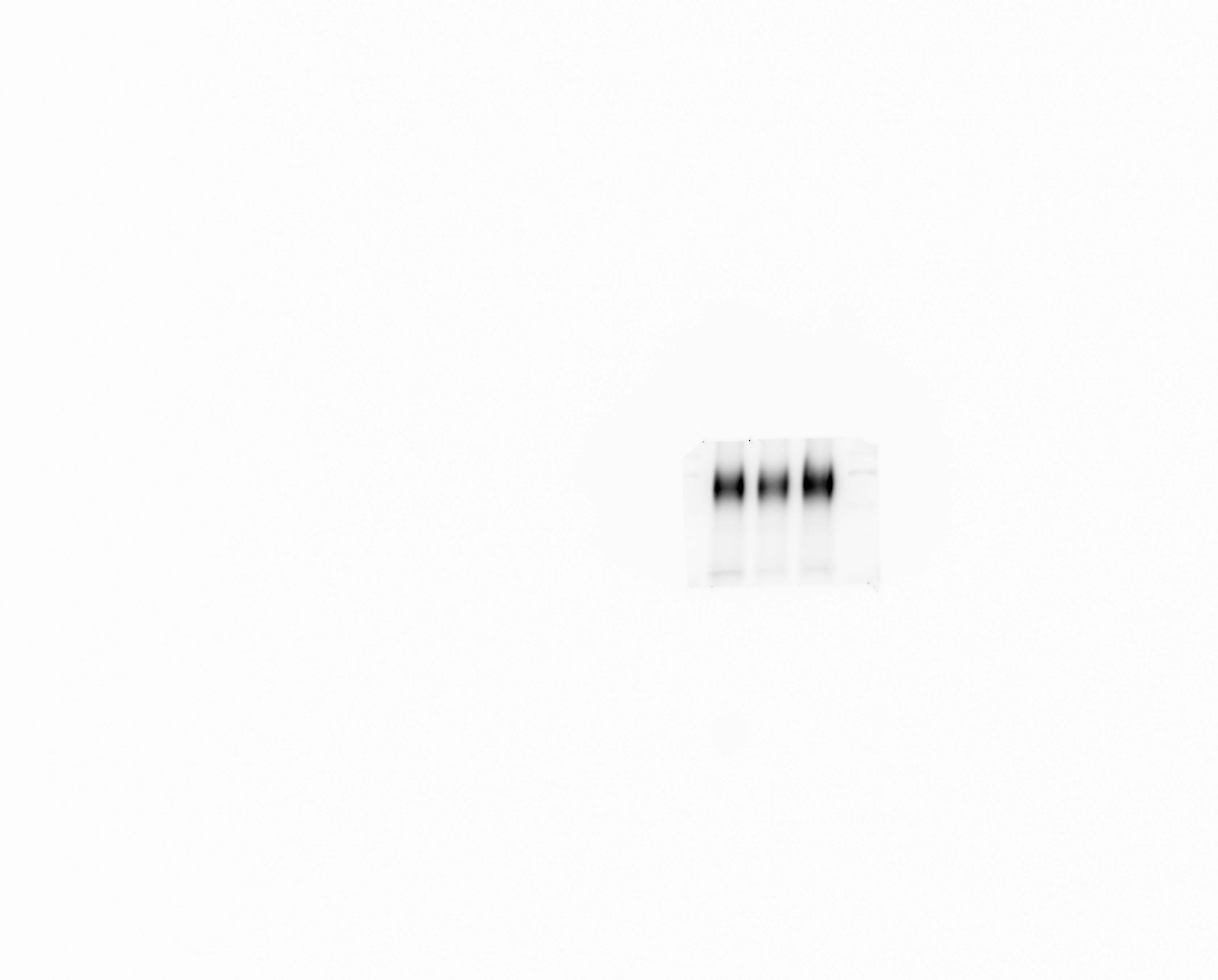

Supplement: Supplementary file 2 — Supporting File 2: advs73976‐sup‐0002‐SuppMat.zip. [file ADVS-13-e11217-s002.zip › WB#U4ee3#U8868#U56fe/xiap#U539f#U59cb#U6570#U636ewb1-JPEG/lamp1_6 oex.jpg]

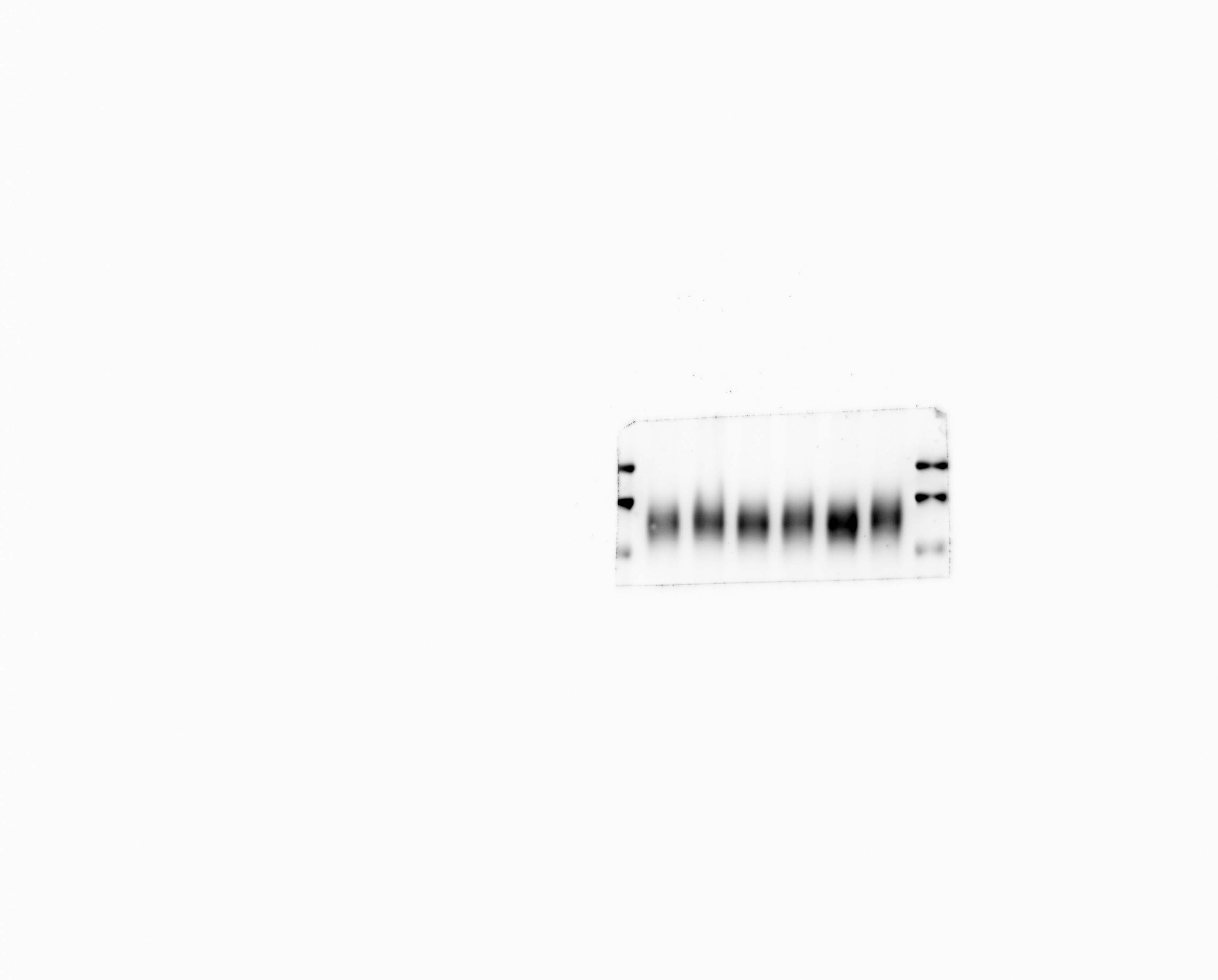

Supplement: Supplementary file 2 — Supporting File 2: advs73976‐sup‐0002‐SuppMat.zip. [file ADVS-13-e11217-s002.zip › WB#U4ee3#U8868#U56fe/xiap#U539f#U59cb#U6570#U636ewb1-JPEG/LAMP1_9.jpg]

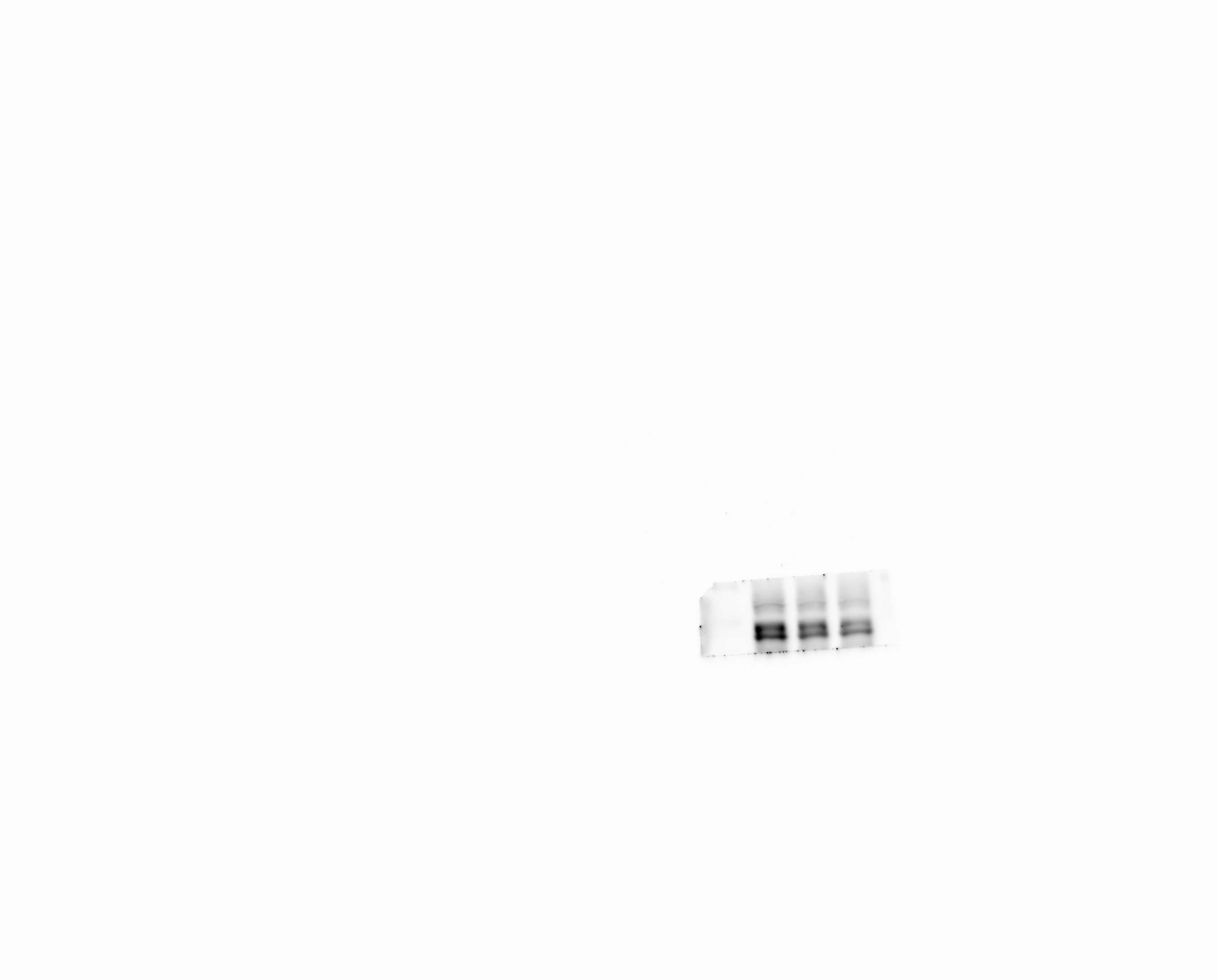

Supplement: Supplementary file 2 — Supporting File 2: advs73976‐sup‐0002‐SuppMat.zip. [file ADVS-13-e11217-s002.zip › WB#U4ee3#U8868#U56fe/xiap#U539f#U59cb#U6570#U636ewb1-JPEG/lamp2_9 six.jpg]

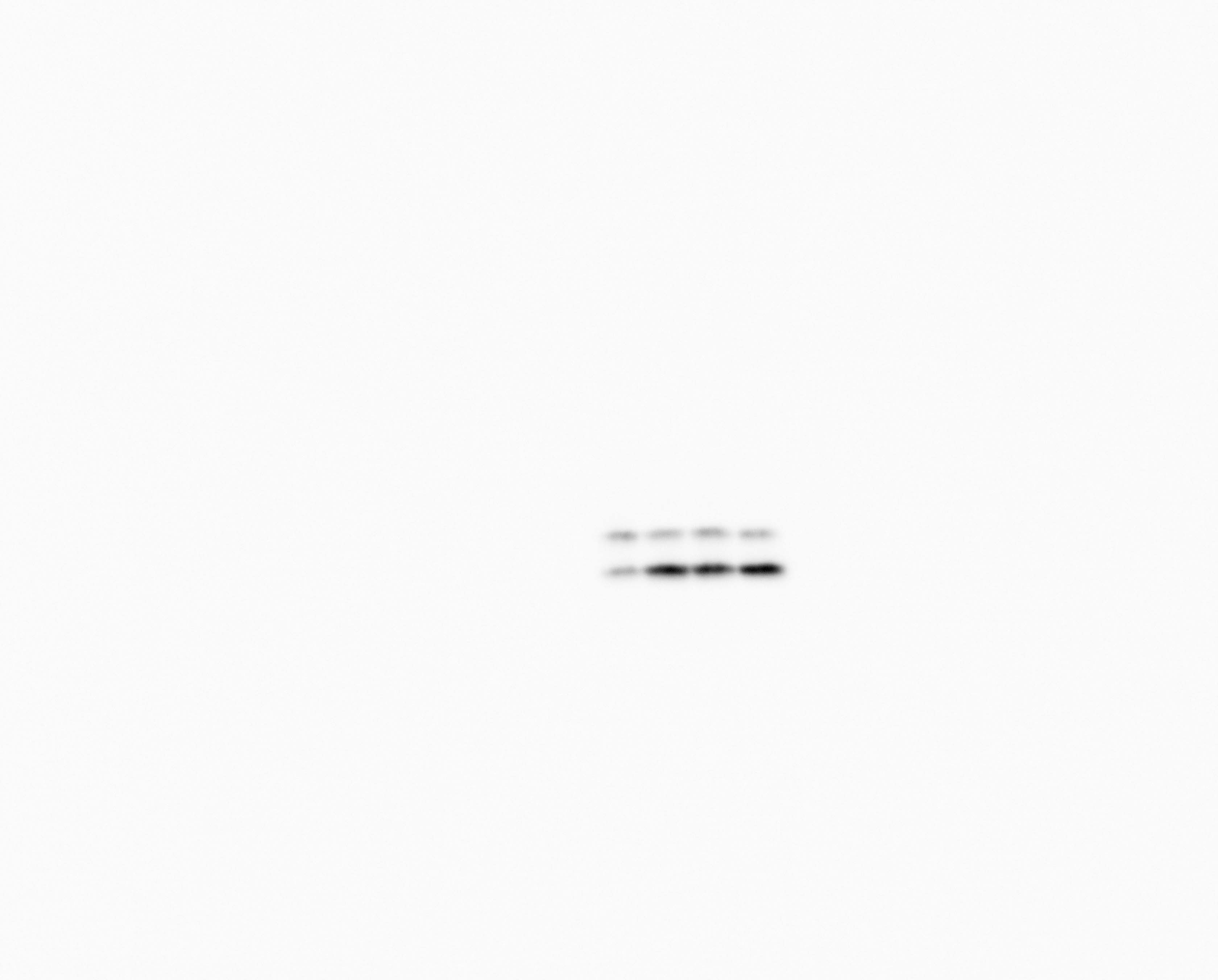

Supplement: Supplementary file 2 — Supporting File 2: advs73976‐sup‐0002‐SuppMat.zip. [file ADVS-13-e11217-s002.zip › WB#U4ee3#U8868#U56fe/xiap#U539f#U59cb#U6570#U636ewb1-JPEG/LC3-1200MS_1#U4ee3#U8868.jpg]

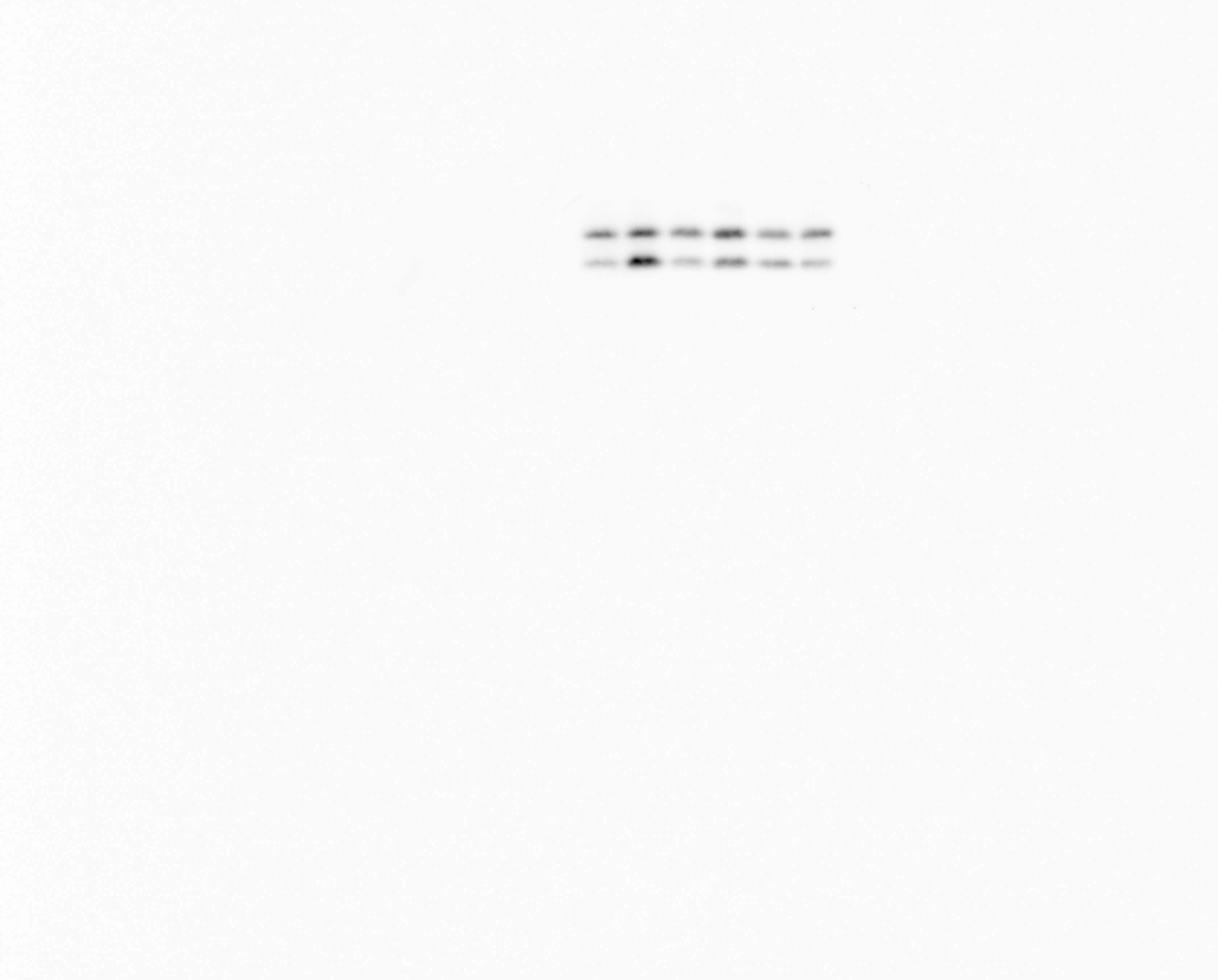

Supplement: Supplementary file 2 — Supporting File 2: advs73976‐sup‐0002‐SuppMat.zip. [file ADVS-13-e11217-s002.zip › WB#U4ee3#U8868#U56fe/xiap#U539f#U59cb#U6570#U636ewb1-JPEG/LC3-_6#U4ee3#U8868.jpg]

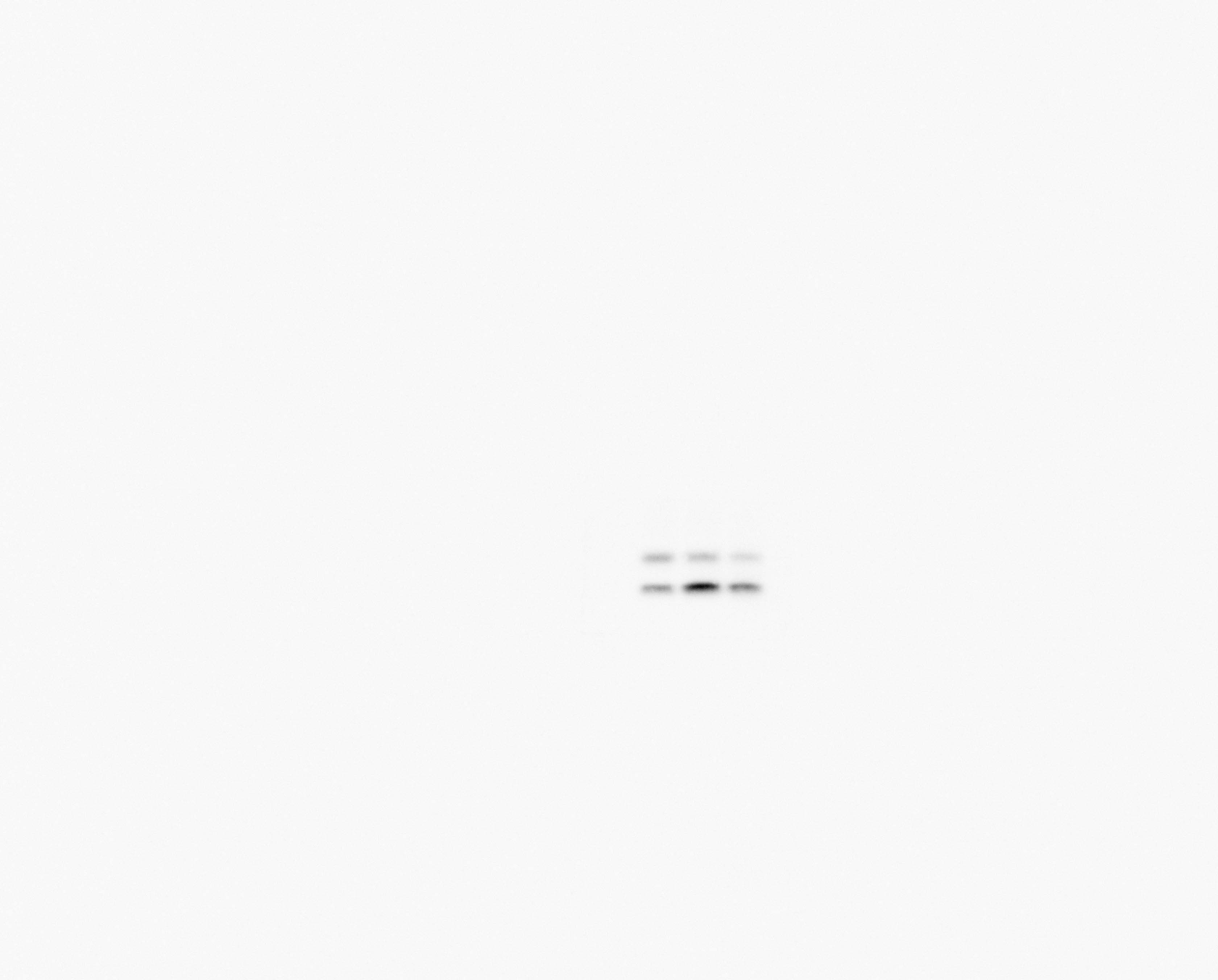

Supplement: Supplementary file 2 — Supporting File 2: advs73976‐sup‐0002‐SuppMat.zip. [file ADVS-13-e11217-s002.zip › WB#U4ee3#U8868#U56fe/xiap#U539f#U59cb#U6570#U636ewb1-JPEG/LC3_1 six.jpg]

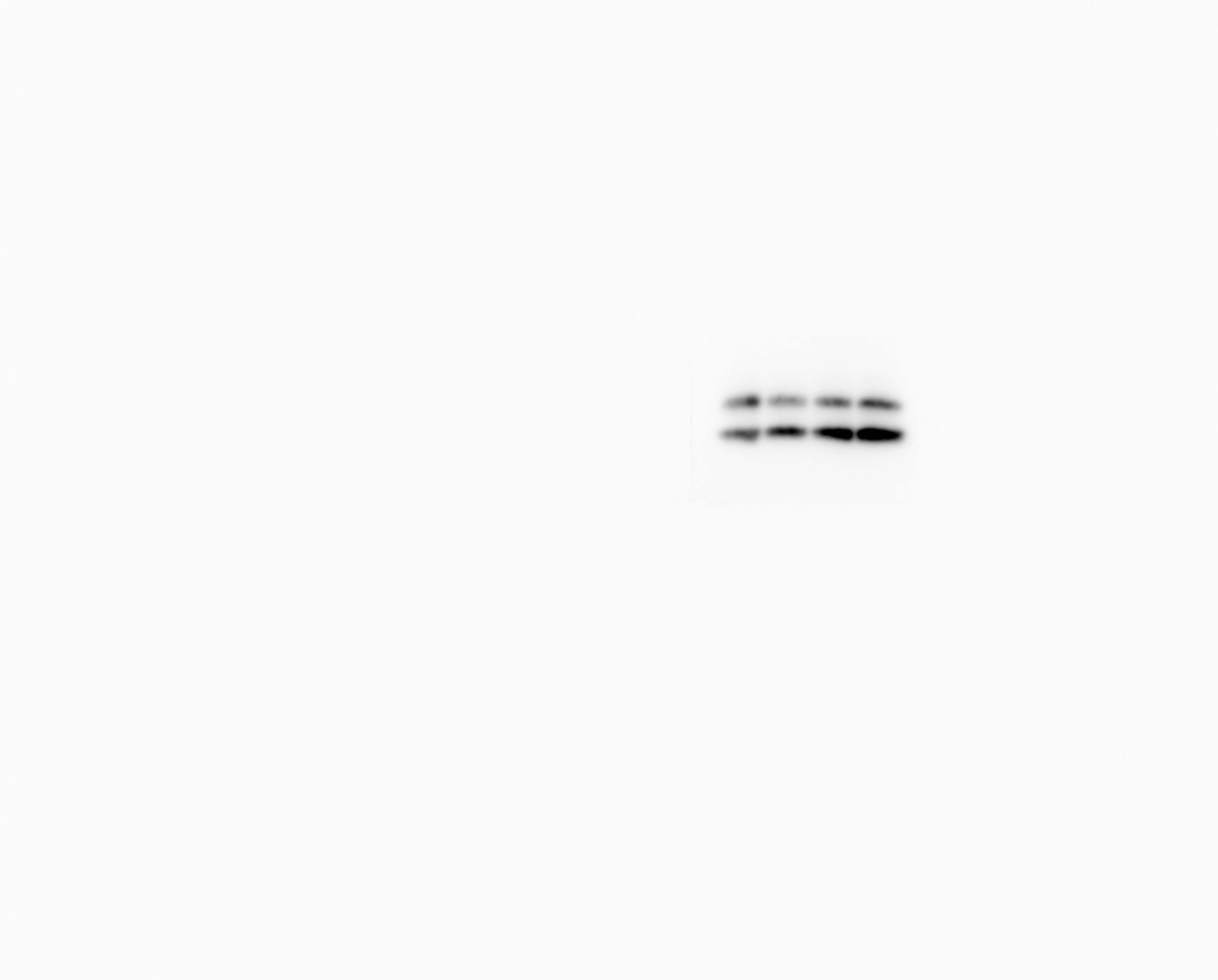

Supplement: Supplementary file 2 — Supporting File 2: advs73976‐sup‐0002‐SuppMat.zip. [file ADVS-13-e11217-s002.zip › WB#U4ee3#U8868#U56fe/xiap#U539f#U59cb#U6570#U636ewb1-JPEG/LC3_10 #U4ee3#U8868 gas.jpg]

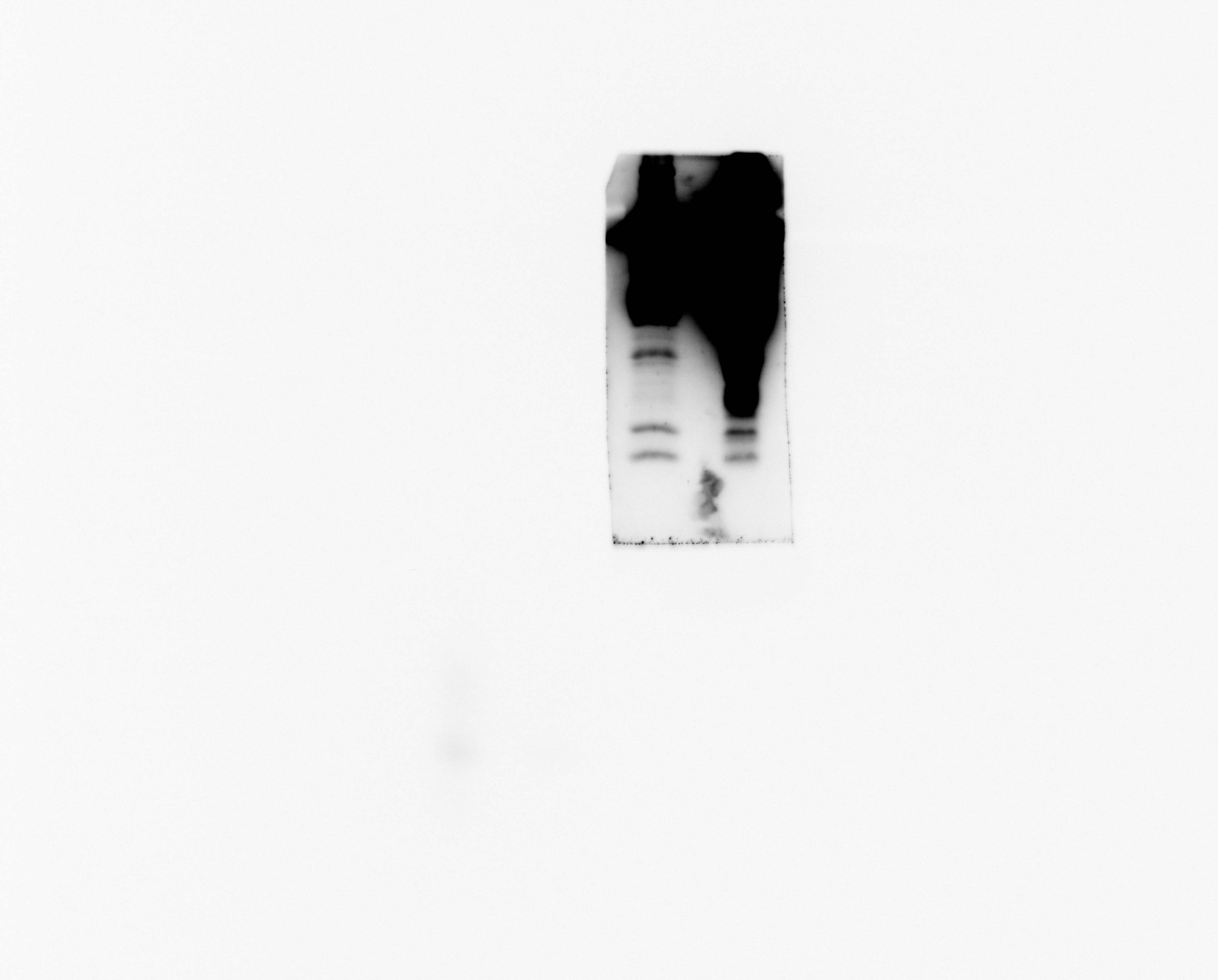

Supplement: Supplementary file 2 — Supporting File 2: advs73976‐sup‐0002‐SuppMat.zip. [file ADVS-13-e11217-s002.zip › WB#U4ee3#U8868#U56fe/xiap#U539f#U59cb#U6570#U636ewb1-JPEG/LC3_20 ip ha.jpg]

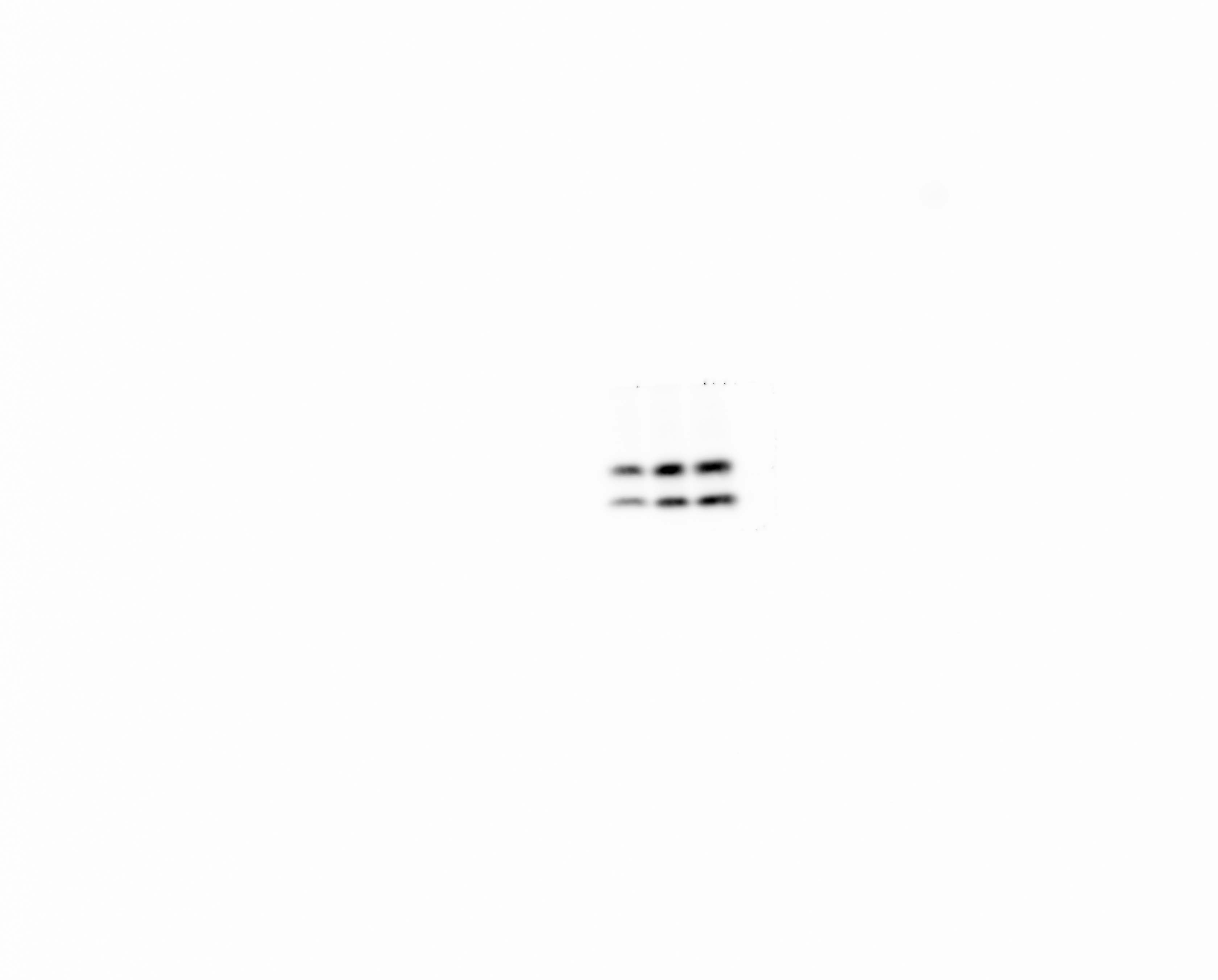

Supplement: Supplementary file 2 — Supporting File 2: advs73976‐sup‐0002‐SuppMat.zip. [file ADVS-13-e11217-s002.zip › WB#U4ee3#U8868#U56fe/xiap#U539f#U59cb#U6570#U636ewb1-JPEG/LC3_7#U4ee3#U8868 oex.jpg]

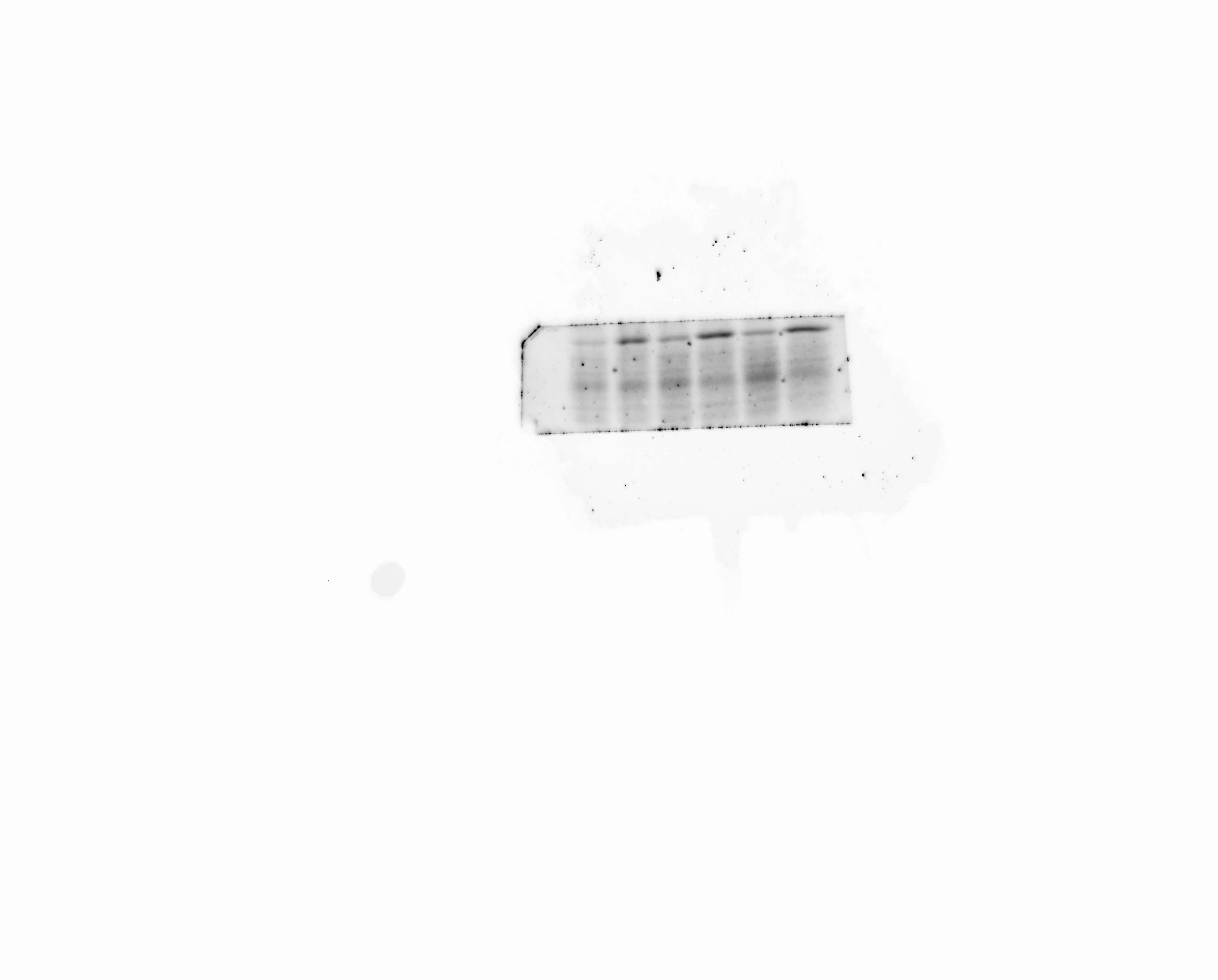

Supplement: Supplementary file 2 — Supporting File 2: advs73976‐sup‐0002‐SuppMat.zip. [file ADVS-13-e11217-s002.zip › WB#U4ee3#U8868#U56fe/xiap#U539f#U59cb#U6570#U636ewb1-JPEG/p-eif2a_8.jpg]

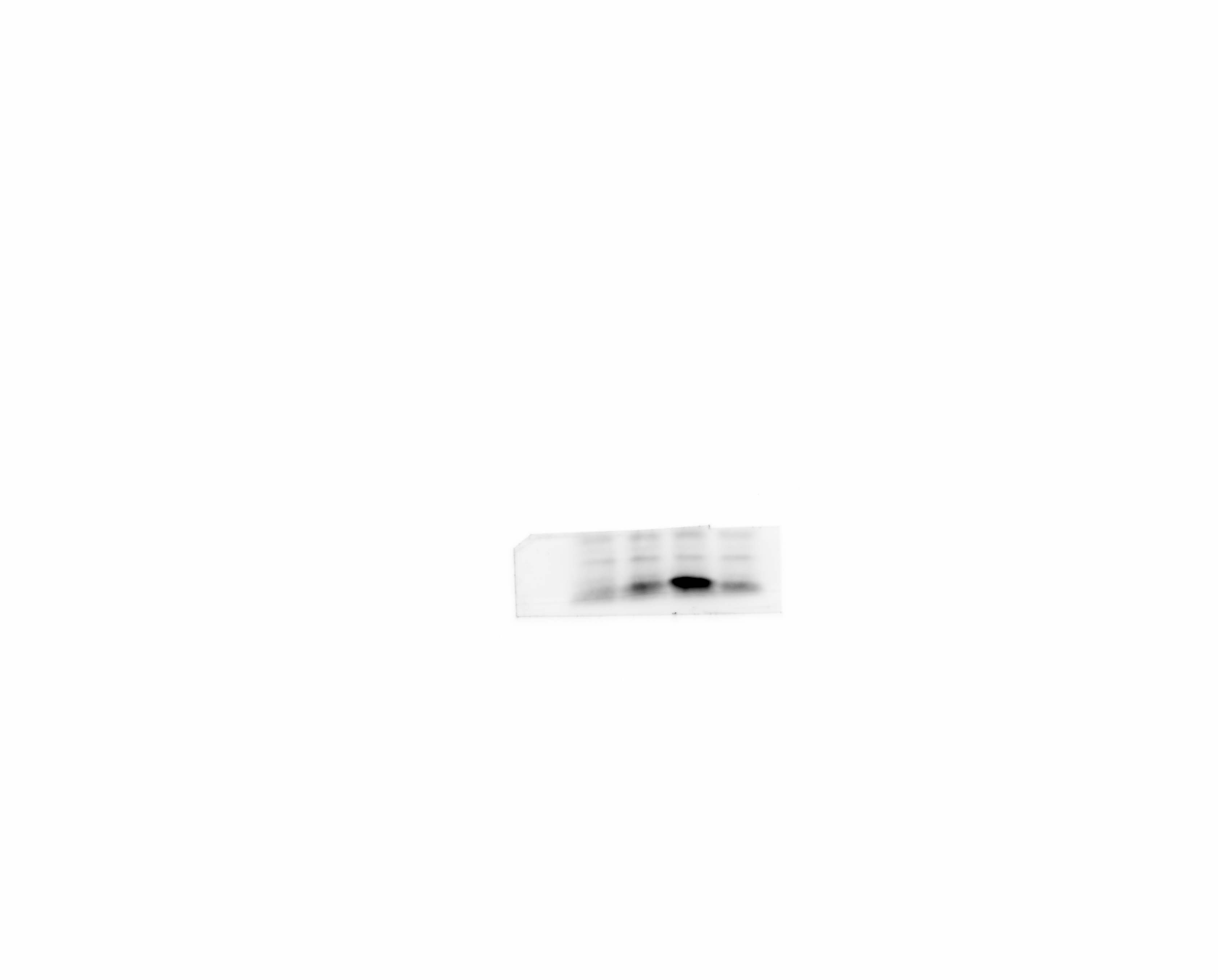

Supplement: Supplementary file 2 — Supporting File 2: advs73976‐sup‐0002‐SuppMat.zip. [file ADVS-13-e11217-s002.zip › WB#U4ee3#U8868#U56fe/xiap#U539f#U59cb#U6570#U636ewb1-JPEG/P21_2#U4ee3#U8868.jpg]

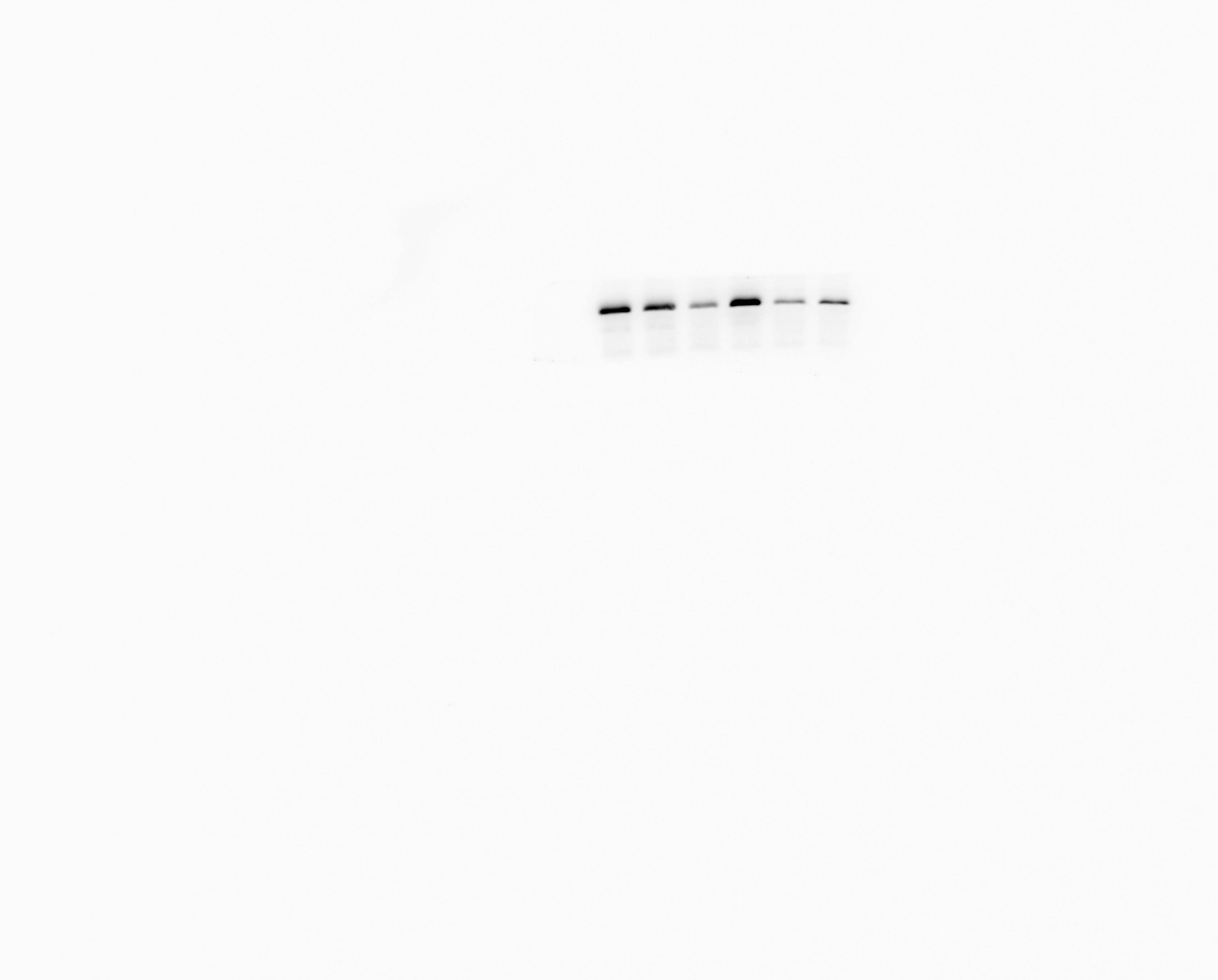

Supplement: Supplementary file 2 — Supporting File 2: advs73976‐sup‐0002‐SuppMat.zip. [file ADVS-13-e11217-s002.zip › WB#U4ee3#U8868#U56fe/xiap#U539f#U59cb#U6570#U636ewb1-JPEG/P62-_9#U4ee3#U8868.jpg]

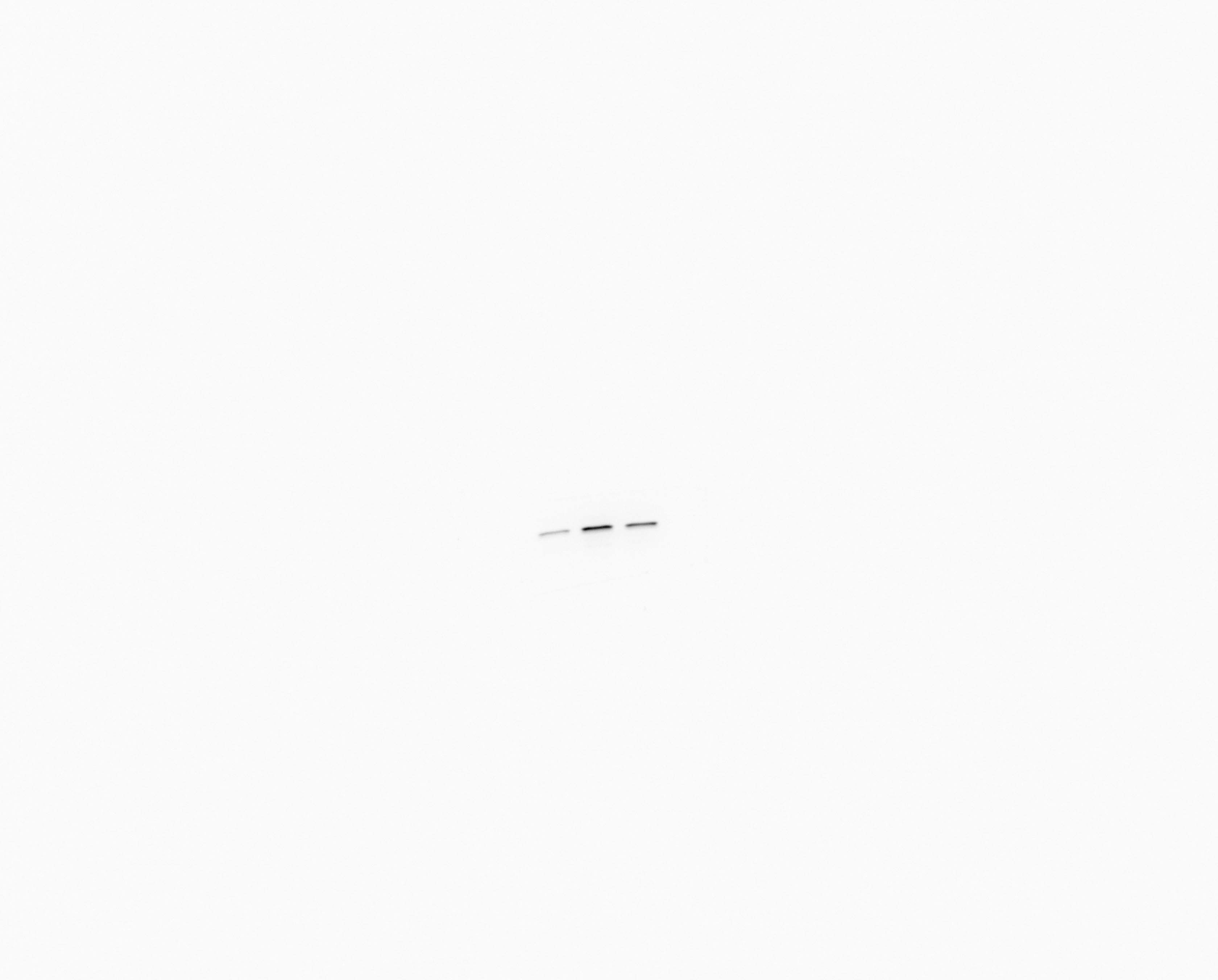

Supplement: Supplementary file 2 — Supporting File 2: advs73976‐sup‐0002‐SuppMat.zip. [file ADVS-13-e11217-s002.zip › WB#U4ee3#U8868#U56fe/xiap#U539f#U59cb#U6570#U636ewb1-JPEG/p62_1#U4ee3#U8868 oex.jpg]

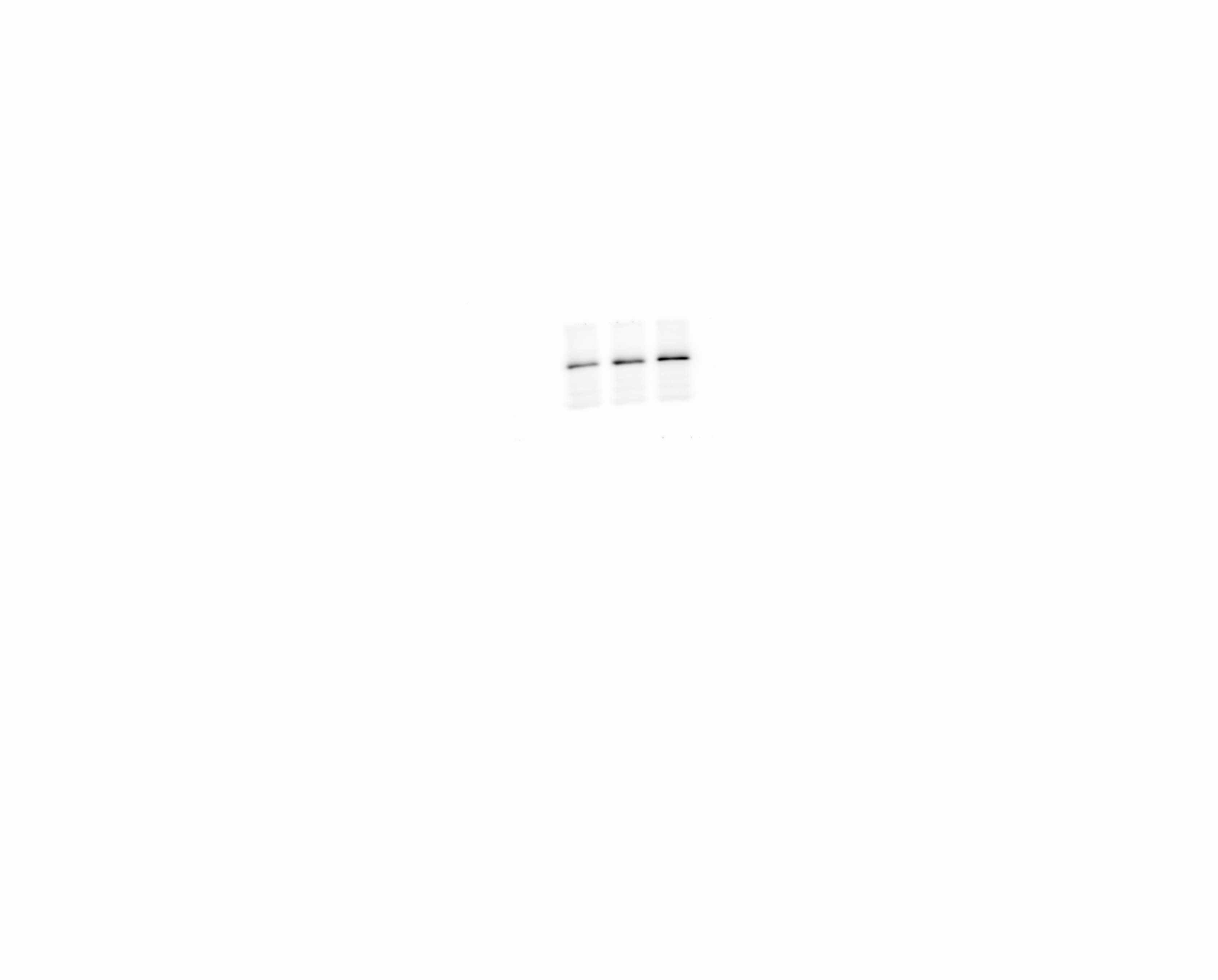

Supplement: Supplementary file 2 — Supporting File 2: advs73976‐sup‐0002‐SuppMat.zip. [file ADVS-13-e11217-s002.zip › WB#U4ee3#U8868#U56fe/xiap#U539f#U59cb#U6570#U636ewb1-JPEG/P62_5#U4ee3#U8868 six.jpg]

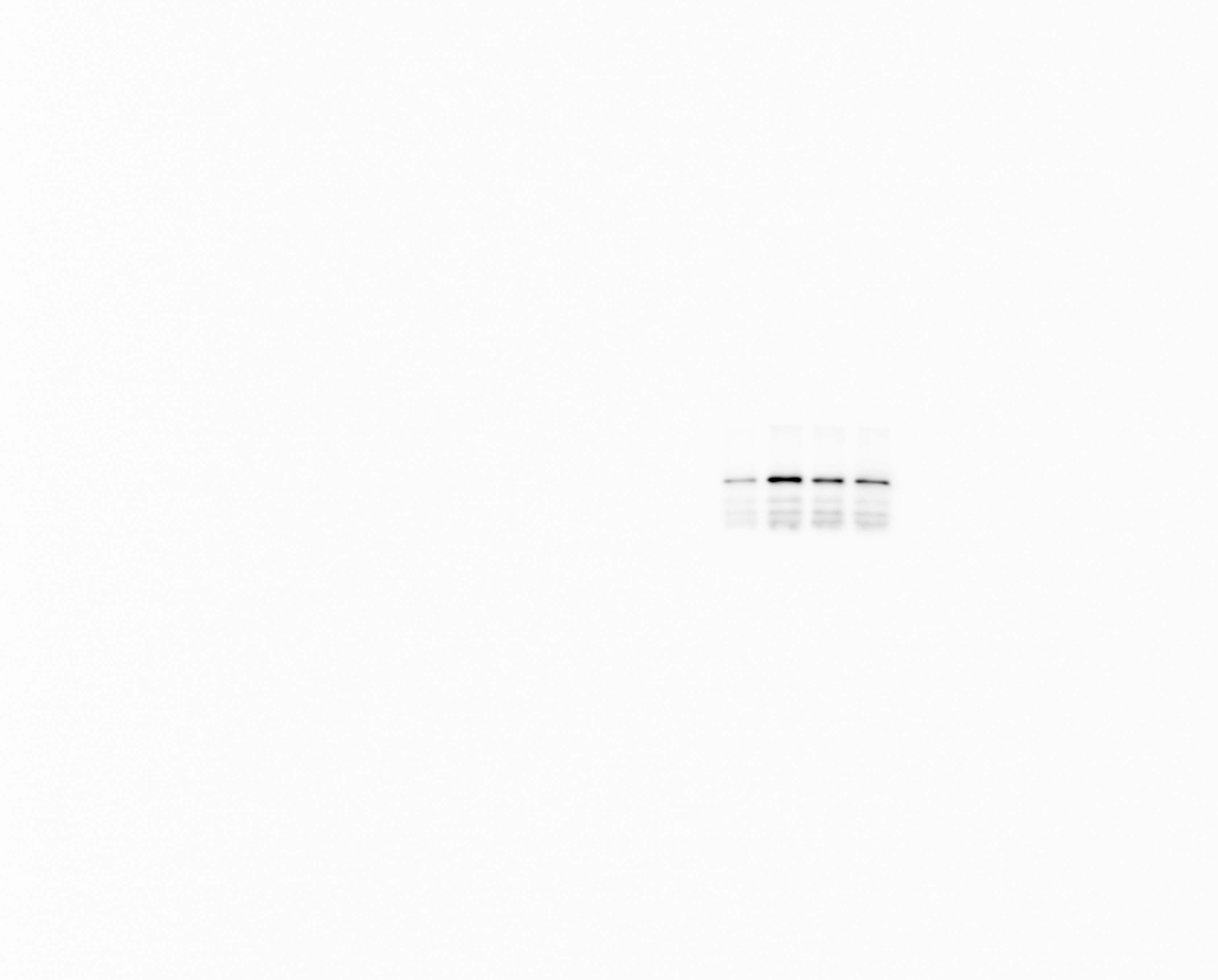

Supplement: Supplementary file 2 — Supporting File 2: advs73976‐sup‐0002‐SuppMat.zip. [file ADVS-13-e11217-s002.zip › WB#U4ee3#U8868#U56fe/xiap#U539f#U59cb#U6570#U636ewb1-JPEG/P62_7 gas.jpg]

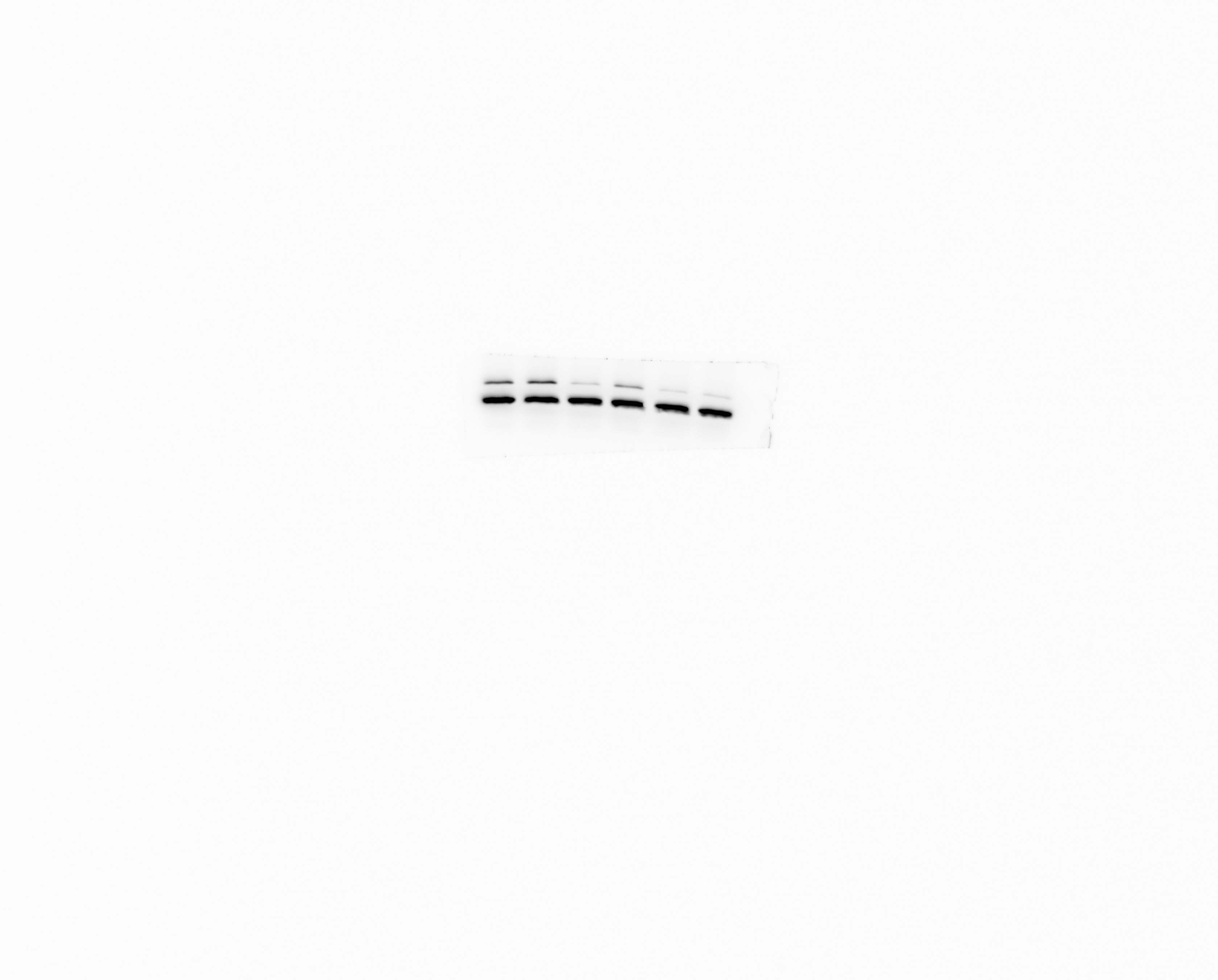

Supplement: Supplementary file 2 — Supporting File 2: advs73976‐sup‐0002‐SuppMat.zip. [file ADVS-13-e11217-s002.zip › WB#U4ee3#U8868#U56fe/xiap#U539f#U59cb#U6570#U636ewb1-JPEG/TUBULIN_5#U4ee3#U8868 lc3.jpg]

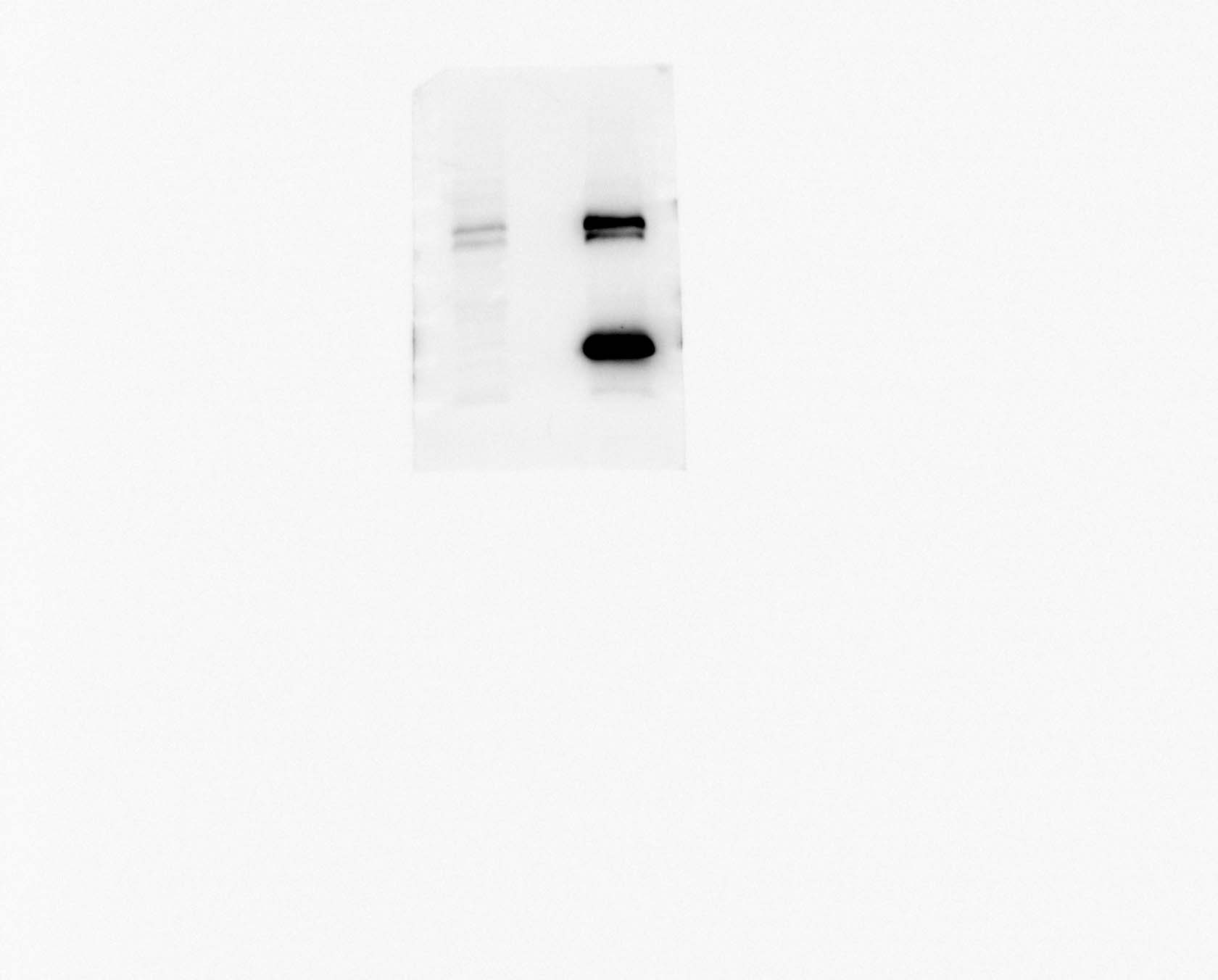

Supplement: Supplementary file 2 — Supporting File 2: advs73976‐sup‐0002‐SuppMat.zip. [file ADVS-13-e11217-s002.zip › WB#U4ee3#U8868#U56fe/xiap#U539f#U59cb#U6570#U636ewb1-JPEG/XIAP 10S_15 #U4ee3#U8868 ip lc3 23-4-20.jpg]

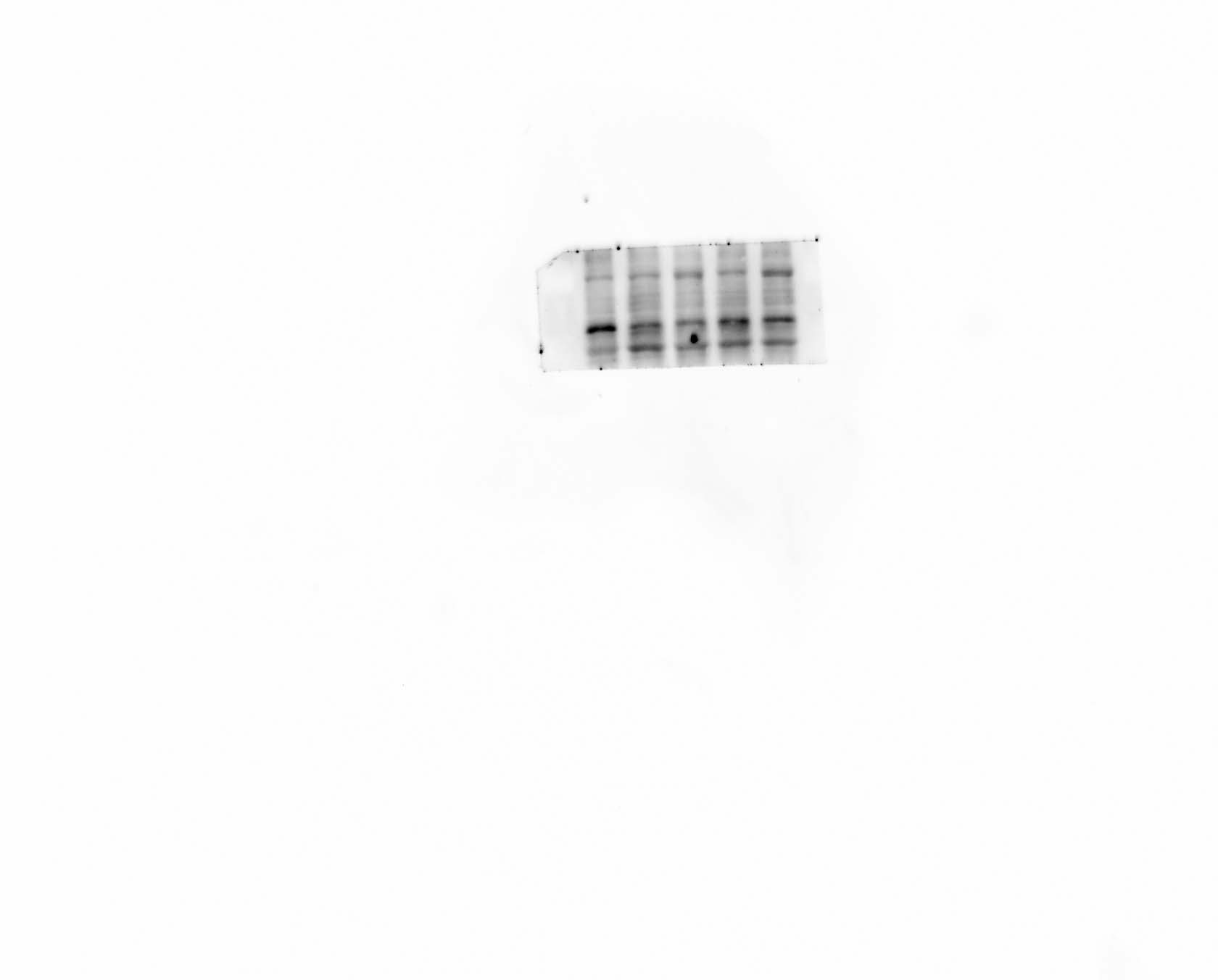

Supplement: Supplementary file 2 — Supporting File 2: advs73976‐sup‐0002‐SuppMat.zip. [file ADVS-13-e11217-s002.zip › WB#U4ee3#U8868#U56fe/xiap#U539f#U59cb#U6570#U636ewb1-JPEG/xiap-1_4 #U4ee3#U8868 mg132.jpg]

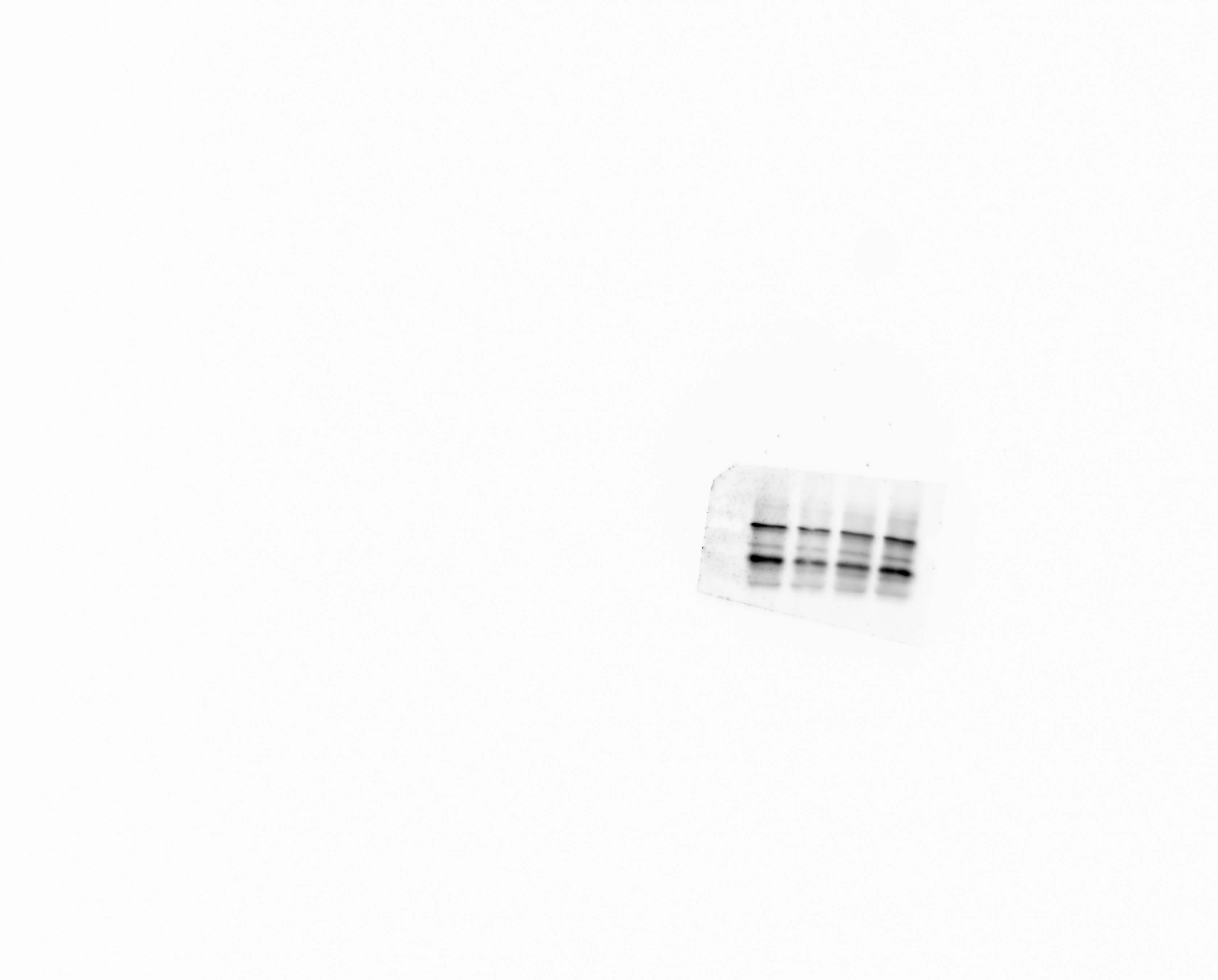

Supplement: Supplementary file 2 — Supporting File 2: advs73976‐sup‐0002‐SuppMat.zip. [file ADVS-13-e11217-s002.zip › WB#U4ee3#U8868#U56fe/xiap#U539f#U59cb#U6570#U636ewb1-JPEG/xiap-_6 shatf4 eif.jpg]

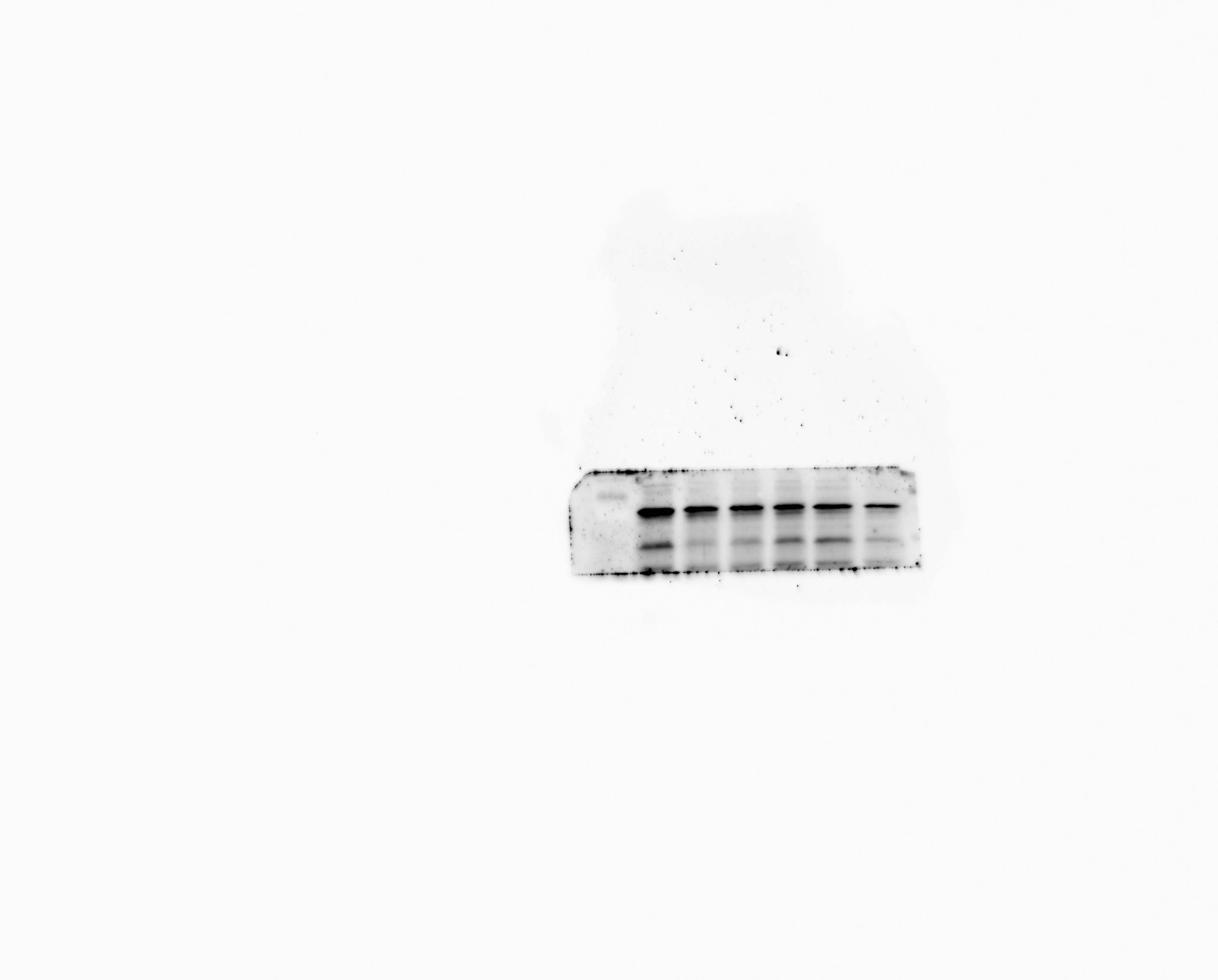

Supplement: Supplementary file 2 — Supporting File 2: advs73976‐sup‐0002‐SuppMat.zip. [file ADVS-13-e11217-s002.zip › WB#U4ee3#U8868#U56fe/xiap#U539f#U59cb#U6570#U636ewb1-JPEG/xiap1_10 gas.jpg]

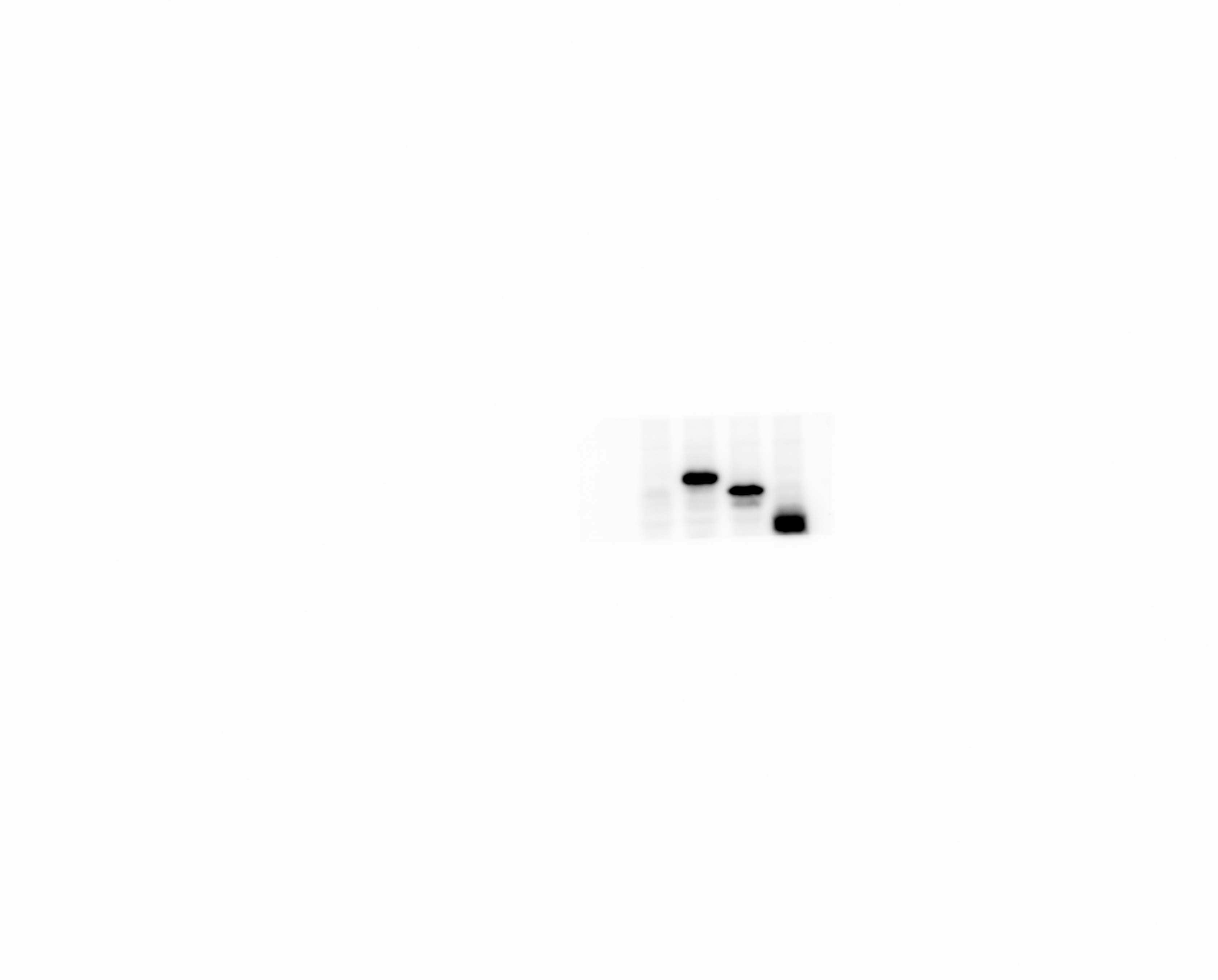

Supplement: Supplementary file 2 — Supporting File 2: advs73976‐sup‐0002‐SuppMat.zip. [file ADVS-13-e11217-s002.zip › WB#U4ee3#U8868#U56fe/xiap#U539f#U59cb#U6570#U636ewb1-JPEG/xiap_10 m12.jpg]

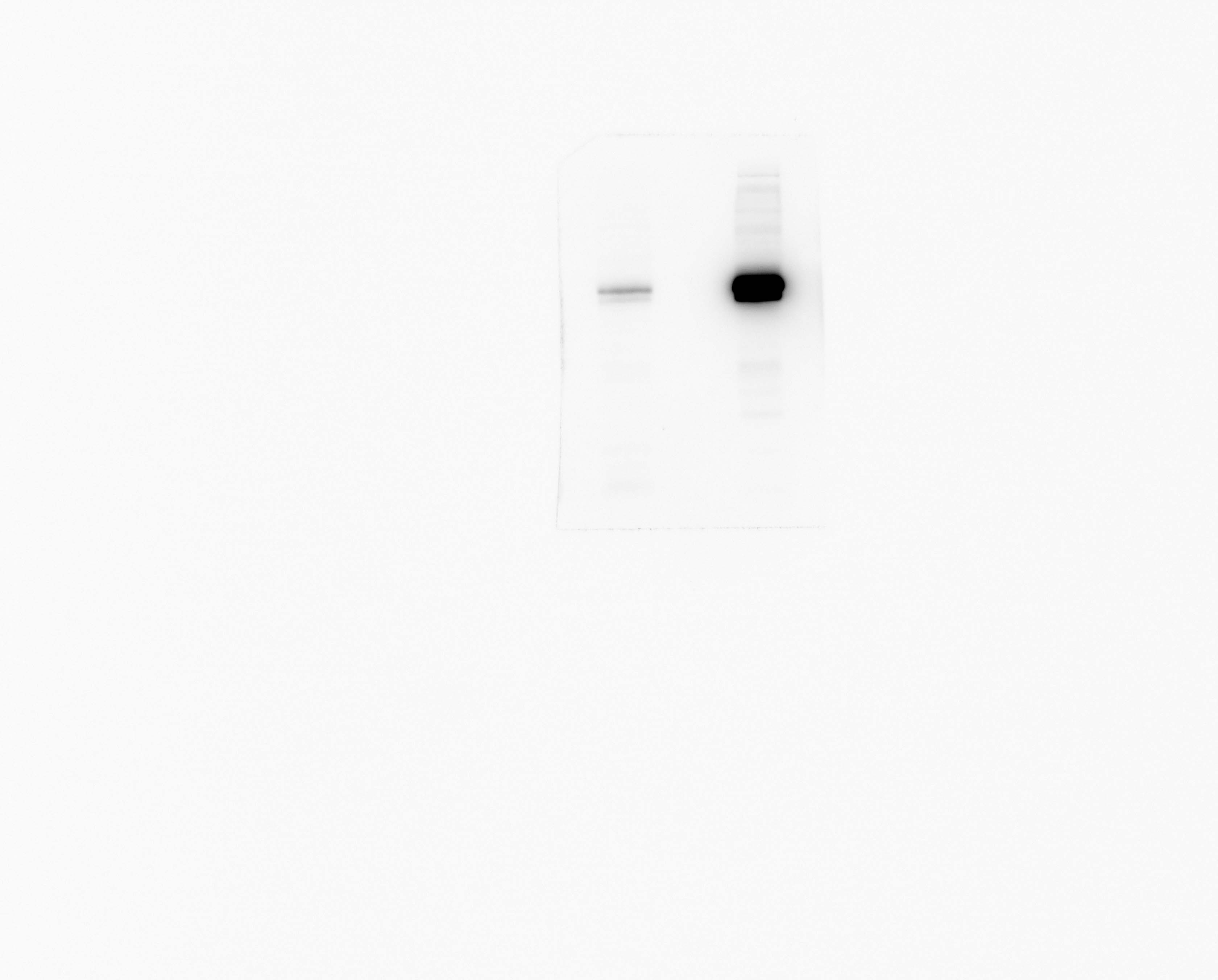

Supplement: Supplementary file 2 — Supporting File 2: advs73976‐sup‐0002‐SuppMat.zip. [file ADVS-13-e11217-s002.zip › WB#U4ee3#U8868#U56fe/xiap#U539f#U59cb#U6570#U636ewb1-JPEG/xiap_15 HA.jpg]

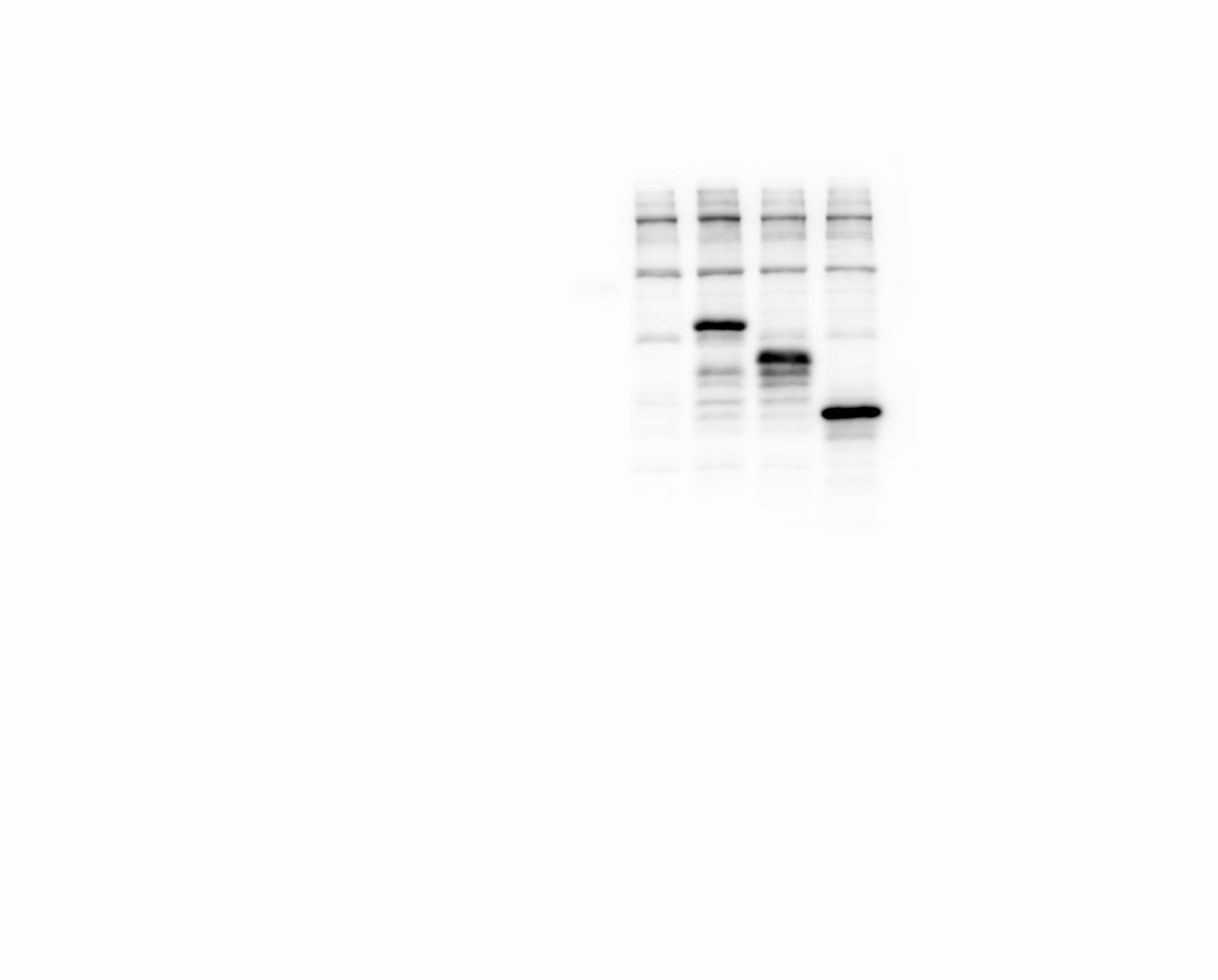

Supplement: Supplementary file 2 — Supporting File 2: advs73976‐sup‐0002‐SuppMat.zip. [file ADVS-13-e11217-s002.zip › WB#U4ee3#U8868#U56fe/xiap#U539f#U59cb#U6570#U636ewb1-JPEG/XIAP_4 db m34.jpg]

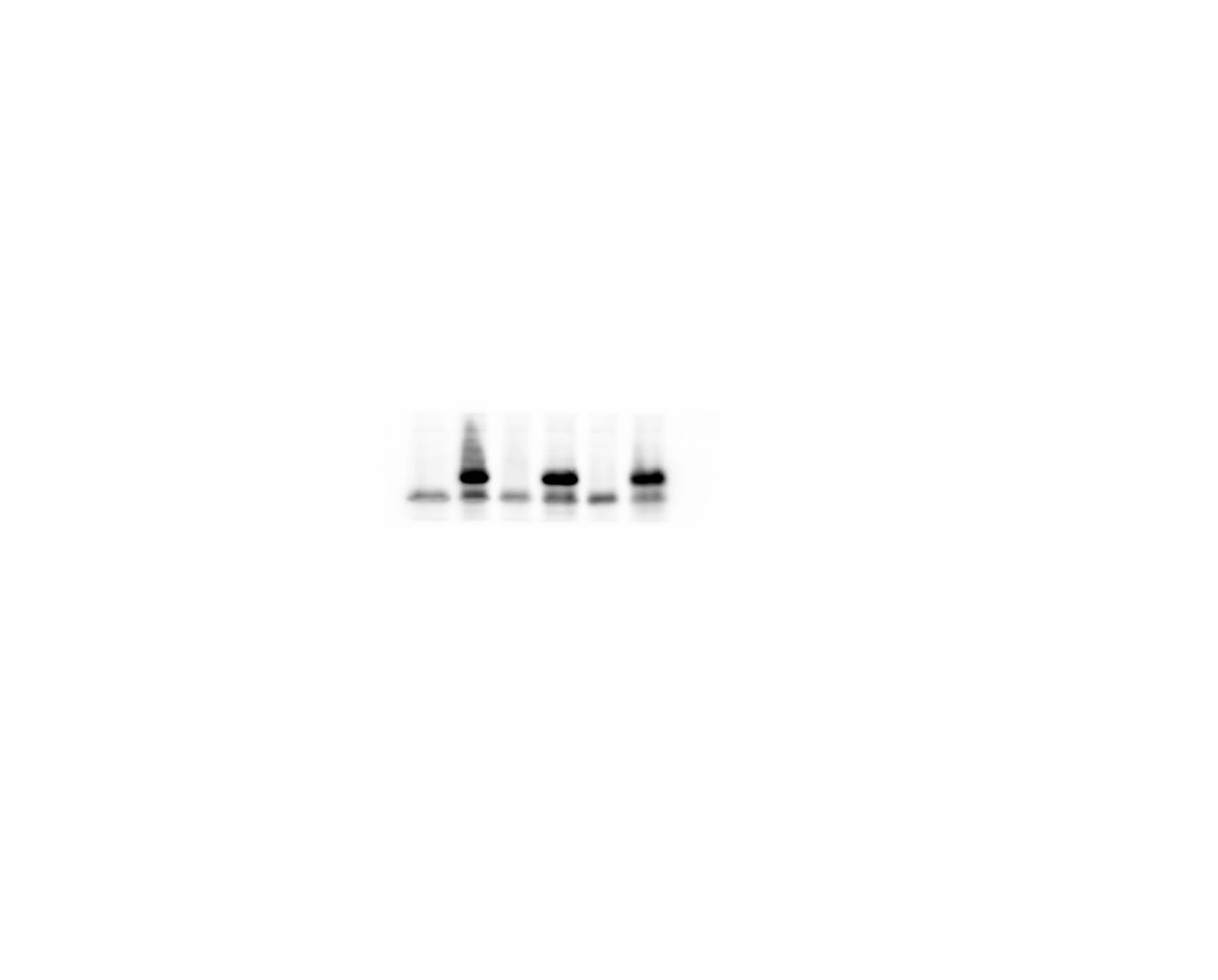

Supplement: Supplementary file 2 — Supporting File 2: advs73976‐sup‐0002‐SuppMat.zip. [file ADVS-13-e11217-s002.zip › WB#U4ee3#U8868#U56fe/xiap#U539f#U59cb#U6570#U636ewb1-JPEG/XIAP_4#U4ee3#U8868.jpg]

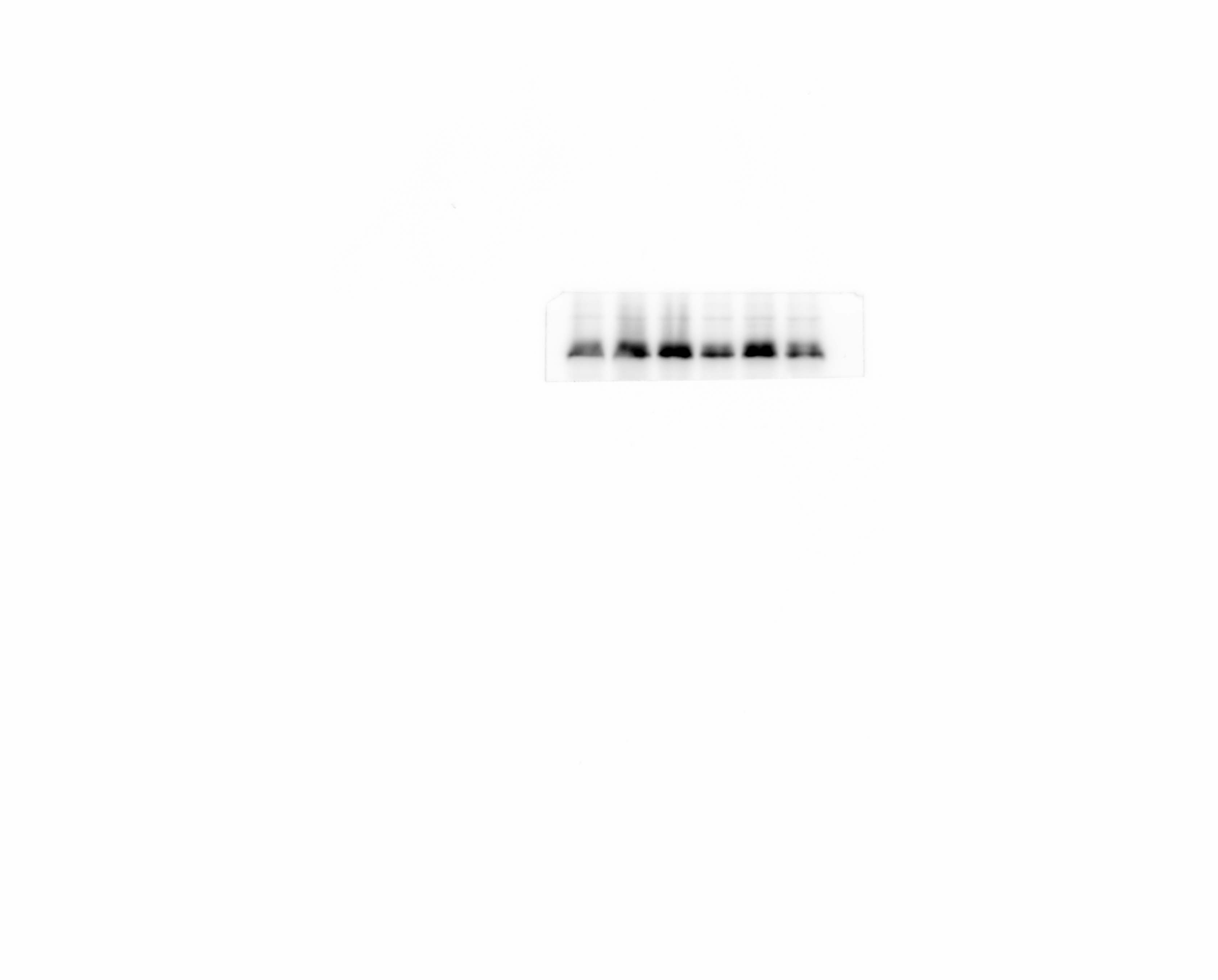

Supplement: Supplementary file 2 — Supporting File 2: advs73976‐sup‐0002‐SuppMat.zip. [file ADVS-13-e11217-s002.zip › WB#U4ee3#U8868#U56fe/xiap#U539f#U59cb#U6570#U636ewb1-JPEG/XIAP_5-#U4ee3#U8868.jpg]

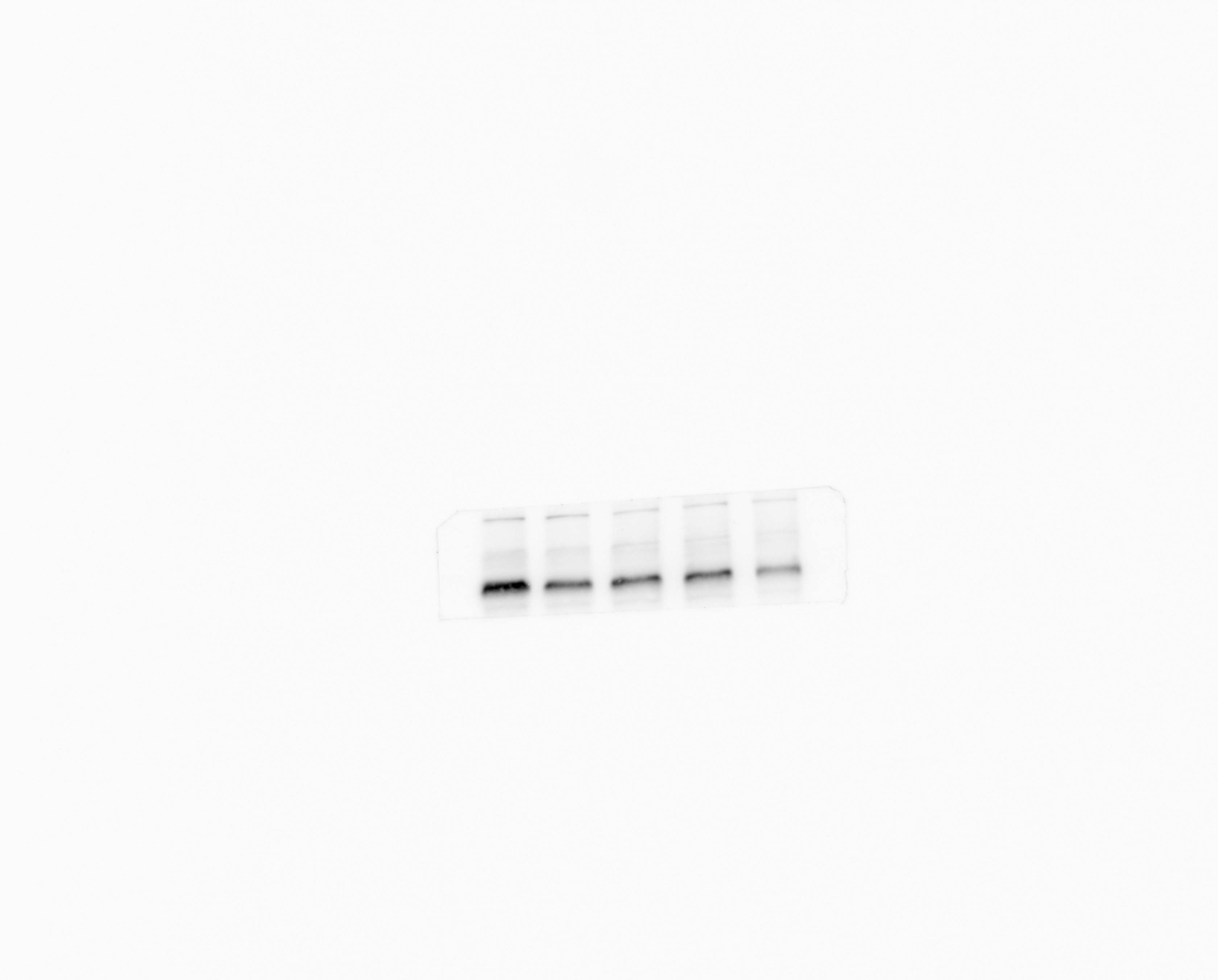

Supplement: Supplementary file 2 — Supporting File 2: advs73976‐sup‐0002‐SuppMat.zip. [file ADVS-13-e11217-s002.zip › WB#U4ee3#U8868#U56fe/xiap#U539f#U59cb#U6570#U636ewb1-JPEG/xiap_new_9 db si.jpg]

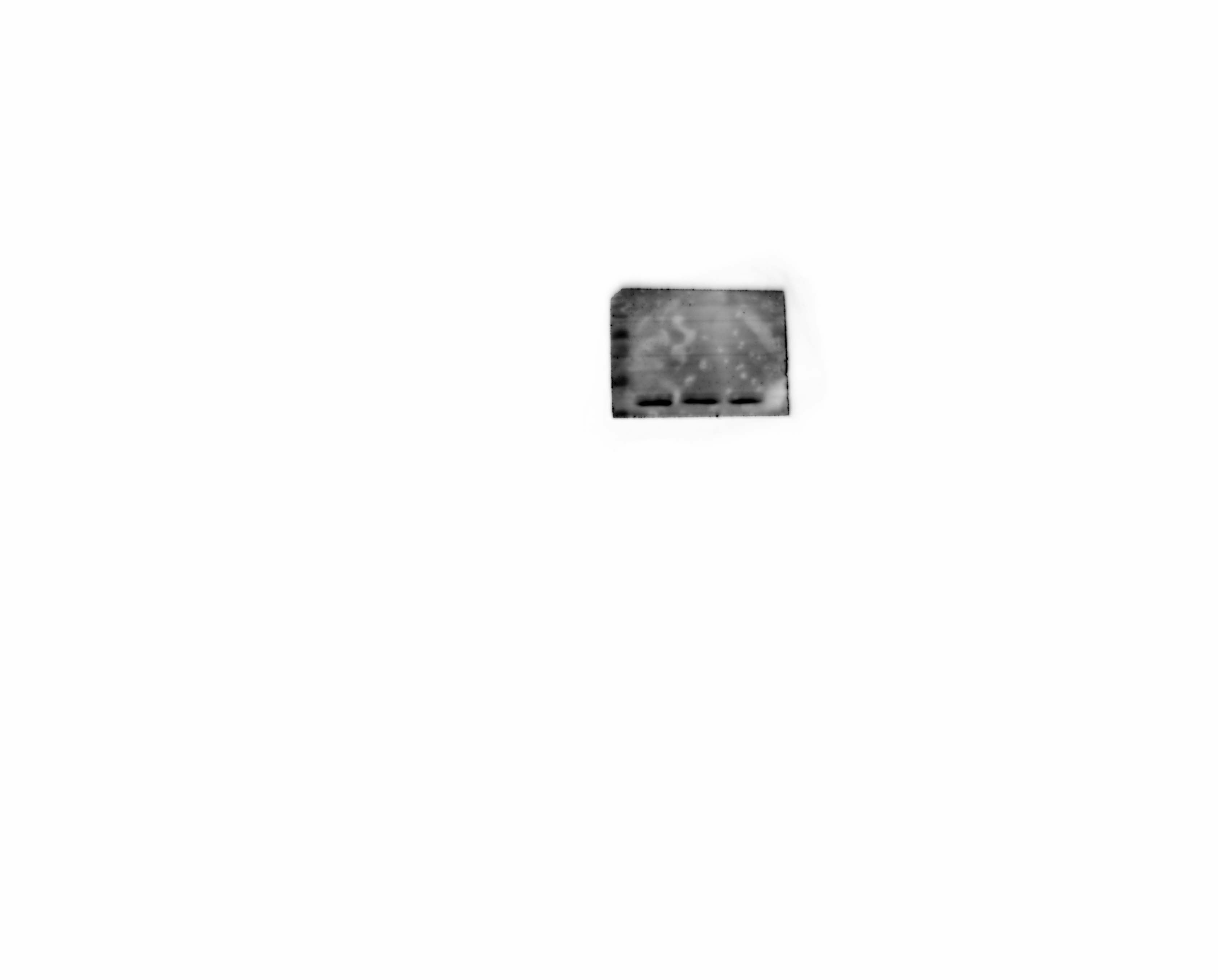

Supplement: Supplementary file 2 — Supporting File 2: advs73976‐sup‐0002‐SuppMat.zip. [file ADVS-13-e11217-s002.zip › WB#U4ee3#U8868#U56fe/xiap#U539f#U59cb#U6570#U636ewb2-JPEG/ACTB1_7-ctsb.jpg]

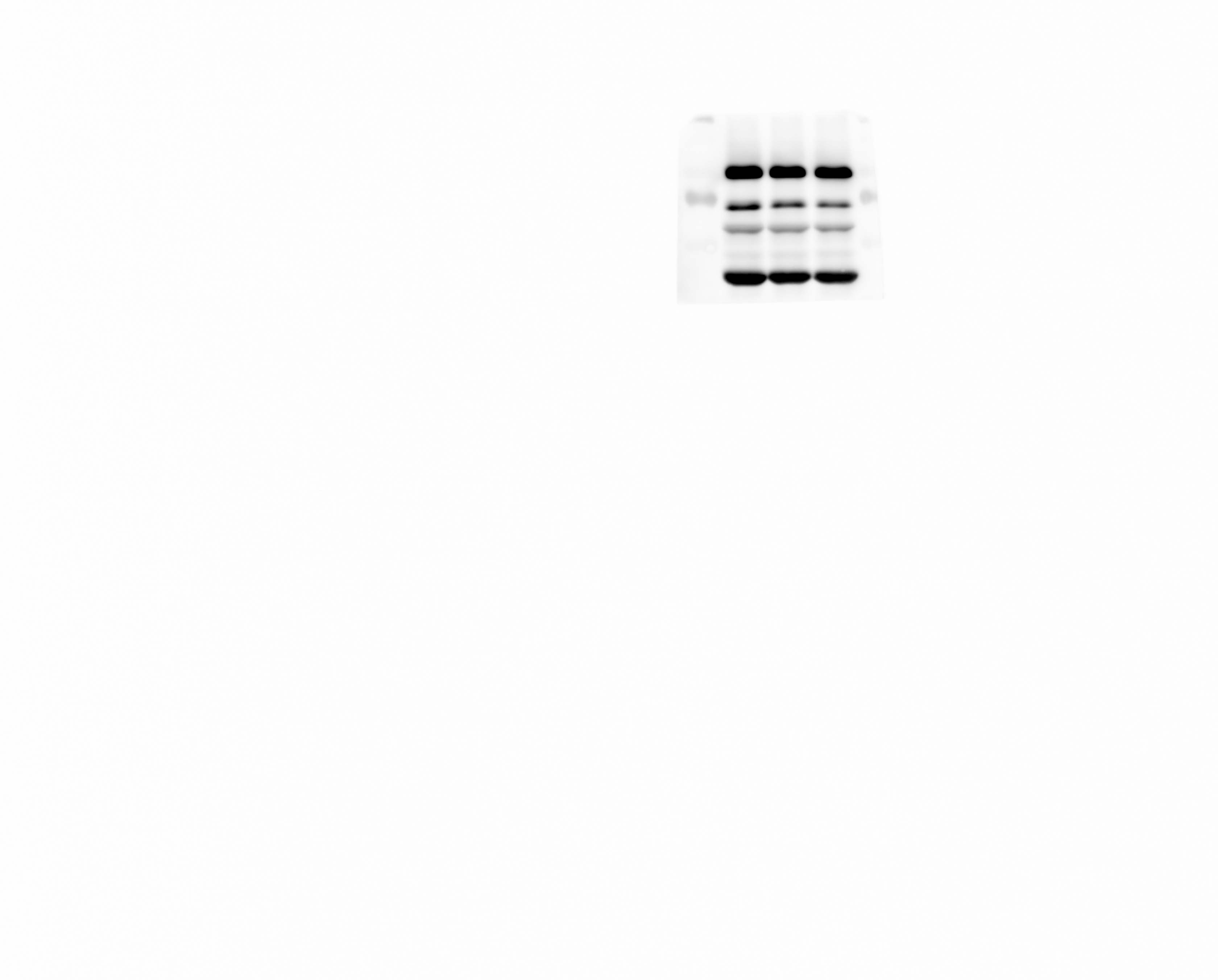

Supplement: Supplementary file 2 — Supporting File 2: advs73976‐sup‐0002‐SuppMat.zip. [file ADVS-13-e11217-s002.zip › WB#U4ee3#U8868#U56fe/xiap#U539f#U59cb#U6570#U636ewb2-JPEG/ACTB_6 chop six.jpg]

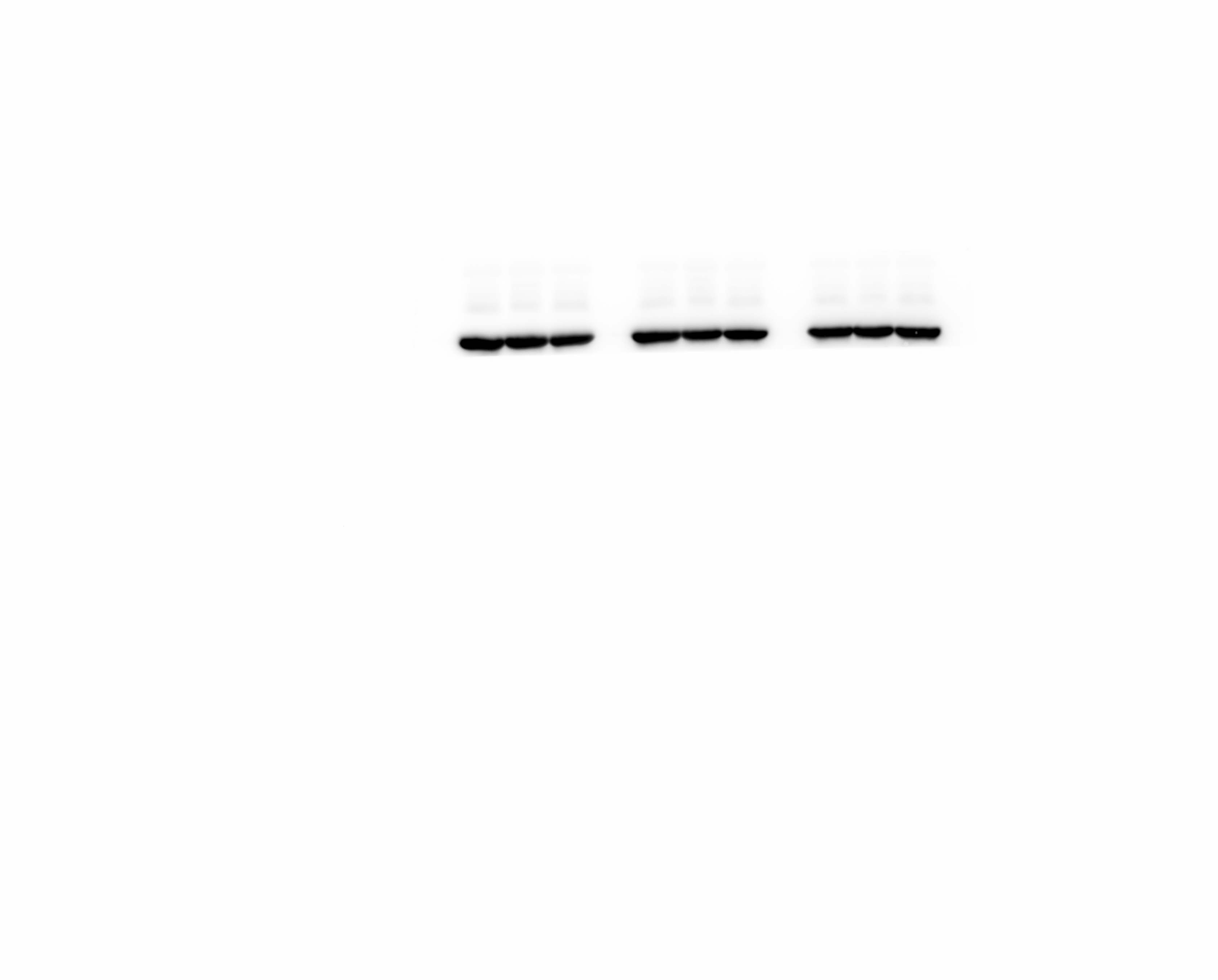

Supplement: Supplementary file 2 — Supporting File 2: advs73976‐sup‐0002‐SuppMat.zip. [file ADVS-13-e11217-s002.zip › WB#U4ee3#U8868#U56fe/xiap#U539f#U59cb#U6570#U636ewb2-JPEG/ACTB_7-p62.jpg]

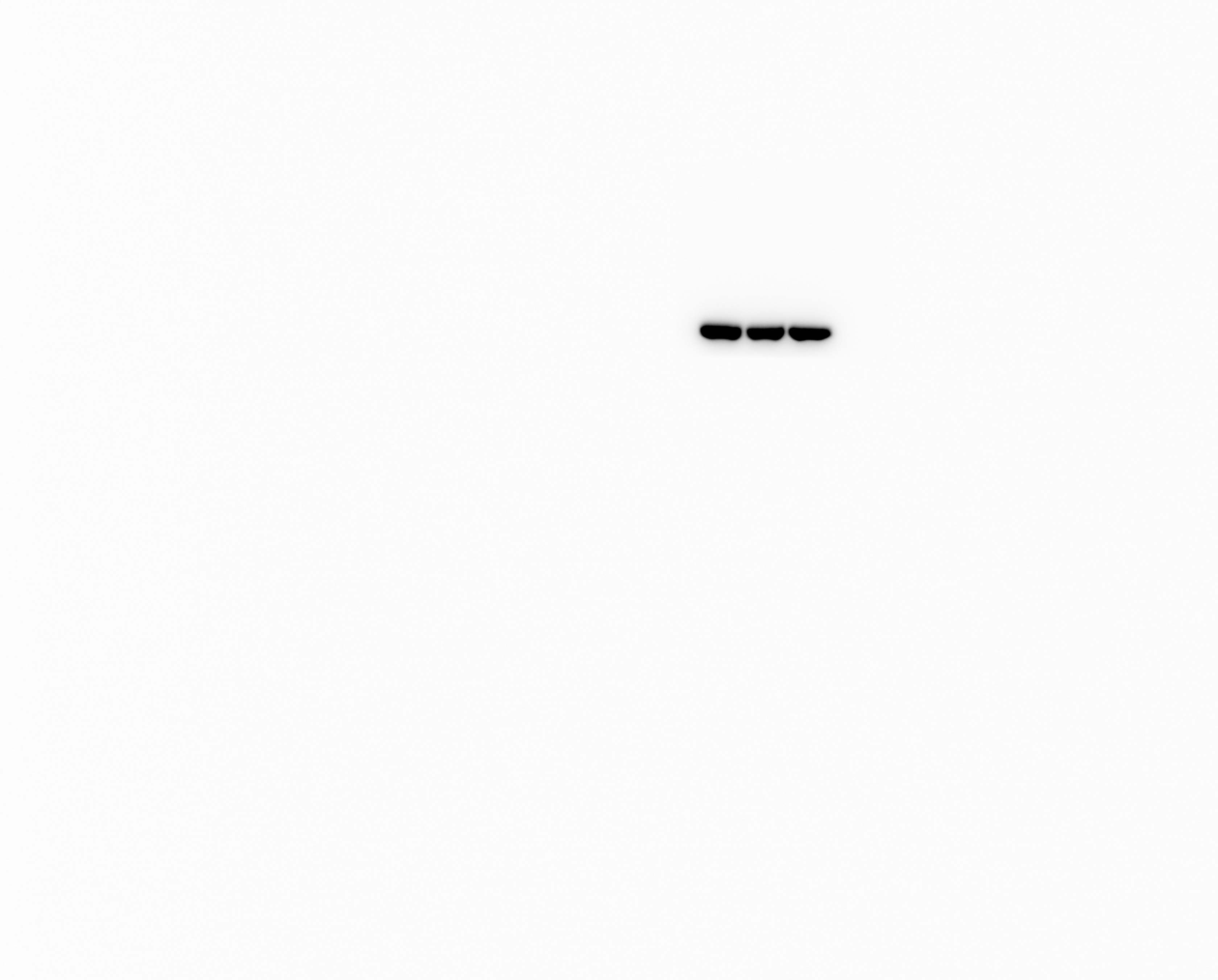

Supplement: Supplementary file 2 — Supporting File 2: advs73976‐sup‐0002‐SuppMat.zip. [file ADVS-13-e11217-s002.zip › WB#U4ee3#U8868#U56fe/xiap#U539f#U59cb#U6570#U636ewb2-JPEG/actin_10 -ctsb oedx.jpg]

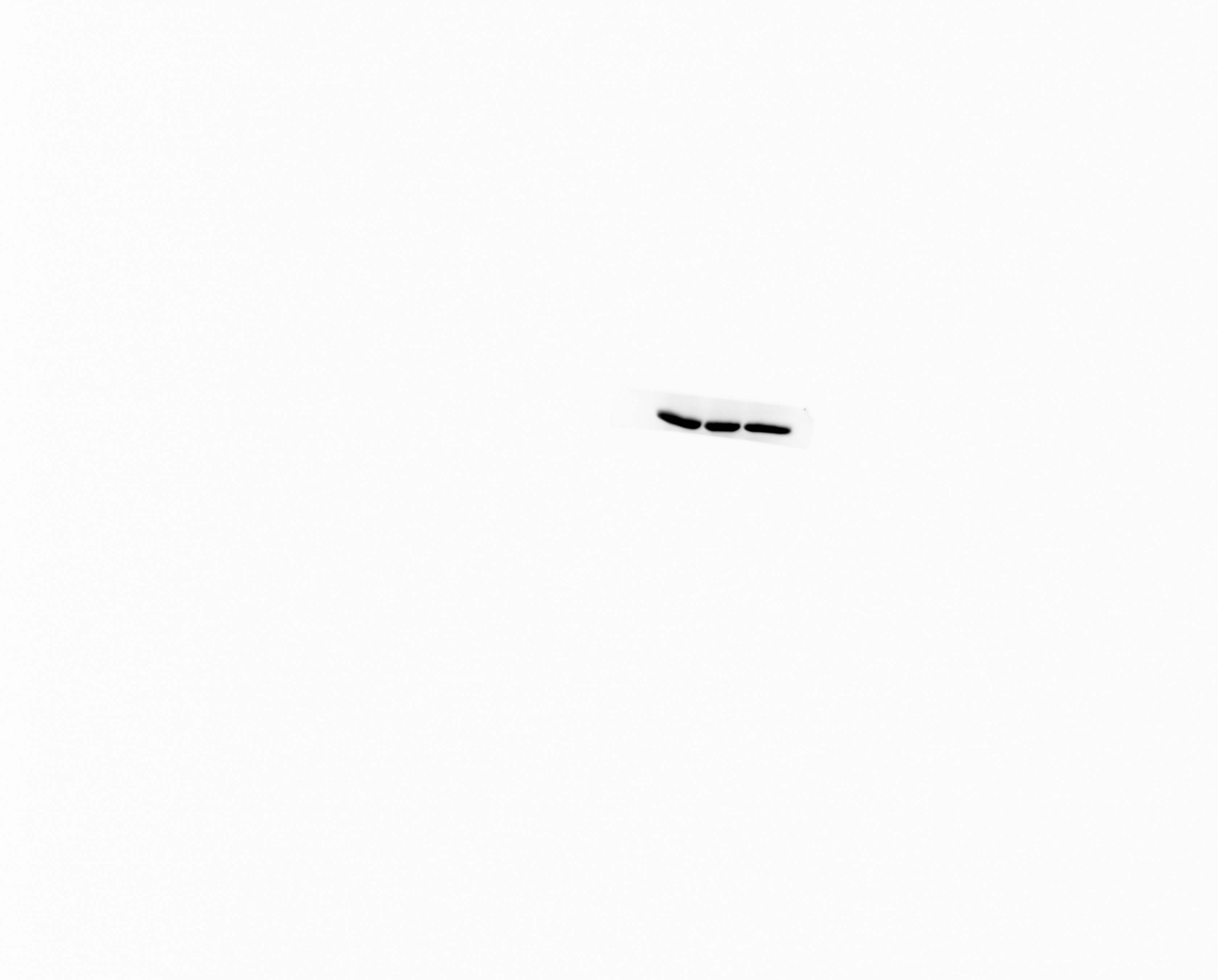

Supplement: Supplementary file 2 — Supporting File 2: advs73976‐sup‐0002‐SuppMat.zip. [file ADVS-13-e11217-s002.zip › WB#U4ee3#U8868#U56fe/xiap#U539f#U59cb#U6570#U636ewb2-JPEG/ACTIN_7 canx oedk.jpg]

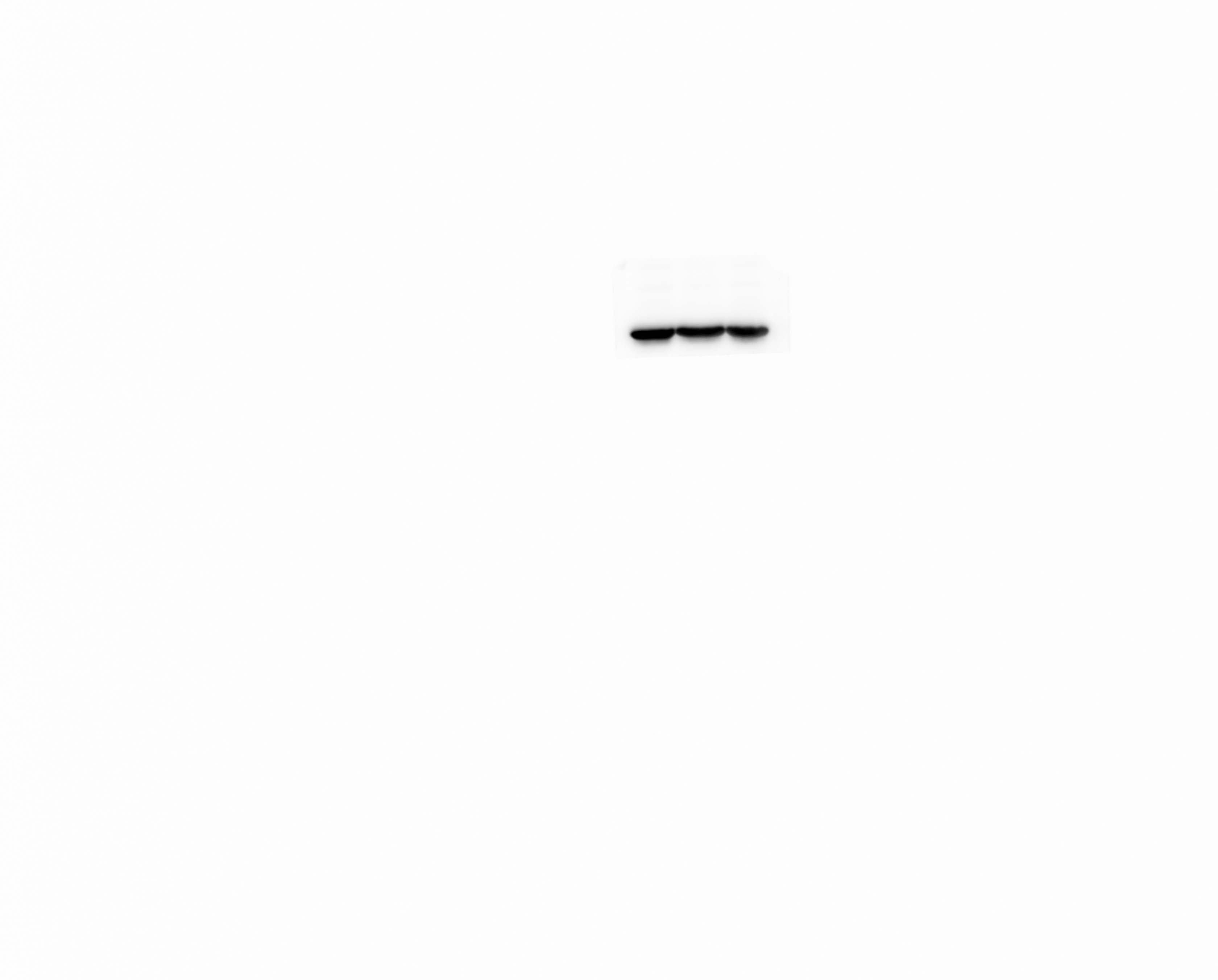

Supplement: Supplementary file 2 — Supporting File 2: advs73976‐sup‐0002‐SuppMat.zip. [file ADVS-13-e11217-s002.zip › WB#U4ee3#U8868#U56fe/xiap#U539f#U59cb#U6570#U636ewb2-JPEG/actin_7 DBXIAP vivo gas.jpg]

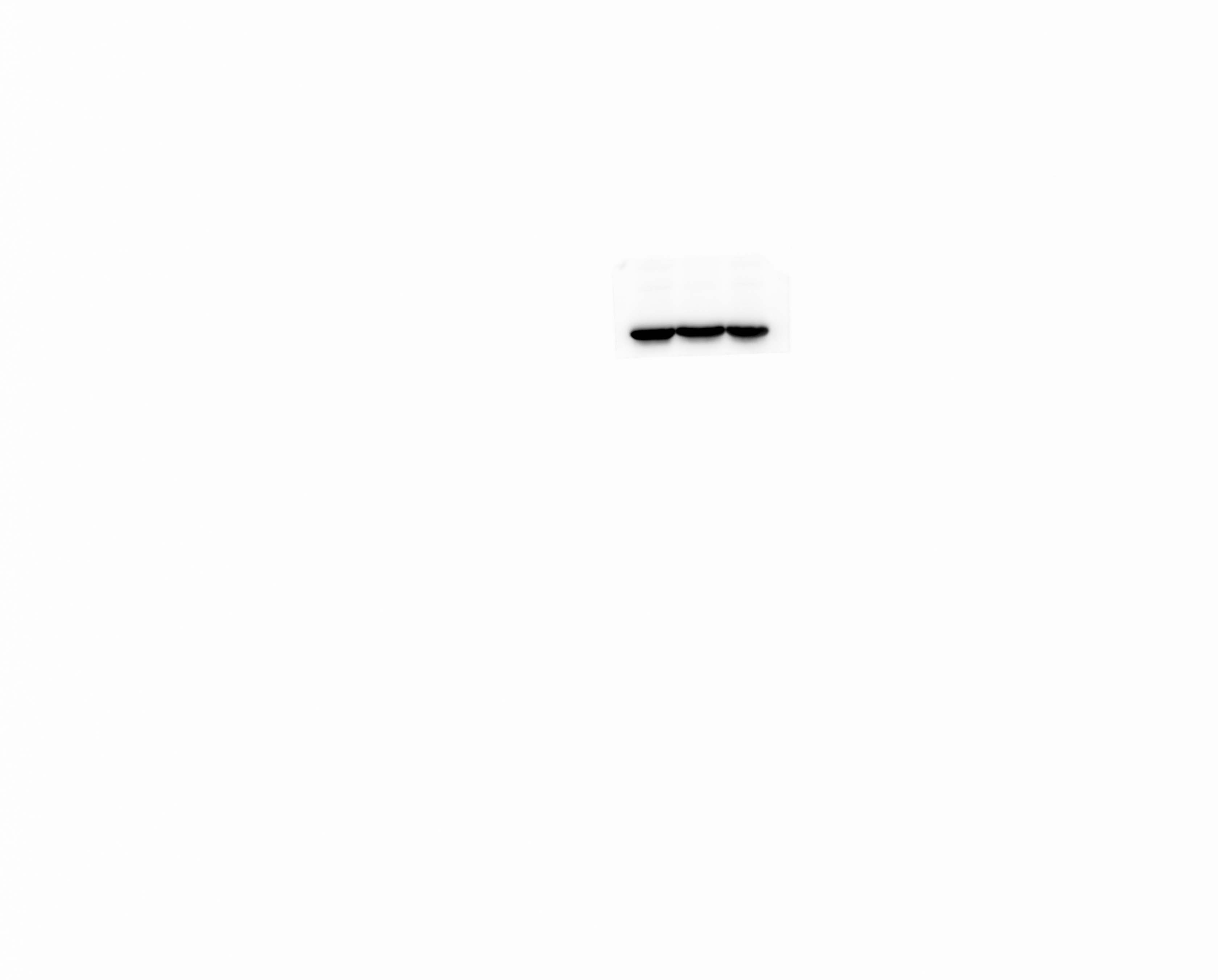

Supplement: Supplementary file 2 — Supporting File 2: advs73976‐sup‐0002‐SuppMat.zip. [file ADVS-13-e11217-s002.zip › WB#U4ee3#U8868#U56fe/xiap#U539f#U59cb#U6570#U636ewb2-JPEG/actin_8-fam.jpg]

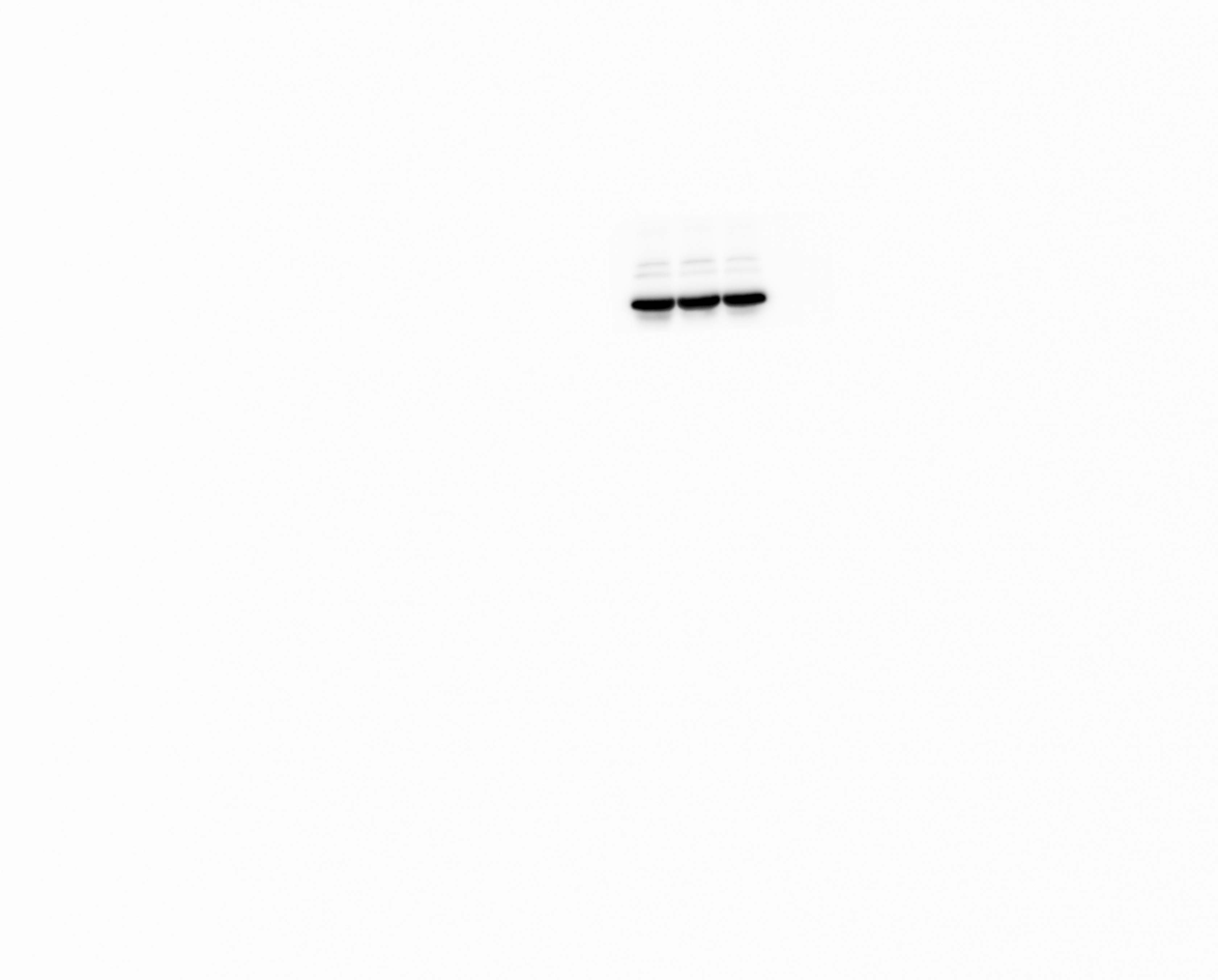

Supplement: Supplementary file 2 — Supporting File 2: advs73976‐sup‐0002‐SuppMat.zip. [file ADVS-13-e11217-s002.zip › WB#U4ee3#U8868#U56fe/xiap#U539f#U59cb#U6570#U636ewb2-JPEG/ACTIN_9 #U4ee3#U8868 p-eif.jpg]

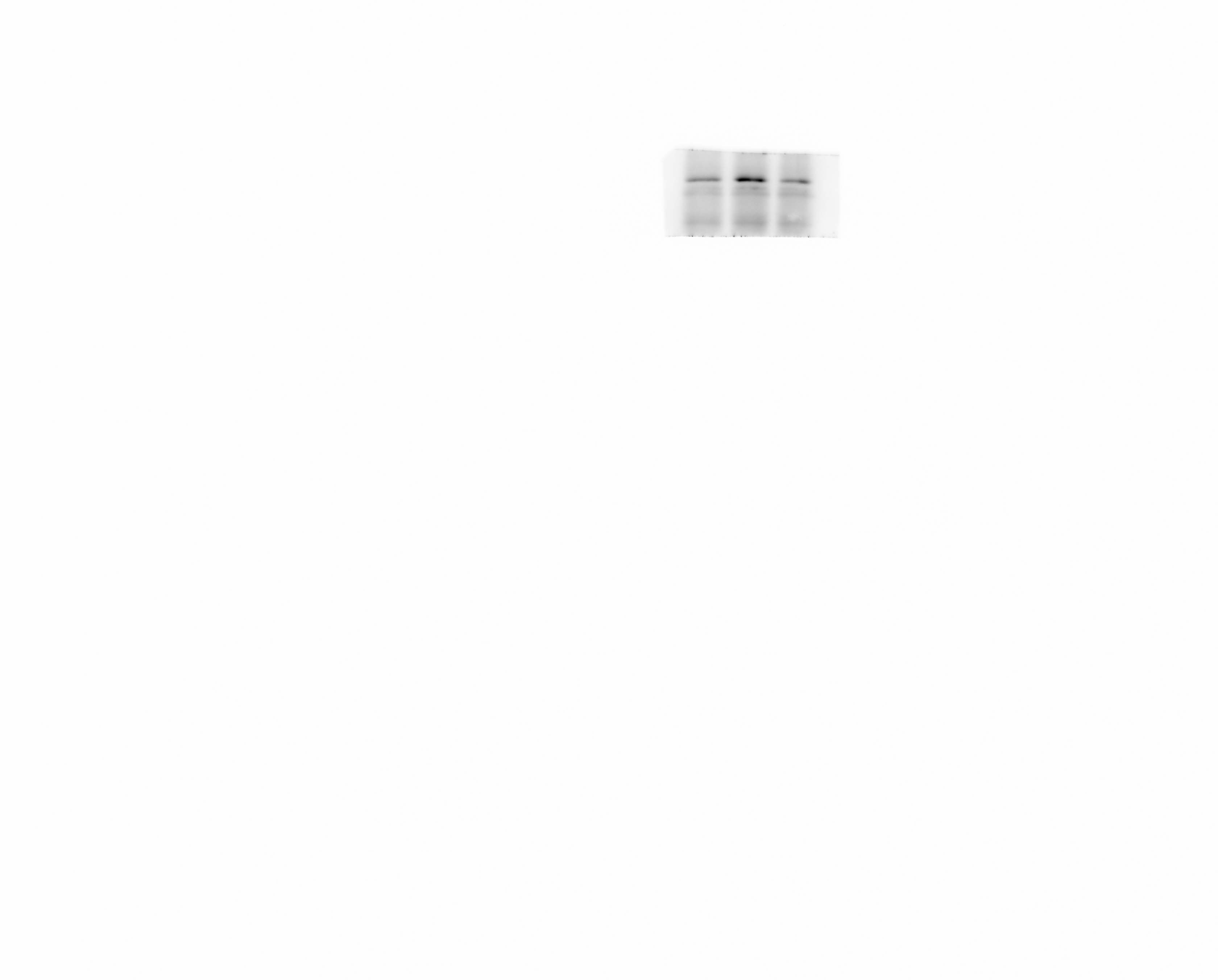

Supplement: Supplementary file 2 — Supporting File 2: advs73976‐sup‐0002‐SuppMat.zip. [file ADVS-13-e11217-s002.zip › WB#U4ee3#U8868#U56fe/xiap#U539f#U59cb#U6570#U636ewb2-JPEG/ATF4_3 #U4ee3#U8868 gas.jpg]

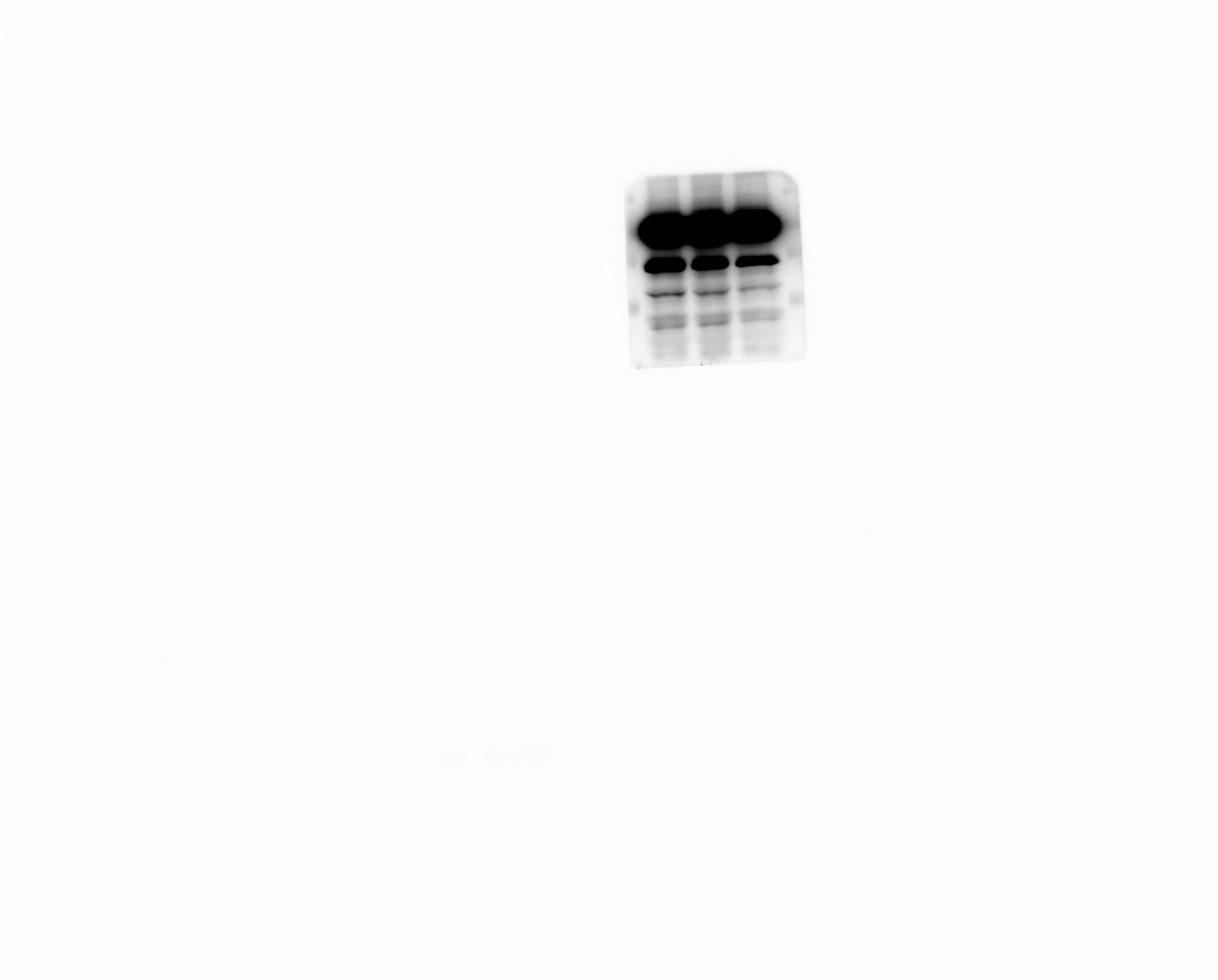

Supplement: Supplementary file 2 — Supporting File 2: advs73976‐sup‐0002‐SuppMat.zip. [file ADVS-13-e11217-s002.zip › WB#U4ee3#U8868#U56fe/xiap#U539f#U59cb#U6570#U636ewb2-JPEG/atl 9 db six.jpg]

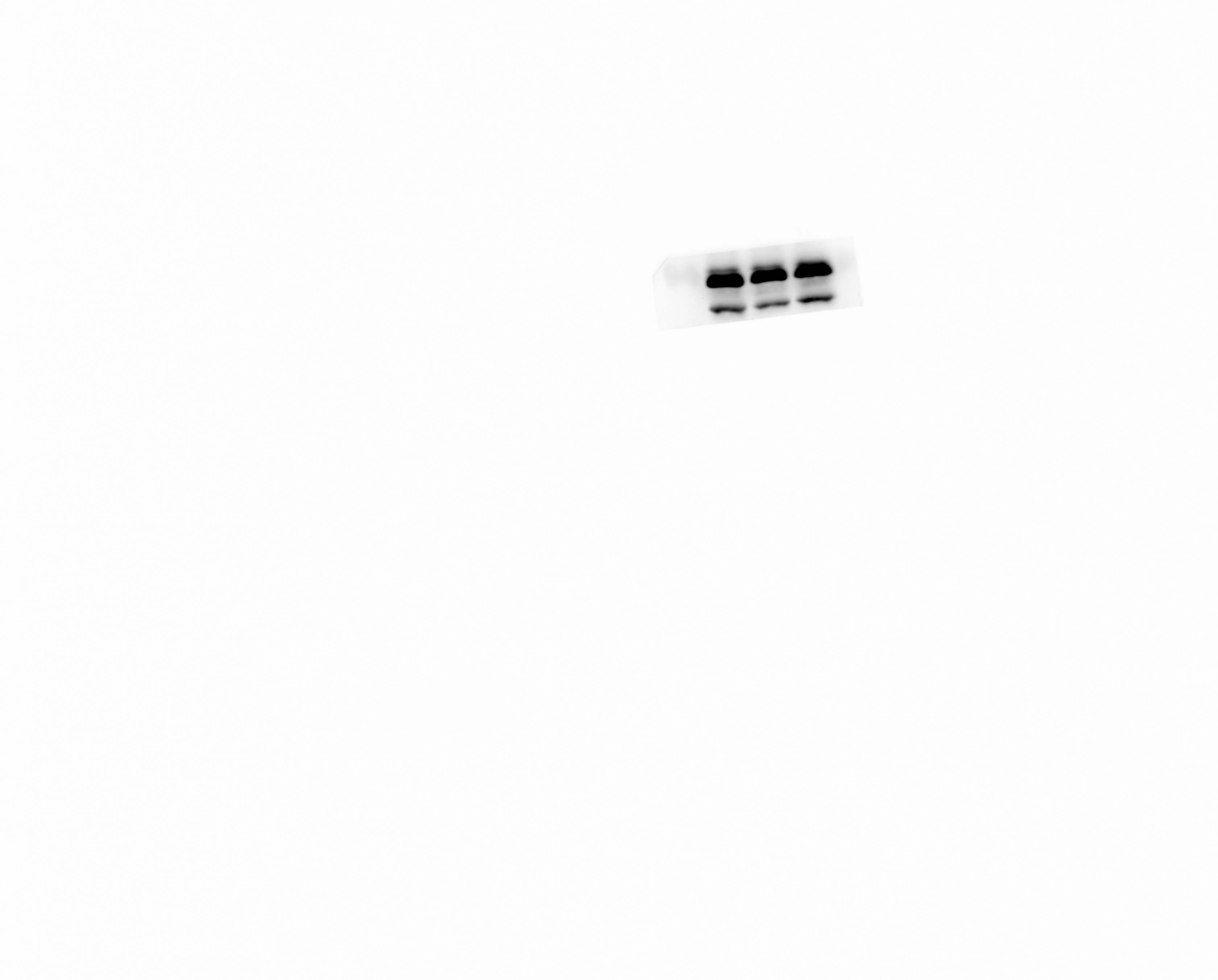

Supplement: Supplementary file 2 — Supporting File 2: advs73976‐sup‐0002‐SuppMat.zip. [file ADVS-13-e11217-s002.zip › WB#U4ee3#U8868#U56fe/xiap#U539f#U59cb#U6570#U636ewb2-JPEG/atl310s_4 oedk.jpg]

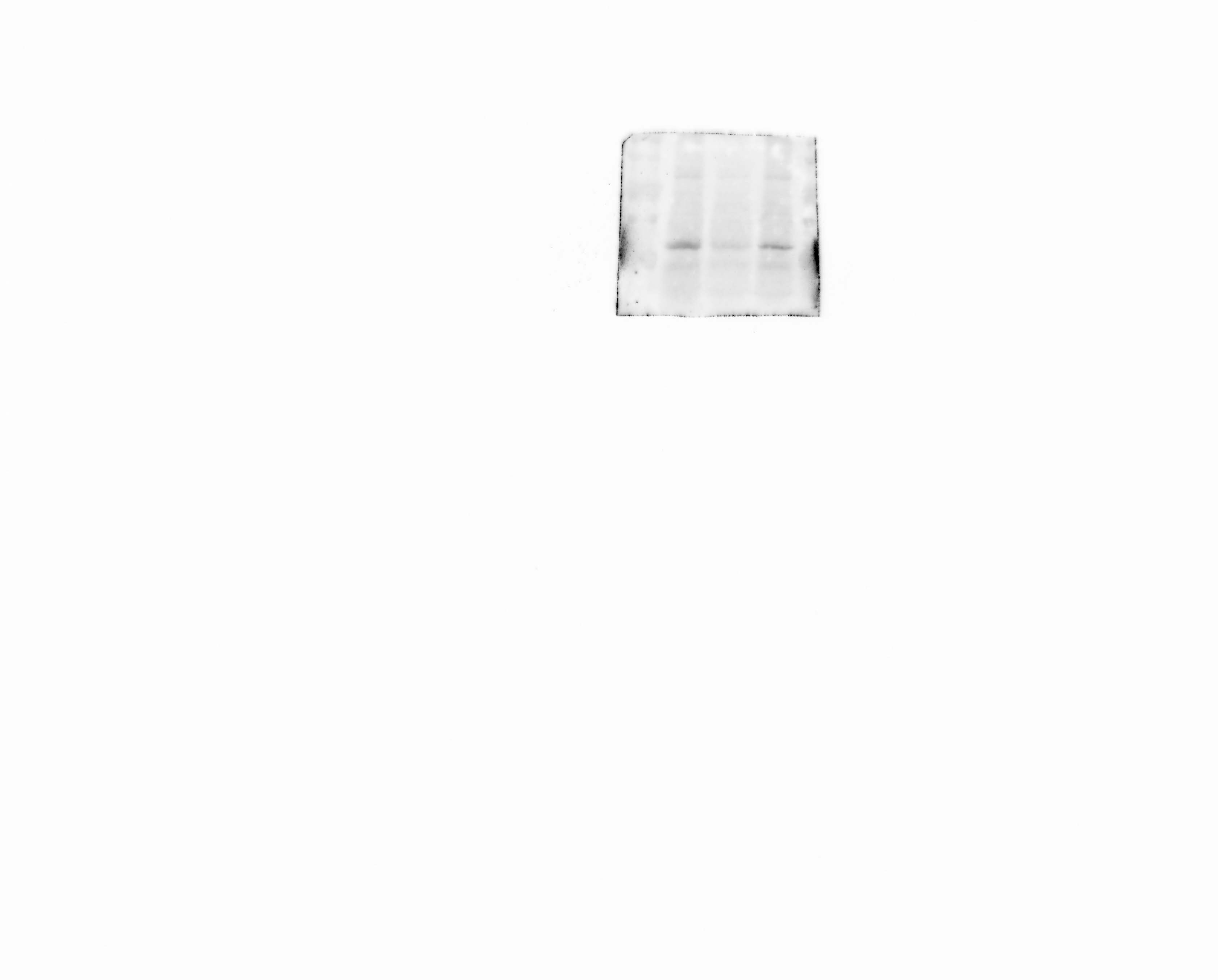

Supplement: Supplementary file 2 — Supporting File 2: advs73976‐sup‐0002‐SuppMat.zip. [file ADVS-13-e11217-s002.zip › WB#U4ee3#U8868#U56fe/xiap#U539f#U59cb#U6570#U636ewb2-JPEG/ATL3_10.jpg]

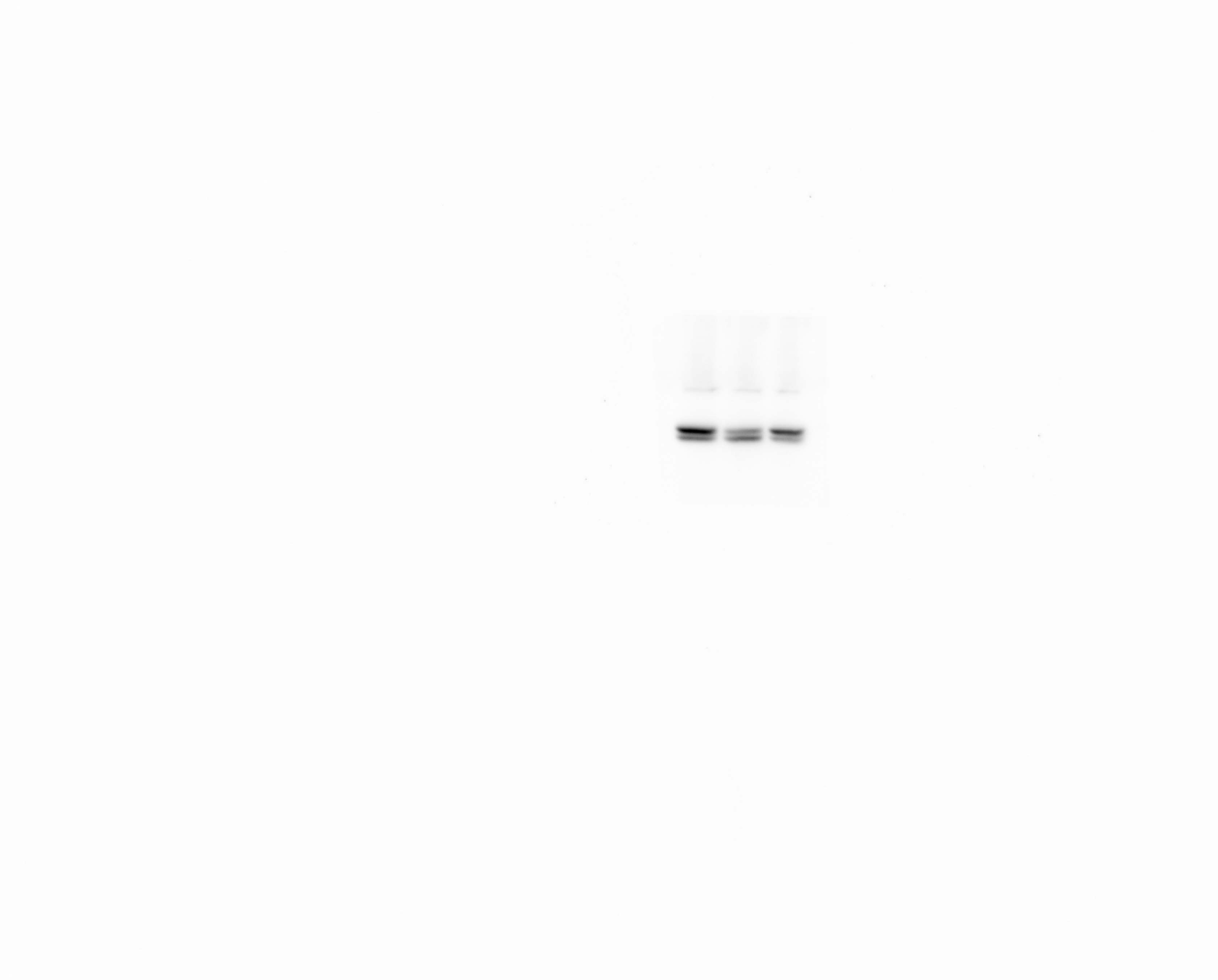

Supplement: Supplementary file 2 — Supporting File 2: advs73976‐sup‐0002‐SuppMat.zip. [file ADVS-13-e11217-s002.zip › WB#U4ee3#U8868#U56fe/xiap#U539f#U59cb#U6570#U636ewb2-JPEG/ATL3_4 oex.jpg]

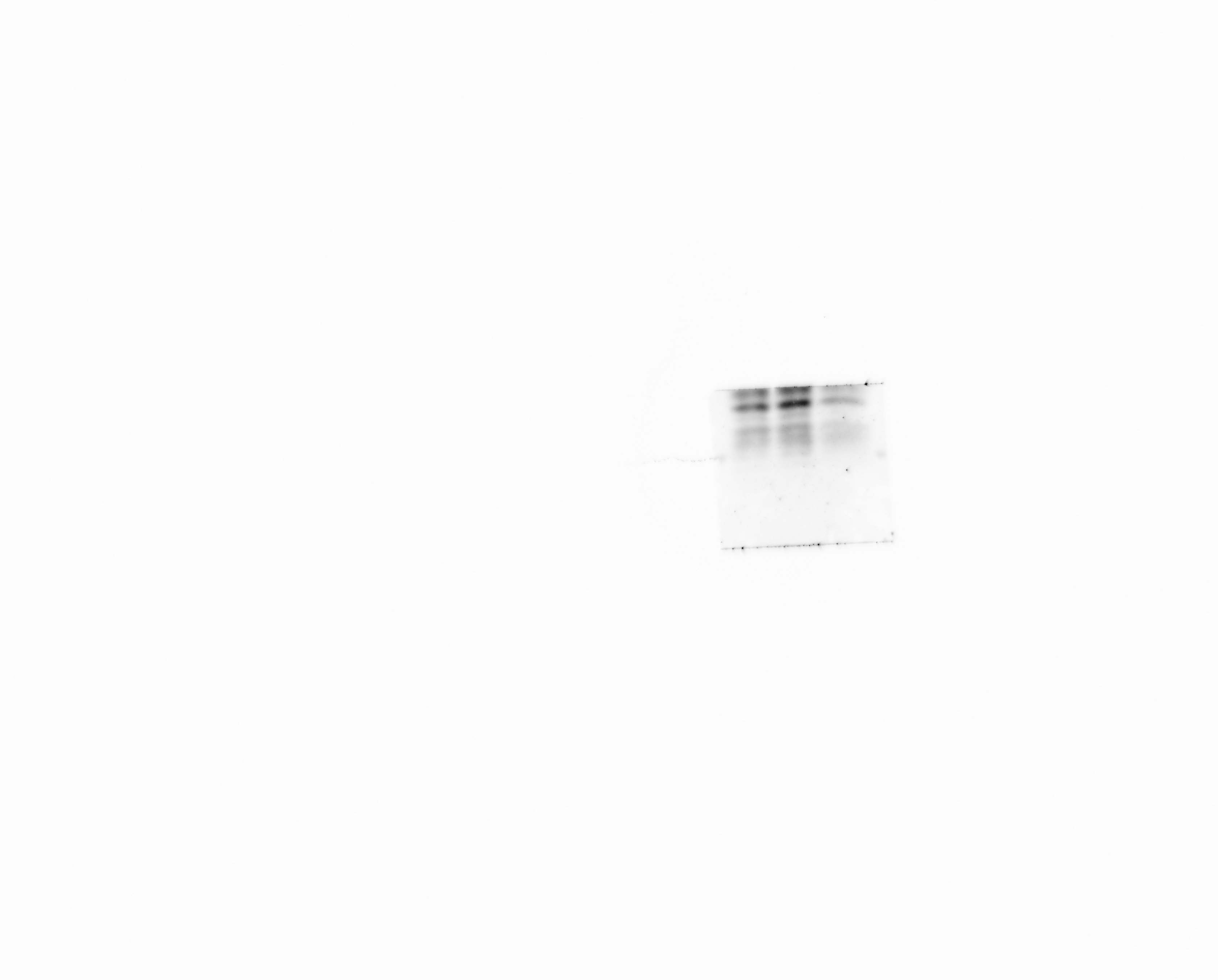

Supplement: Supplementary file 2 — Supporting File 2: advs73976‐sup‐0002‐SuppMat.zip. [file ADVS-13-e11217-s002.zip › WB#U4ee3#U8868#U56fe/xiap#U539f#U59cb#U6570#U636ewb2-JPEG/c-casps3-_5 #U4ee3#U8868 oedk.jpg]

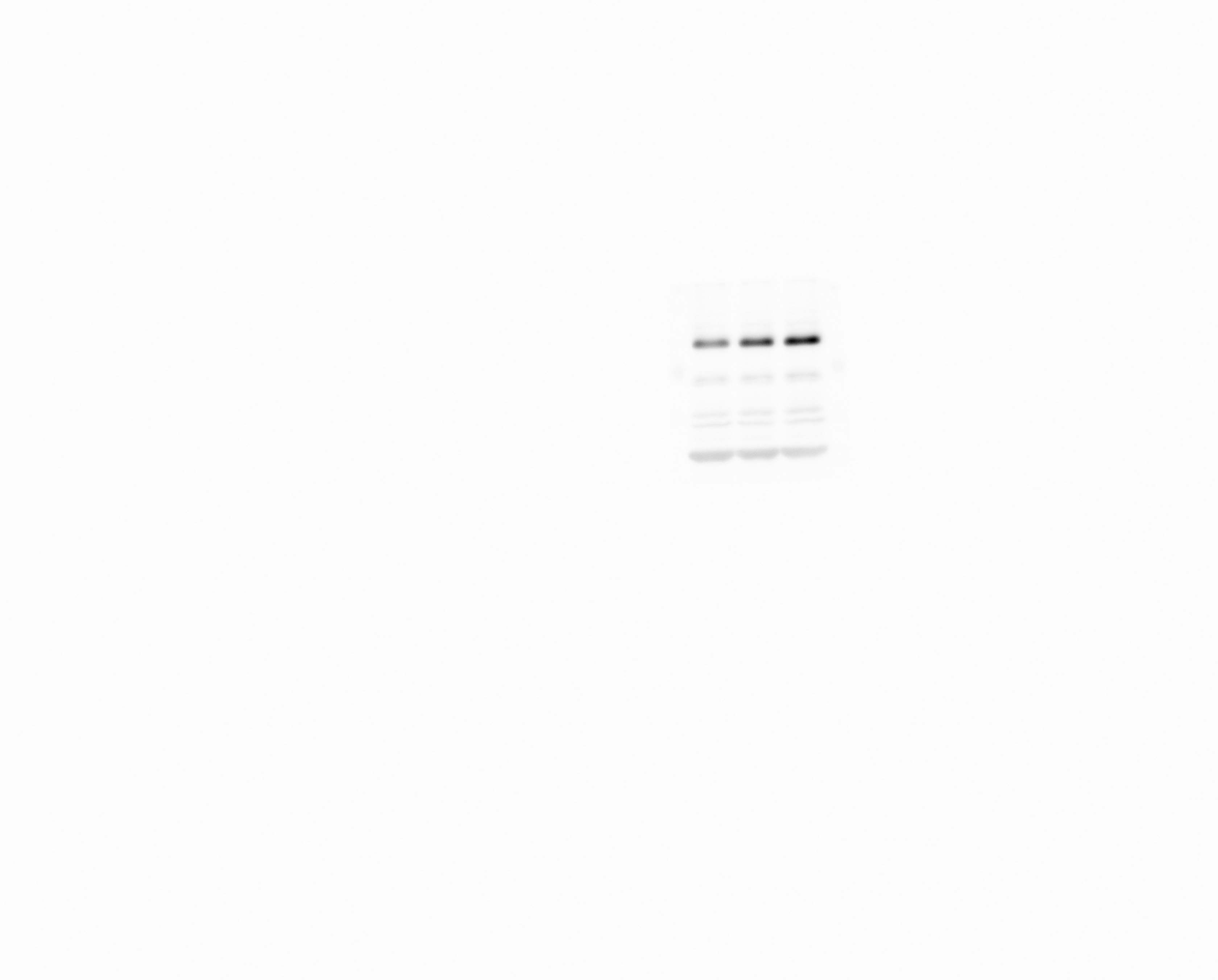

Supplement: Supplementary file 2 — Supporting File 2: advs73976‐sup‐0002‐SuppMat.zip. [file ADVS-13-e11217-s002.zip › WB#U4ee3#U8868#U56fe/xiap#U539f#U59cb#U6570#U636ewb2-JPEG/canx1_1 six.jpg]

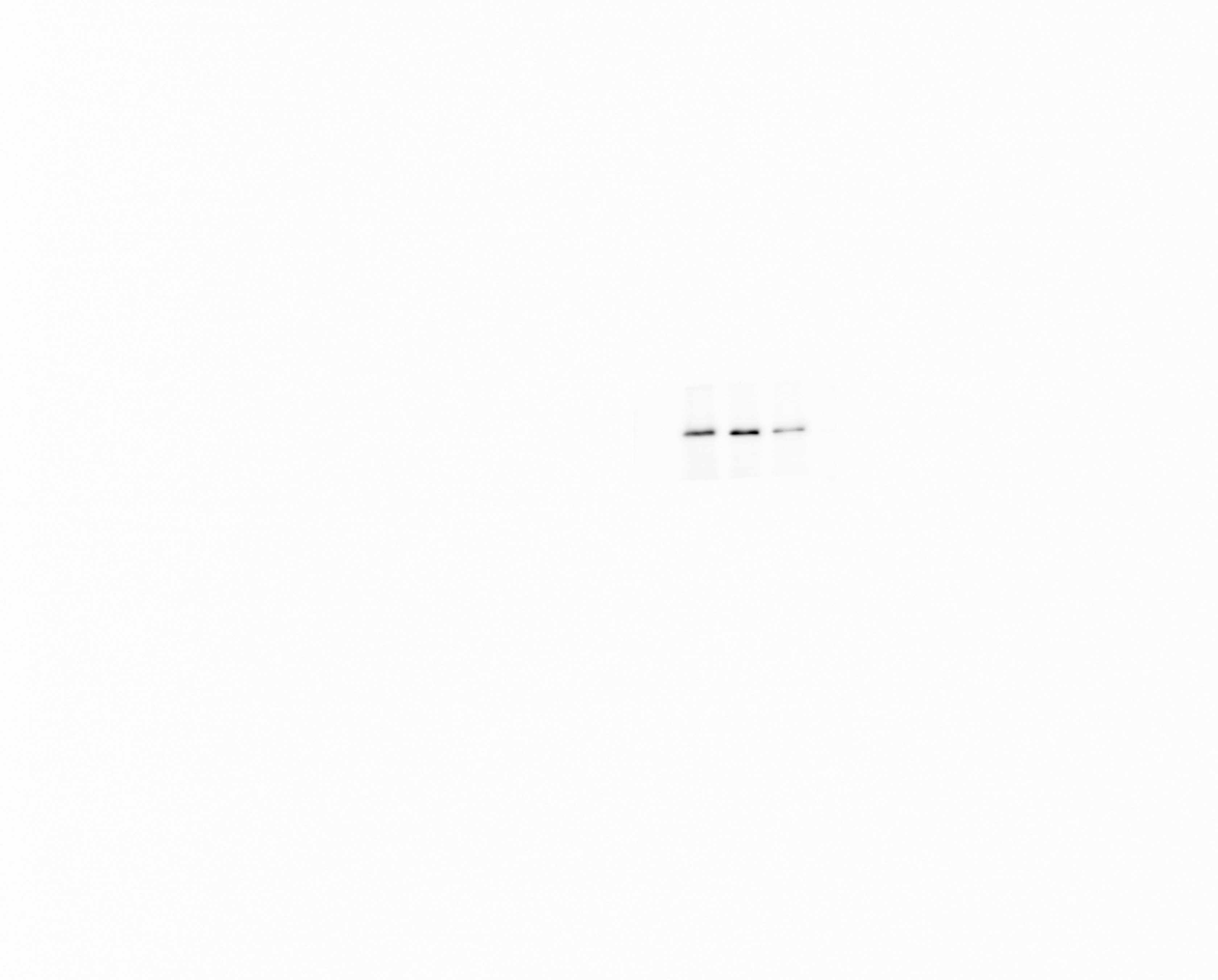

Supplement: Supplementary file 2 — Supporting File 2: advs73976‐sup‐0002‐SuppMat.zip. [file ADVS-13-e11217-s002.zip › WB#U4ee3#U8868#U56fe/xiap#U539f#U59cb#U6570#U636ewb2-JPEG/CANX1_7#U4ee3#U8868 oedk.jpg]

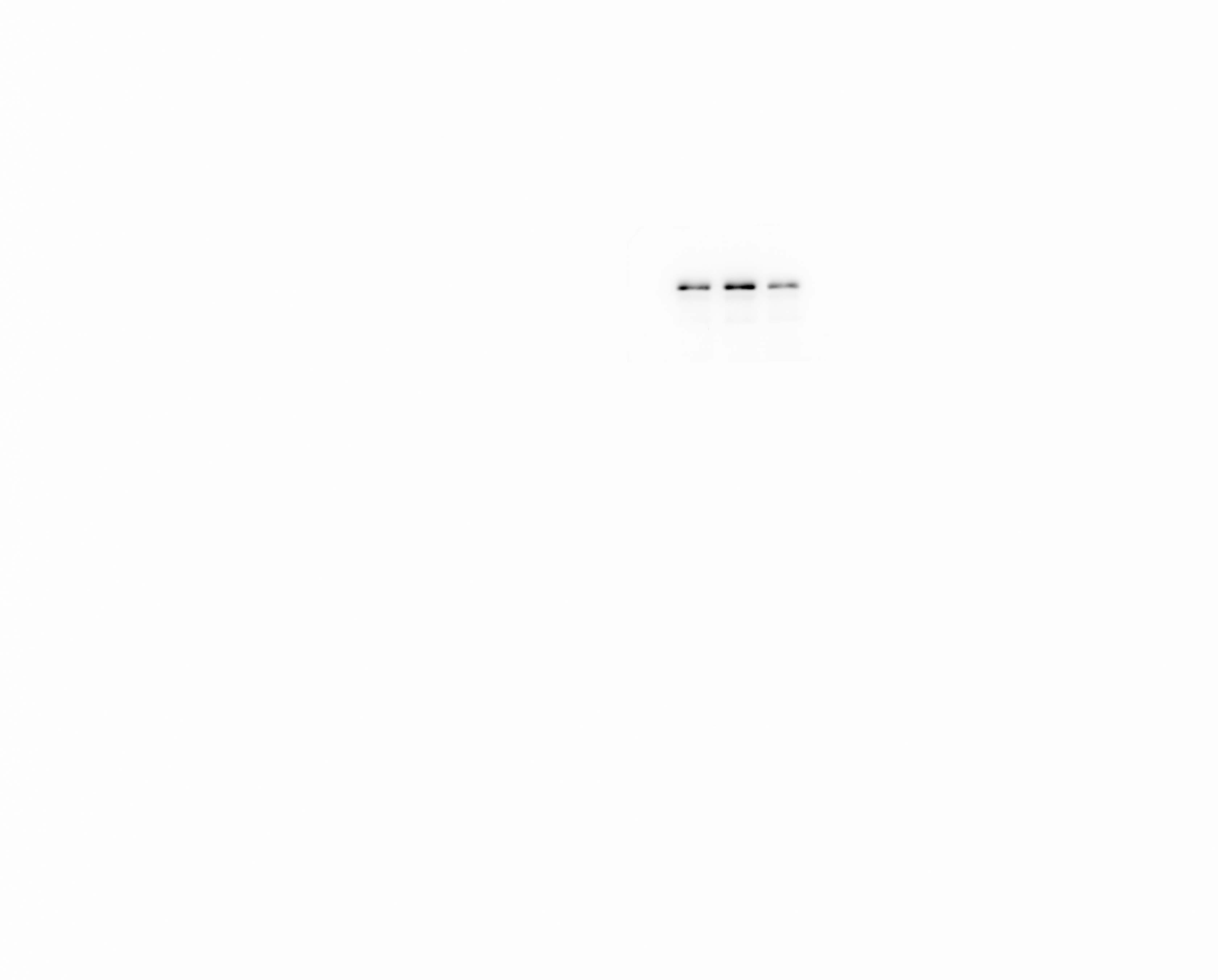

Supplement: Supplementary file 2 — Supporting File 2: advs73976‐sup‐0002‐SuppMat.zip. [file ADVS-13-e11217-s002.zip › WB#U4ee3#U8868#U56fe/xiap#U539f#U59cb#U6570#U636ewb2-JPEG/CANX_5 DB GAS.jpg]

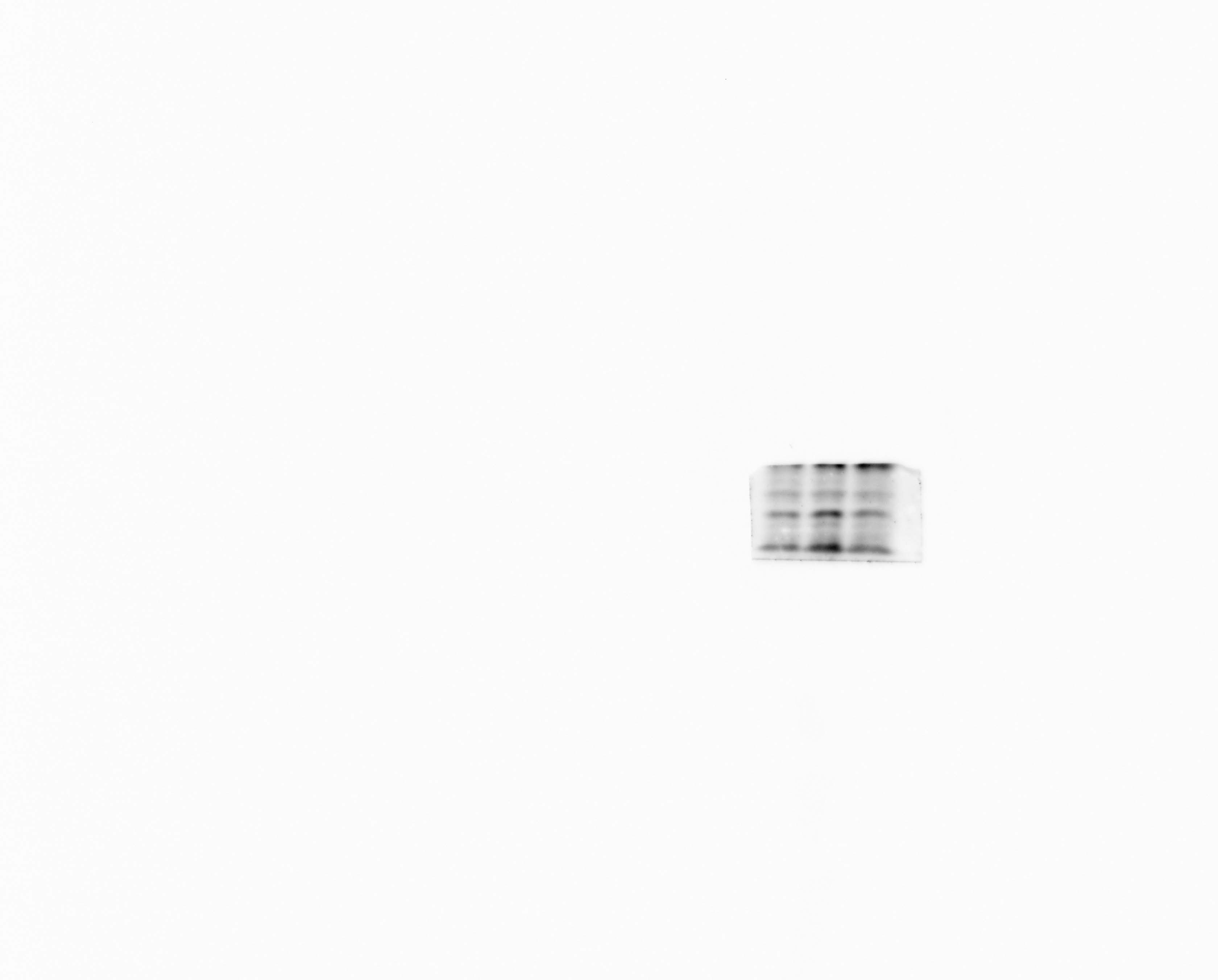

Supplement: Supplementary file 2 — Supporting File 2: advs73976‐sup‐0002‐SuppMat.zip. [file ADVS-13-e11217-s002.zip › WB#U4ee3#U8868#U56fe/xiap#U539f#U59cb#U6570#U636ewb2-JPEG/CHOP1_6 #U4ee3#U8868 oedk.jpg]

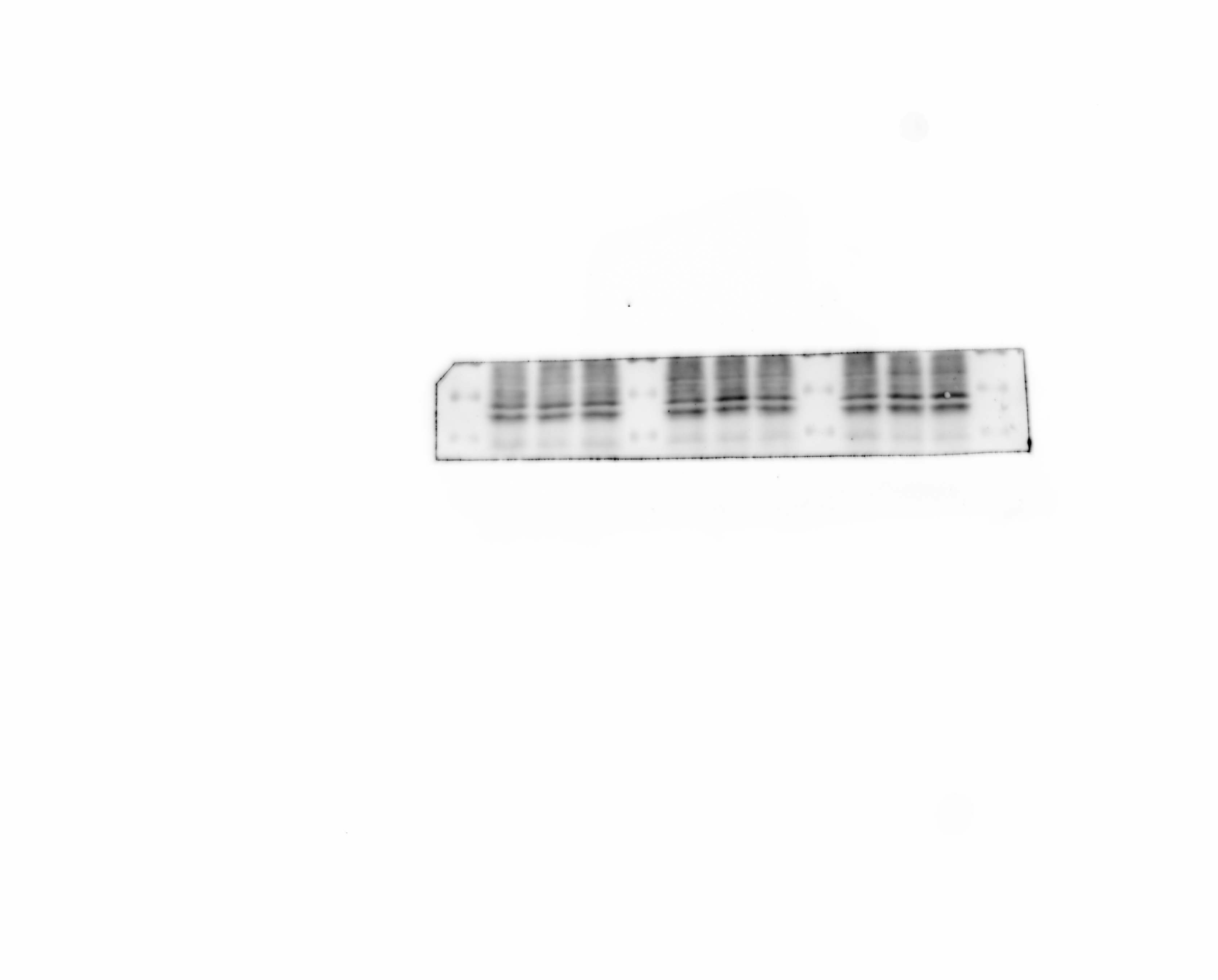

Supplement: Supplementary file 2 — Supporting File 2: advs73976‐sup‐0002‐SuppMat.zip. [file ADVS-13-e11217-s002.zip › WB#U4ee3#U8868#U56fe/xiap#U539f#U59cb#U6570#U636ewb2-JPEG/CHOP1_8.jpg]

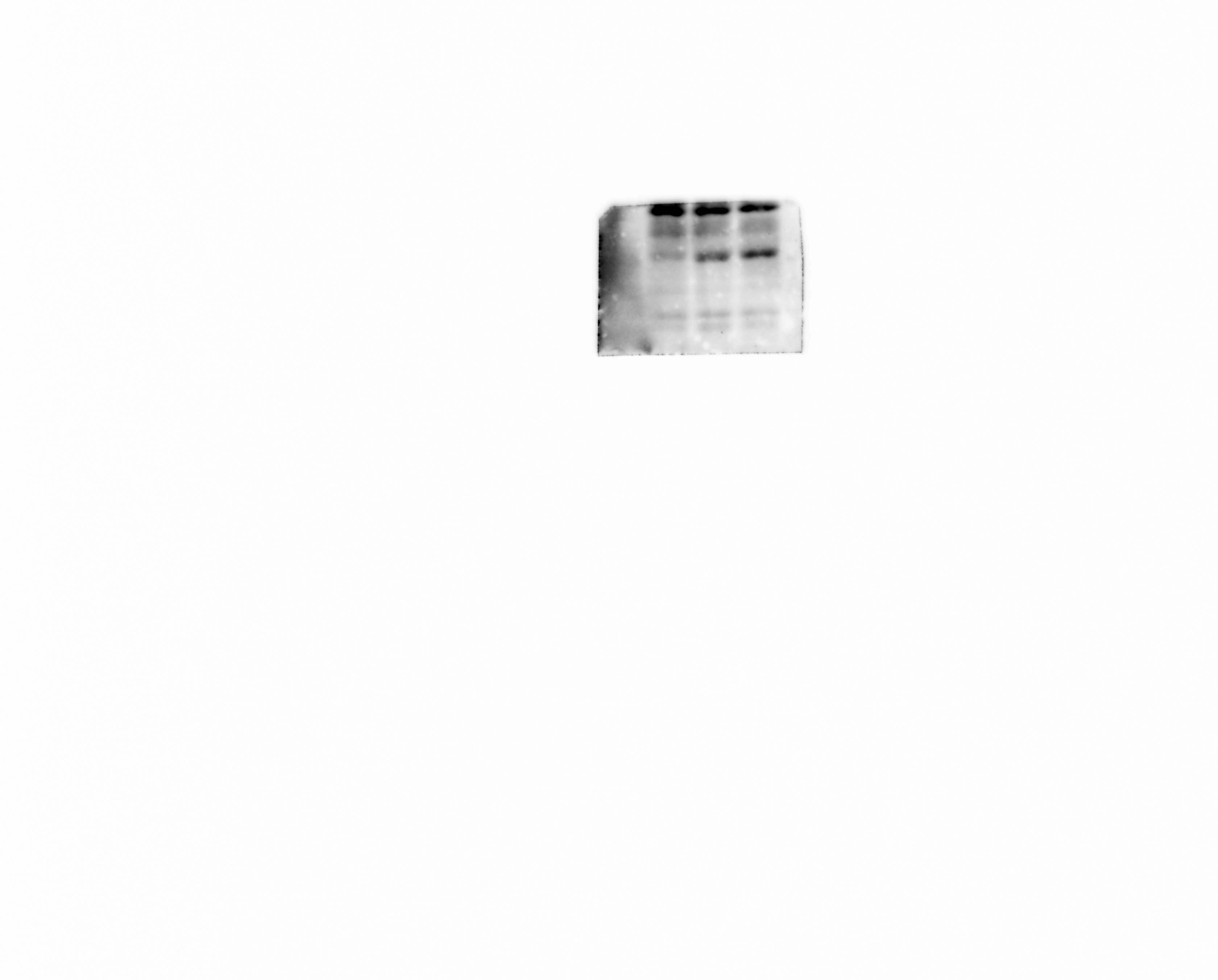

Supplement: Supplementary file 2 — Supporting File 2: advs73976‐sup‐0002‐SuppMat.zip. [file ADVS-13-e11217-s002.zip › WB#U4ee3#U8868#U56fe/xiap#U539f#U59cb#U6570#U636ewb2-JPEG/CHOP_6 six.jpg]

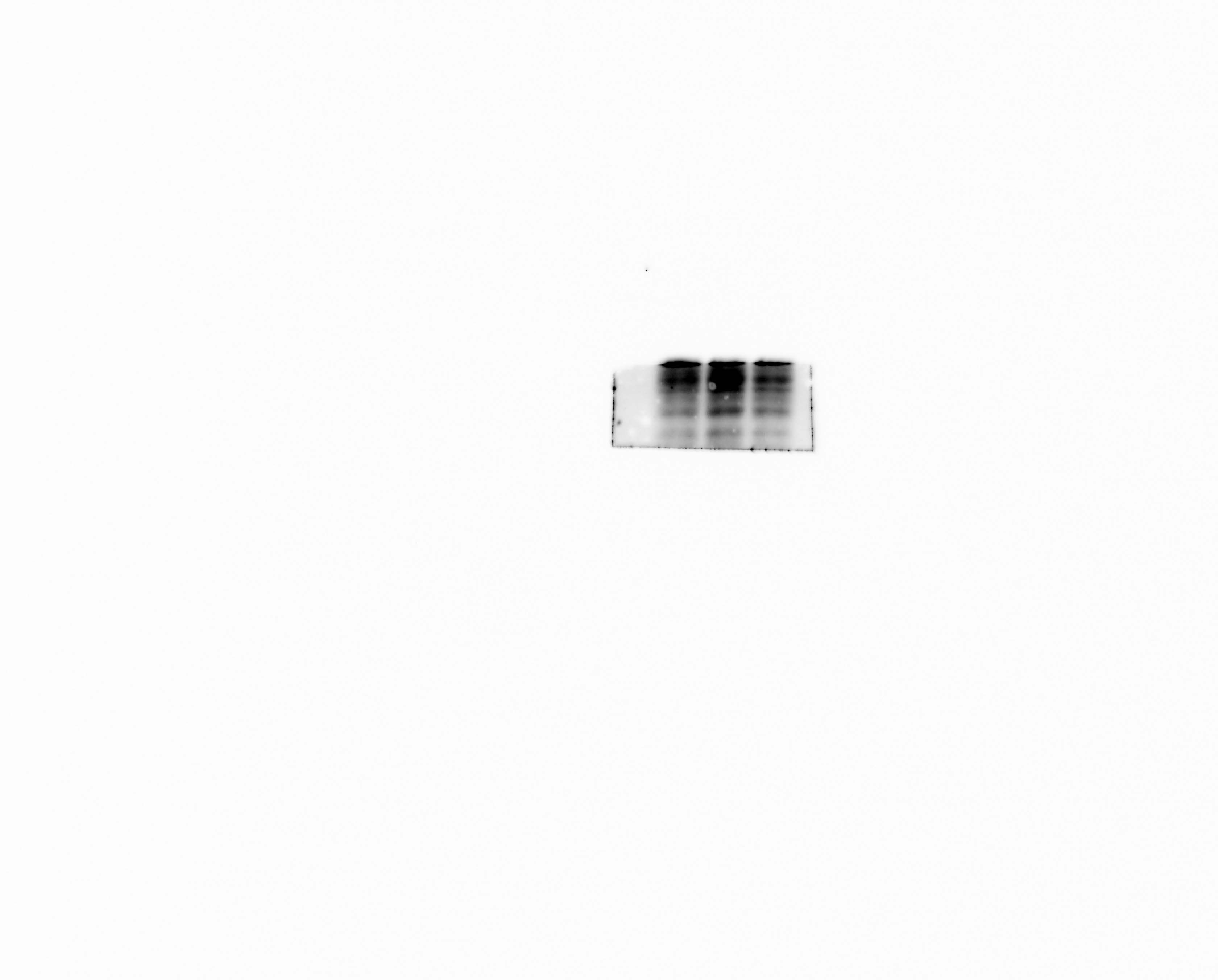

Supplement: Supplementary file 2 — Supporting File 2: advs73976‐sup‐0002‐SuppMat.zip. [file ADVS-13-e11217-s002.zip › WB#U4ee3#U8868#U56fe/xiap#U539f#U59cb#U6570#U636ewb2-JPEG/CHOP_9 #U4ee3#U8868.jpg]

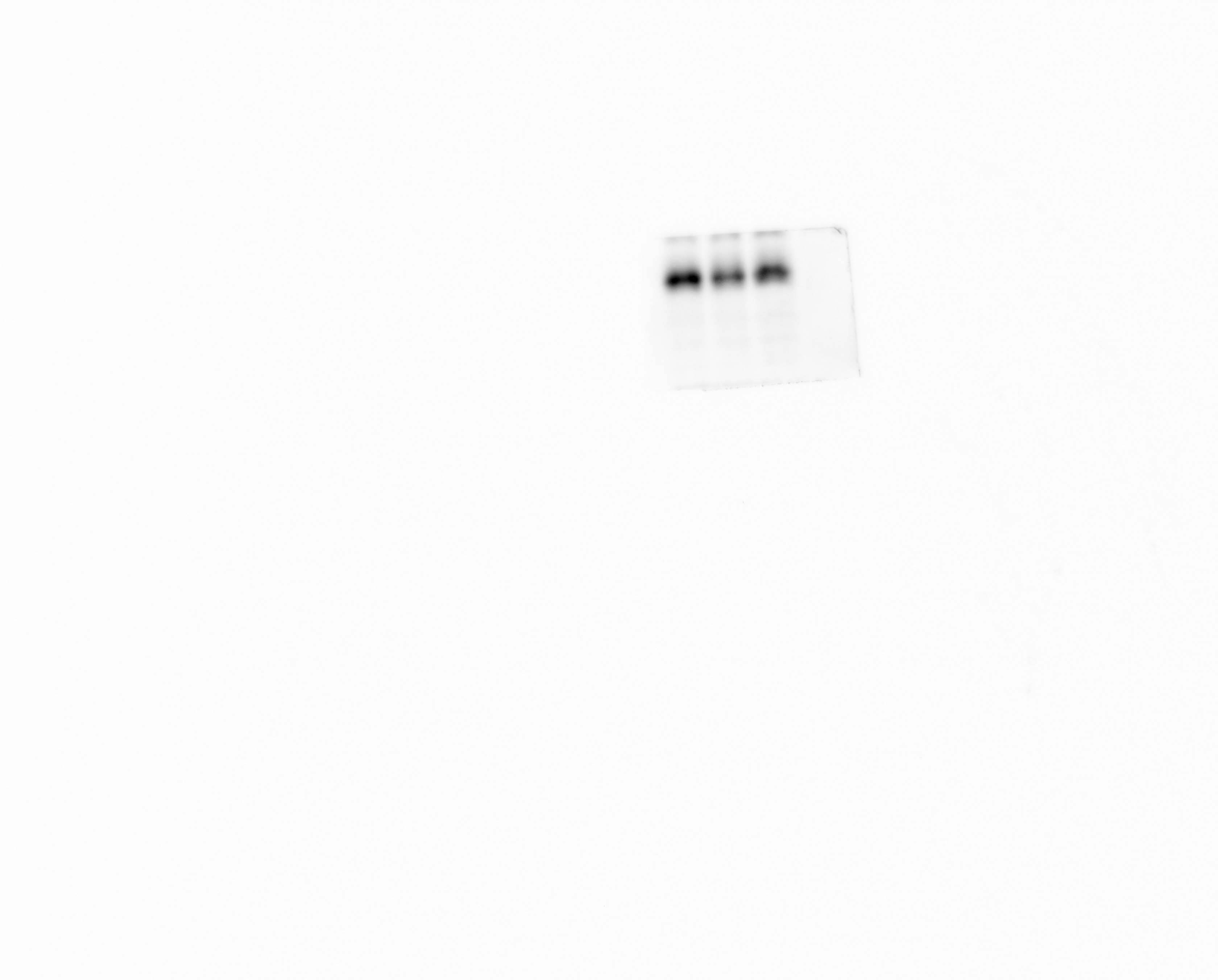

Supplement: Supplementary file 2 — Supporting File 2: advs73976‐sup‐0002‐SuppMat.zip. [file ADVS-13-e11217-s002.zip › WB#U4ee3#U8868#U56fe/xiap#U539f#U59cb#U6570#U636ewb2-JPEG/ctsb1_9 #U4ee3#U8868 oedk.jpg]

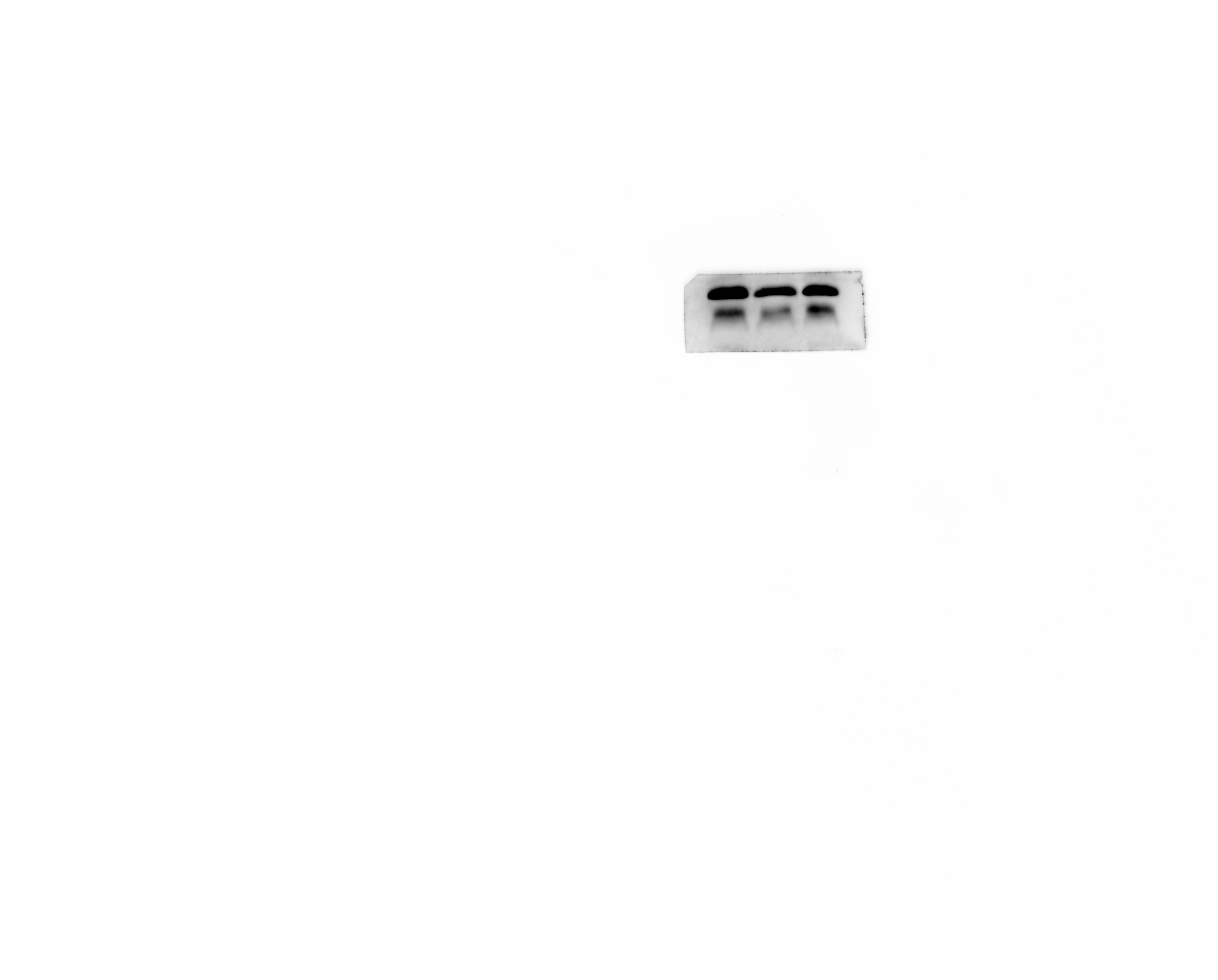

Supplement: Supplementary file 2 — Supporting File 2: advs73976‐sup‐0002‐SuppMat.zip. [file ADVS-13-e11217-s002.zip › WB#U4ee3#U8868#U56fe/xiap#U539f#U59cb#U6570#U636ewb2-JPEG/CTSB_10.jpg]

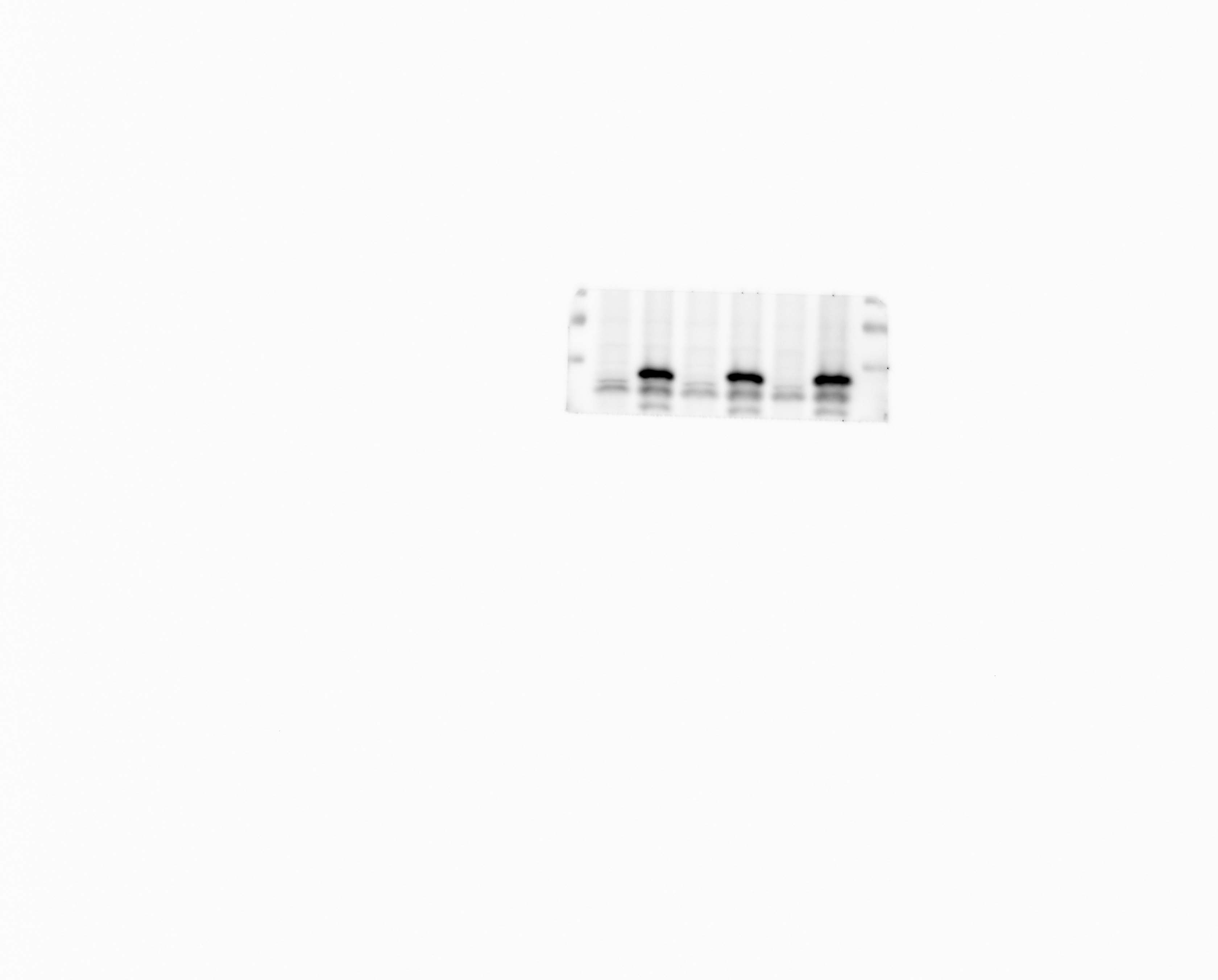

Supplement: Supplementary file 2 — Supporting File 2: advs73976‐sup‐0002‐SuppMat.zip. [file ADVS-13-e11217-s002.zip › WB#U4ee3#U8868#U56fe/xiap#U539f#U59cb#U6570#U636ewb2-JPEG/DK-FG_8#U4ee3#U8868#U6548#U7387.jpg]

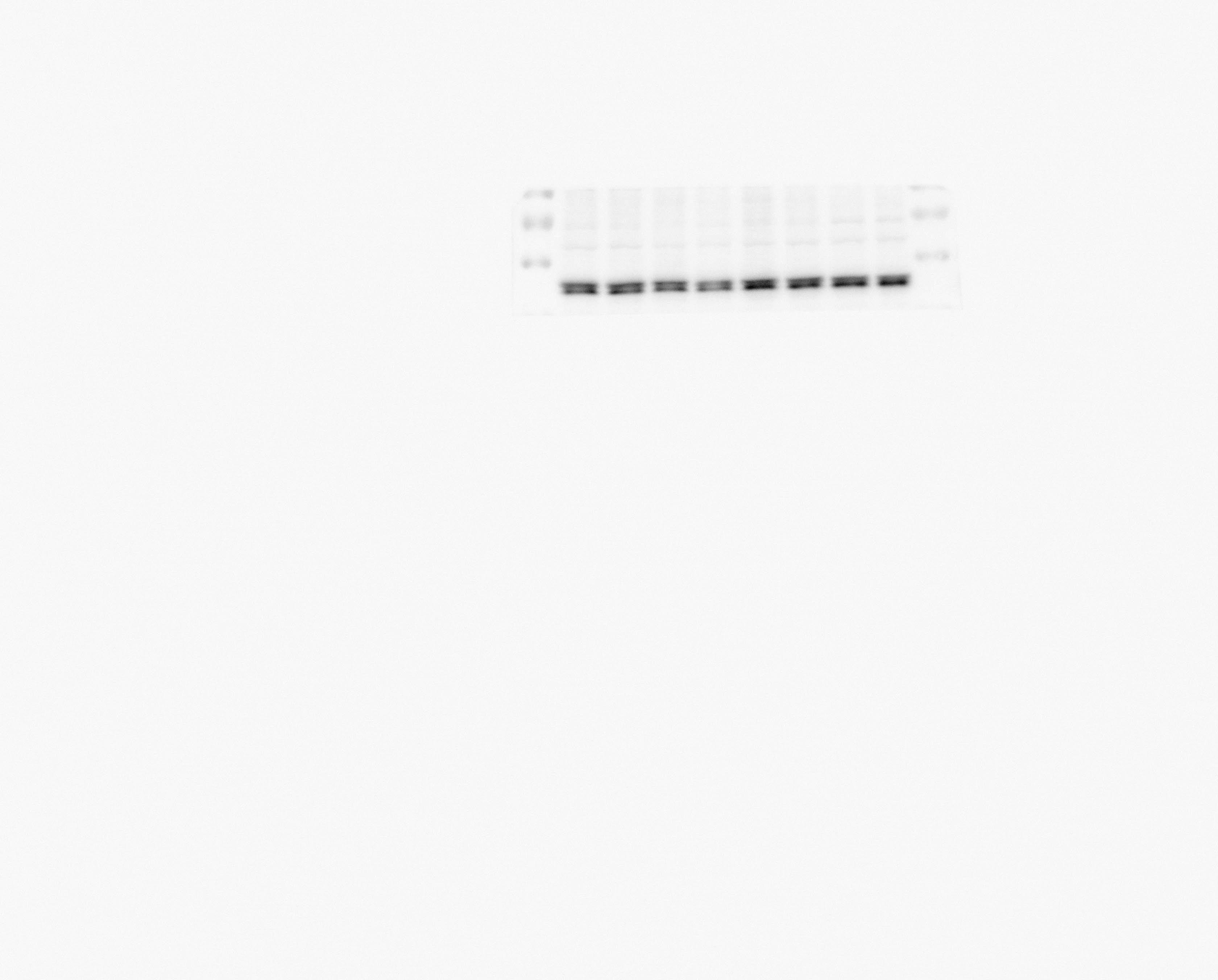

Supplement: Supplementary file 2 — Supporting File 2: advs73976‐sup‐0002‐SuppMat.zip. [file ADVS-13-e11217-s002.zip › WB#U4ee3#U8868#U56fe/xiap#U539f#U59cb#U6570#U636ewb2-JPEG/DK1_1 #U4ee3#U8868 oechx.jpg]

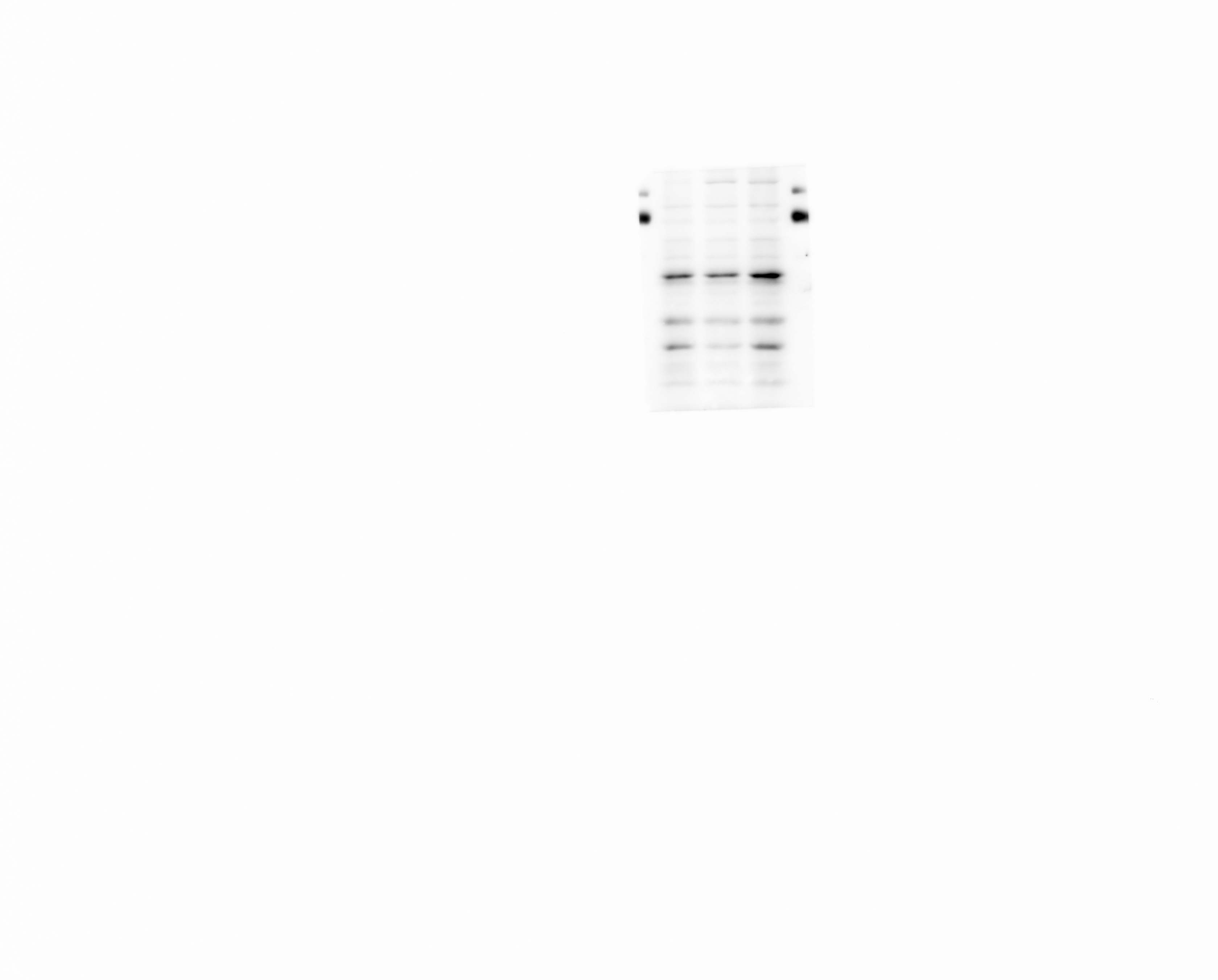

Supplement: Supplementary file 2 — Supporting File 2: advs73976‐sup‐0002‐SuppMat.zip. [file ADVS-13-e11217-s002.zip › WB#U4ee3#U8868#U56fe/xiap#U539f#U59cb#U6570#U636ewb2-JPEG/DK1_5db oex.jpg]

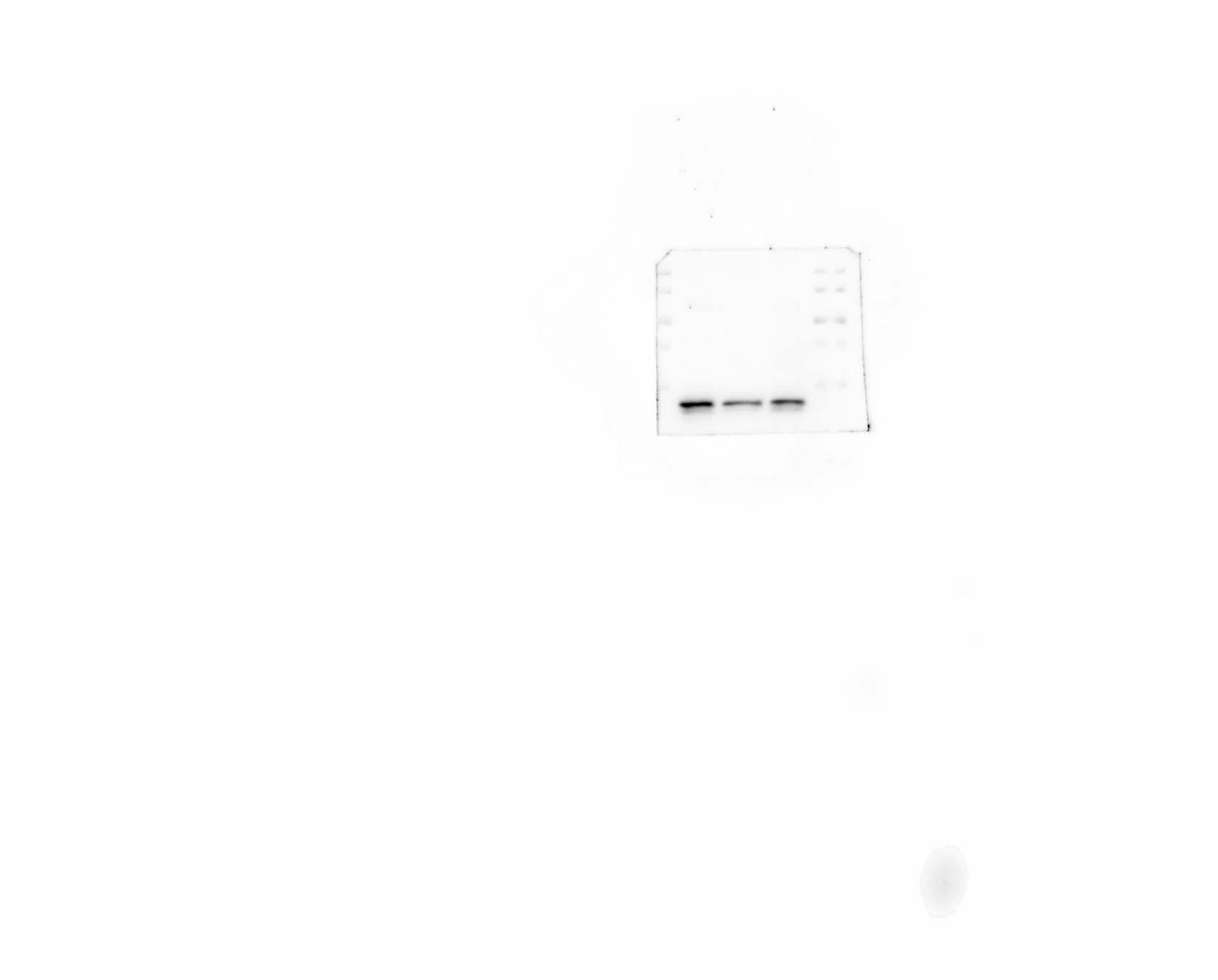

Supplement: Supplementary file 2 — Supporting File 2: advs73976‐sup‐0002‐SuppMat.zip. [file ADVS-13-e11217-s002.zip › WB#U4ee3#U8868#U56fe/xiap#U539f#U59cb#U6570#U636ewb2-JPEG/DK1_7.jpg]

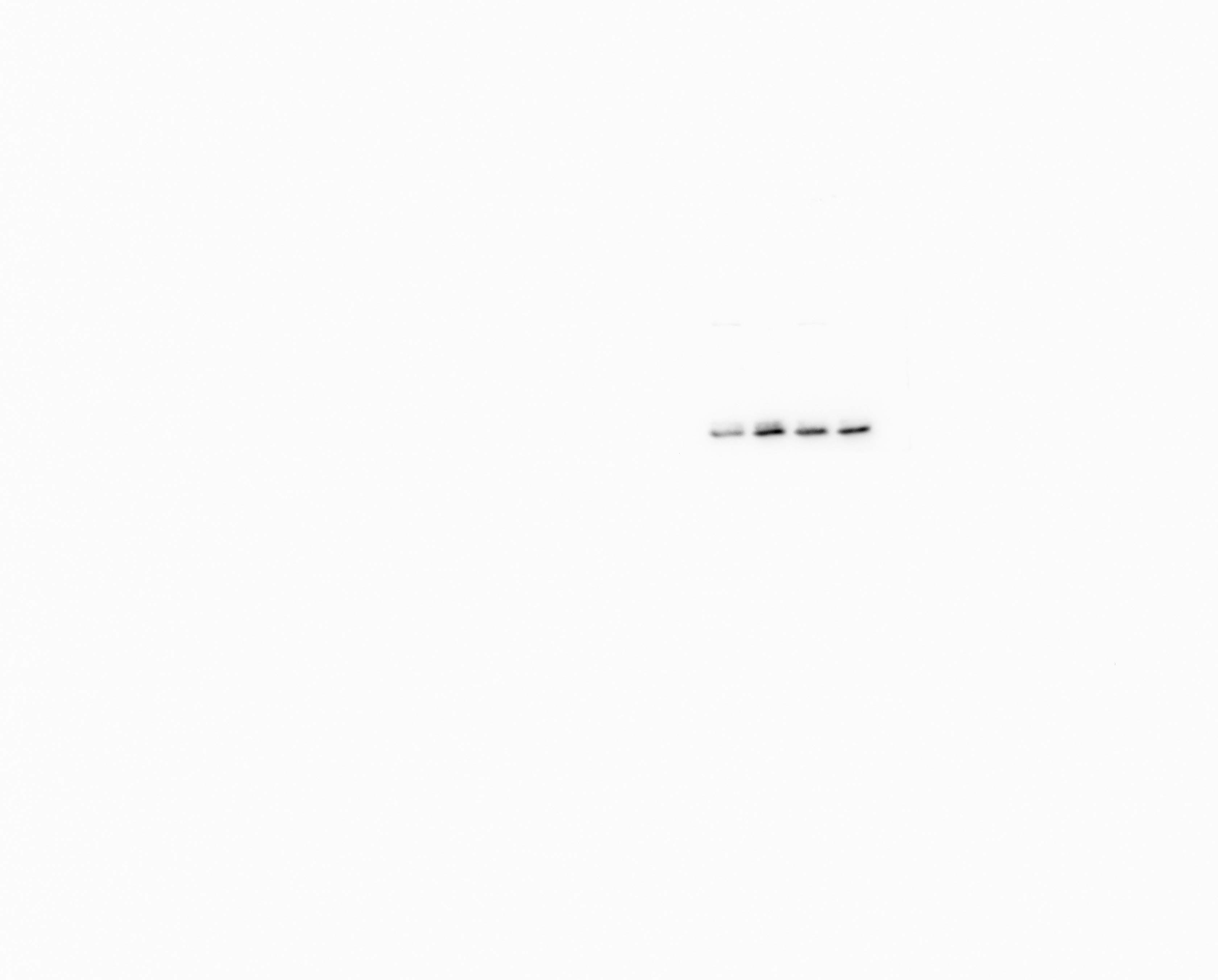

Supplement: Supplementary file 2 — Supporting File 2: advs73976‐sup‐0002‐SuppMat.zip. [file ADVS-13-e11217-s002.zip › WB#U4ee3#U8868#U56fe/xiap#U539f#U59cb#U6570#U636ewb2-JPEG/dk_1 oex.jpg]

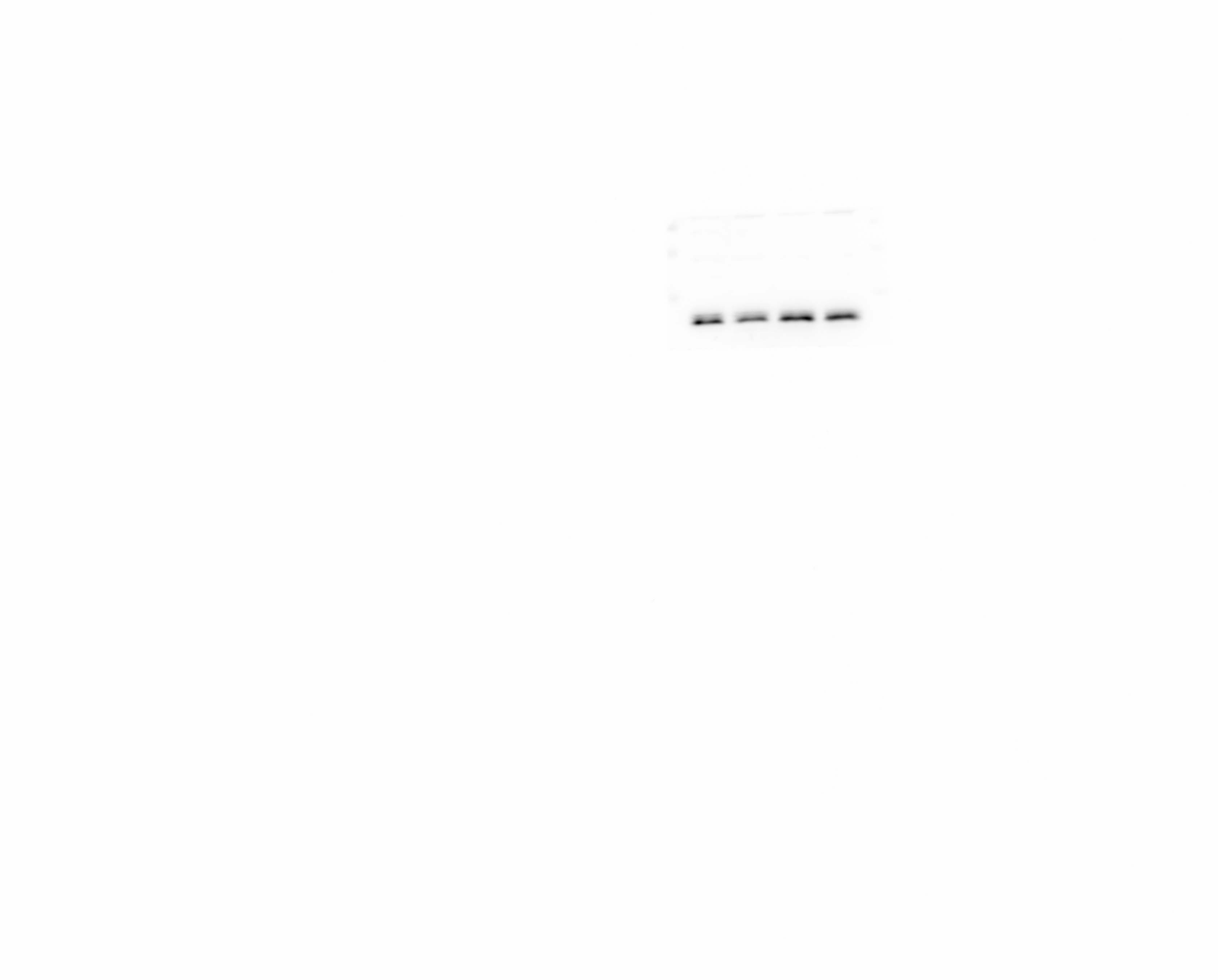

Supplement: Supplementary file 2 — Supporting File 2: advs73976‐sup‐0002‐SuppMat.zip. [file ADVS-13-e11217-s002.zip › WB#U4ee3#U8868#U56fe/xiap#U539f#U59cb#U6570#U636ewb2-JPEG/dk_4#U4ee3#U8868 mg.jpg]

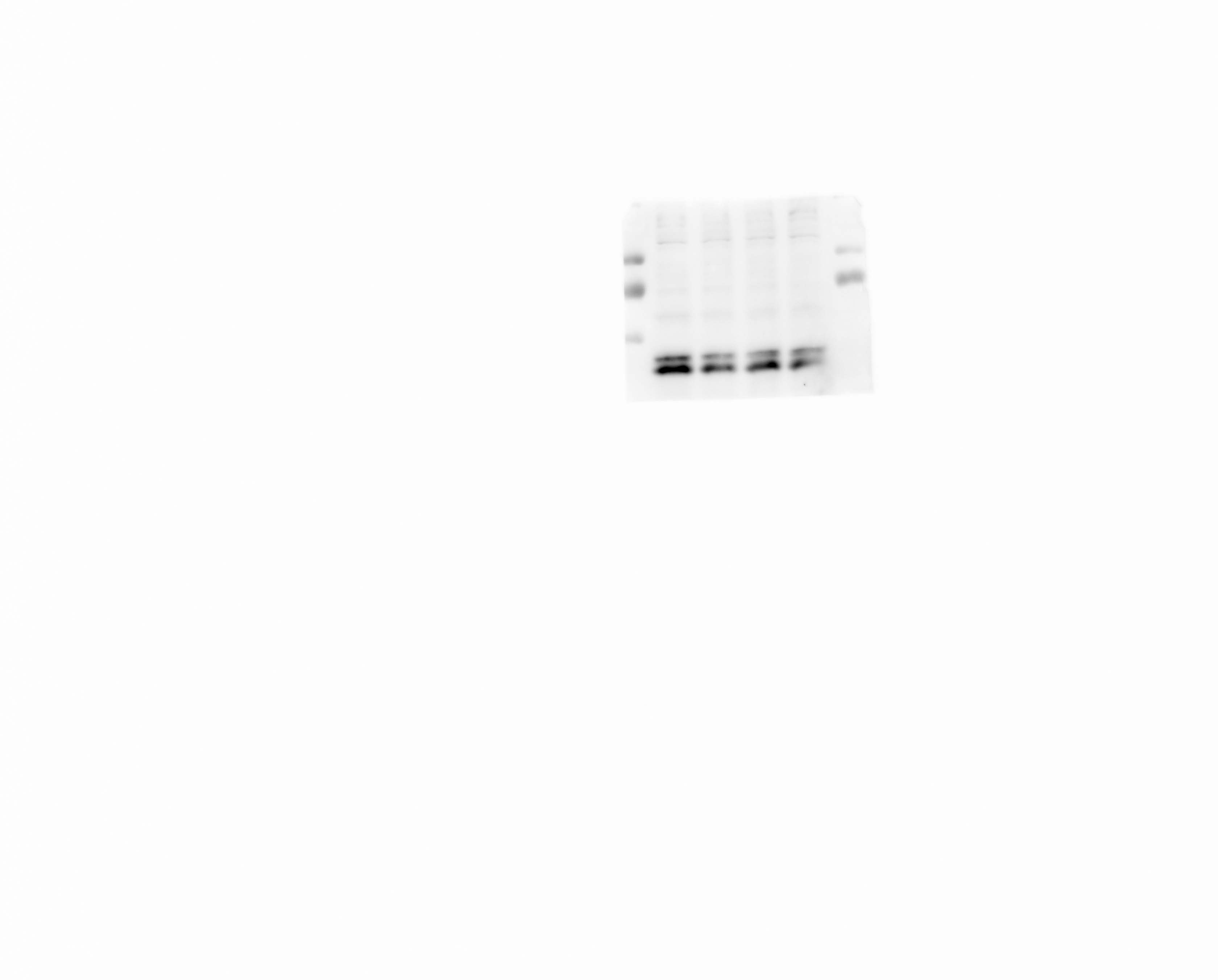

Supplement: Supplementary file 2 — Supporting File 2: advs73976‐sup‐0002‐SuppMat.zip. [file ADVS-13-e11217-s002.zip › WB#U4ee3#U8868#U56fe/xiap#U539f#U59cb#U6570#U636ewb2-JPEG/dk_5 six.jpg]

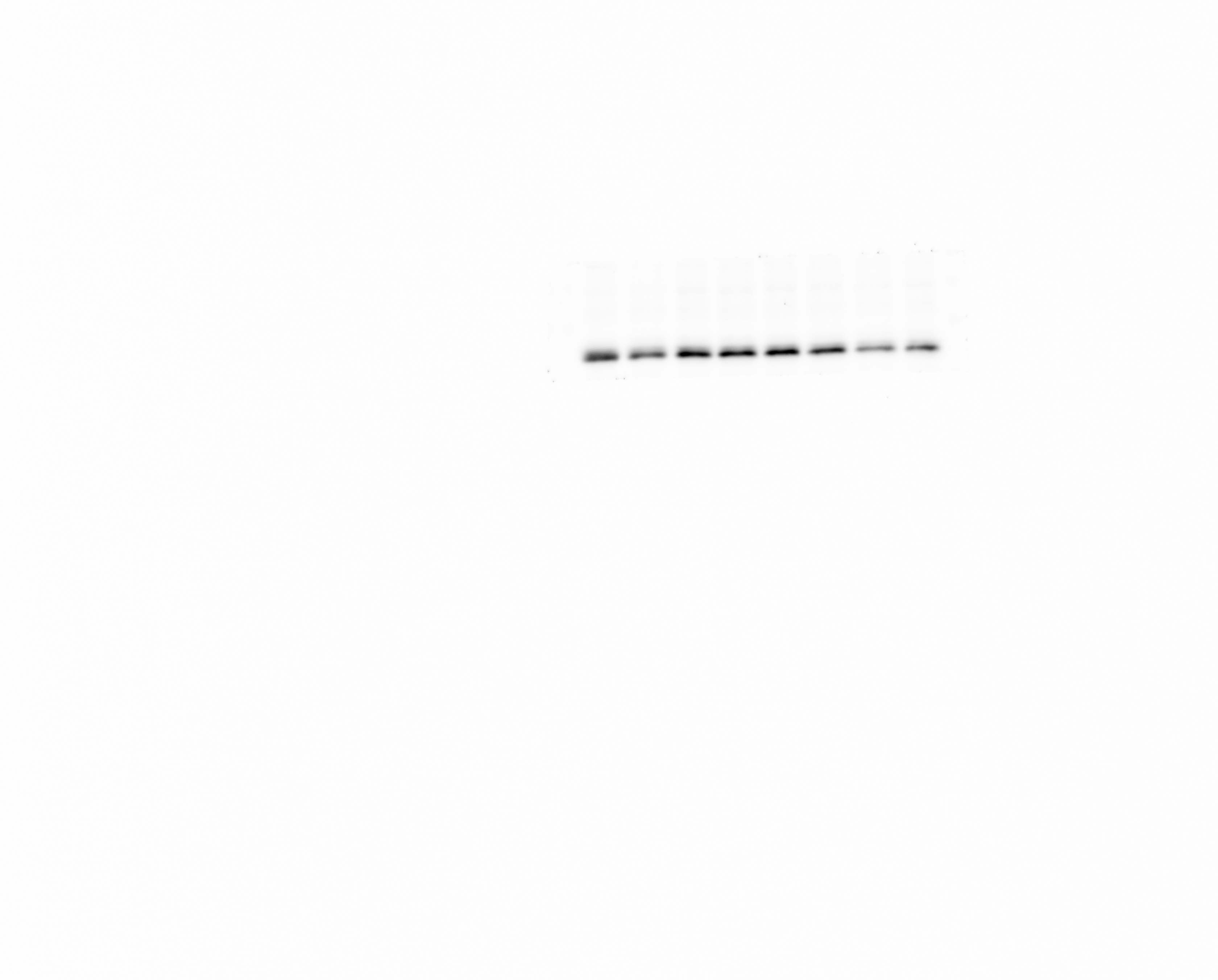

Supplement: Supplementary file 2 — Supporting File 2: advs73976‐sup‐0002‐SuppMat.zip. [file ADVS-13-e11217-s002.zip › WB#U4ee3#U8868#U56fe/xiap#U539f#U59cb#U6570#U636ewb2-JPEG/DK_6 mut xiap#U4ee3#U8868.jpg]

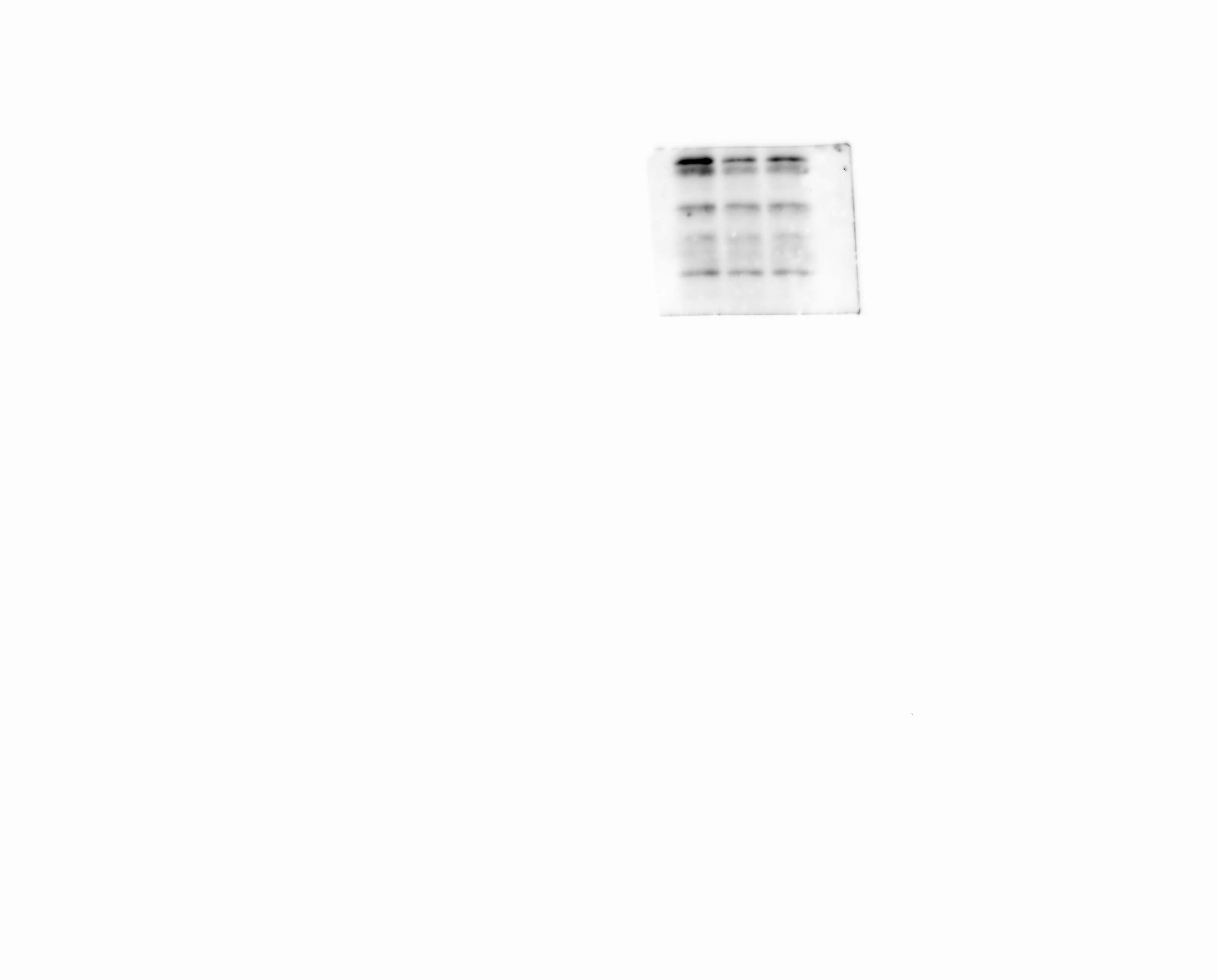

Supplement: Supplementary file 2 — Supporting File 2: advs73976‐sup‐0002‐SuppMat.zip. [file ADVS-13-e11217-s002.zip › WB#U4ee3#U8868#U56fe/xiap#U539f#U59cb#U6570#U636ewb2-JPEG/DK_6 DB GAS.jpg]
