# Supplementary figures and images for: XIAP Stabilizes DDRGK1 to Promote ER‐Phagy and Protects Against Noise‐Induced Hearing Loss (part 1 of 2)
Source: Adv Sci (Weinh). 2026 Jan 26;13(18):e11217. doi: 10.1002/advs.202511217 (PMC13042907; doi:10.1002/advs.202511217)

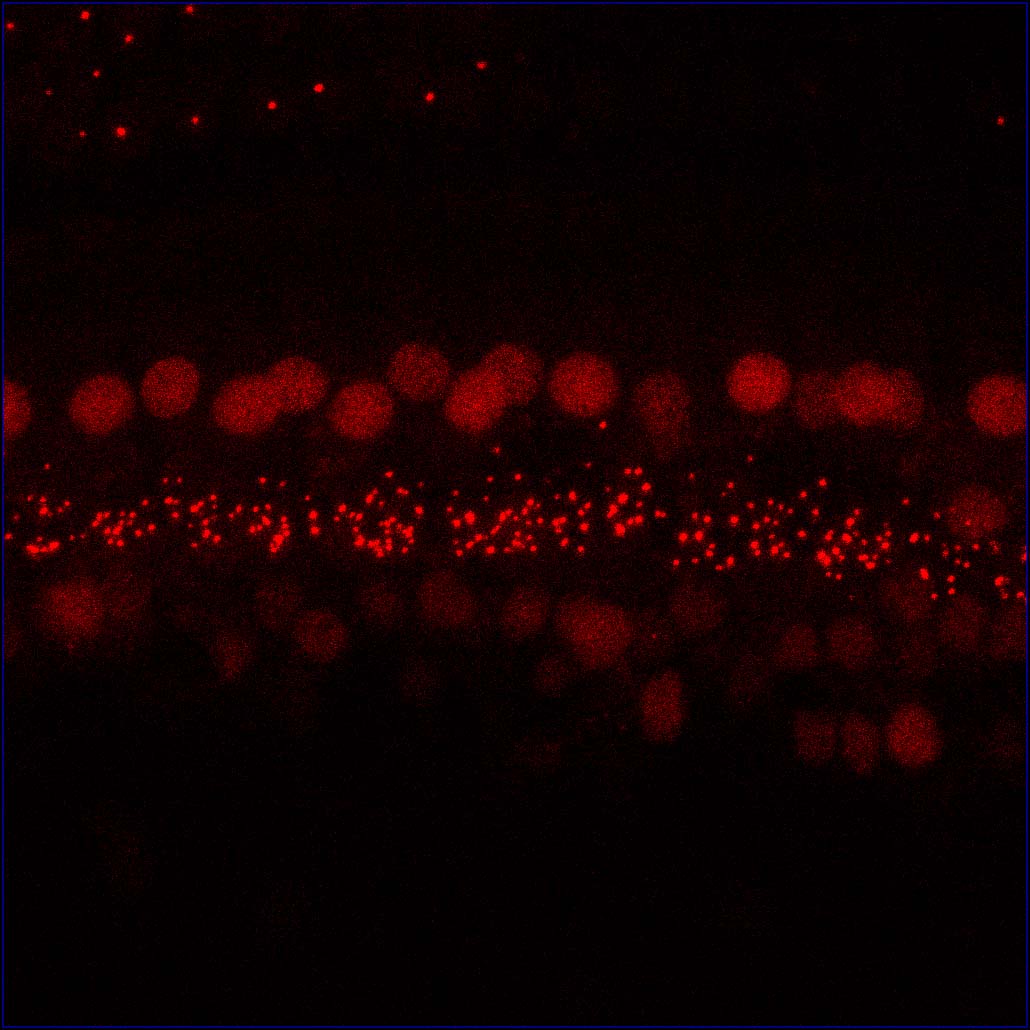

Supplement: Supplementary file 2 — Supporting File 2: advs73976‐sup‐0002‐SuppMat.zip. [file ADVS-13-e11217-s002.zip › ctbp2-JPEG/A GAS.jpg]

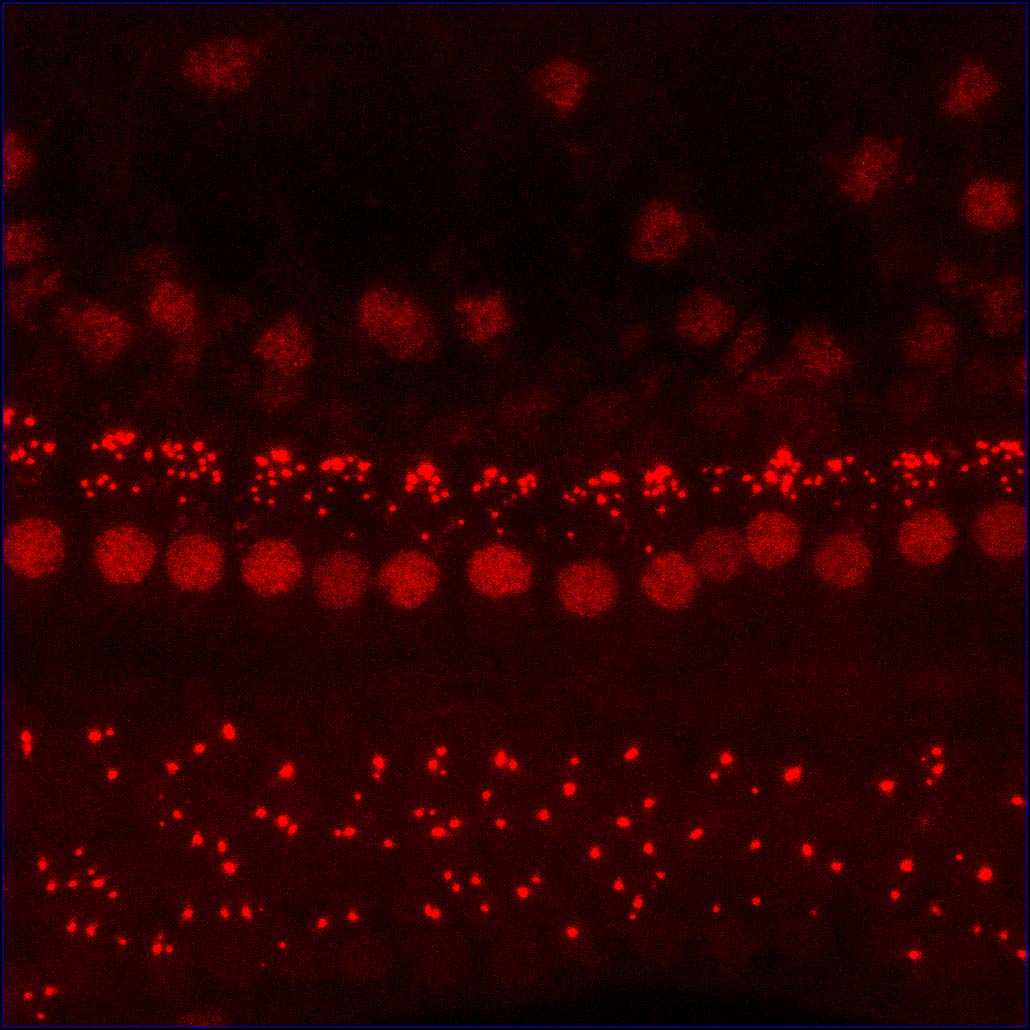

Supplement: Supplementary file 2 — Supporting File 2: advs73976‐sup‐0002‐SuppMat.zip. [file ADVS-13-e11217-s002.zip › ctbp2-JPEG/A N+G.jpg]

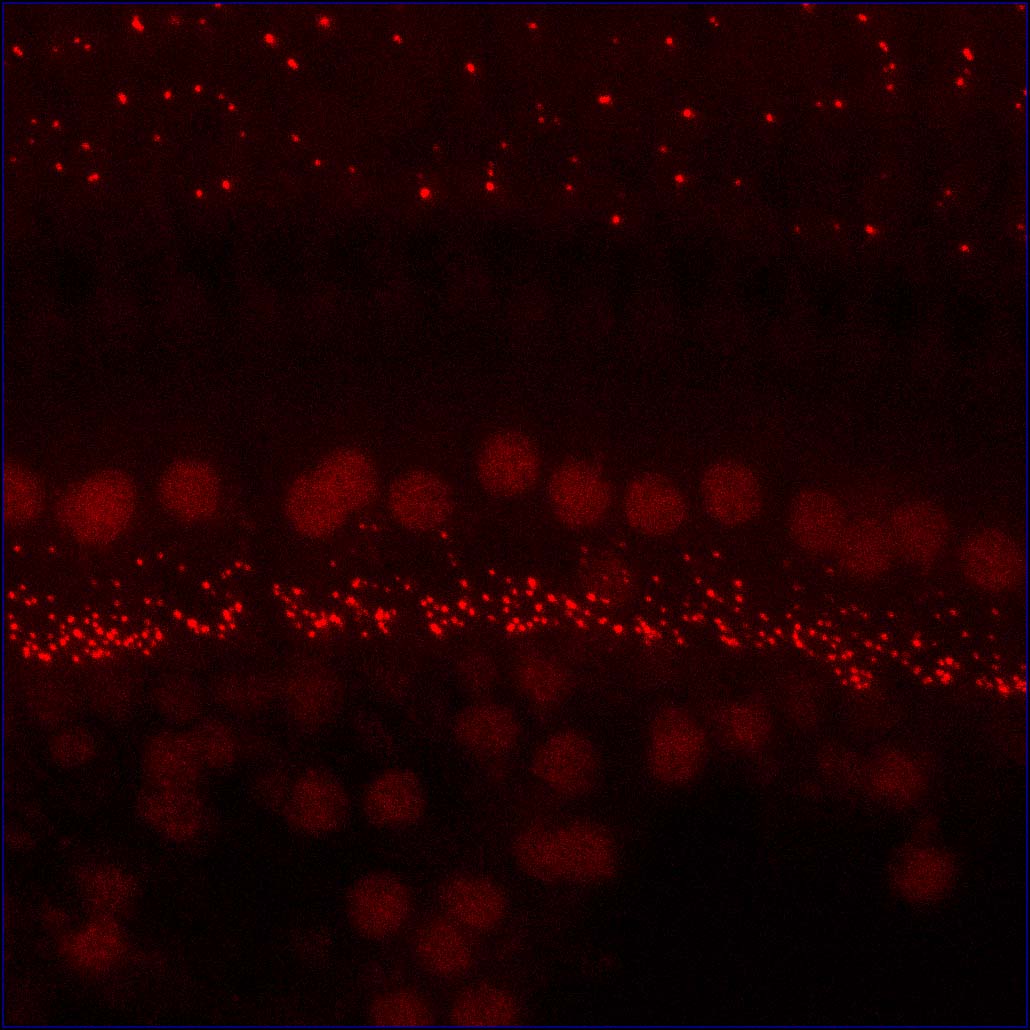

Supplement: Supplementary file 2 — Supporting File 2: advs73976‐sup‐0002‐SuppMat.zip. [file ADVS-13-e11217-s002.zip › ctbp2-JPEG/A CT M2.jpg]

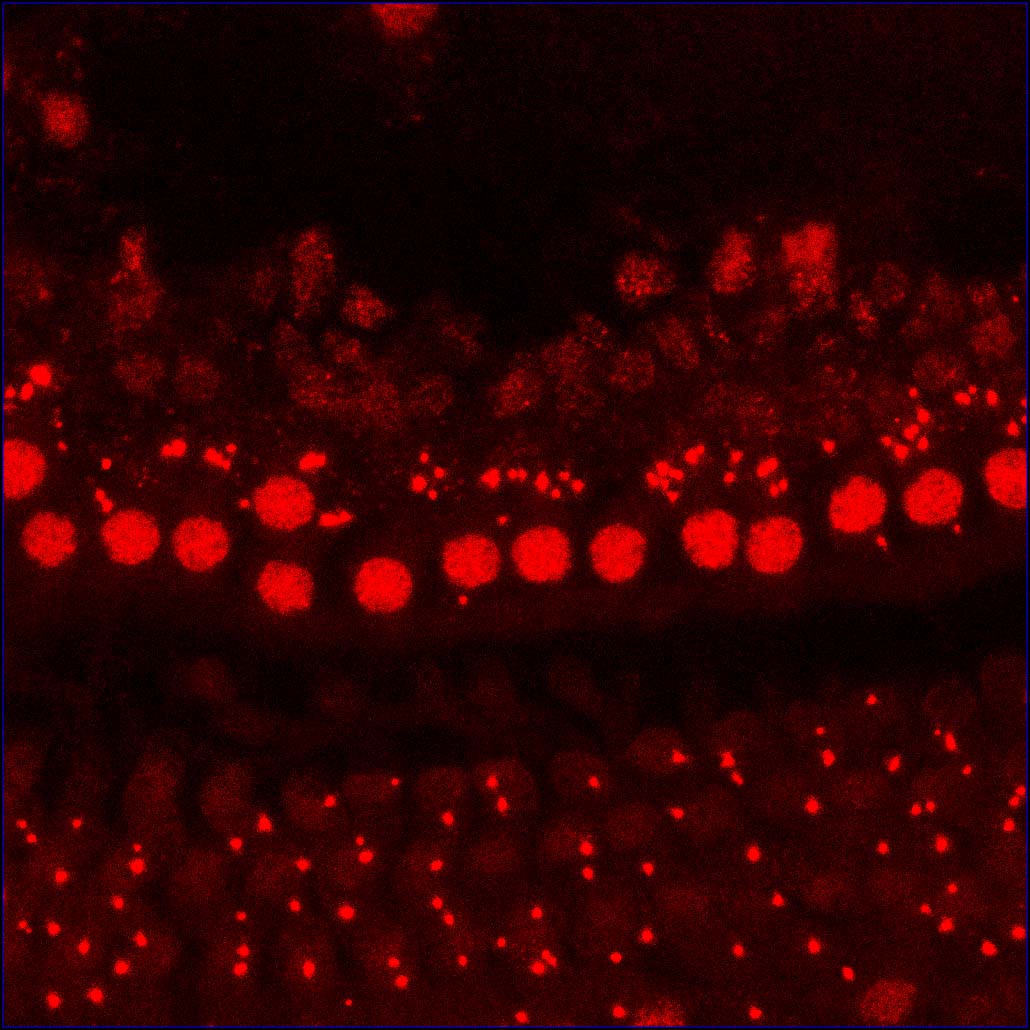

Supplement: Supplementary file 2 — Supporting File 2: advs73976‐sup‐0002‐SuppMat.zip. [file ADVS-13-e11217-s002.zip › ctbp2-JPEG/A3 N.jpg]

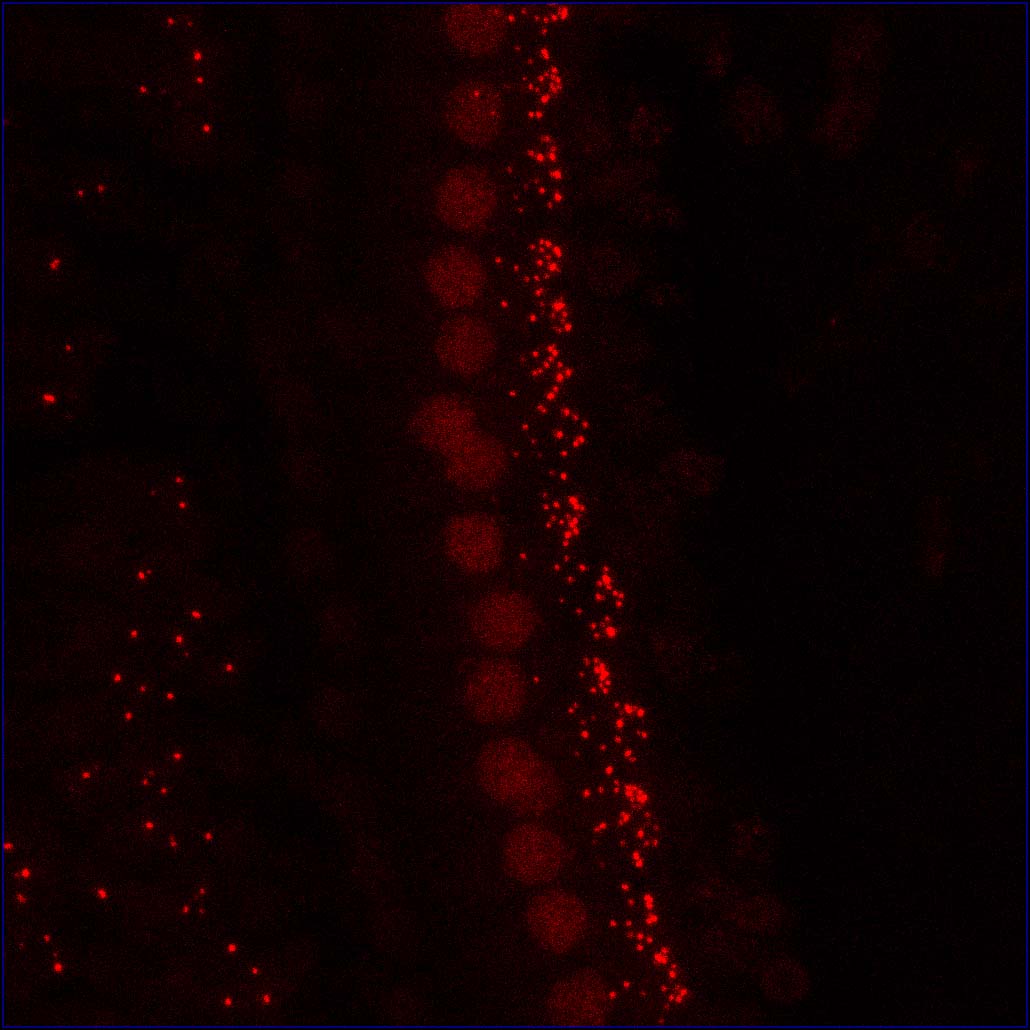

Supplement: Supplementary file 2 — Supporting File 2: advs73976‐sup‐0002‐SuppMat.zip. [file ADVS-13-e11217-s002.zip › ctbp2-JPEG/B CT.jpg]

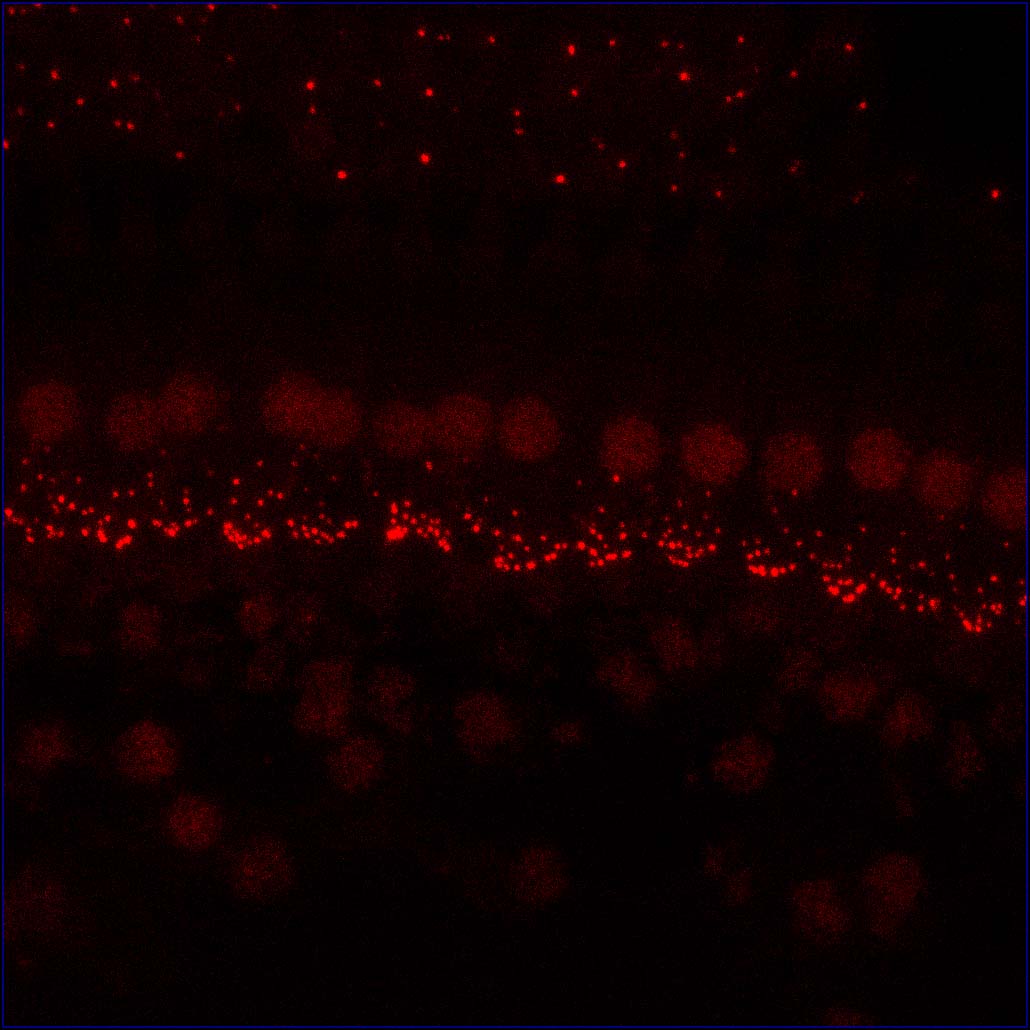

Supplement: Supplementary file 2 — Supporting File 2: advs73976‐sup‐0002‐SuppMat.zip. [file ADVS-13-e11217-s002.zip › ctbp2-JPEG/B GAS.jpg]

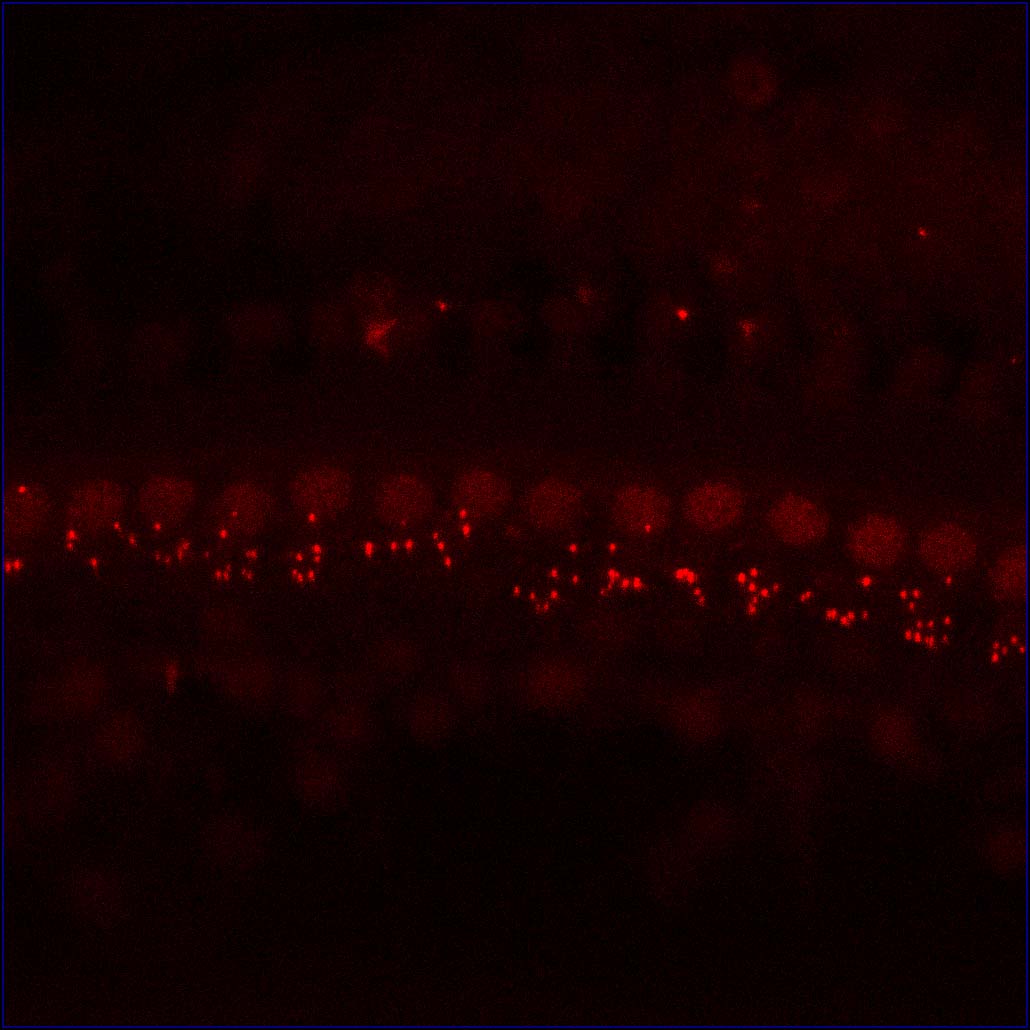

Supplement: Supplementary file 2 — Supporting File 2: advs73976‐sup‐0002‐SuppMat.zip. [file ADVS-13-e11217-s002.zip › ctbp2-JPEG/B4.jpg]

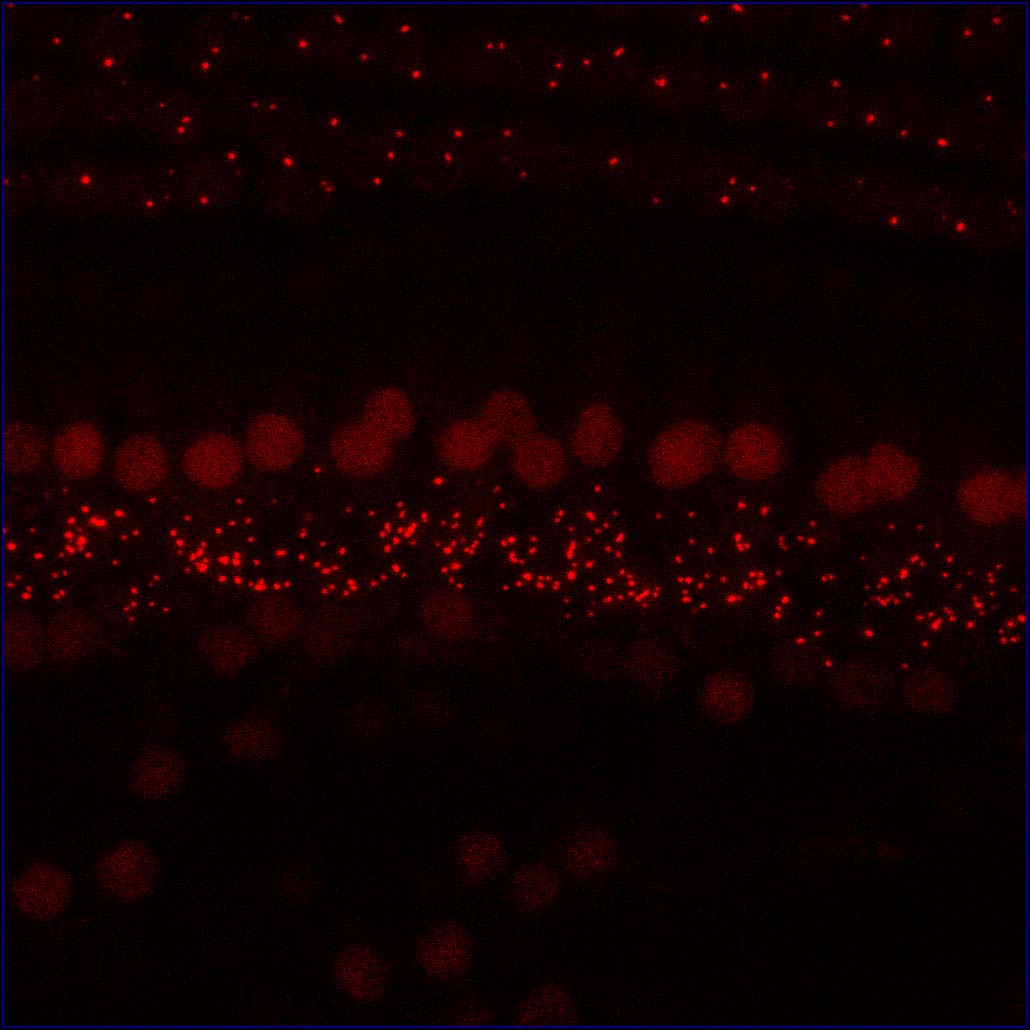

Supplement: Supplementary file 2 — Supporting File 2: advs73976‐sup‐0002‐SuppMat.zip. [file ADVS-13-e11217-s002.zip › ctbp2-JPEG/M CT.jpg]

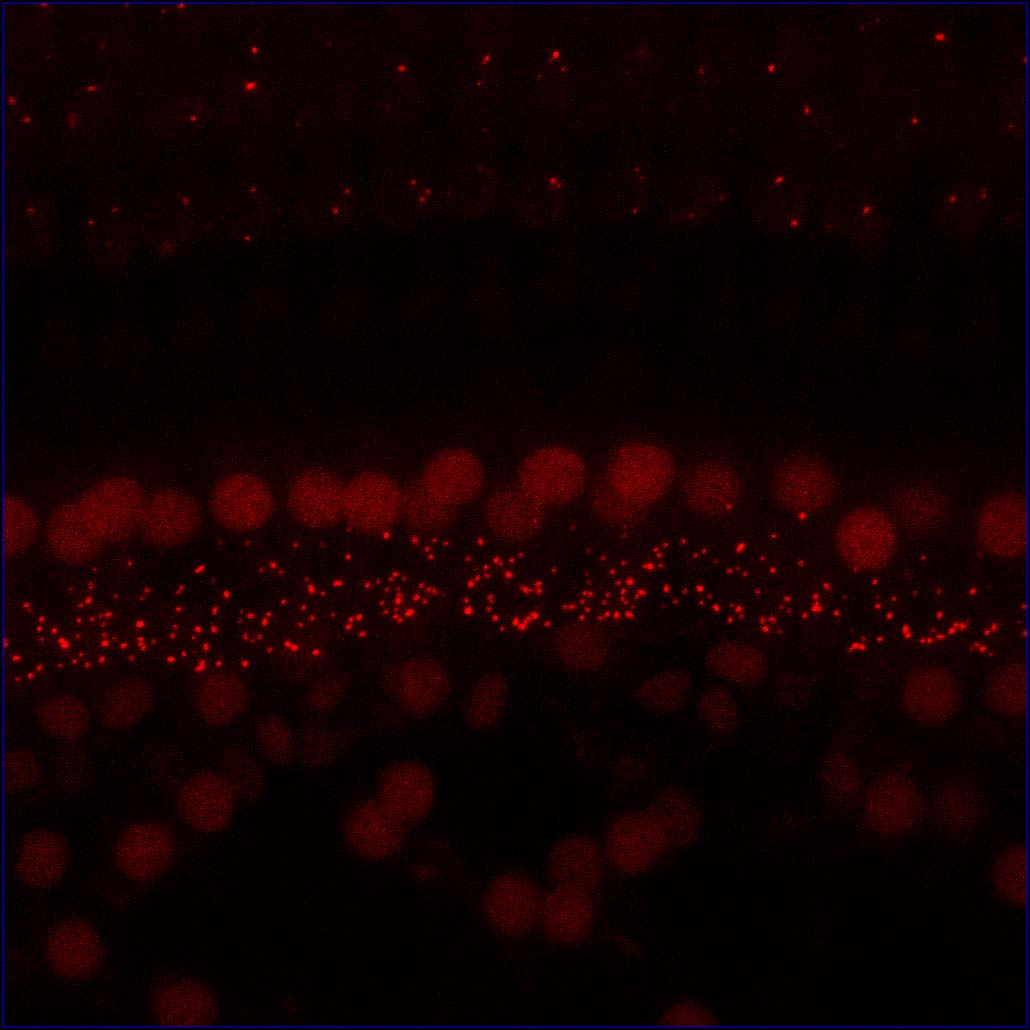

Supplement: Supplementary file 2 — Supporting File 2: advs73976‐sup‐0002‐SuppMat.zip. [file ADVS-13-e11217-s002.zip › ctbp2-JPEG/M GAS.jpg]

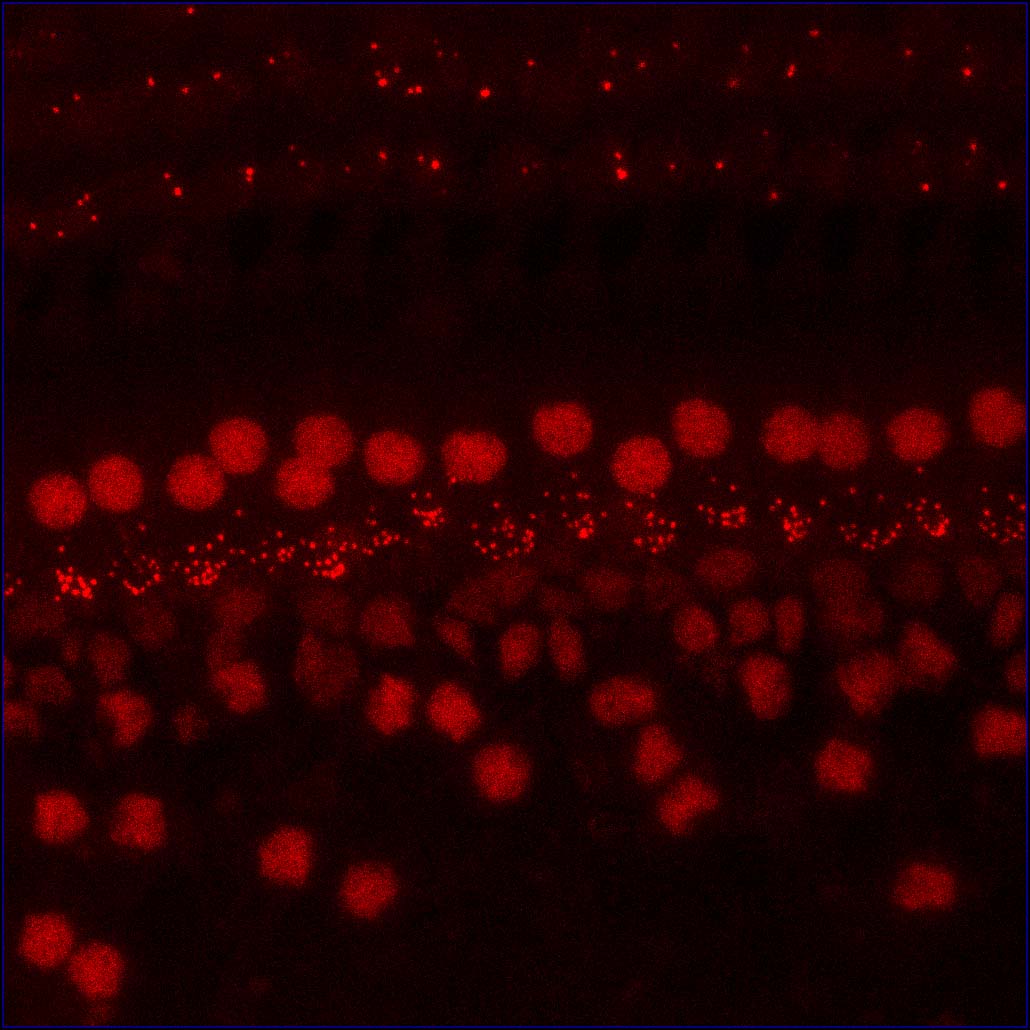

Supplement: Supplementary file 2 — Supporting File 2: advs73976‐sup‐0002‐SuppMat.zip. [file ADVS-13-e11217-s002.zip › ctbp2-JPEG/M N+G.jpg]

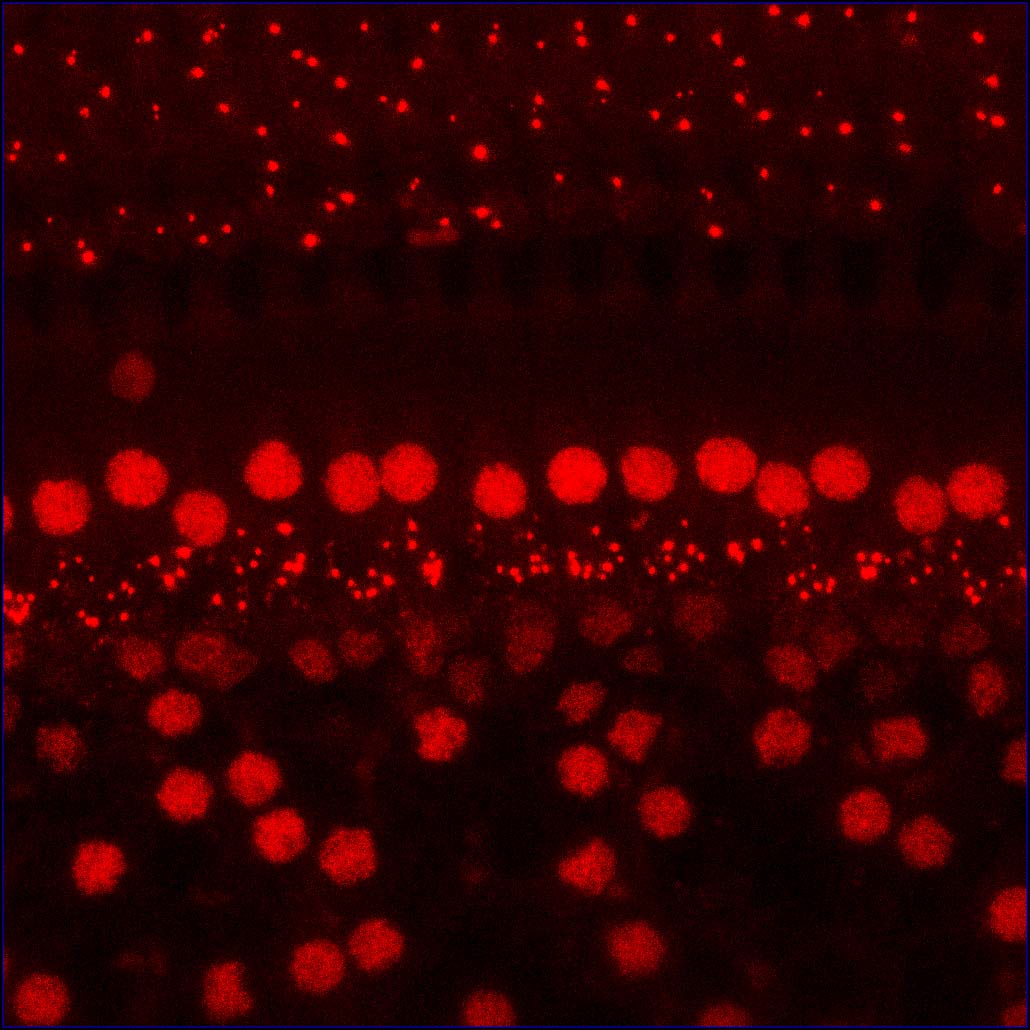

Supplement: Supplementary file 2 — Supporting File 2: advs73976‐sup‐0002‐SuppMat.zip. [file ADVS-13-e11217-s002.zip › ctbp2-JPEG/M8 N.jpg]

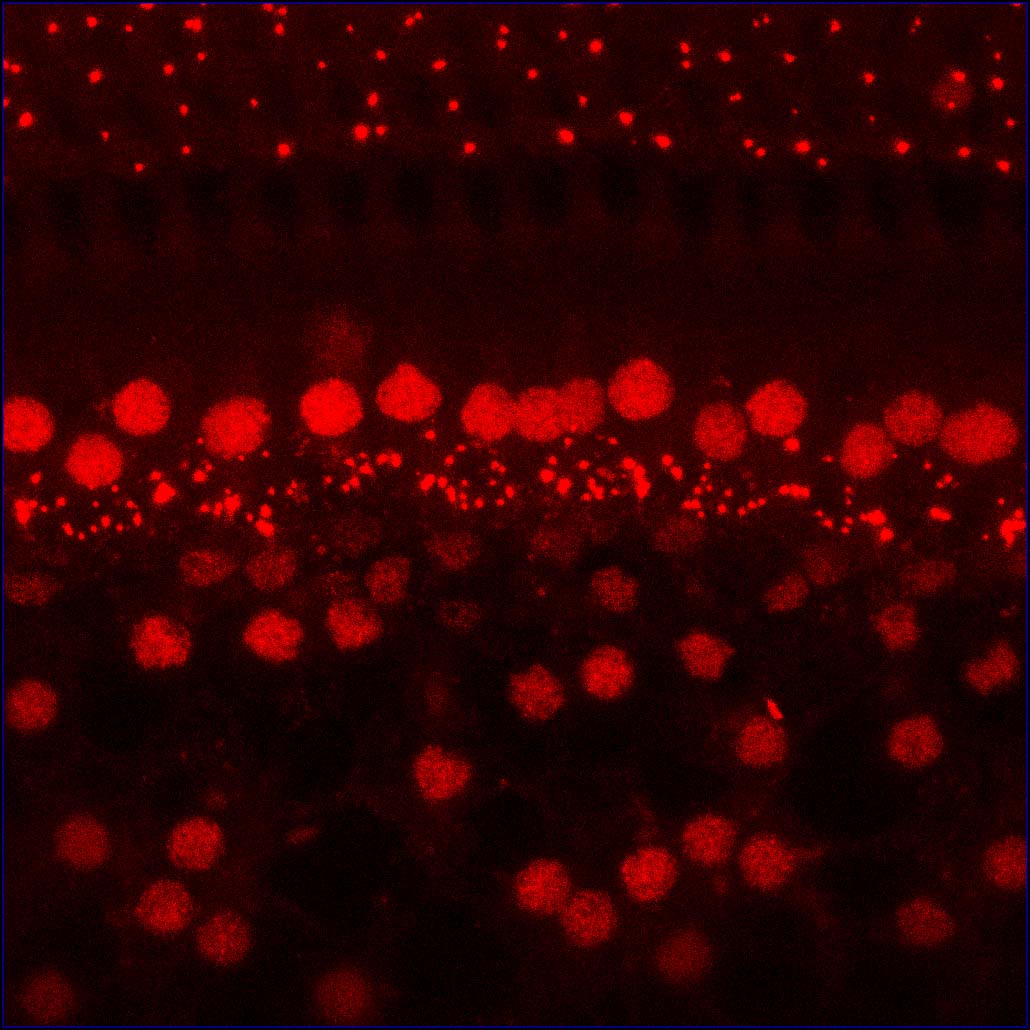

Supplement: Supplementary file 2 — Supporting File 2: advs73976‐sup‐0002‐SuppMat.zip. [file ADVS-13-e11217-s002.zip › ctbp2-JPEG/M9 B N+G.jpg]

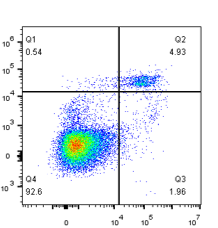

Supplement: Supplementary file 2 — Supporting File 2: advs73976‐sup‐0002‐SuppMat.zip. [file ADVS-13-e11217-s002.zip › FACS/Annexin V-PI/S1-A/12h.png]

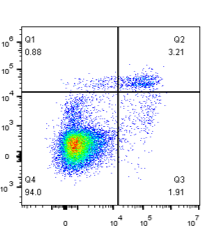

Supplement: Supplementary file 2 — Supporting File 2: advs73976‐sup‐0002‐SuppMat.zip. [file ADVS-13-e11217-s002.zip › FACS/Annexin V-PI/S1-A/1h.png]

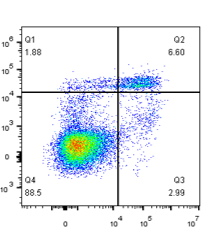

Supplement: Supplementary file 2 — Supporting File 2: advs73976‐sup‐0002‐SuppMat.zip. [file ADVS-13-e11217-s002.zip › FACS/Annexin V-PI/S1-A/24h.png]

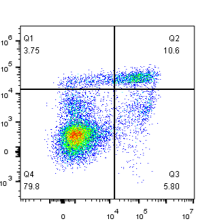

Supplement: Supplementary file 2 — Supporting File 2: advs73976‐sup‐0002‐SuppMat.zip. [file ADVS-13-e11217-s002.zip › FACS/Annexin V-PI/S1-A/H2O2-12h.png]

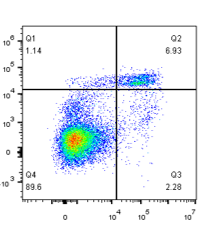

Supplement: Supplementary file 2 — Supporting File 2: advs73976‐sup‐0002‐SuppMat.zip. [file ADVS-13-e11217-s002.zip › FACS/Annexin V-PI/S1-A/H2O2-1h.png]

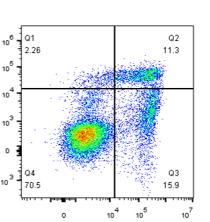

Supplement: Supplementary file 2 — Supporting File 2: advs73976‐sup‐0002‐SuppMat.zip. [file ADVS-13-e11217-s002.zip › FACS/Annexin V-PI/S1-A/H2O2-24h.png]

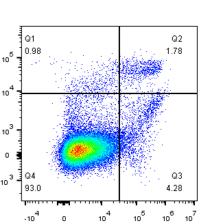

Supplement: Supplementary file 2 — Supporting File 2: advs73976‐sup‐0002‐SuppMat.zip. [file ADVS-13-e11217-s002.zip › FACS/Annexin V-PI/S1-M/Control-C.png]

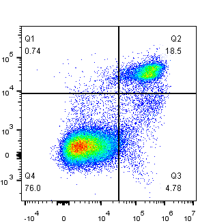

Supplement: Supplementary file 2 — Supporting File 2: advs73976‐sup‐0002‐SuppMat.zip. [file ADVS-13-e11217-s002.zip › FACS/Annexin V-PI/S1-M/Control-H.png]

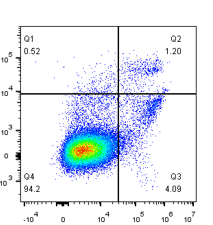

Supplement: Supplementary file 2 — Supporting File 2: advs73976‐sup‐0002‐SuppMat.zip. [file ADVS-13-e11217-s002.zip › FACS/Annexin V-PI/S1-M/XIAP-C.png]

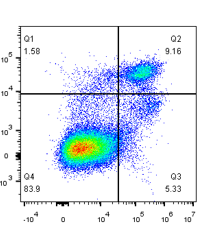

Supplement: Supplementary file 2 — Supporting File 2: advs73976‐sup‐0002‐SuppMat.zip. [file ADVS-13-e11217-s002.zip › FACS/Annexin V-PI/S1-M/XIAP-H.png]

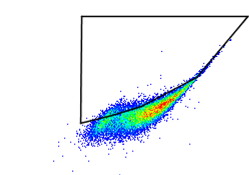

Supplement: Supplementary file 2 — Supporting File 2: advs73976‐sup‐0002‐SuppMat.zip. [file ADVS-13-e11217-s002.zip › FACS/KDEL-RAMP4-FACS/KDEL-12h-H2O2.png]

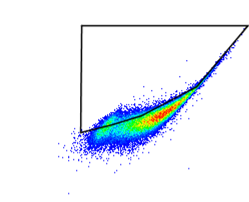

Supplement: Supplementary file 2 — Supporting File 2: advs73976‐sup‐0002‐SuppMat.zip. [file ADVS-13-e11217-s002.zip › FACS/KDEL-RAMP4-FACS/KDEL-1h-C.png]

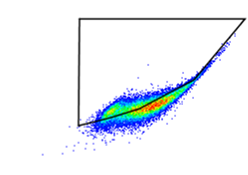

Supplement: Supplementary file 2 — Supporting File 2: advs73976‐sup‐0002‐SuppMat.zip. [file ADVS-13-e11217-s002.zip › FACS/KDEL-RAMP4-FACS/KDEL-1h-H2O2.png]

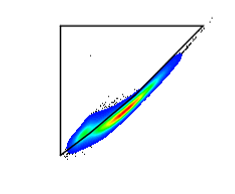

Supplement: Supplementary file 2 — Supporting File 2: advs73976‐sup‐0002‐SuppMat.zip. [file ADVS-13-e11217-s002.zip › FACS/KDEL-RAMP4-FACS/KDEL-Ctrl-C-1.png]

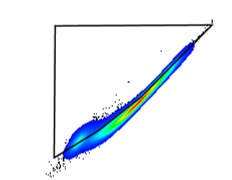

Supplement: Supplementary file 2 — Supporting File 2: advs73976‐sup‐0002‐SuppMat.zip. [file ADVS-13-e11217-s002.zip › FACS/KDEL-RAMP4-FACS/KDEL-Ctrl-C-2.png]

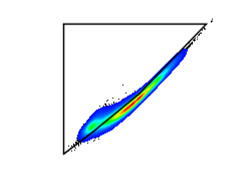

Supplement: Supplementary file 2 — Supporting File 2: advs73976‐sup‐0002‐SuppMat.zip. [file ADVS-13-e11217-s002.zip › FACS/KDEL-RAMP4-FACS/KDEL-Ctrl-H2O2-1.png]

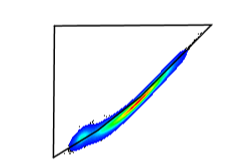

Supplement: Supplementary file 2 — Supporting File 2: advs73976‐sup‐0002‐SuppMat.zip. [file ADVS-13-e11217-s002.zip › FACS/KDEL-RAMP4-FACS/KDEL-Ctrl-H2O2-2.png]

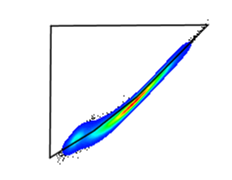

Supplement: Supplementary file 2 — Supporting File 2: advs73976‐sup‐0002‐SuppMat.zip. [file ADVS-13-e11217-s002.zip › FACS/KDEL-RAMP4-FACS/KDEL-DDRGK1-C-1.png]

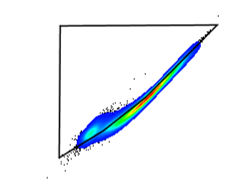

Supplement: Supplementary file 2 — Supporting File 2: advs73976‐sup‐0002‐SuppMat.zip. [file ADVS-13-e11217-s002.zip › FACS/KDEL-RAMP4-FACS/KDEL-DDRGK1-H2O2.png]

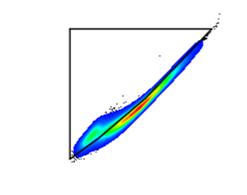

Supplement: Supplementary file 2 — Supporting File 2: advs73976‐sup‐0002‐SuppMat.zip. [file ADVS-13-e11217-s002.zip › FACS/KDEL-RAMP4-FACS/KDEL-XIAP-C-1.png]

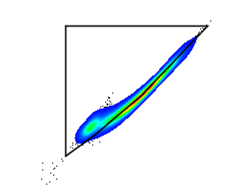

Supplement: Supplementary file 2 — Supporting File 2: advs73976‐sup‐0002‐SuppMat.zip. [file ADVS-13-e11217-s002.zip › FACS/KDEL-RAMP4-FACS/KDEL-XIAP-H2O2.png]

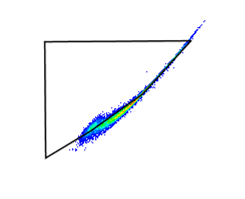

Supplement: Supplementary file 2 — Supporting File 2: advs73976‐sup‐0002‐SuppMat.zip. [file ADVS-13-e11217-s002.zip › FACS/KDEL-RAMP4-FACS/RAMP4-12h-H2O2.png]

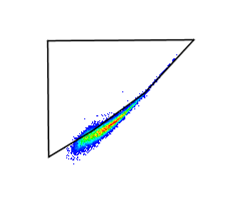

Supplement: Supplementary file 2 — Supporting File 2: advs73976‐sup‐0002‐SuppMat.zip. [file ADVS-13-e11217-s002.zip › FACS/KDEL-RAMP4-FACS/RAMP4-1h-C.png]

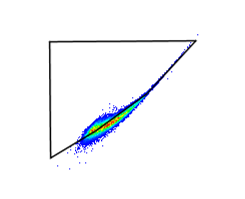

Supplement: Supplementary file 2 — Supporting File 2: advs73976‐sup‐0002‐SuppMat.zip. [file ADVS-13-e11217-s002.zip › FACS/KDEL-RAMP4-FACS/RAMP4-1h-H2O2.png]

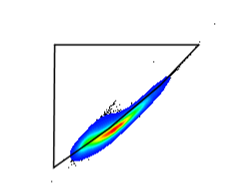

Supplement: Supplementary file 2 — Supporting File 2: advs73976‐sup‐0002‐SuppMat.zip. [file ADVS-13-e11217-s002.zip › FACS/KDEL-RAMP4-FACS/RAMP4-Ctrl-C-.png]

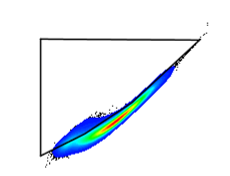

Supplement: Supplementary file 2 — Supporting File 2: advs73976‐sup‐0002‐SuppMat.zip. [file ADVS-13-e11217-s002.zip › FACS/KDEL-RAMP4-FACS/RAMP4-Ctrl-C-2.png]

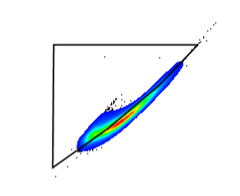

Supplement: Supplementary file 2 — Supporting File 2: advs73976‐sup‐0002‐SuppMat.zip. [file ADVS-13-e11217-s002.zip › FACS/KDEL-RAMP4-FACS/RAMP4-Ctrl-H2O2-1.png]

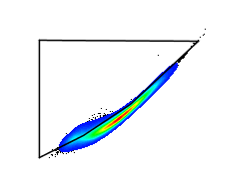

Supplement: Supplementary file 2 — Supporting File 2: advs73976‐sup‐0002‐SuppMat.zip. [file ADVS-13-e11217-s002.zip › FACS/KDEL-RAMP4-FACS/RAMP4-Ctrl-H2O2-2.png]

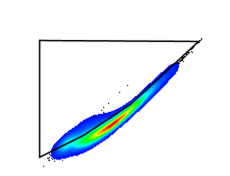

Supplement: Supplementary file 2 — Supporting File 2: advs73976‐sup‐0002‐SuppMat.zip. [file ADVS-13-e11217-s002.zip › FACS/KDEL-RAMP4-FACS/RAMP4-DDRGK1-C-.png]

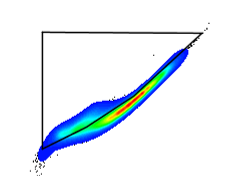

Supplement: Supplementary file 2 — Supporting File 2: advs73976‐sup‐0002‐SuppMat.zip. [file ADVS-13-e11217-s002.zip › FACS/KDEL-RAMP4-FACS/RAMP4-DDRGK1-H2O2-.png]

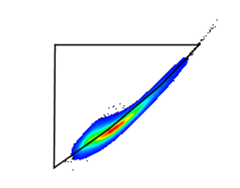

Supplement: Supplementary file 2 — Supporting File 2: advs73976‐sup‐0002‐SuppMat.zip. [file ADVS-13-e11217-s002.zip › FACS/KDEL-RAMP4-FACS/RAMP4-XIAP-C-.png]

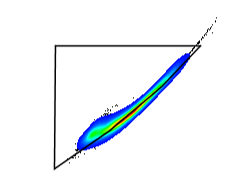

Supplement: Supplementary file 2 — Supporting File 2: advs73976‐sup‐0002‐SuppMat.zip. [file ADVS-13-e11217-s002.zip › FACS/KDEL-RAMP4-FACS/RAMP4-XIAP-H2O2-.png]

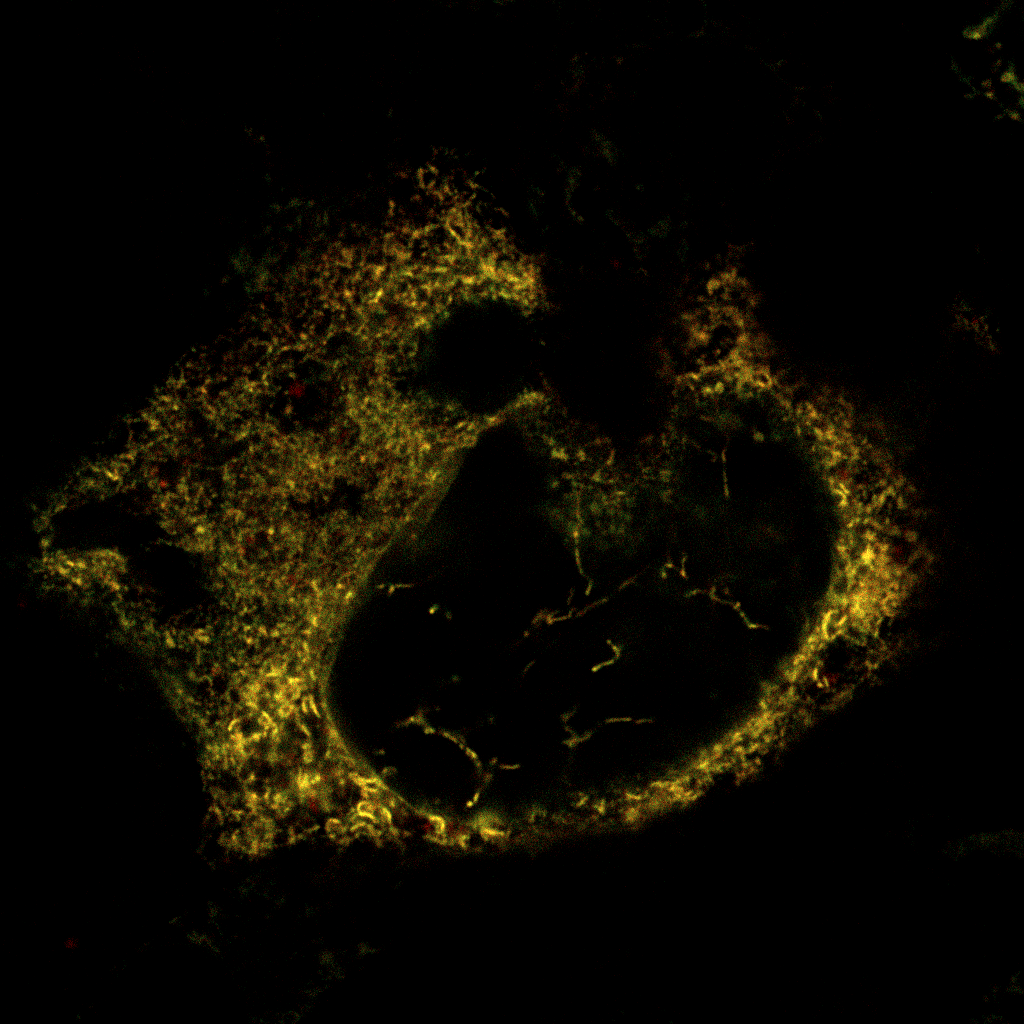

Supplement: Supplementary file 2 — Supporting File 2: advs73976‐sup‐0002‐SuppMat.zip. [file ADVS-13-e11217-s002.zip › KDEL-RAMP4-IF/KDEL-Ctrl-C-3_c1-2.tif]

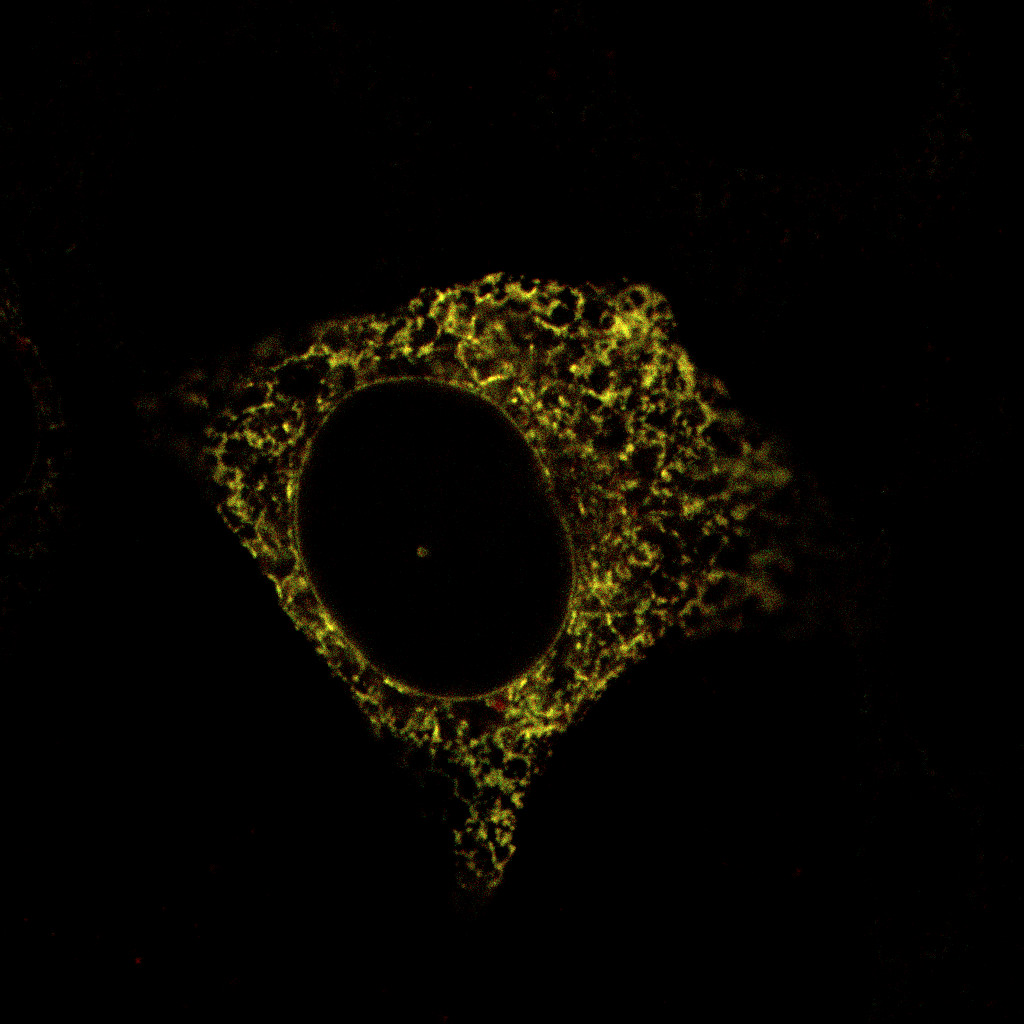

Supplement: Supplementary file 2 — Supporting File 2: advs73976‐sup‐0002‐SuppMat.zip. [file ADVS-13-e11217-s002.zip › KDEL-RAMP4-IF/KDEL-Ctrl-Control-2-adj_c1-2.tif]

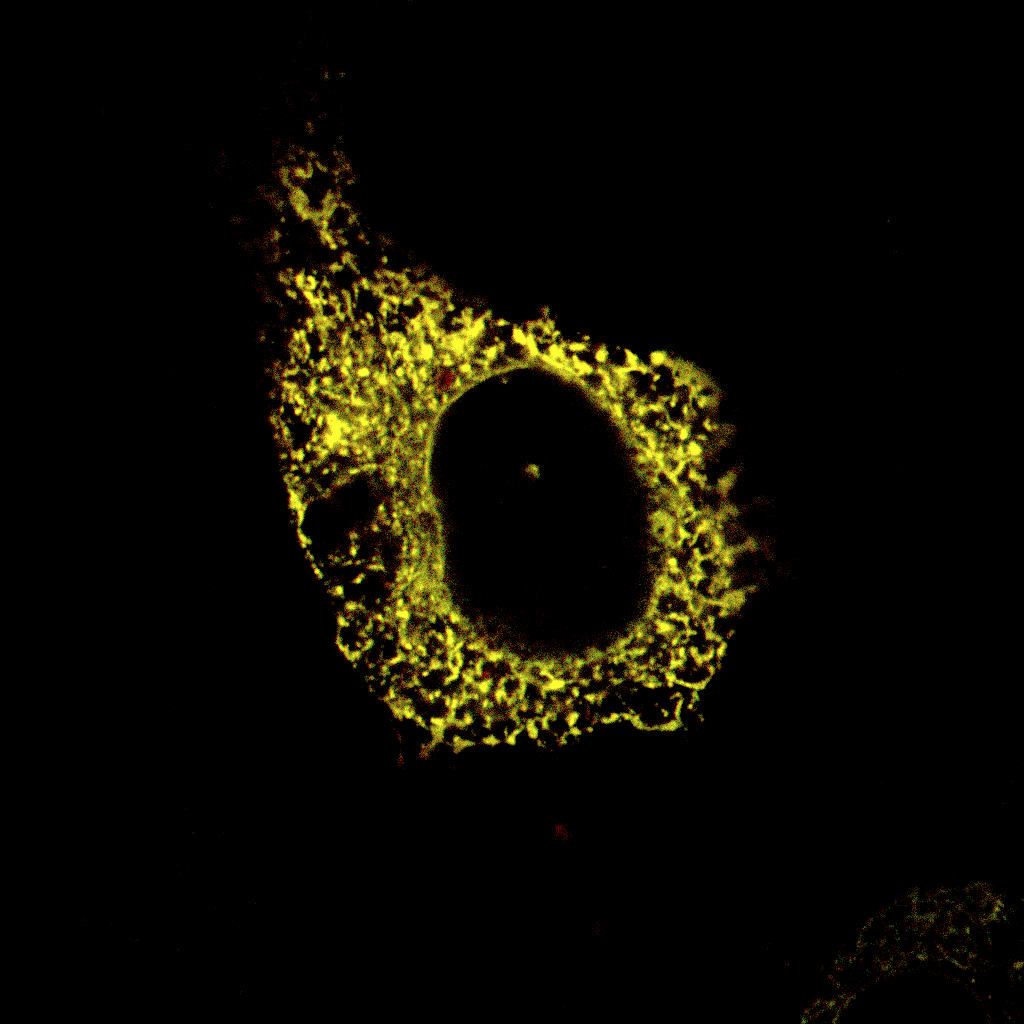

Supplement: Supplementary file 2 — Supporting File 2: advs73976‐sup‐0002‐SuppMat.zip. [file ADVS-13-e11217-s002.zip › KDEL-RAMP4-IF/KDEL-Ctrl-H2O2-3_c1-2.tif]

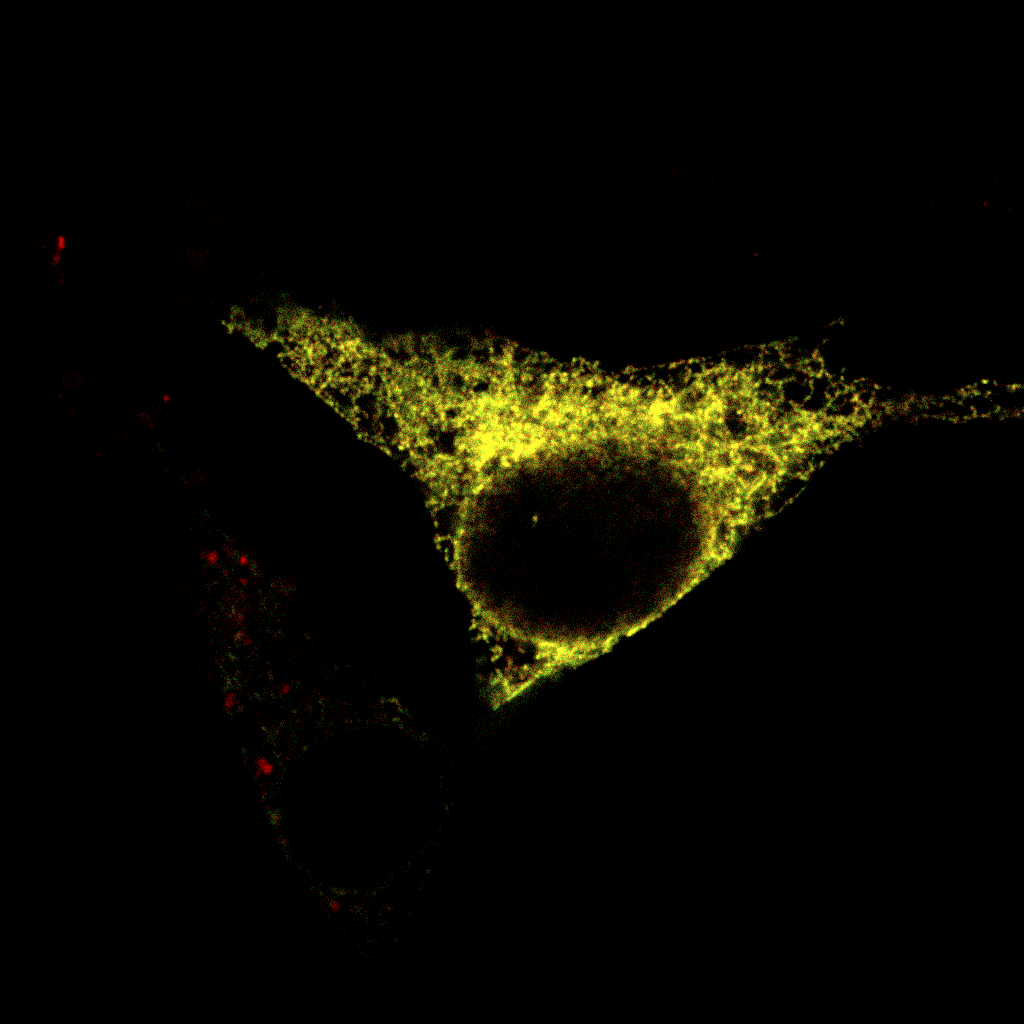

Supplement: Supplementary file 2 — Supporting File 2: advs73976‐sup‐0002‐SuppMat.zip. [file ADVS-13-e11217-s002.zip › KDEL-RAMP4-IF/KDEL-Ctrl-H2O2-6-adj_c1-2.tif]

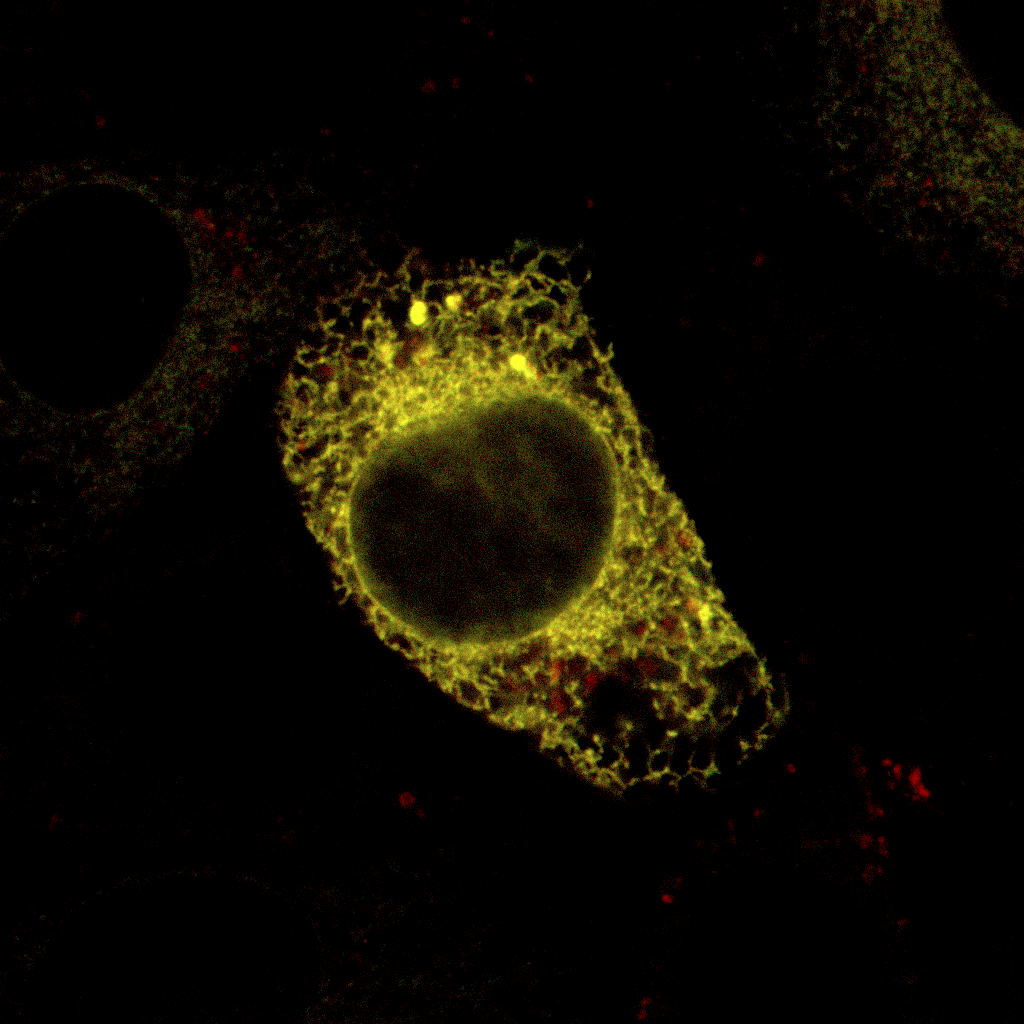

Supplement: Supplementary file 2 — Supporting File 2: advs73976‐sup‐0002‐SuppMat.zip. [file ADVS-13-e11217-s002.zip › KDEL-RAMP4-IF/KDEL-DDRGK1-C-8_c1-2.tif]

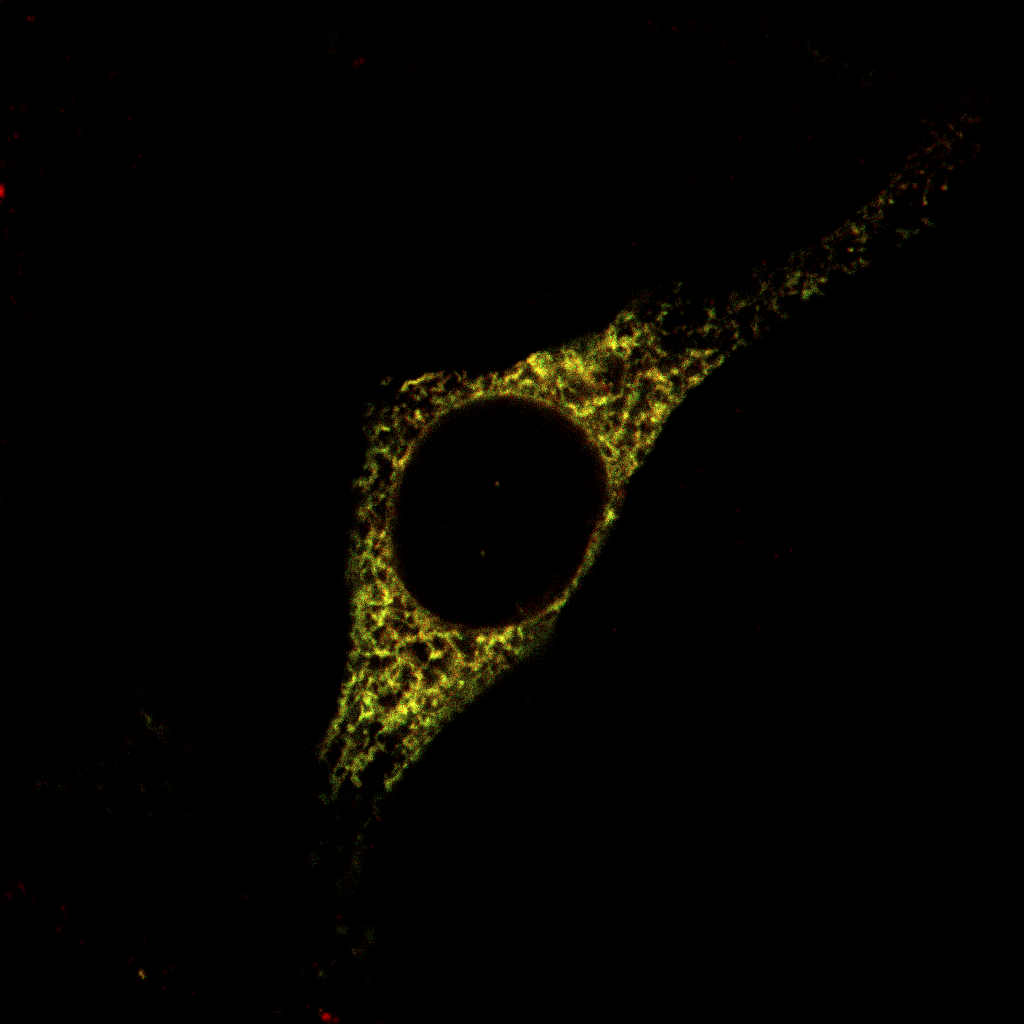

Supplement: Supplementary file 2 — Supporting File 2: advs73976‐sup‐0002‐SuppMat.zip. [file ADVS-13-e11217-s002.zip › KDEL-RAMP4-IF/KDEL-XIAP-Control-3-adj_c1-2.tif]

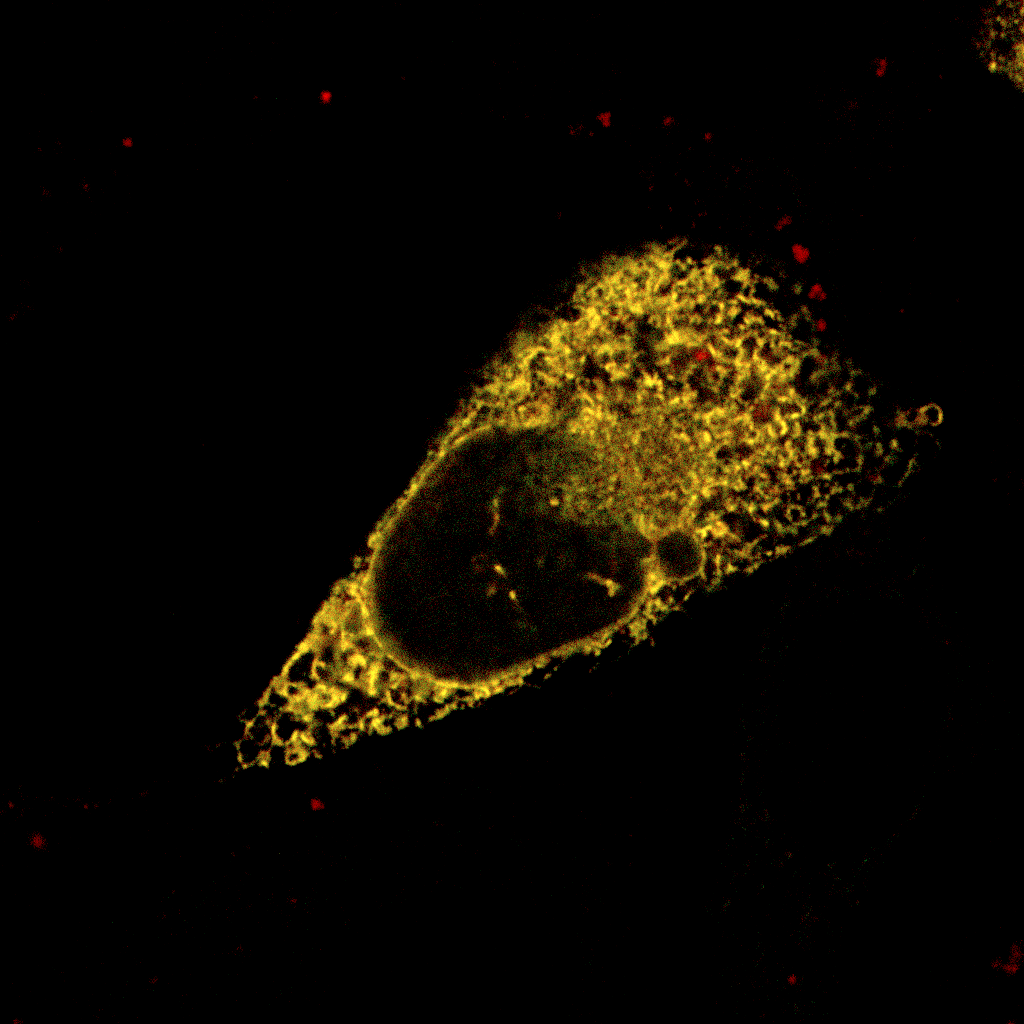

Supplement: Supplementary file 2 — Supporting File 2: advs73976‐sup‐0002‐SuppMat.zip. [file ADVS-13-e11217-s002.zip › KDEL-RAMP4-IF/KDEL-XIAPH2O2-4-adj_c1-2.tif]

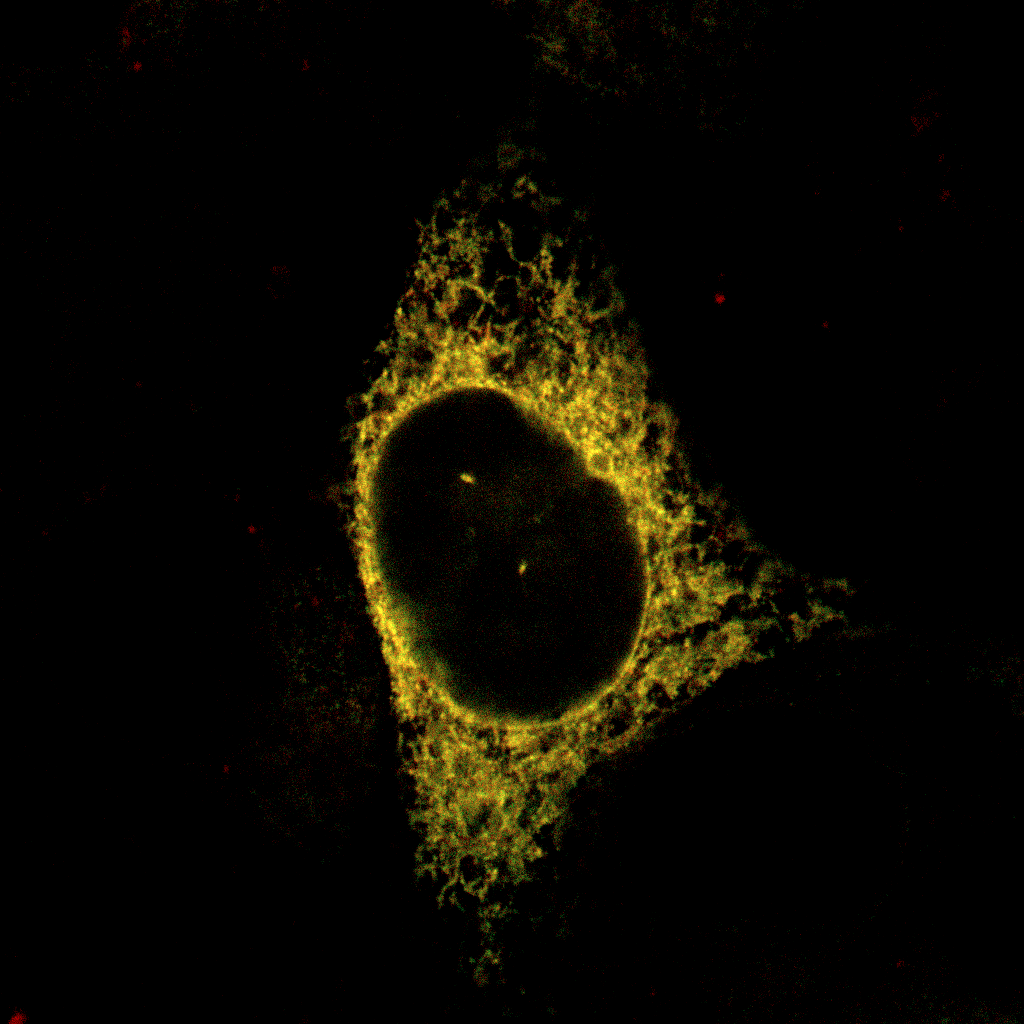

Supplement: Supplementary file 2 — Supporting File 2: advs73976‐sup‐0002‐SuppMat.zip. [file ADVS-13-e11217-s002.zip › KDEL-RAMP4-IF/RAMP4-Ctrl-C-5_c1-2.tif]

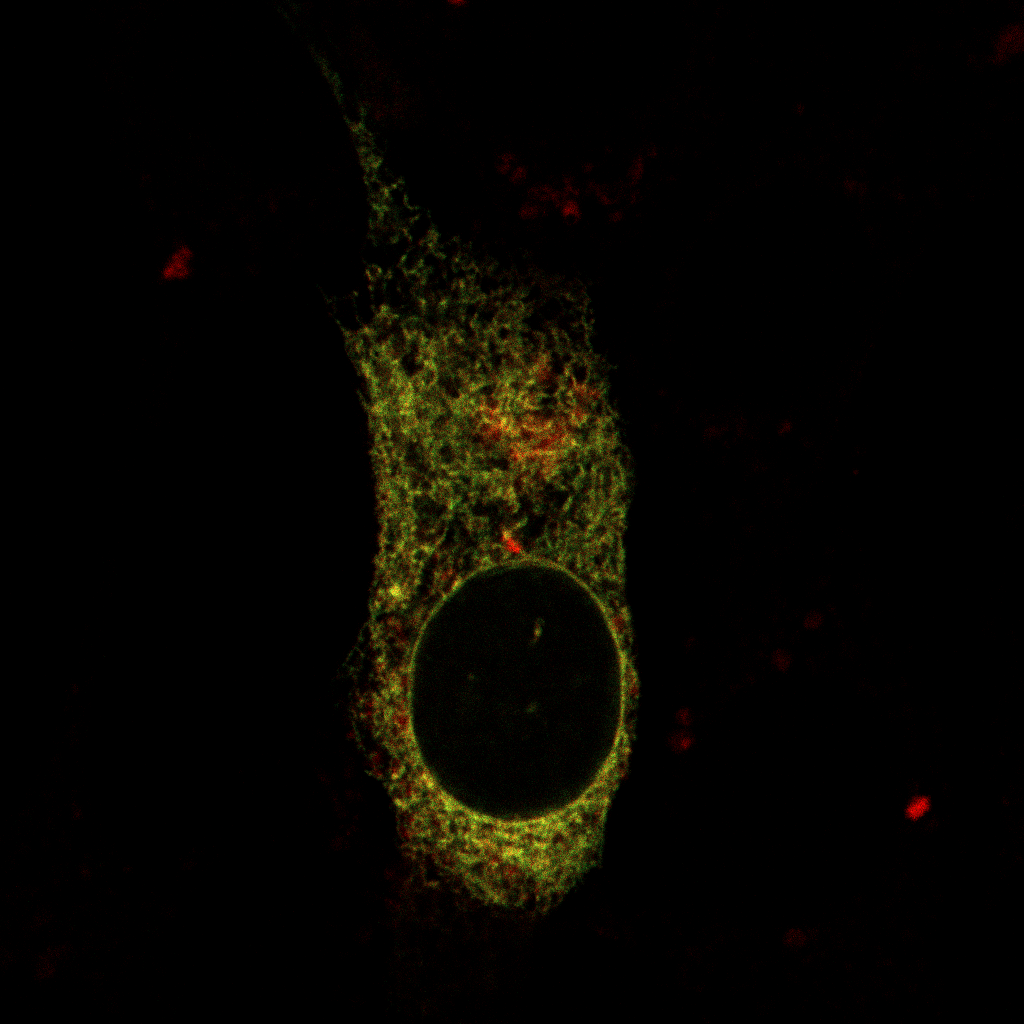

Supplement: Supplementary file 2 — Supporting File 2: advs73976‐sup‐0002‐SuppMat.zip. [file ADVS-13-e11217-s002.zip › KDEL-RAMP4-IF/RAMP4-Ctrl-control-3-adj_c1-2.tif]

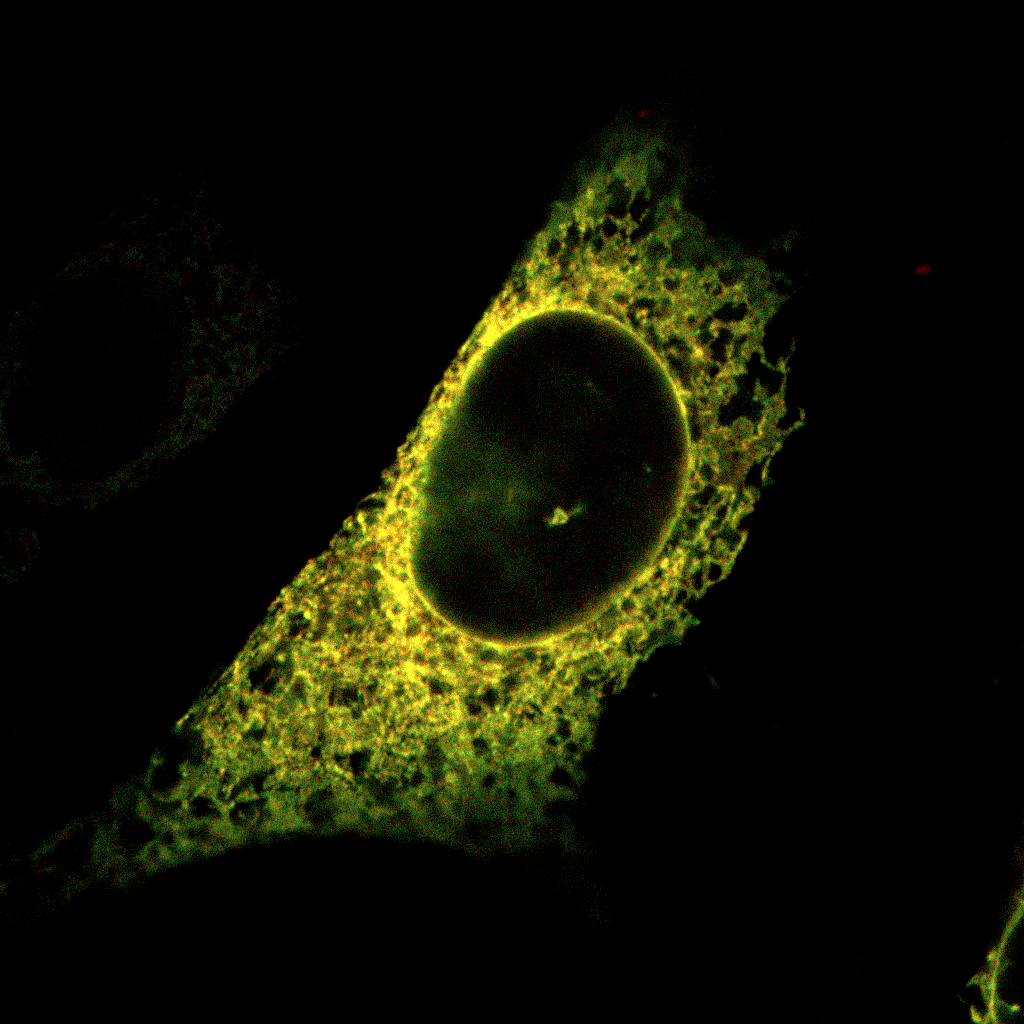

Supplement: Supplementary file 2 — Supporting File 2: advs73976‐sup‐0002‐SuppMat.zip. [file ADVS-13-e11217-s002.zip › KDEL-RAMP4-IF/RAMP4-Ctrl-H2O2-10_c1-2.tif]

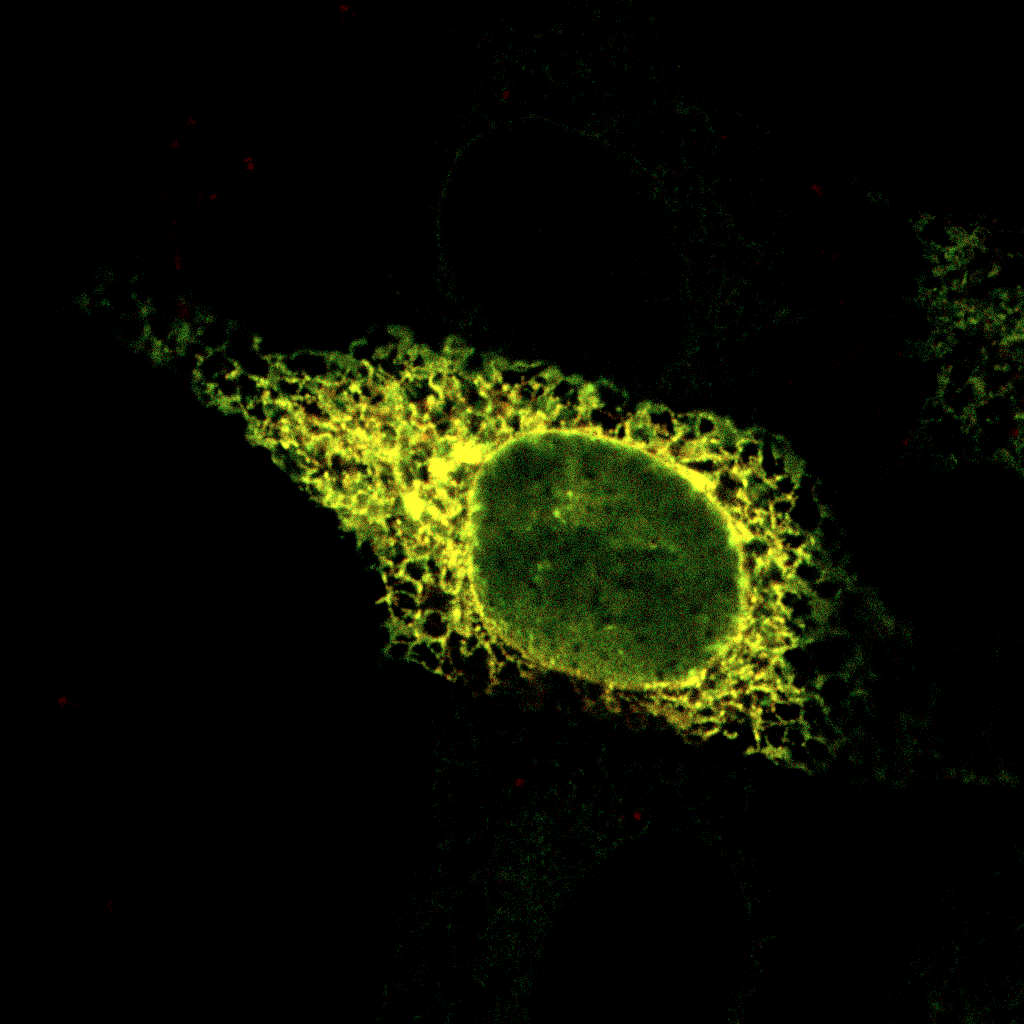

Supplement: Supplementary file 2 — Supporting File 2: advs73976‐sup‐0002‐SuppMat.zip. [file ADVS-13-e11217-s002.zip › KDEL-RAMP4-IF/RAMP4-Ctrl-H2O2-9-adj_c1-2.tif]

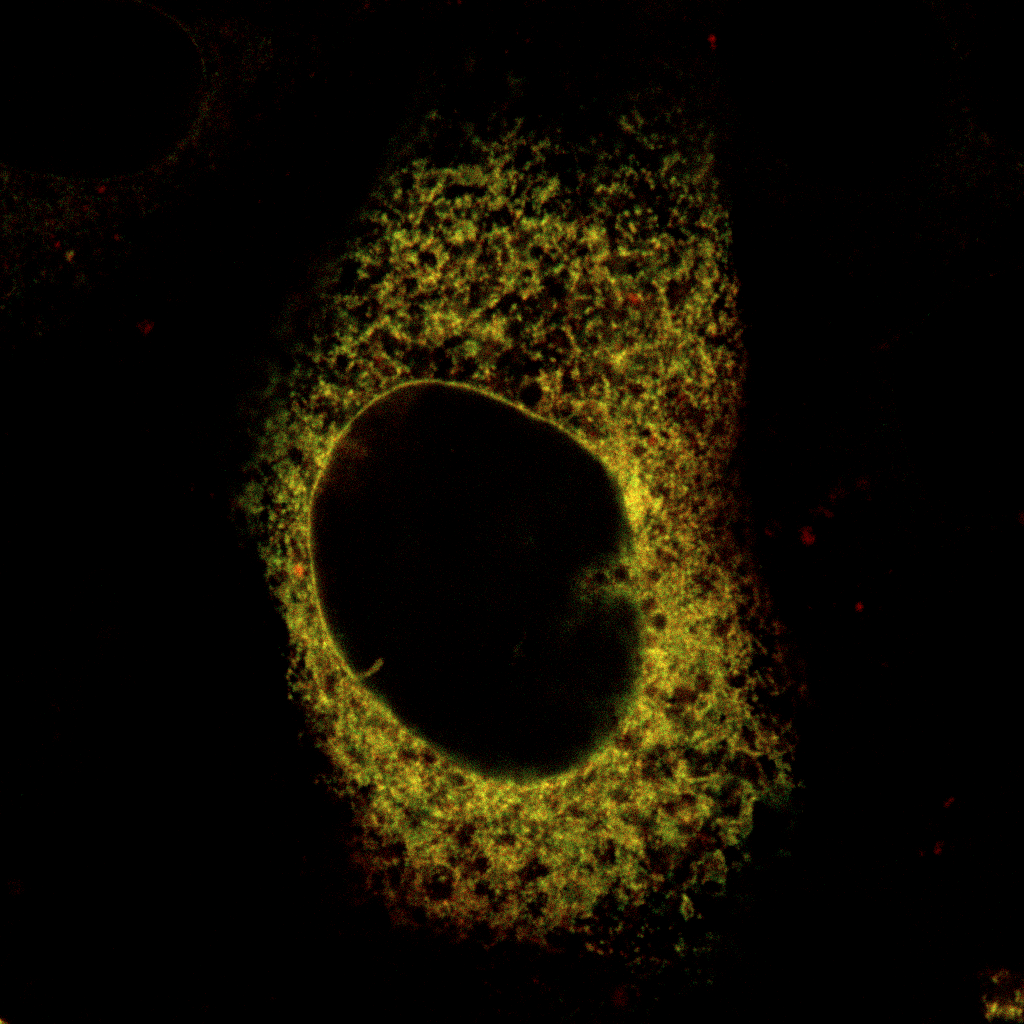

Supplement: Supplementary file 2 — Supporting File 2: advs73976‐sup‐0002‐SuppMat.zip. [file ADVS-13-e11217-s002.zip › KDEL-RAMP4-IF/RAMP4-DDRGK1-C-7_c1-2.tif]

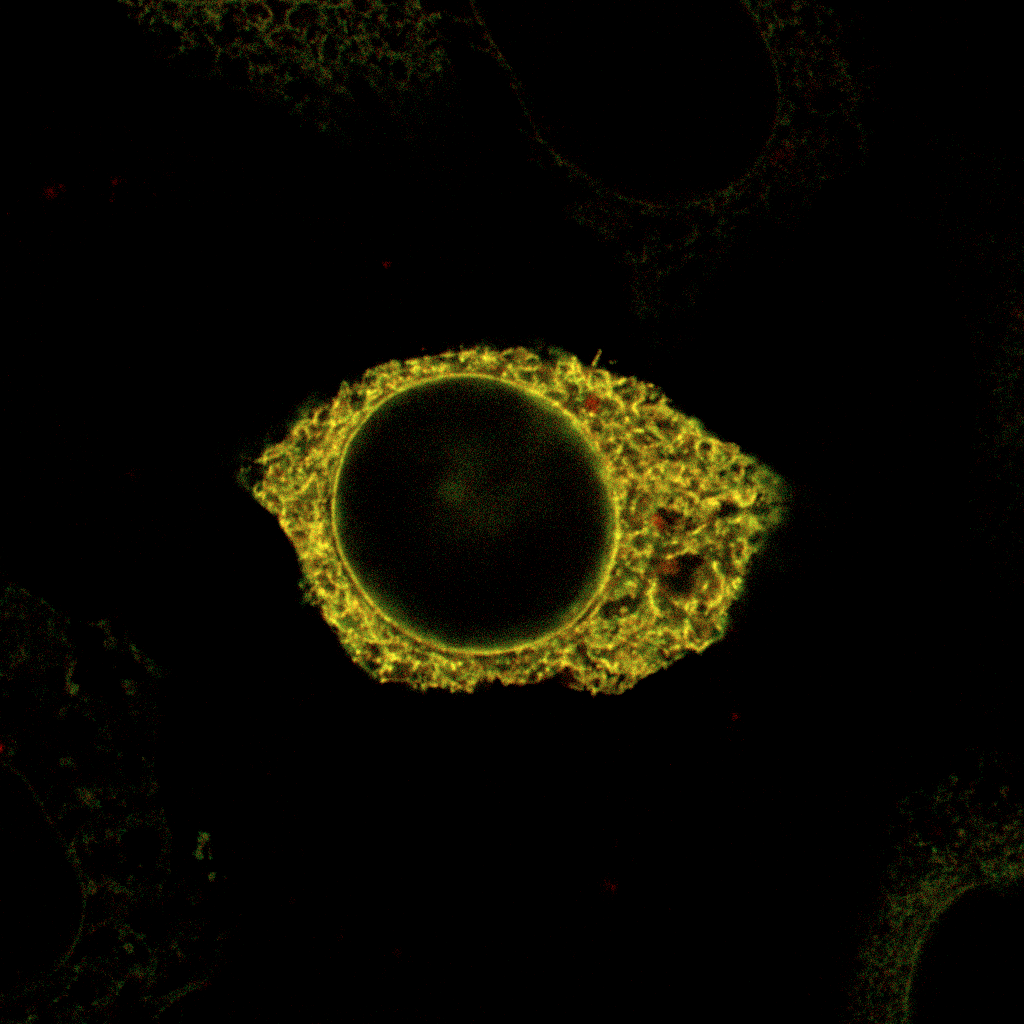

Supplement: Supplementary file 2 — Supporting File 2: advs73976‐sup‐0002‐SuppMat.zip. [file ADVS-13-e11217-s002.zip › KDEL-RAMP4-IF/RAMP4-DDRGK1-H2O2-1_c1-2.tif]

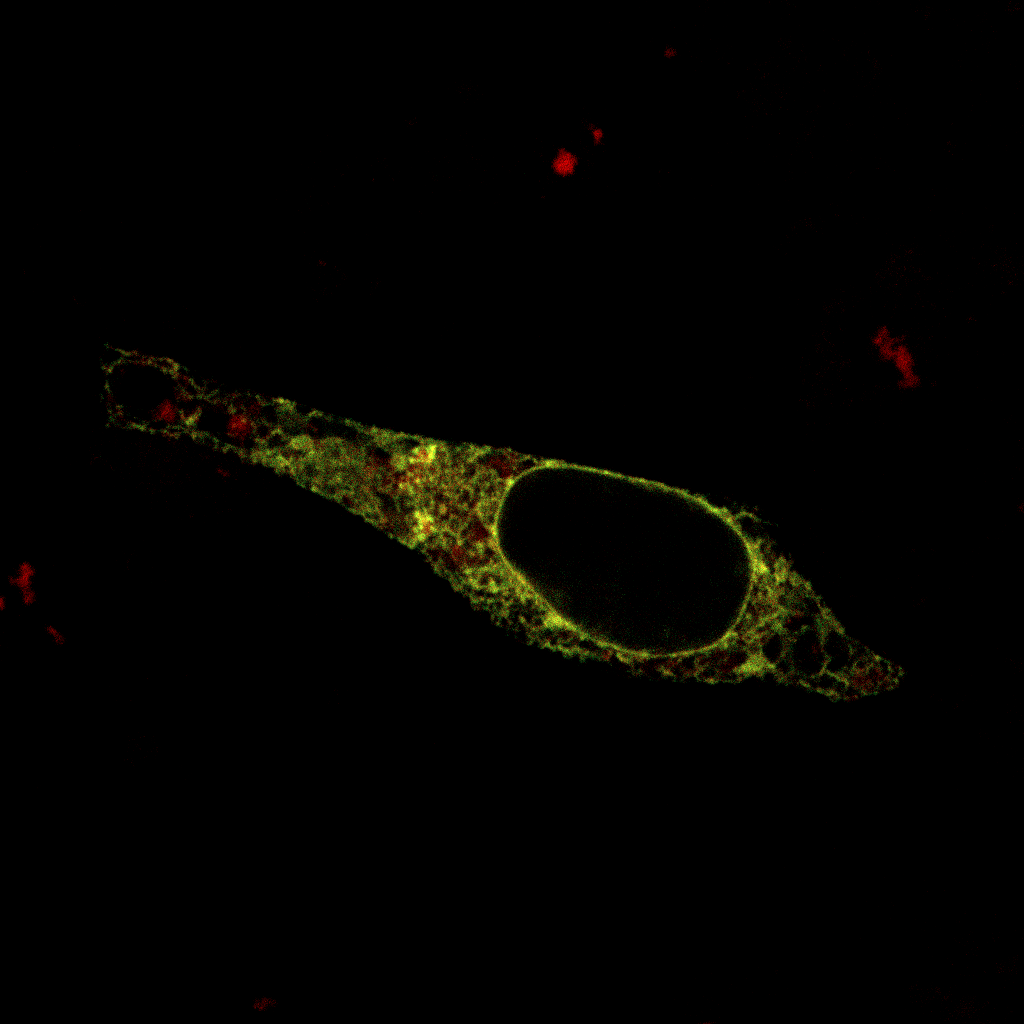

Supplement: Supplementary file 2 — Supporting File 2: advs73976‐sup‐0002‐SuppMat.zip. [file ADVS-13-e11217-s002.zip › KDEL-RAMP4-IF/RAMP4-XIAP-Control-4-adj_c1-2.tif]

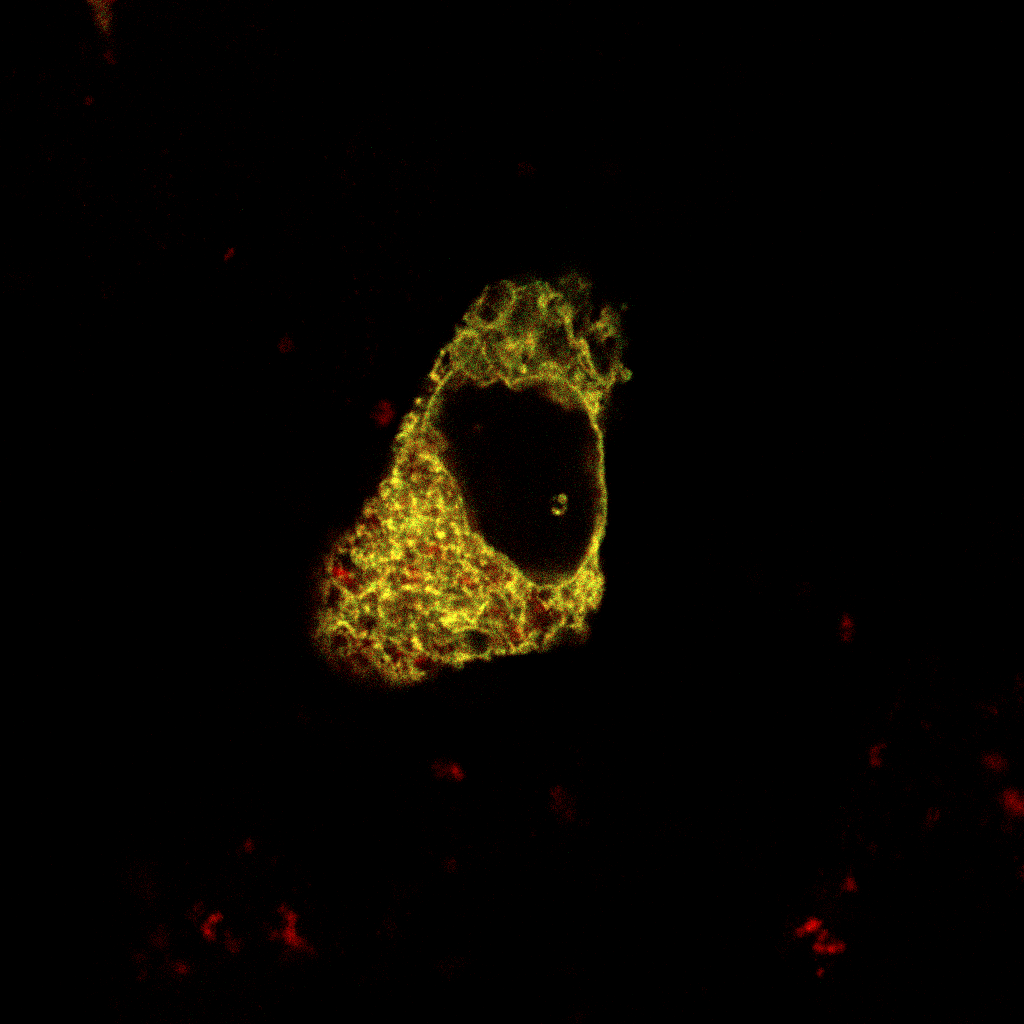

Supplement: Supplementary file 2 — Supporting File 2: advs73976‐sup‐0002‐SuppMat.zip. [file ADVS-13-e11217-s002.zip › KDEL-RAMP4-IF/RAMP4-XIAP-H2O2-10-adj_c1-2.tif]

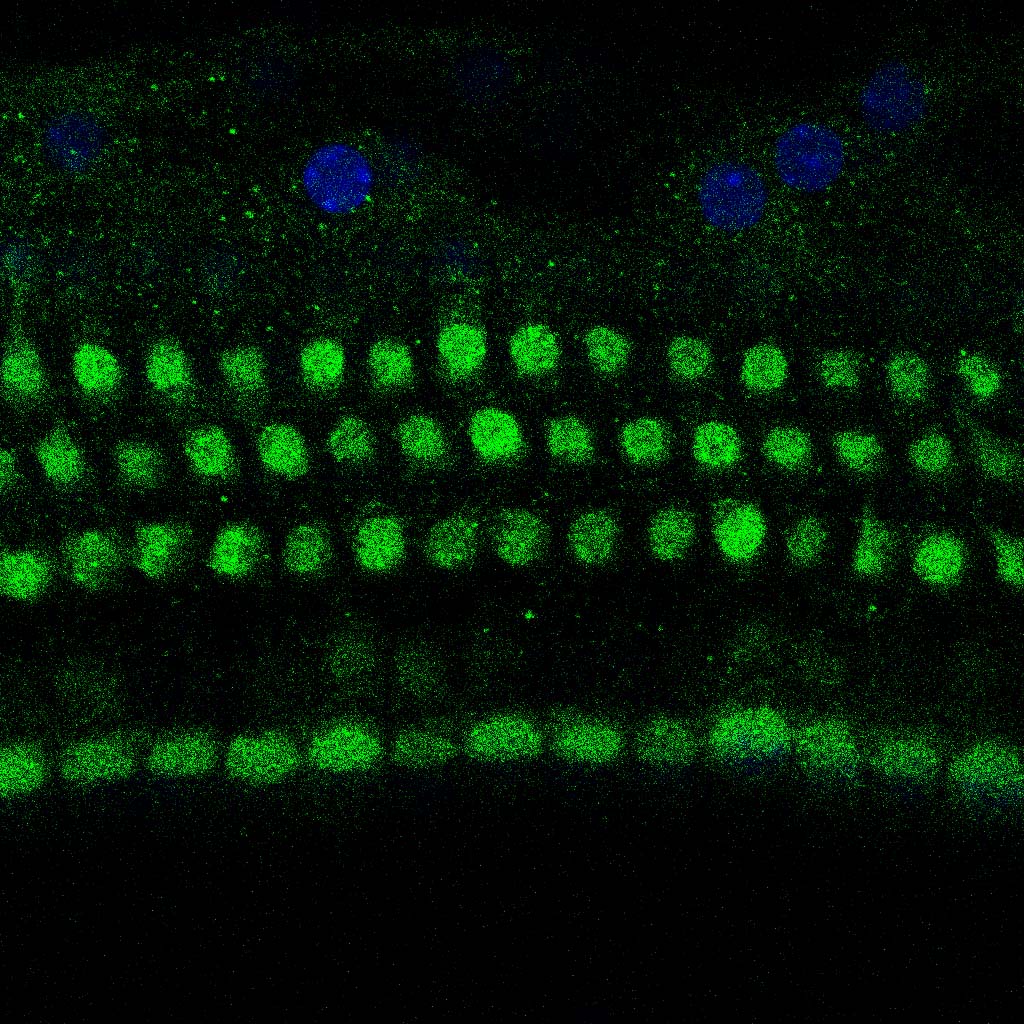

Supplement: Supplementary file 2 — Supporting File 2: advs73976‐sup‐0002‐SuppMat.zip. [file ADVS-13-e11217-s002.zip › Myo7a-JPEG/A6_z4c1+2.jpg]

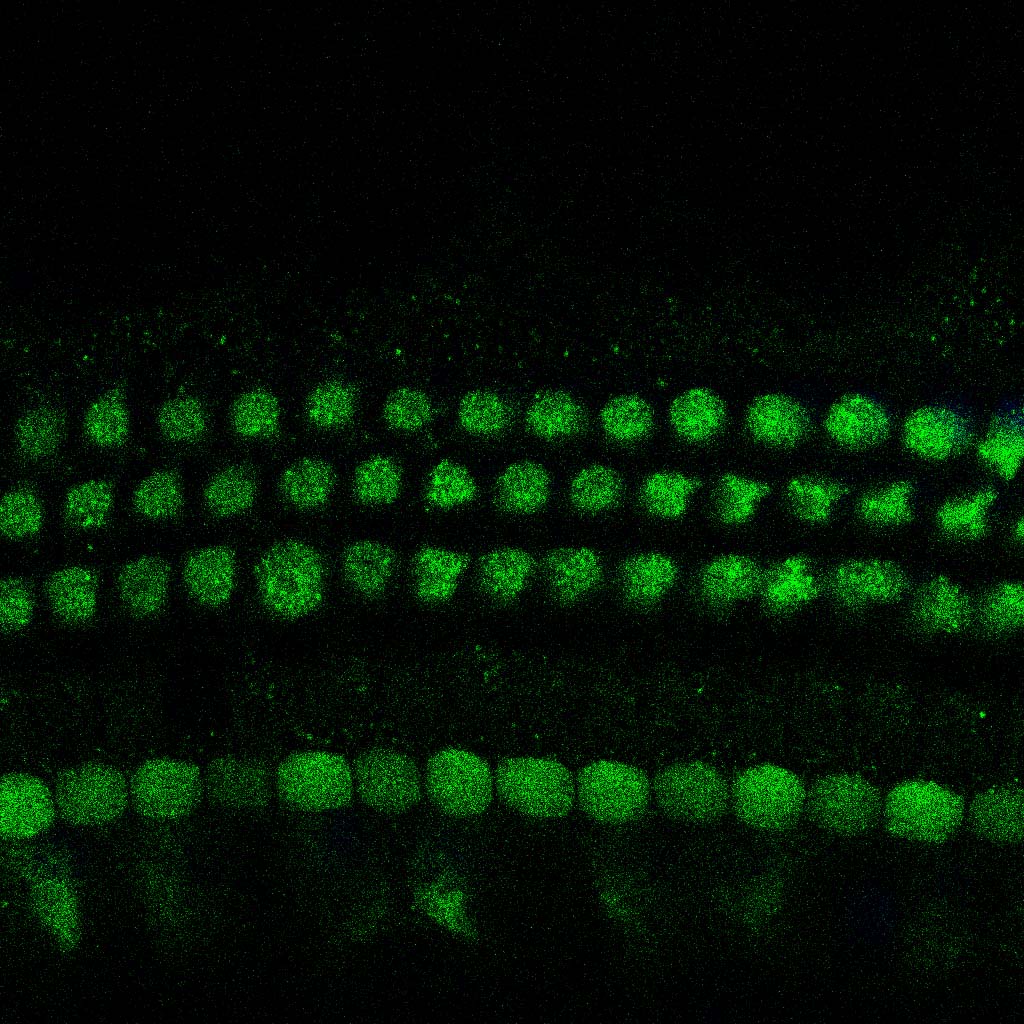

Supplement: Supplementary file 2 — Supporting File 2: advs73976‐sup‐0002‐SuppMat.zip. [file ADVS-13-e11217-s002.zip › Myo7a-JPEG/A8_z03c1+2.jpg]

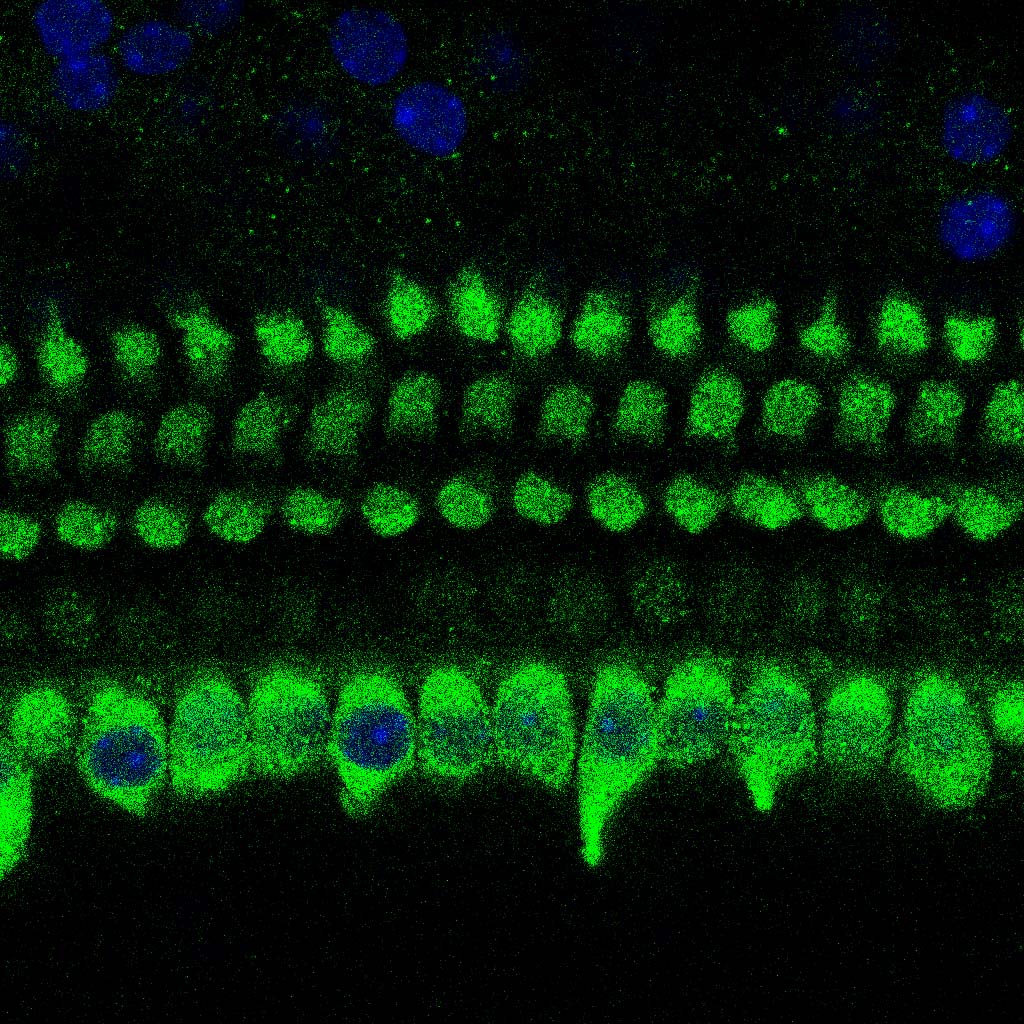

Supplement: Supplementary file 2 — Supporting File 2: advs73976‐sup‐0002‐SuppMat.zip. [file ADVS-13-e11217-s002.zip › Myo7a-JPEG/A9_z3c1+2.jpg]

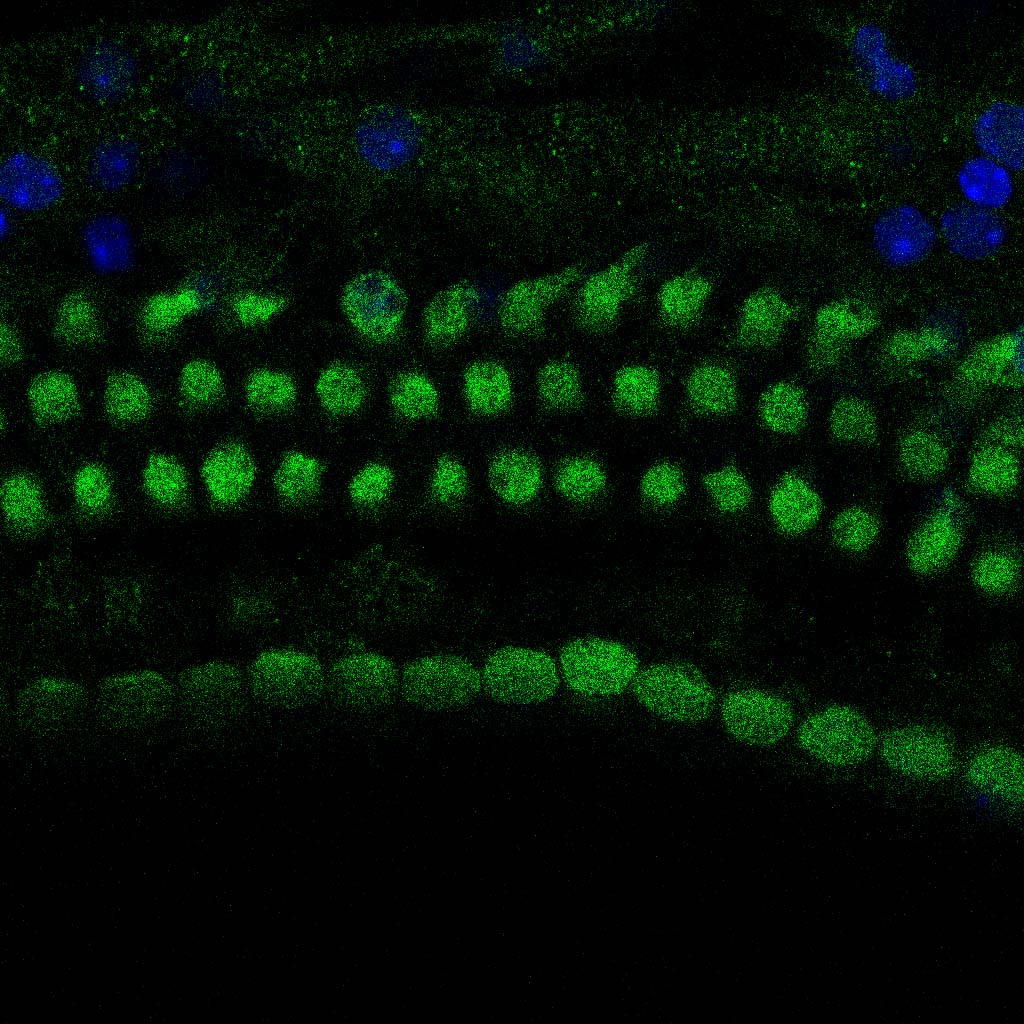

Supplement: Supplementary file 2 — Supporting File 2: advs73976‐sup‐0002‐SuppMat.zip. [file ADVS-13-e11217-s002.zip › Myo7a-JPEG/ap4_z3c1+2.jpg]

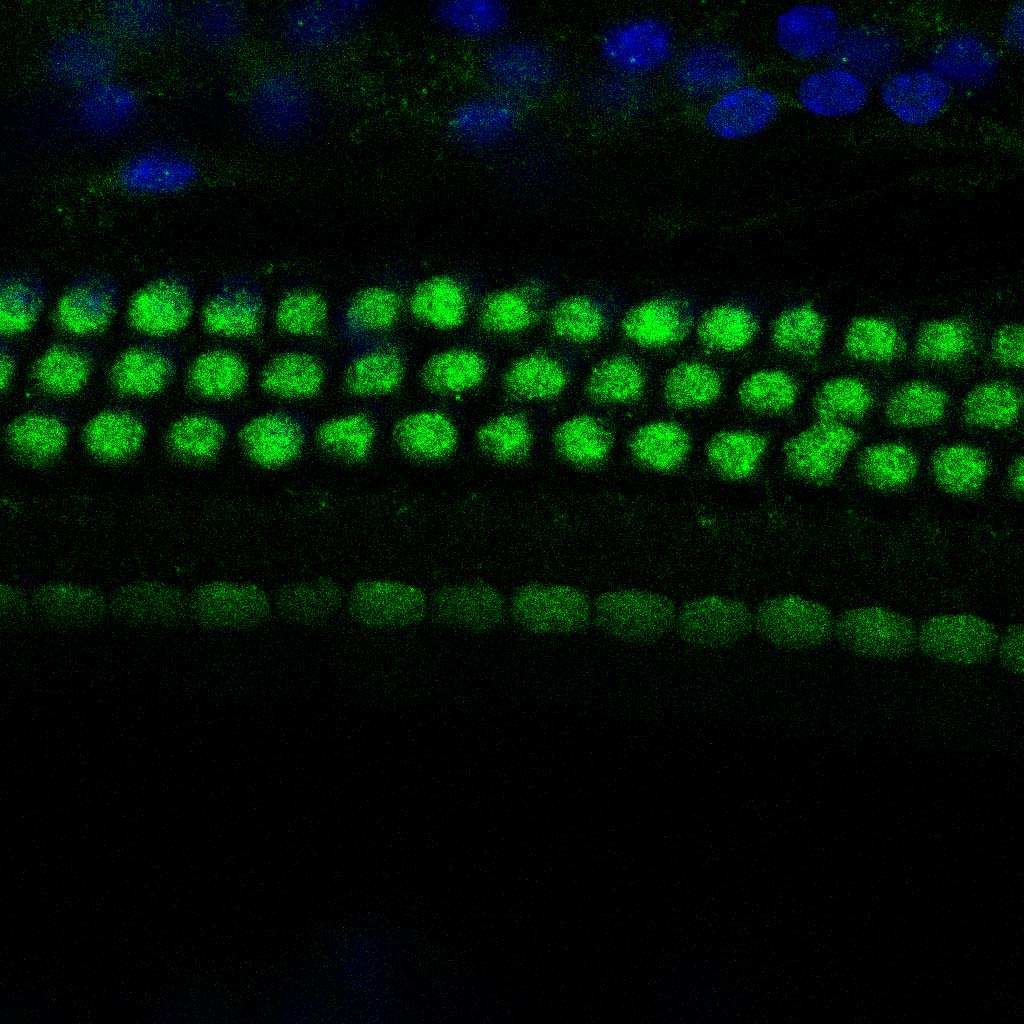

Supplement: Supplementary file 2 — Supporting File 2: advs73976‐sup‐0002‐SuppMat.zip. [file ADVS-13-e11217-s002.zip › Myo7a-JPEG/B CTR1_z3c1+2.jpg]

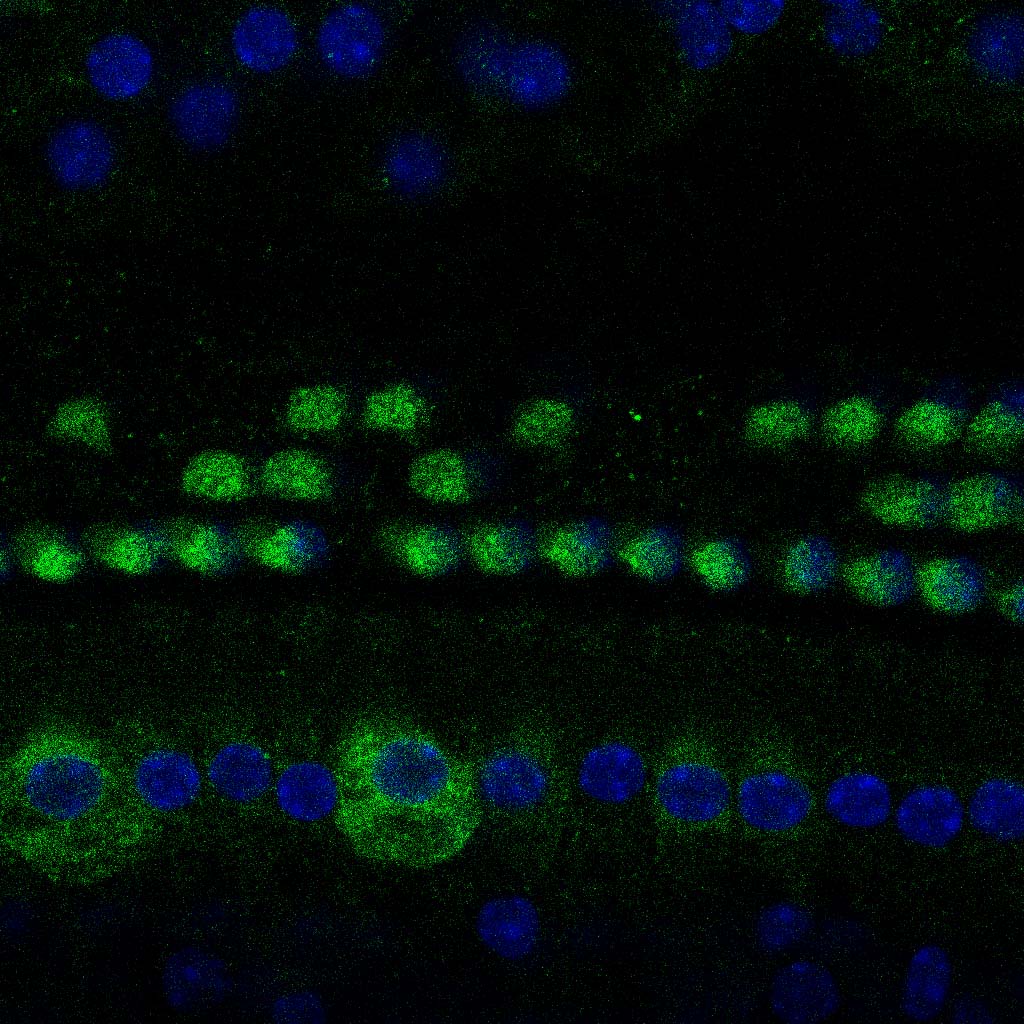

Supplement: Supplementary file 2 — Supporting File 2: advs73976‐sup‐0002‐SuppMat.zip. [file ADVS-13-e11217-s002.zip › Myo7a-JPEG/b17_z5c1+2.jpg]

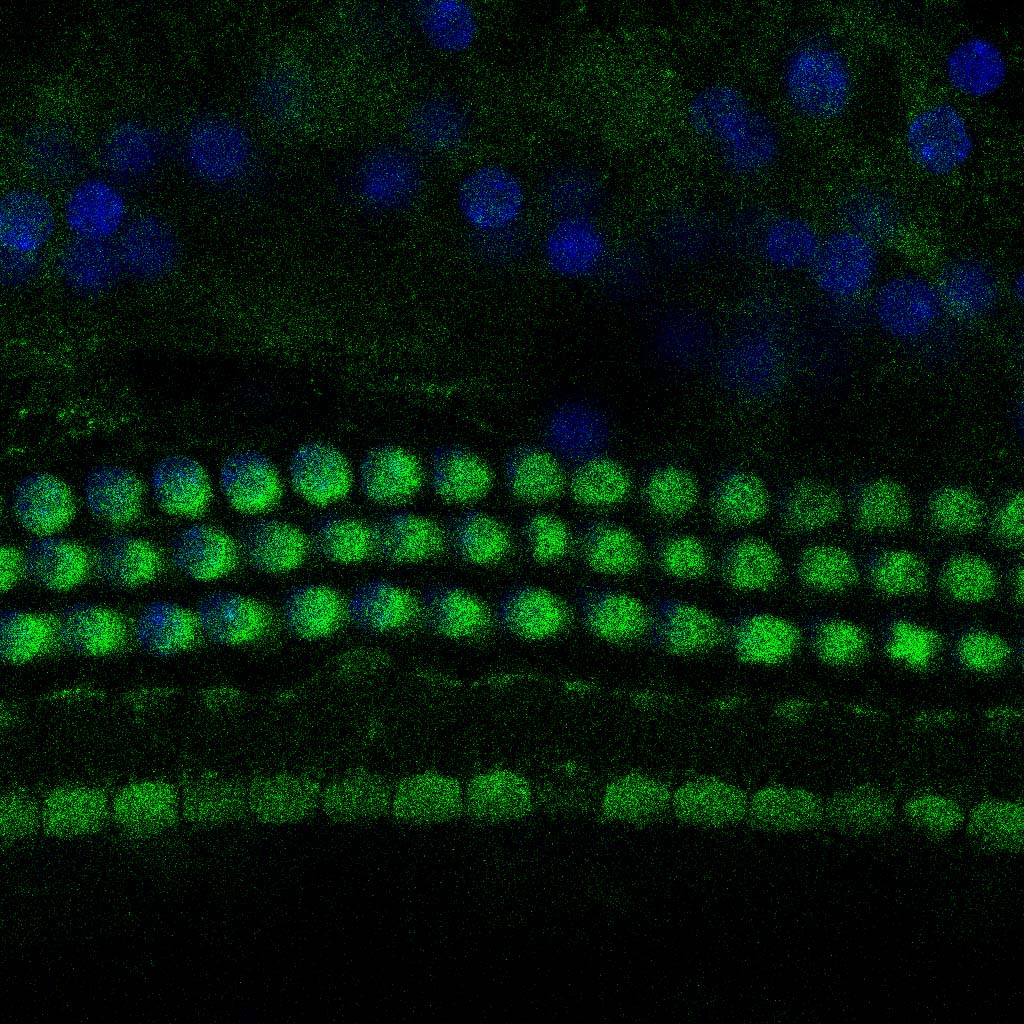

Supplement: Supplementary file 2 — Supporting File 2: advs73976‐sup‐0002‐SuppMat.zip. [file ADVS-13-e11217-s002.zip › Myo7a-JPEG/ba4_z4c1+2.jpg]

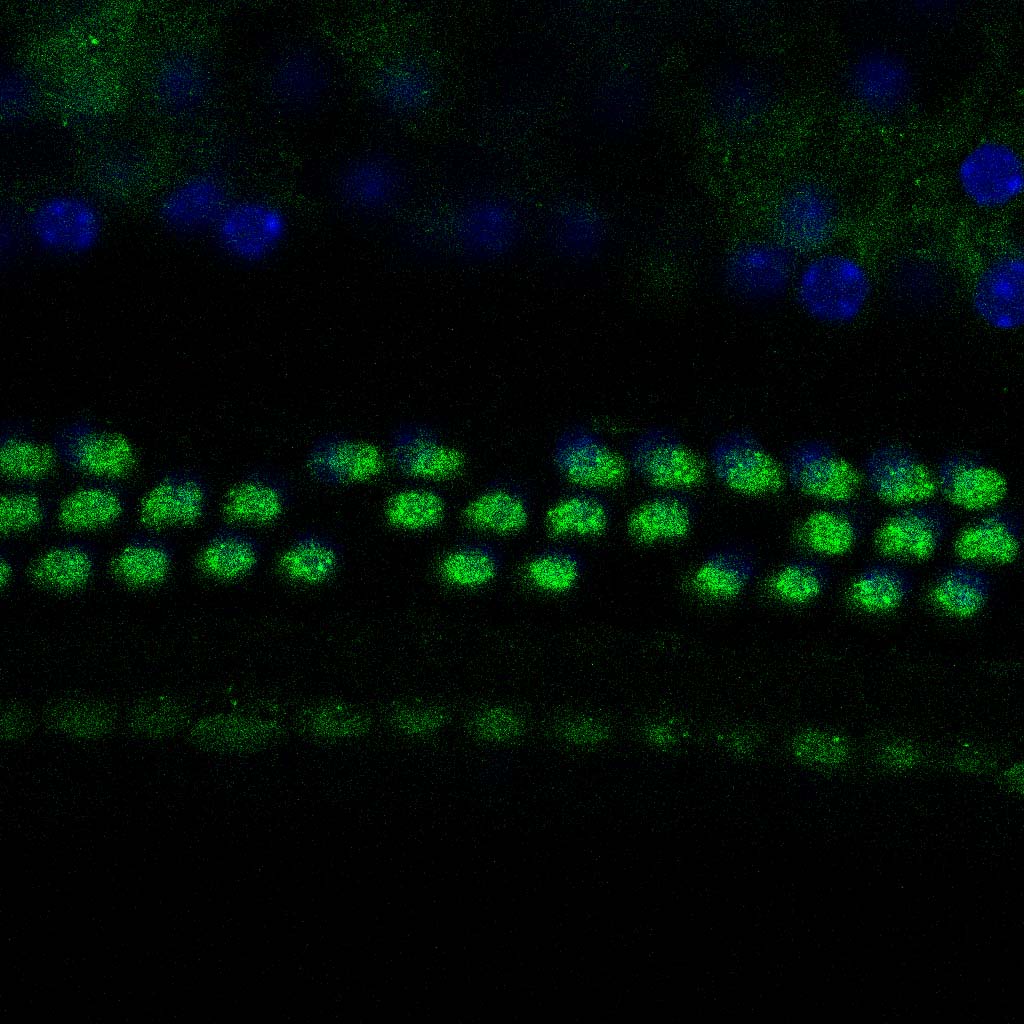

Supplement: Supplementary file 2 — Supporting File 2: advs73976‐sup‐0002‐SuppMat.zip. [file ADVS-13-e11217-s002.zip › Myo7a-JPEG/gb13_z3c1+2.jpg]

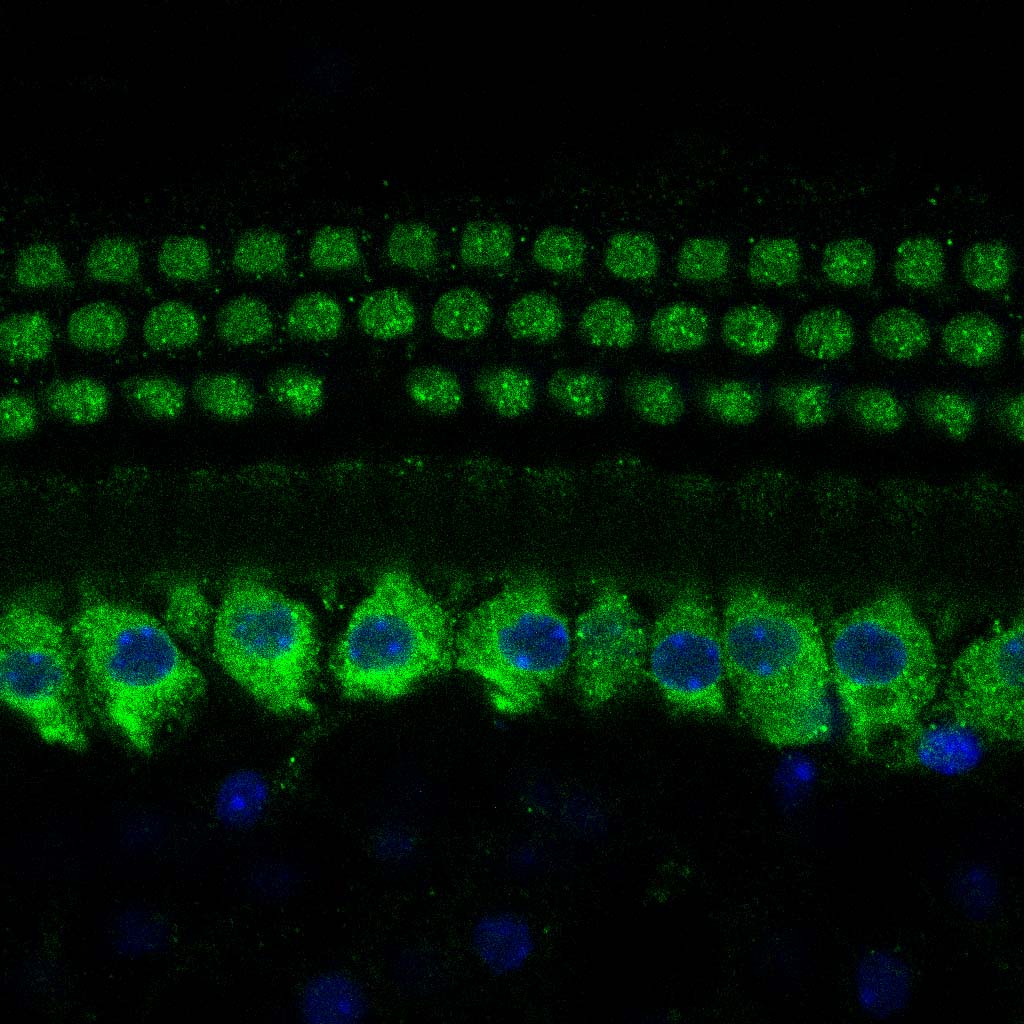

Supplement: Supplementary file 2 — Supporting File 2: advs73976‐sup‐0002‐SuppMat.zip. [file ADVS-13-e11217-s002.zip › Myo7a-JPEG/m13 g+n_z2c1+2.jpg]

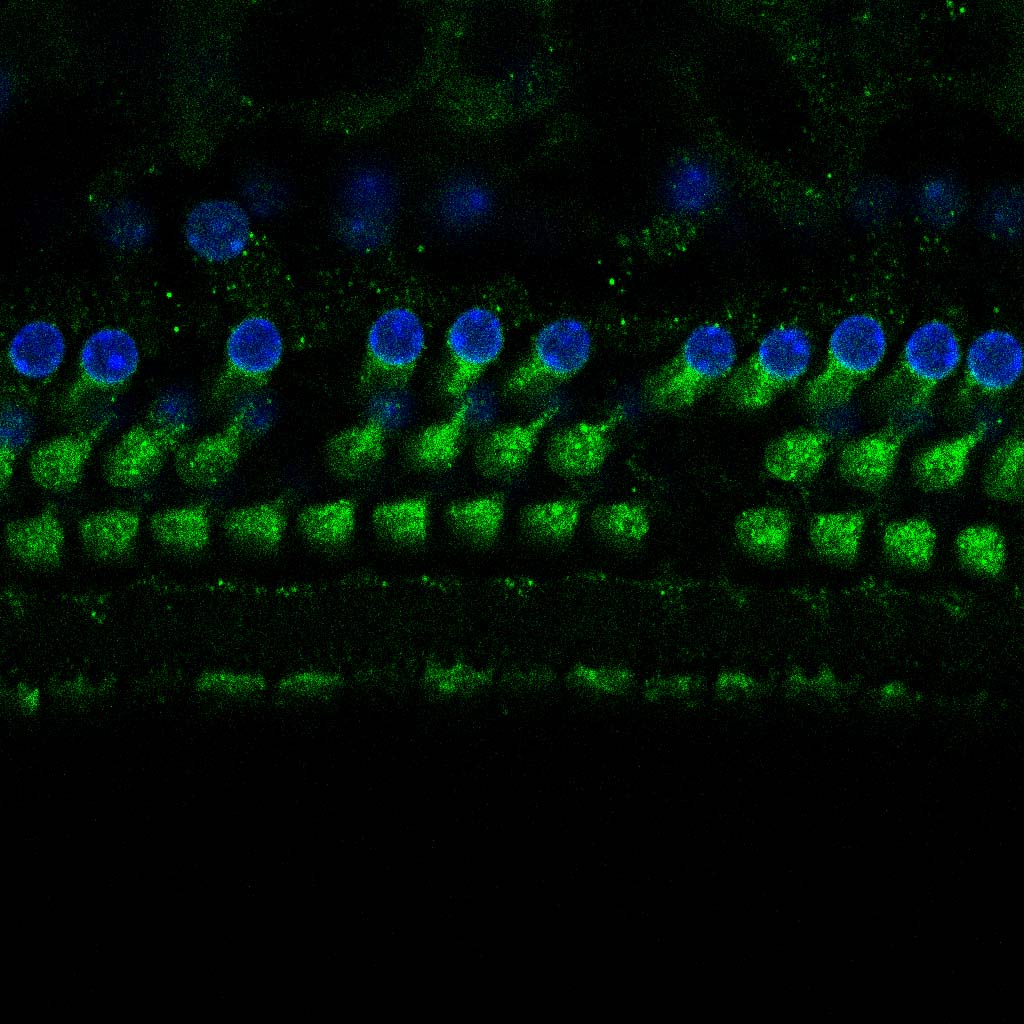

Supplement: Supplementary file 2 — Supporting File 2: advs73976‐sup‐0002‐SuppMat.zip. [file ADVS-13-e11217-s002.zip › Myo7a-JPEG/m3 n_z04c1+2.jpg]

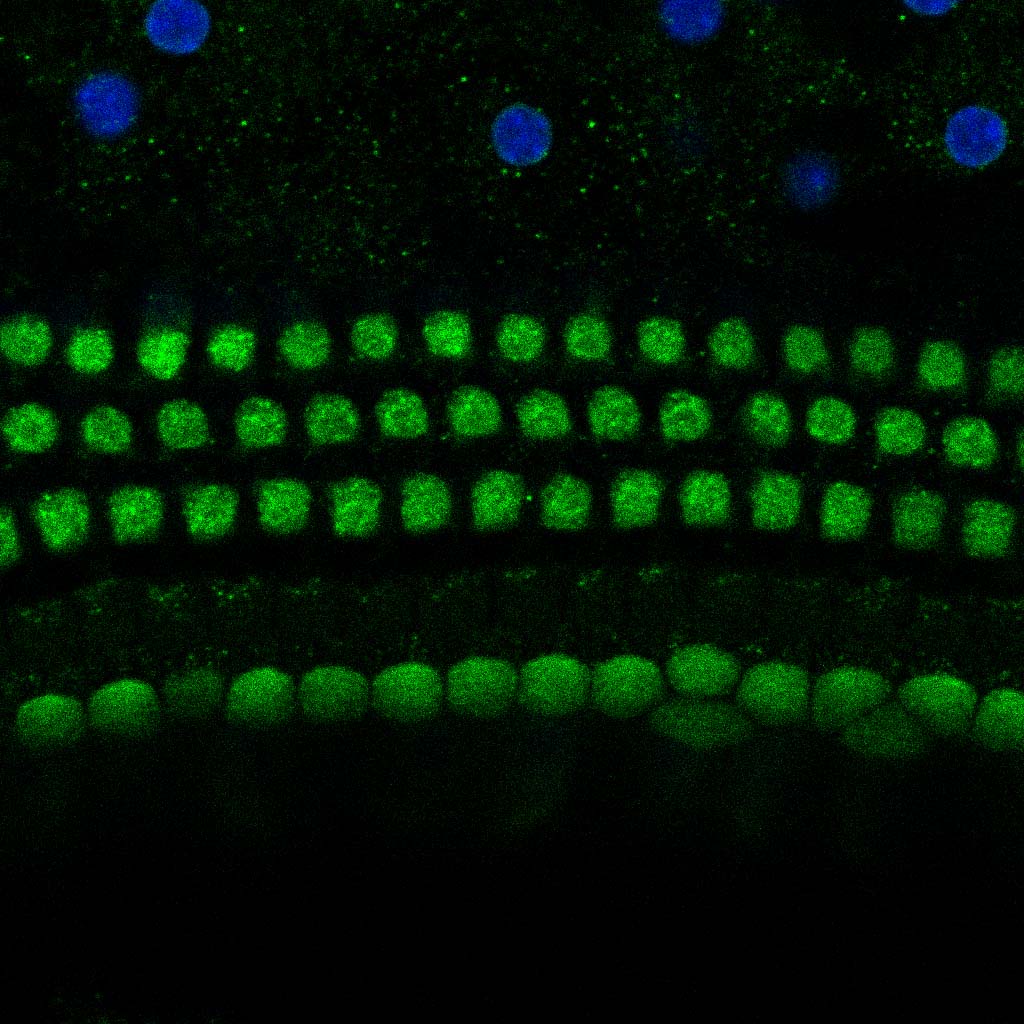

Supplement: Supplementary file 2 — Supporting File 2: advs73976‐sup‐0002‐SuppMat.zip. [file ADVS-13-e11217-s002.zip › Myo7a-JPEG/M3_z2c1+2.jpg]

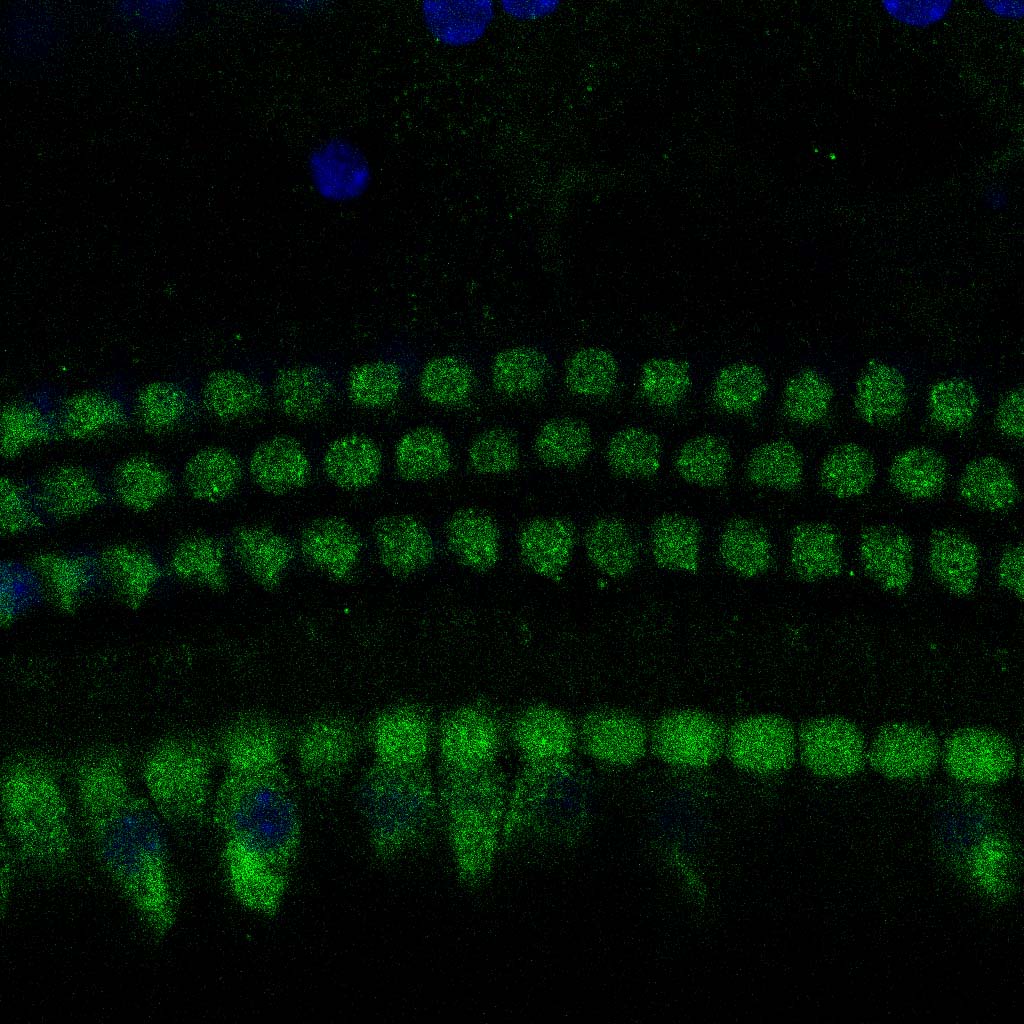

Supplement: Supplementary file 2 — Supporting File 2: advs73976‐sup‐0002‐SuppMat.zip. [file ADVS-13-e11217-s002.zip › Myo7a-JPEG/MG6_z2c1+2.jpg]

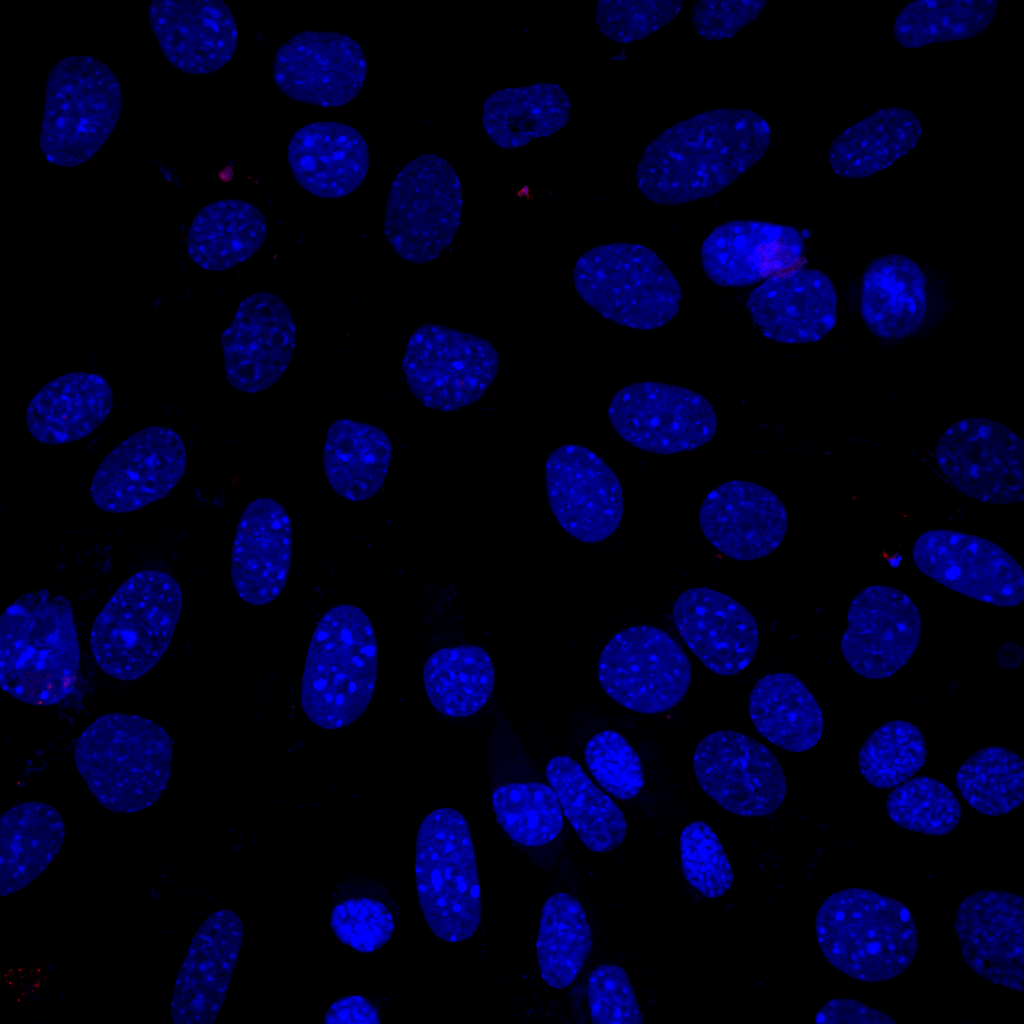

Supplement: Supplementary file 2 — Supporting File 2: advs73976‐sup‐0002‐SuppMat.zip. [file ADVS-13-e11217-s002.zip › TUNEL/figure S1/CONTROL-3_c1-2.tif]

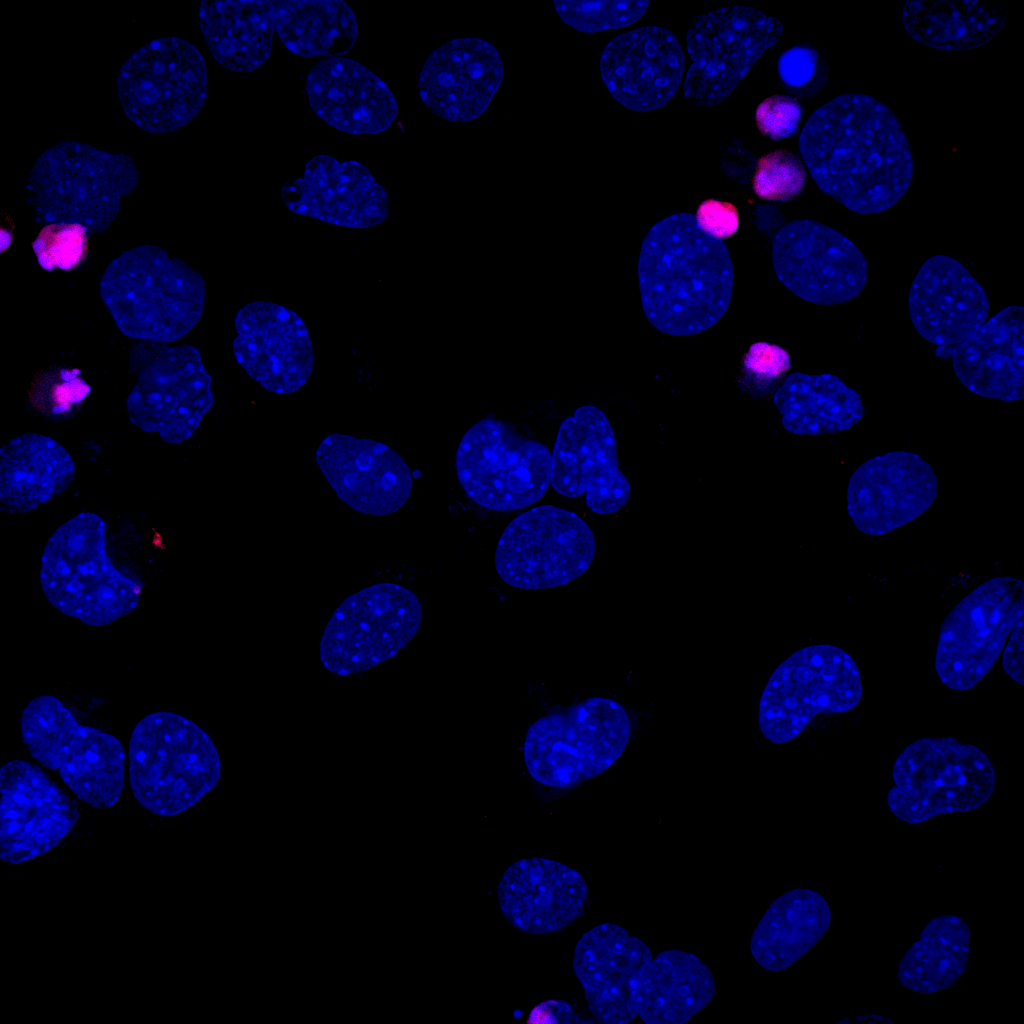

Supplement: Supplementary file 2 — Supporting File 2: advs73976‐sup‐0002‐SuppMat.zip. [file ADVS-13-e11217-s002.zip › TUNEL/figure S1/CONTROL-H2O2-12_c1-2.tif]

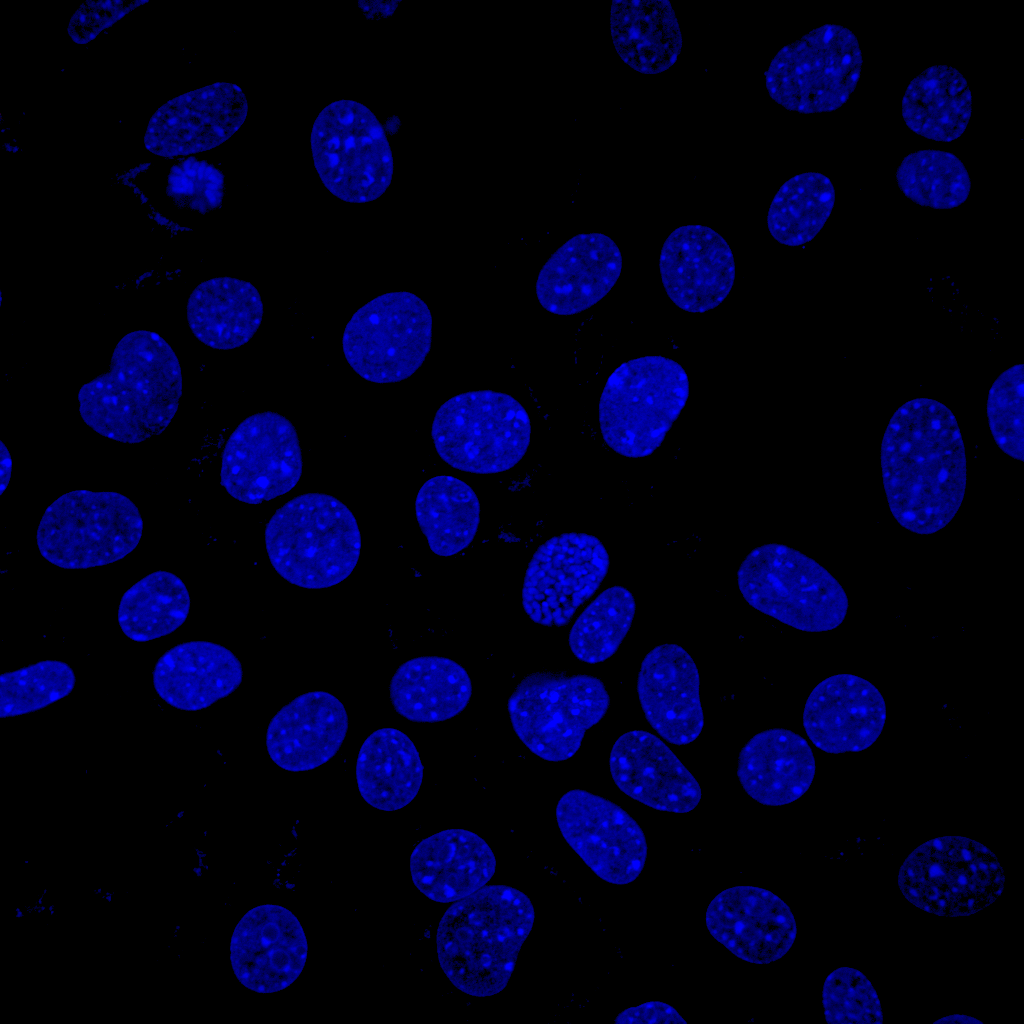

Supplement: Supplementary file 2 — Supporting File 2: advs73976‐sup‐0002‐SuppMat.zip. [file ADVS-13-e11217-s002.zip › TUNEL/figure S1/XIAP-CONTROL-4_c1-2.tif]

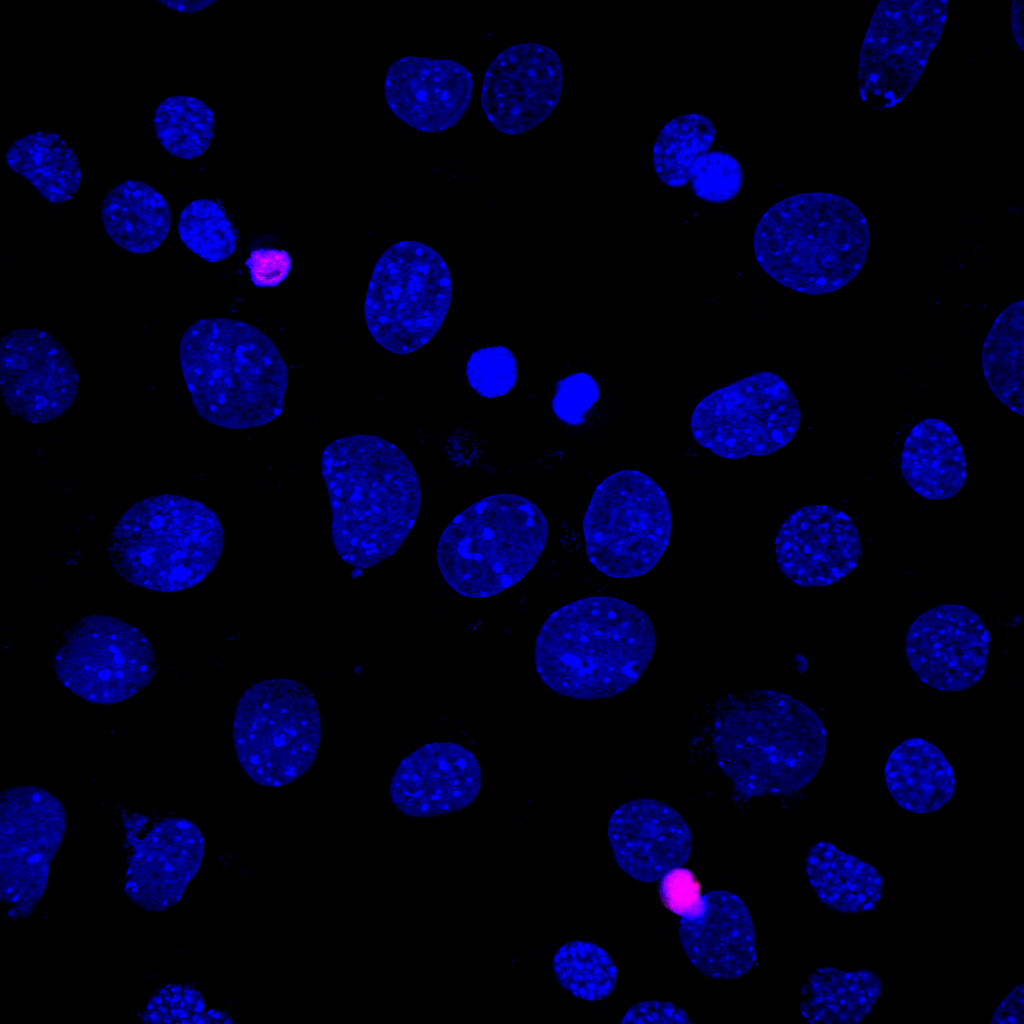

Supplement: Supplementary file 2 — Supporting File 2: advs73976‐sup‐0002‐SuppMat.zip. [file ADVS-13-e11217-s002.zip › TUNEL/figure S1/XIAP-H2O2-6_c1-2.tif]

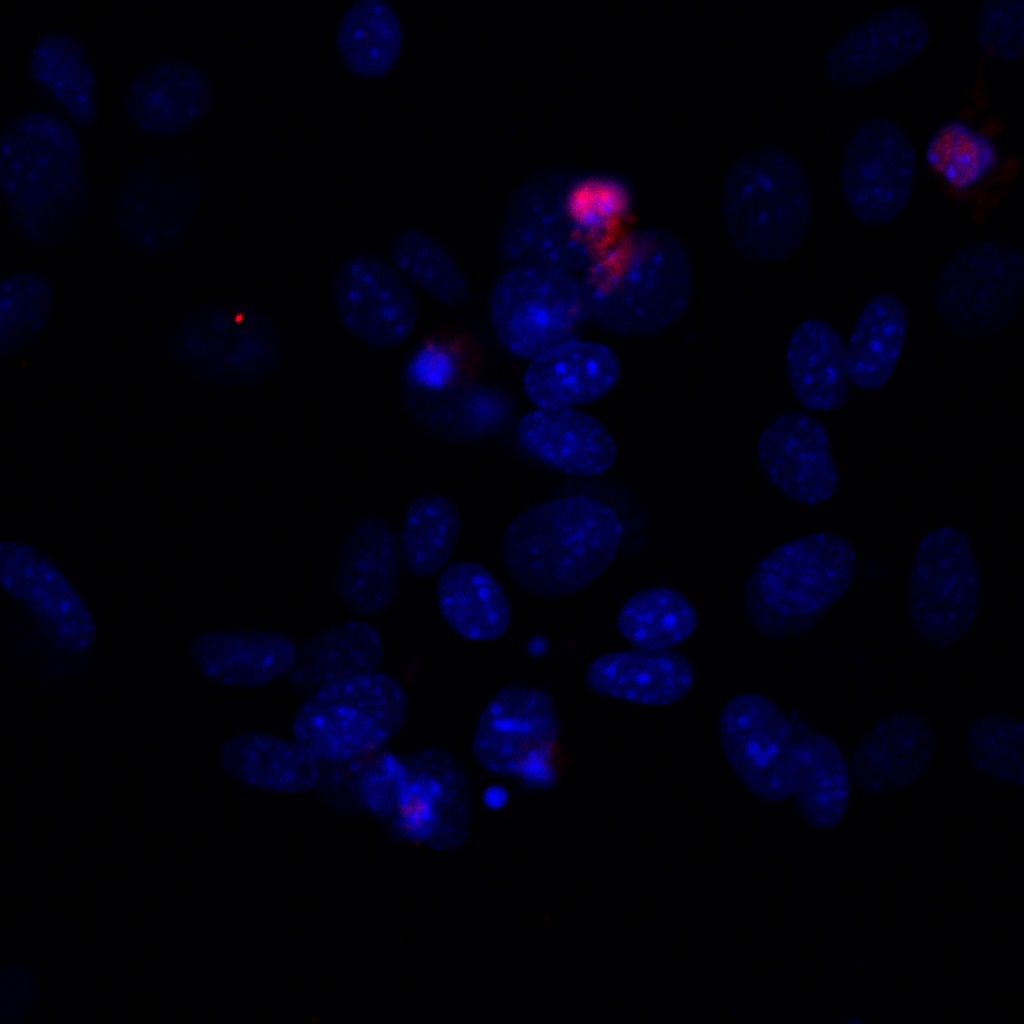

Supplement: Supplementary file 2 — Supporting File 2: advs73976‐sup‐0002‐SuppMat.zip. [file ADVS-13-e11217-s002.zip › TUNEL/figure1/12h1-2_c1+2.jpg]

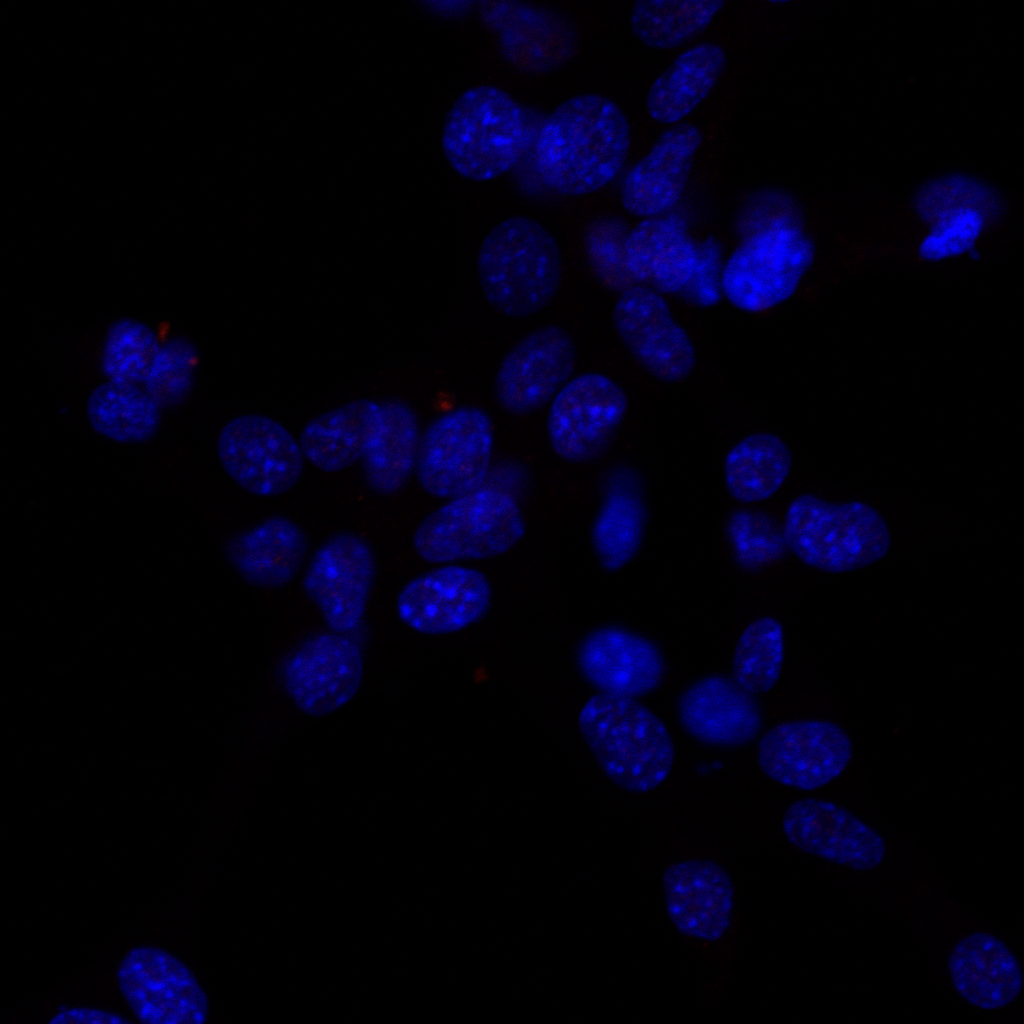

Supplement: Supplementary file 2 — Supporting File 2: advs73976‐sup‐0002‐SuppMat.zip. [file ADVS-13-e11217-s002.zip › TUNEL/figure1/1h 2_c1+2.tif]

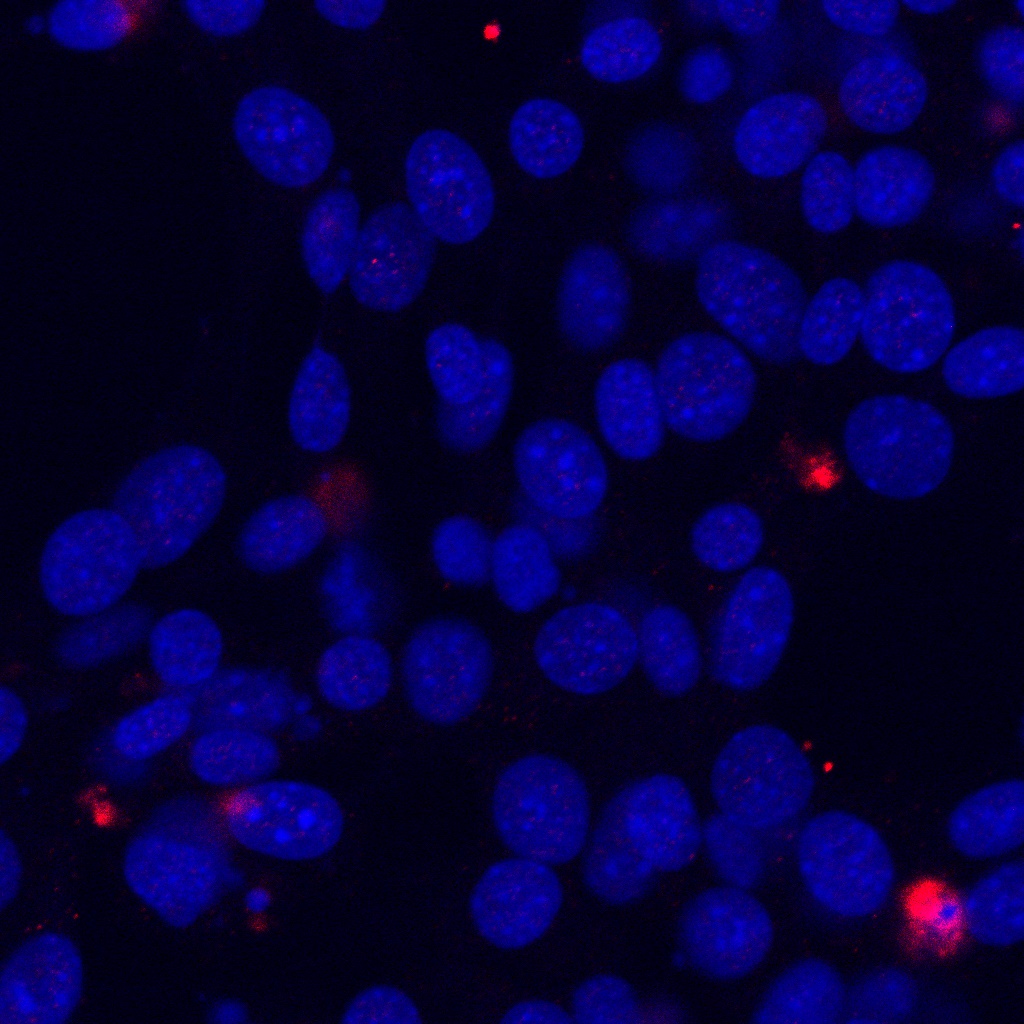

Supplement: Supplementary file 2 — Supporting File 2: advs73976‐sup‐0002‐SuppMat.zip. [file ADVS-13-e11217-s002.zip › TUNEL/figure1/24h1_c1+2.jpg]

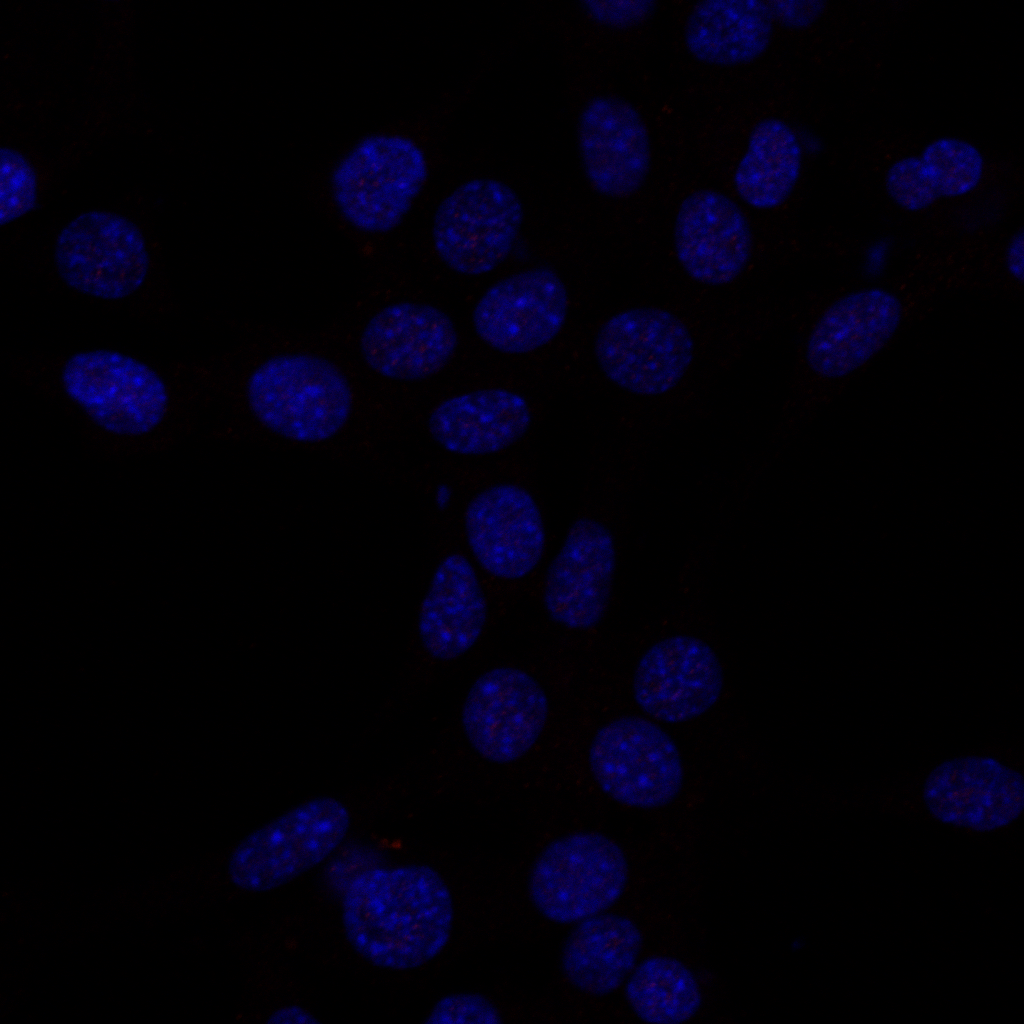

Supplement: Supplementary file 2 — Supporting File 2: advs73976‐sup‐0002‐SuppMat.zip. [file ADVS-13-e11217-s002.zip › TUNEL/figure1/ctrl 1h 3_c1+2.tif]

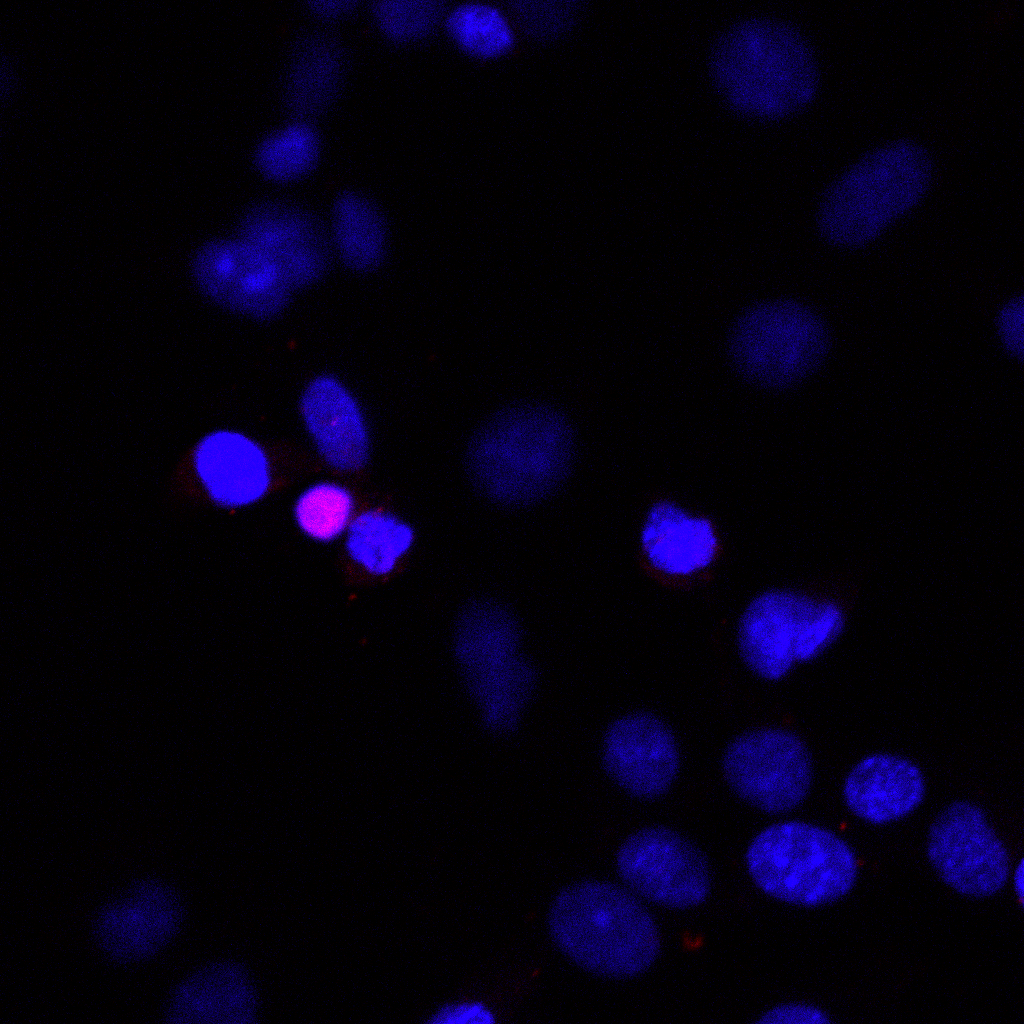

Supplement: Supplementary file 2 — Supporting File 2: advs73976‐sup‐0002‐SuppMat.zip. [file ADVS-13-e11217-s002.zip › TUNEL/figure1/oc1 1h h2o2 1mM 2_c1+2.tif]

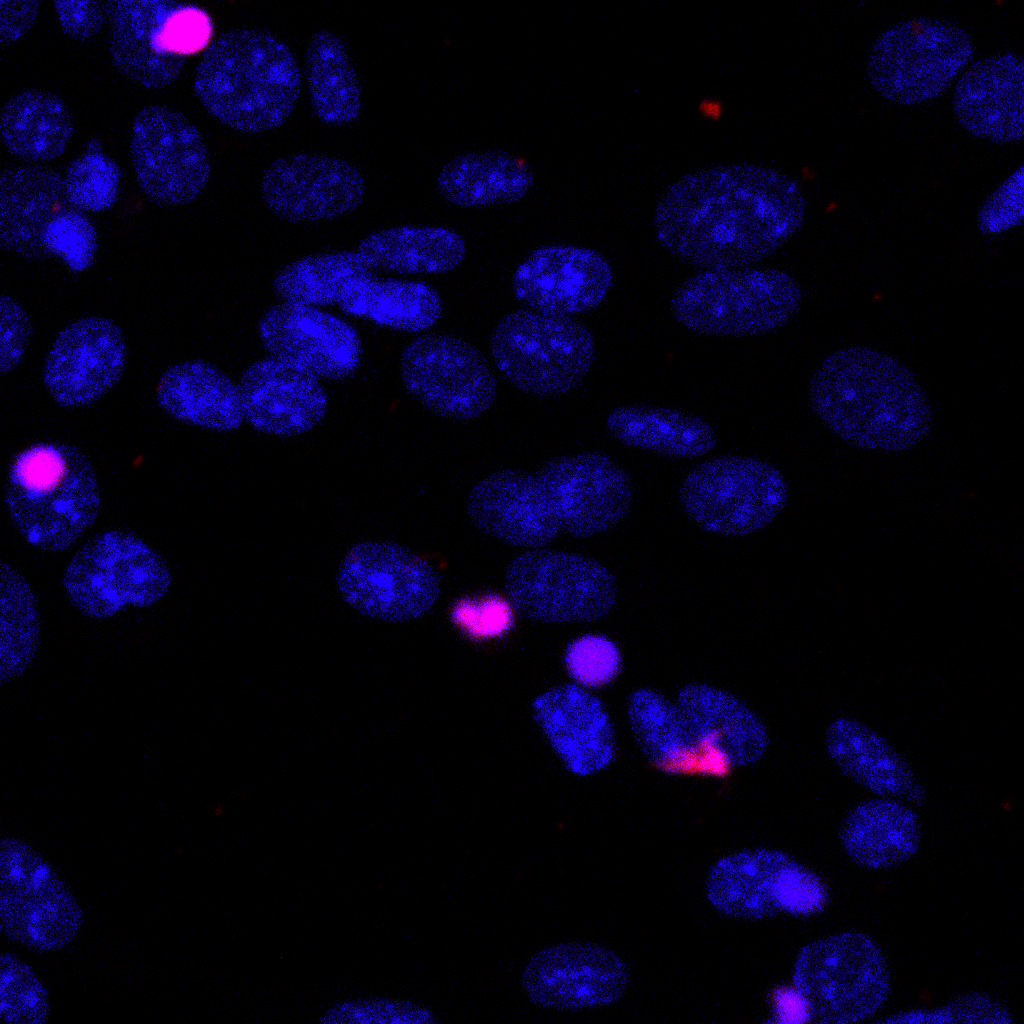

Supplement: Supplementary file 2 — Supporting File 2: advs73976‐sup‐0002‐SuppMat.zip. [file ADVS-13-e11217-s002.zip › TUNEL/figure1/oc112h h2o2 1mM 2_c1+2.tif]

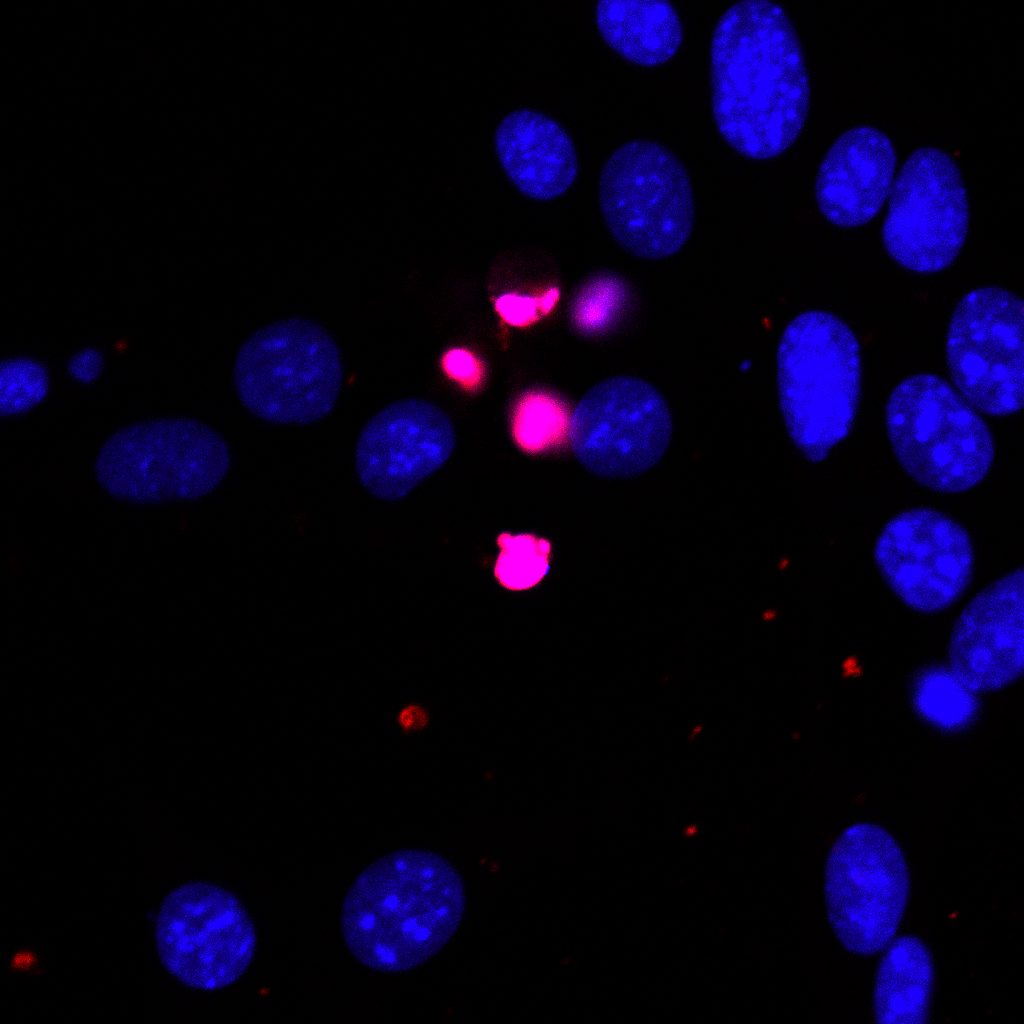

Supplement: Supplementary file 2 — Supporting File 2: advs73976‐sup‐0002‐SuppMat.zip. [file ADVS-13-e11217-s002.zip › TUNEL/figure1/oc124h h2o2 1mM 3_c1+2.tif]

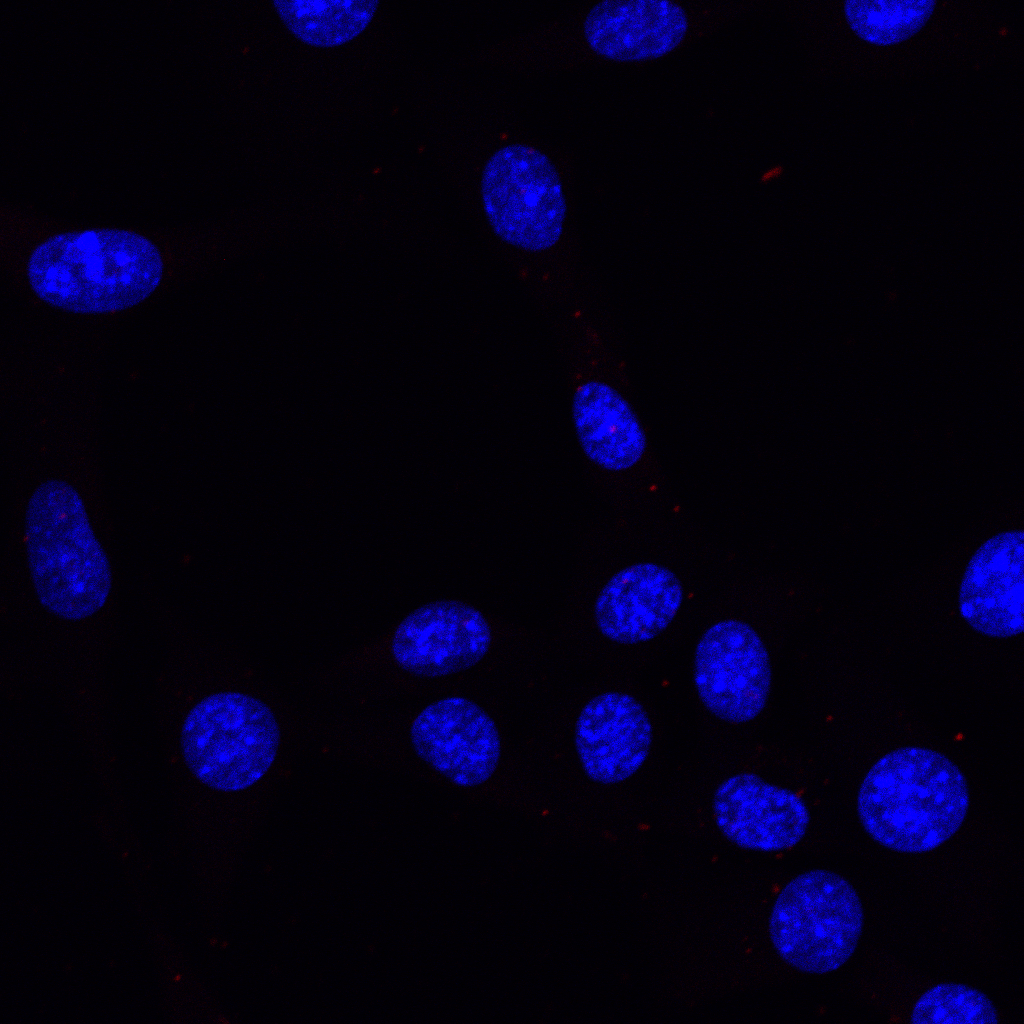

Supplement: Supplementary file 2 — Supporting File 2: advs73976‐sup‐0002‐SuppMat.zip. [file ADVS-13-e11217-s002.zip › TUNEL/figure1/oc1ctrl 1h h2o2 1mM 2_c1+2.tif]

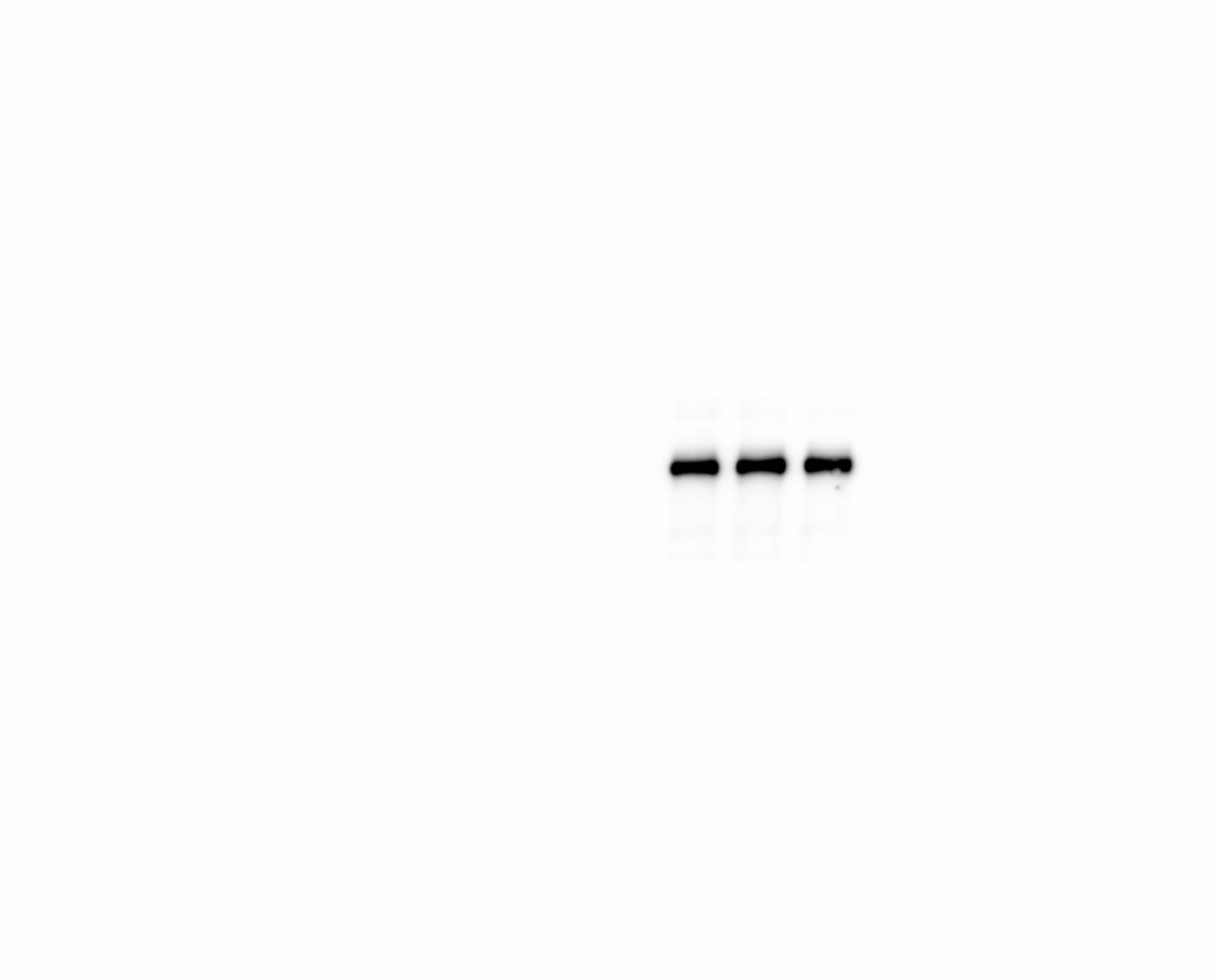

Supplement: Supplementary file 2 — Supporting File 2: advs73976‐sup‐0002‐SuppMat.zip. [file ADVS-13-e11217-s002.zip › WB#U4ee3#U8868#U56fe/Figure S2 Ub/K228R-JPEG/GAPDH-2_5.jpg]

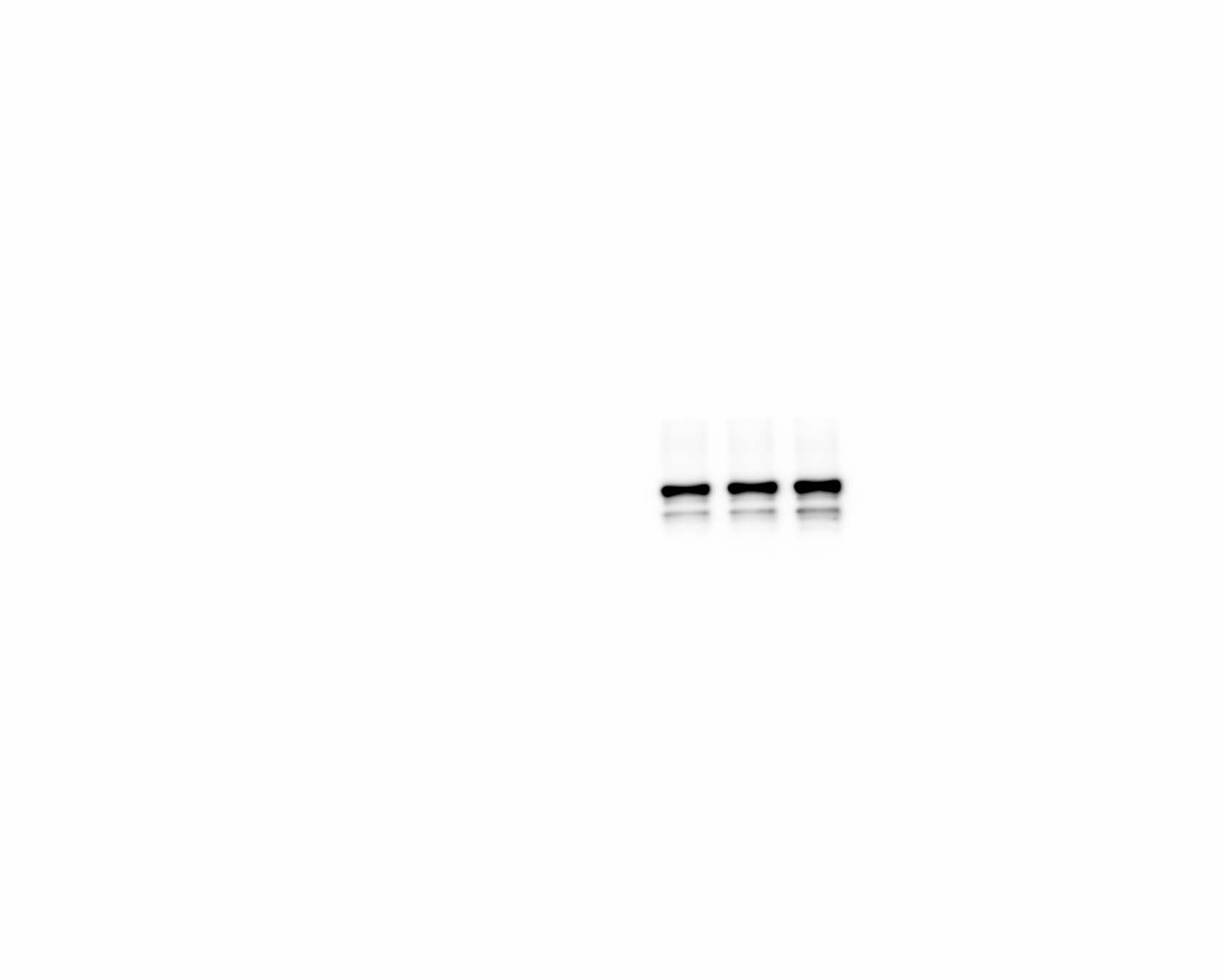

Supplement: Supplementary file 2 — Supporting File 2: advs73976‐sup‐0002‐SuppMat.zip. [file ADVS-13-e11217-s002.zip › WB#U4ee3#U8868#U56fe/Figure S2 Ub/K228R-JPEG/HA-1_8.jpg]

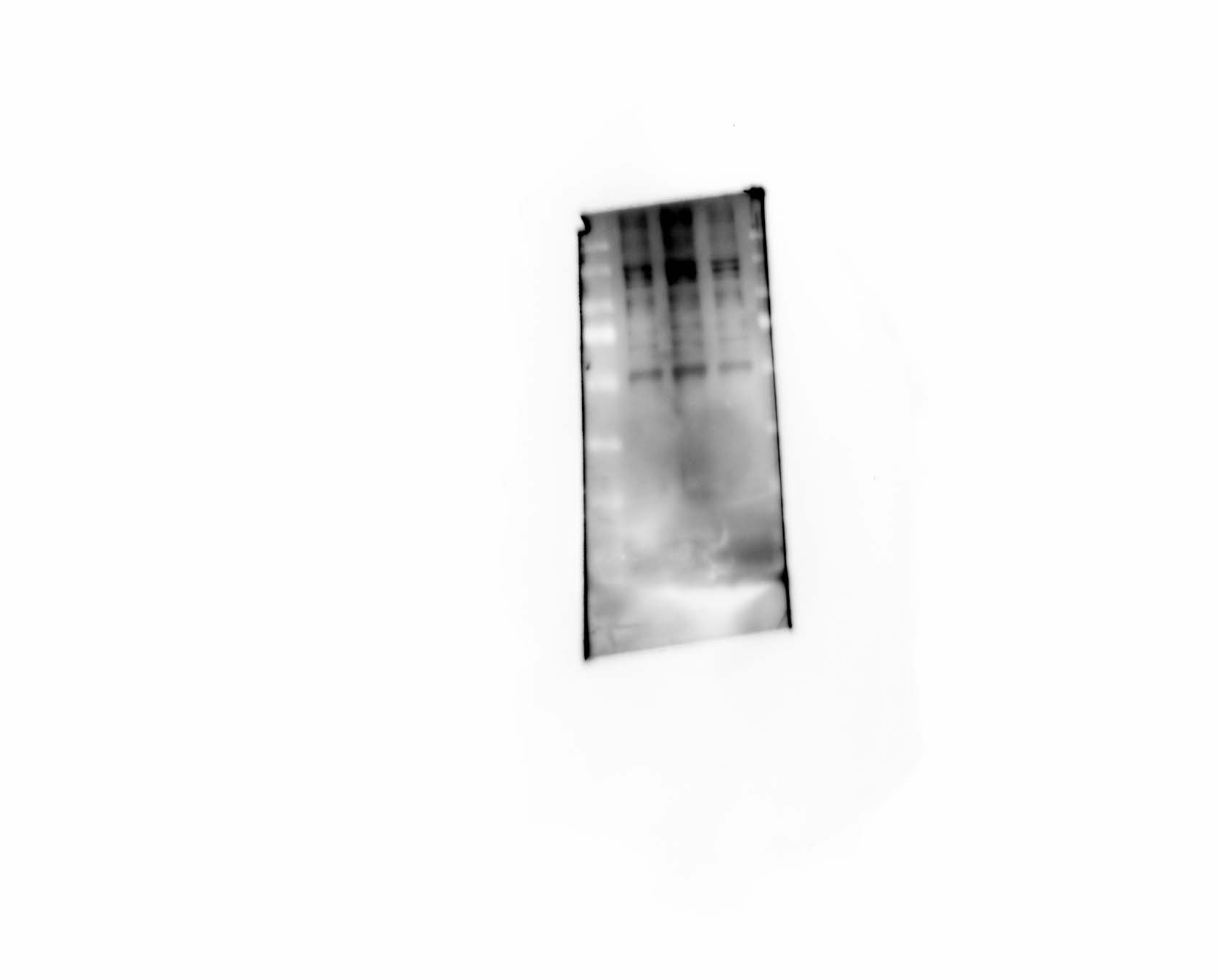

Supplement: Supplementary file 2 — Supporting File 2: advs73976‐sup‐0002‐SuppMat.zip. [file ADVS-13-e11217-s002.zip › WB#U4ee3#U8868#U56fe/Figure S2 Ub/K228R-JPEG/UB-4_4.jpg]

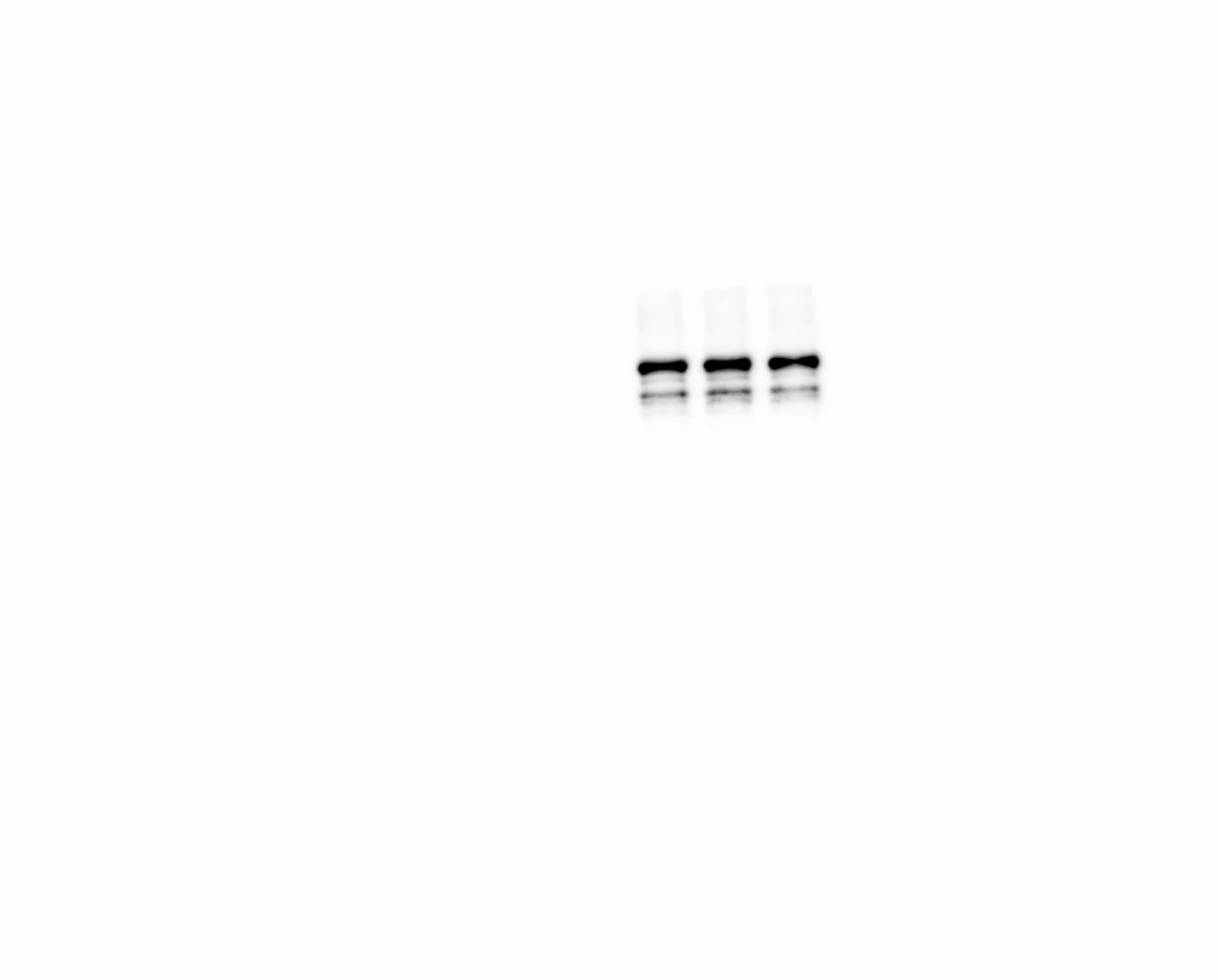

Supplement: Supplementary file 2 — Supporting File 2: advs73976‐sup‐0002‐SuppMat.zip. [file ADVS-13-e11217-s002.zip › WB#U4ee3#U8868#U56fe/Figure S2 Ub/XIAP-JPEG/HA-2_10.jpg]

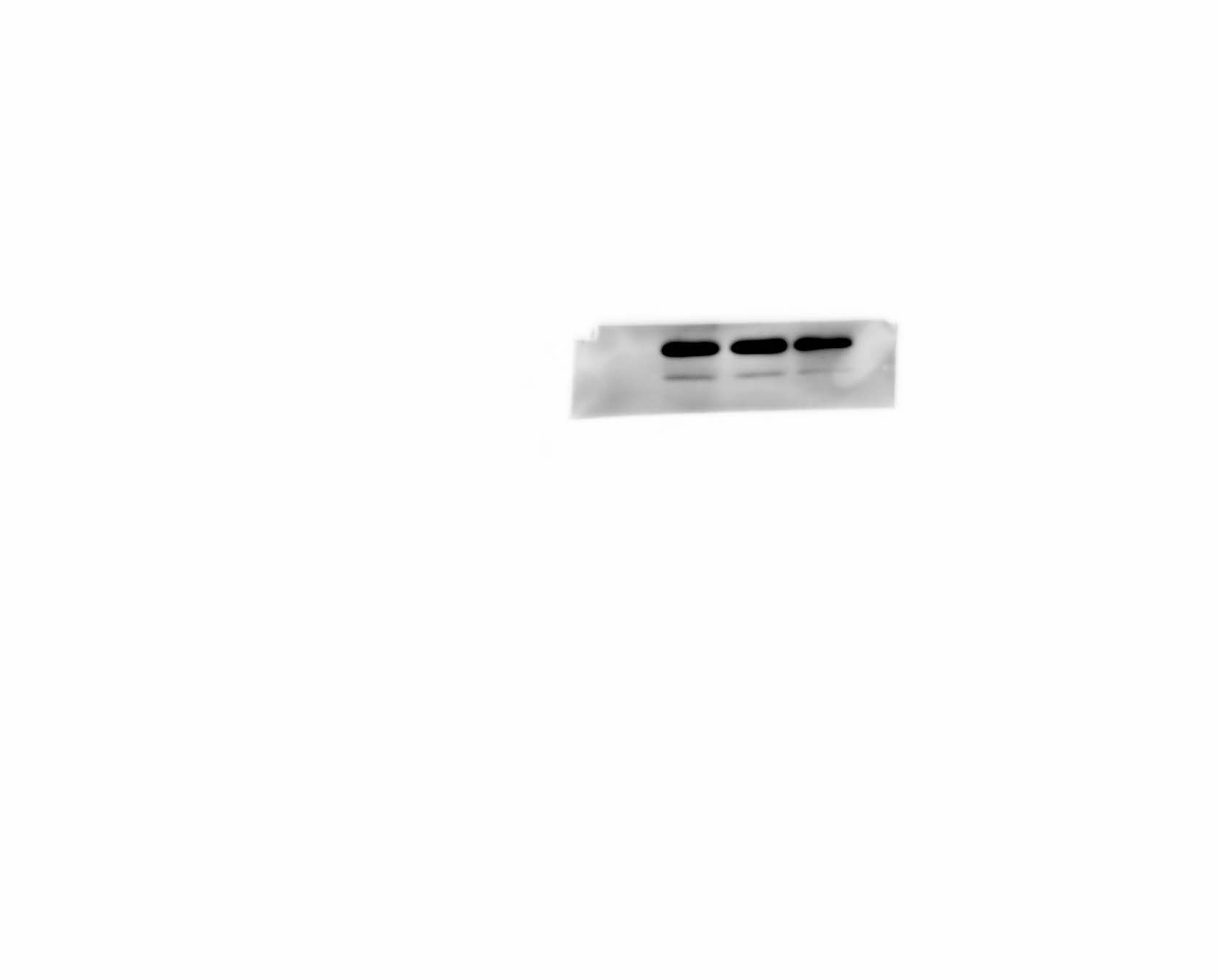

Supplement: Supplementary file 2 — Supporting File 2: advs73976‐sup‐0002‐SuppMat.zip. [file ADVS-13-e11217-s002.zip › WB#U4ee3#U8868#U56fe/Figure S2 Ub/XIAP-JPEG/INPUT-GAPDH-1_5.jpg]

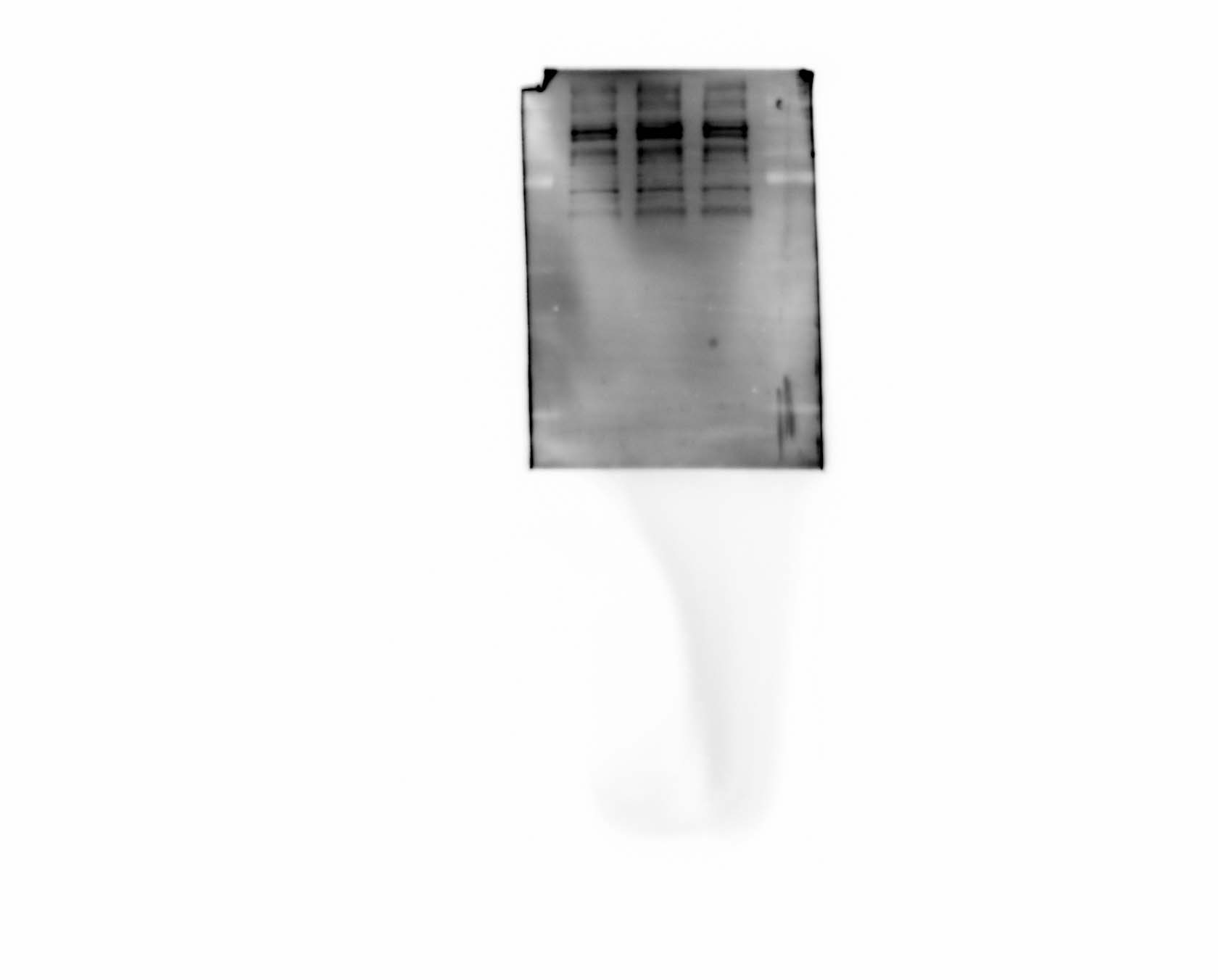

Supplement: Supplementary file 2 — Supporting File 2: advs73976‐sup‐0002‐SuppMat.zip. [file ADVS-13-e11217-s002.zip › WB#U4ee3#U8868#U56fe/Figure S2 Ub/XIAP-JPEG/UB-2-3_7.jpg]

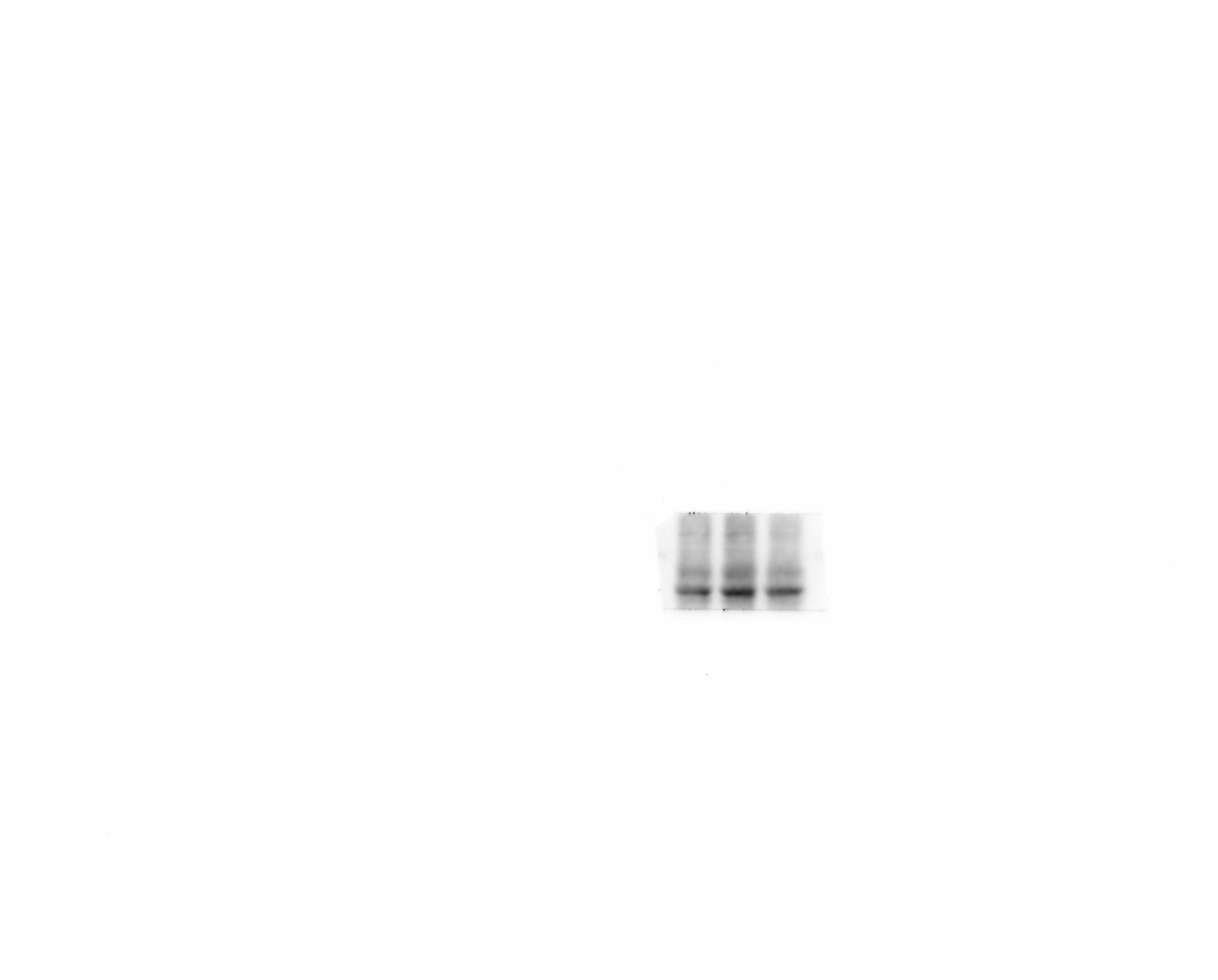

Supplement: Supplementary file 2 — Supporting File 2: advs73976‐sup‐0002‐SuppMat.zip. [file ADVS-13-e11217-s002.zip › WB#U4ee3#U8868#U56fe/xiap#U539f#U59cb#U6570#U636ewb1-JPEG/3-nt_6 oex.jpg]

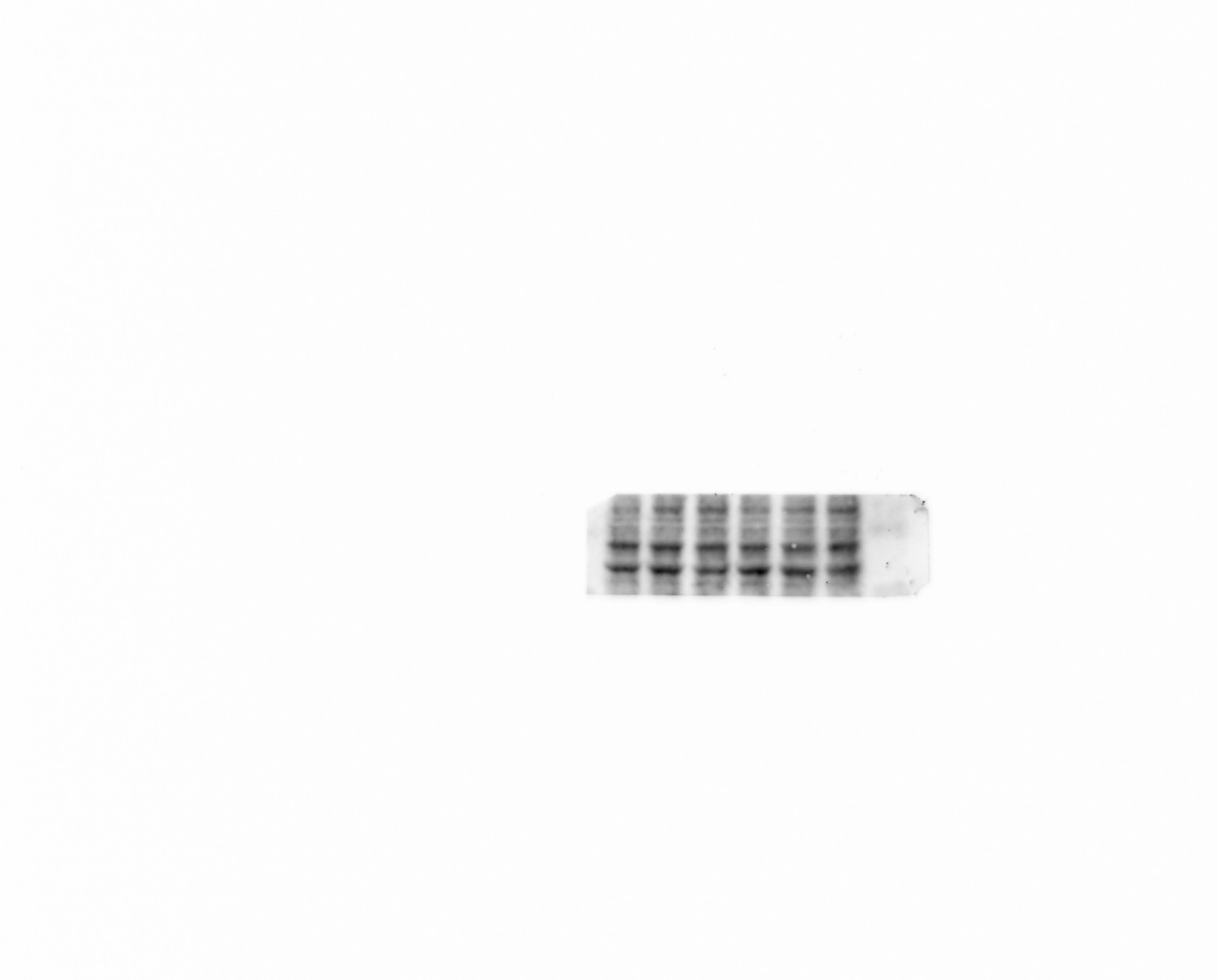

Supplement: Supplementary file 2 — Supporting File 2: advs73976‐sup‐0002‐SuppMat.zip. [file ADVS-13-e11217-s002.zip › WB#U4ee3#U8868#U56fe/xiap#U539f#U59cb#U6570#U636ewb1-JPEG/3-nt_6#U4ee3#U8868.jpg]

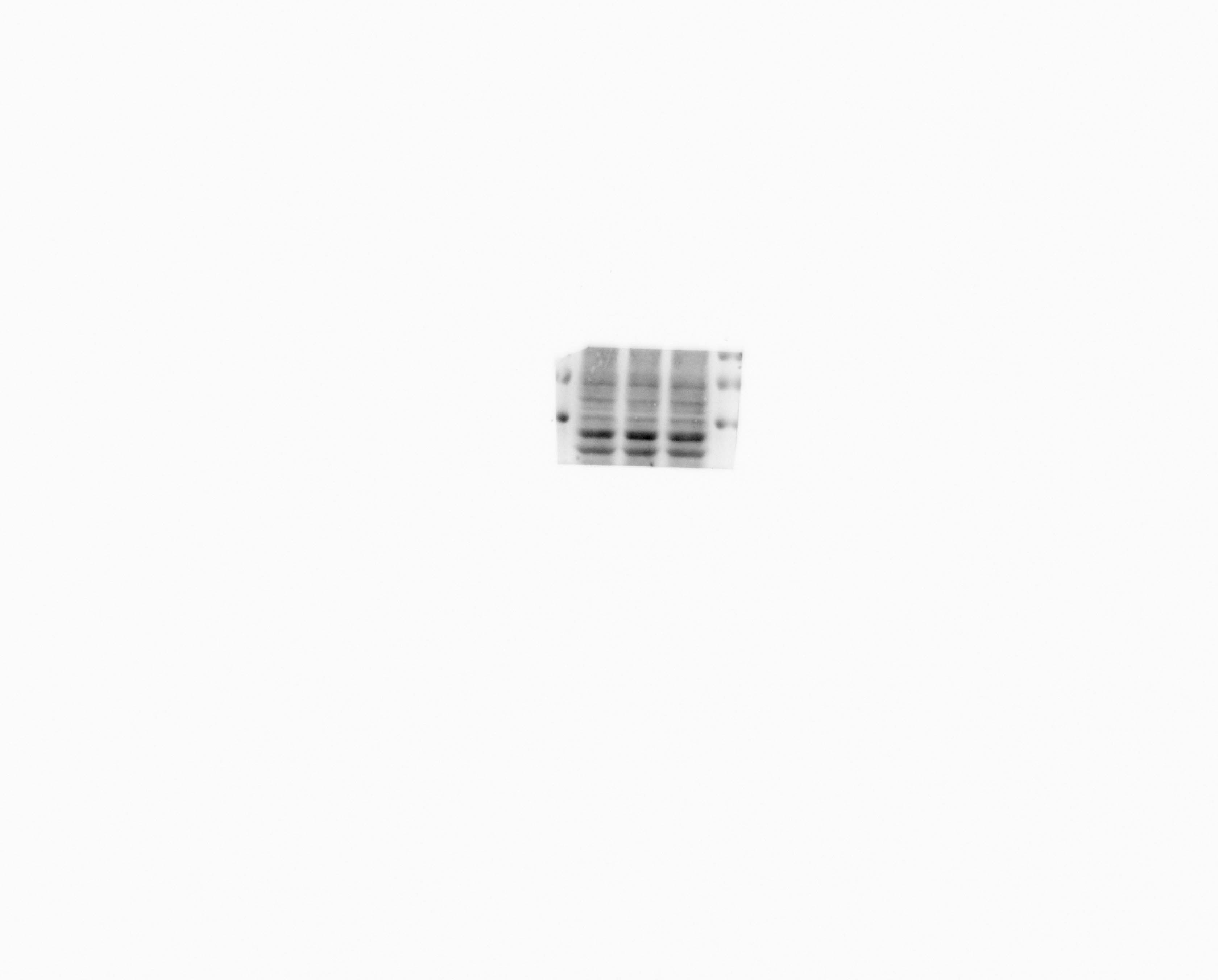

Supplement: Supplementary file 2 — Supporting File 2: advs73976‐sup‐0002‐SuppMat.zip. [file ADVS-13-e11217-s002.zip › WB#U4ee3#U8868#U56fe/xiap#U539f#U59cb#U6570#U636ewb1-JPEG/4-HNE_1#U4ee3#U8868 oex.jpg]

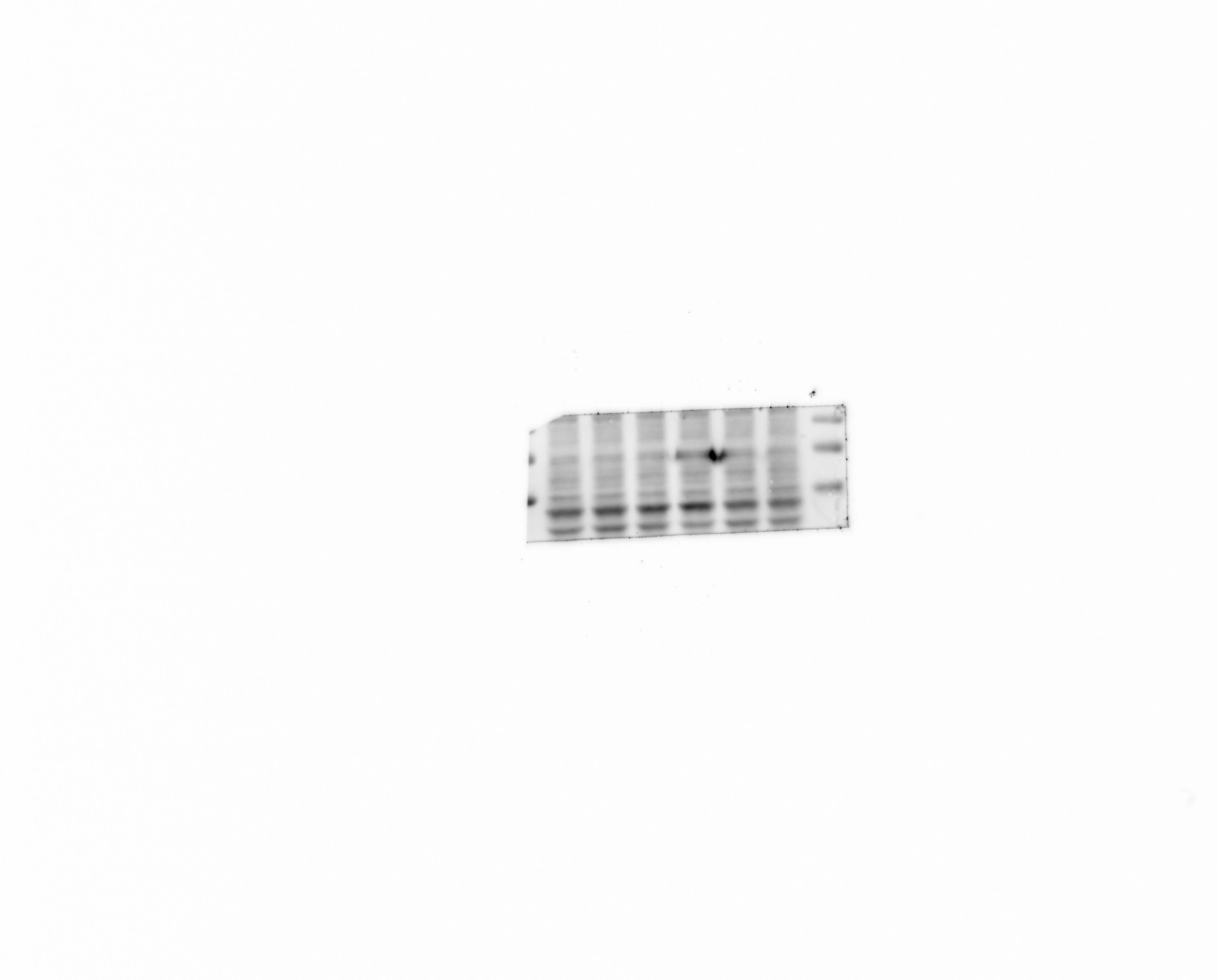

Supplement: Supplementary file 2 — Supporting File 2: advs73976‐sup‐0002‐SuppMat.zip. [file ADVS-13-e11217-s002.zip › WB#U4ee3#U8868#U56fe/xiap#U539f#U59cb#U6570#U636ewb1-JPEG/4-HNE_6.jpg]

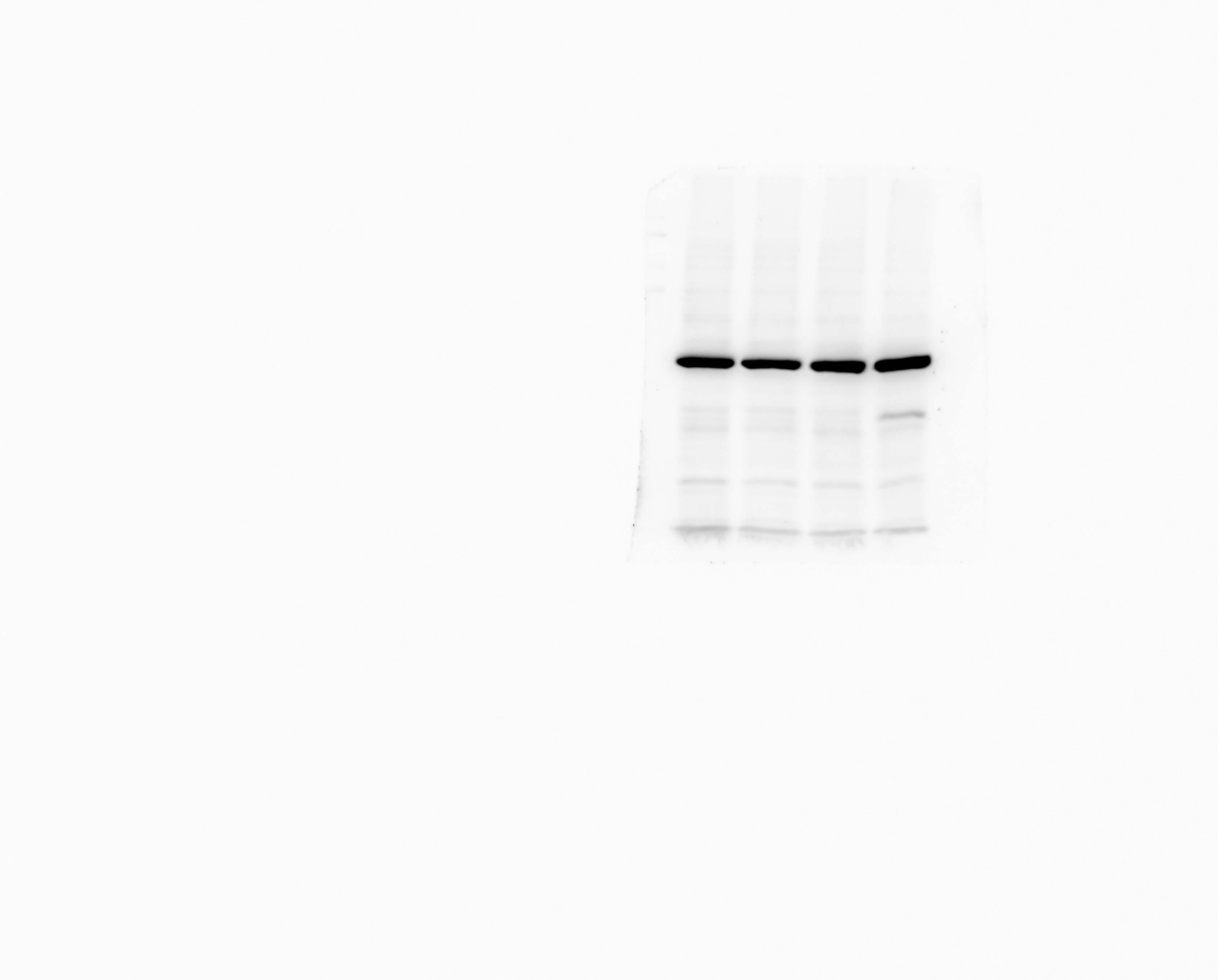

Supplement: Supplementary file 2 — Supporting File 2: advs73976‐sup‐0002‐SuppMat.zip. [file ADVS-13-e11217-s002.zip › WB#U4ee3#U8868#U56fe/xiap#U539f#U59cb#U6570#U636ewb1-JPEG/ACTIN-FLAG_10 db m34.jpg]

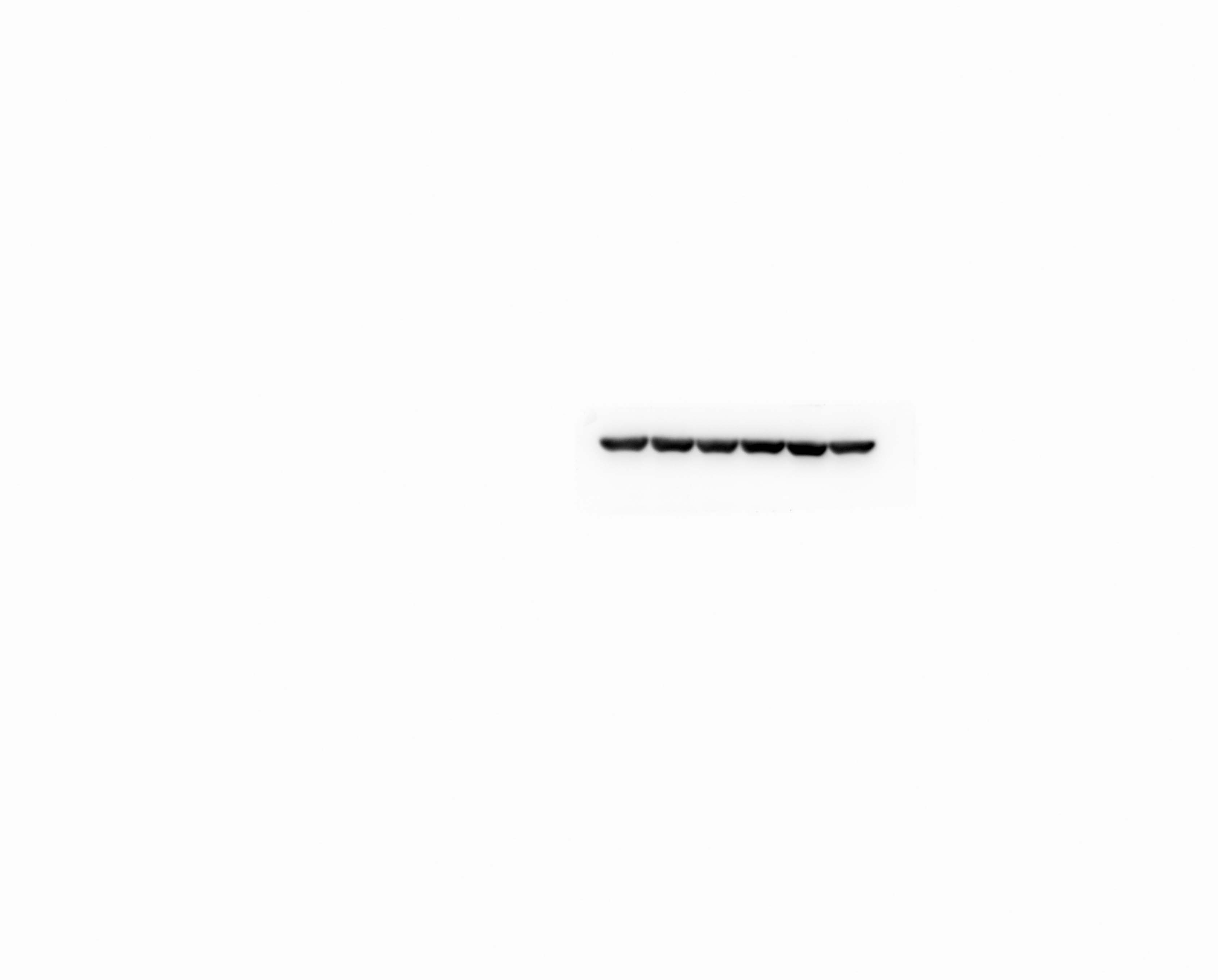

Supplement: Supplementary file 2 — Supporting File 2: advs73976‐sup‐0002‐SuppMat.zip. [file ADVS-13-e11217-s002.zip › WB#U4ee3#U8868#U56fe/xiap#U539f#U59cb#U6570#U636ewb1-JPEG/ACTIN_5 lamp1.jpg]

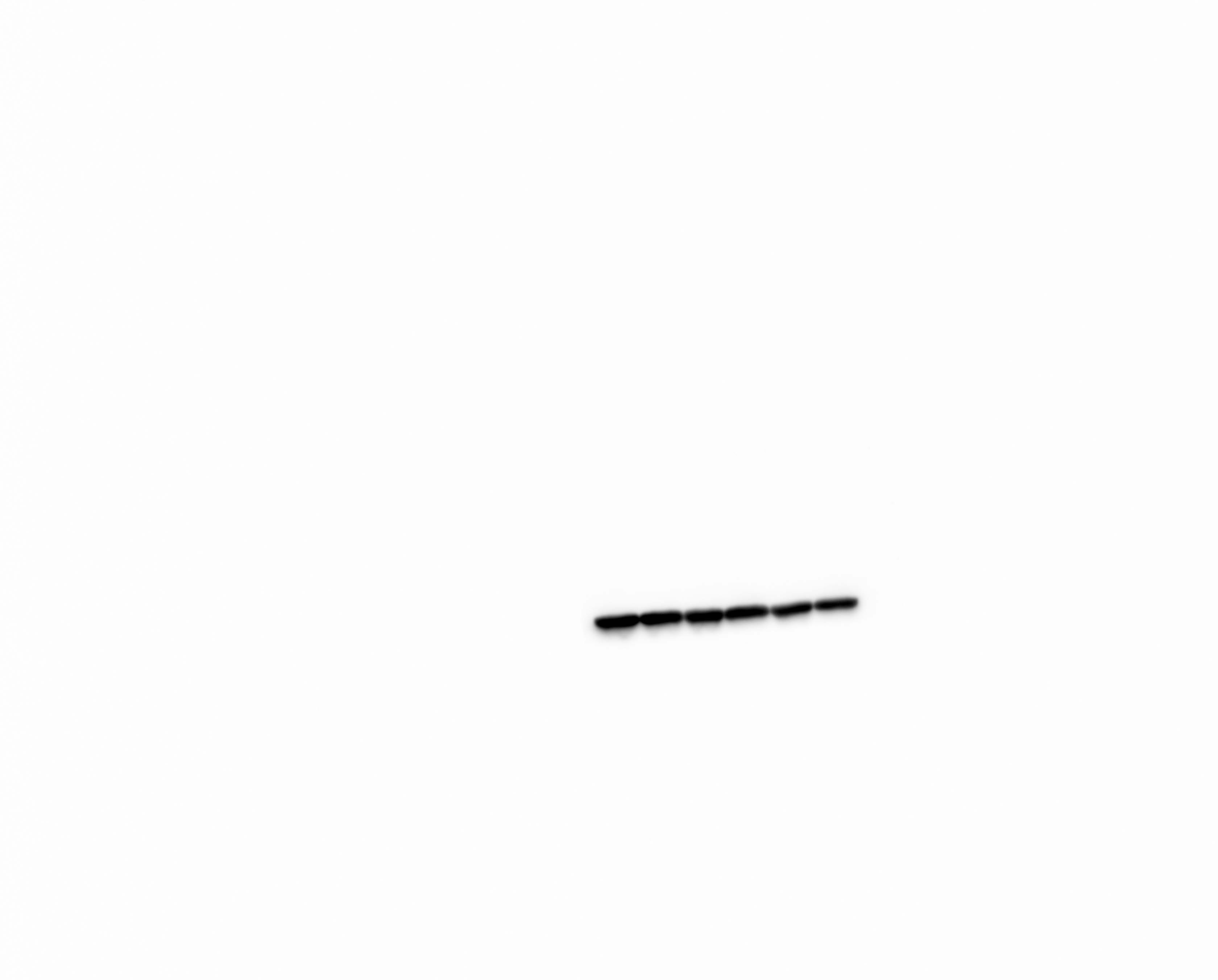

Supplement: Supplementary file 2 — Supporting File 2: advs73976‐sup‐0002‐SuppMat.zip. [file ADVS-13-e11217-s002.zip › WB#U4ee3#U8868#U56fe/xiap#U539f#U59cb#U6570#U636ewb1-JPEG/ACTIN_7 DB EIF2A.jpg]

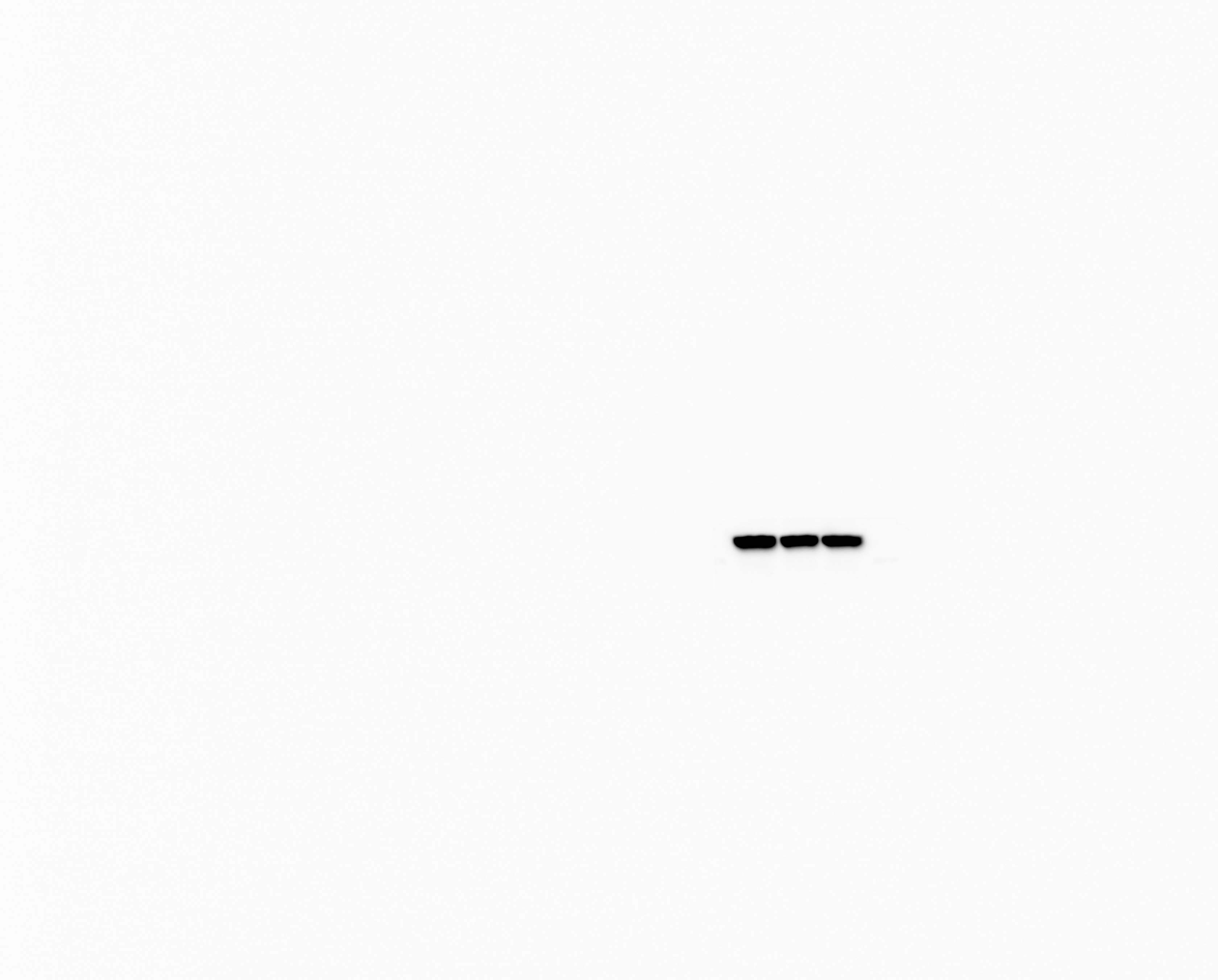

Supplement: Supplementary file 2 — Supporting File 2: advs73976‐sup‐0002‐SuppMat.zip. [file ADVS-13-e11217-s002.zip › WB#U4ee3#U8868#U56fe/xiap#U539f#U59cb#U6570#U636ewb1-JPEG/ACTIN_7 lamp1 oex.jpg]

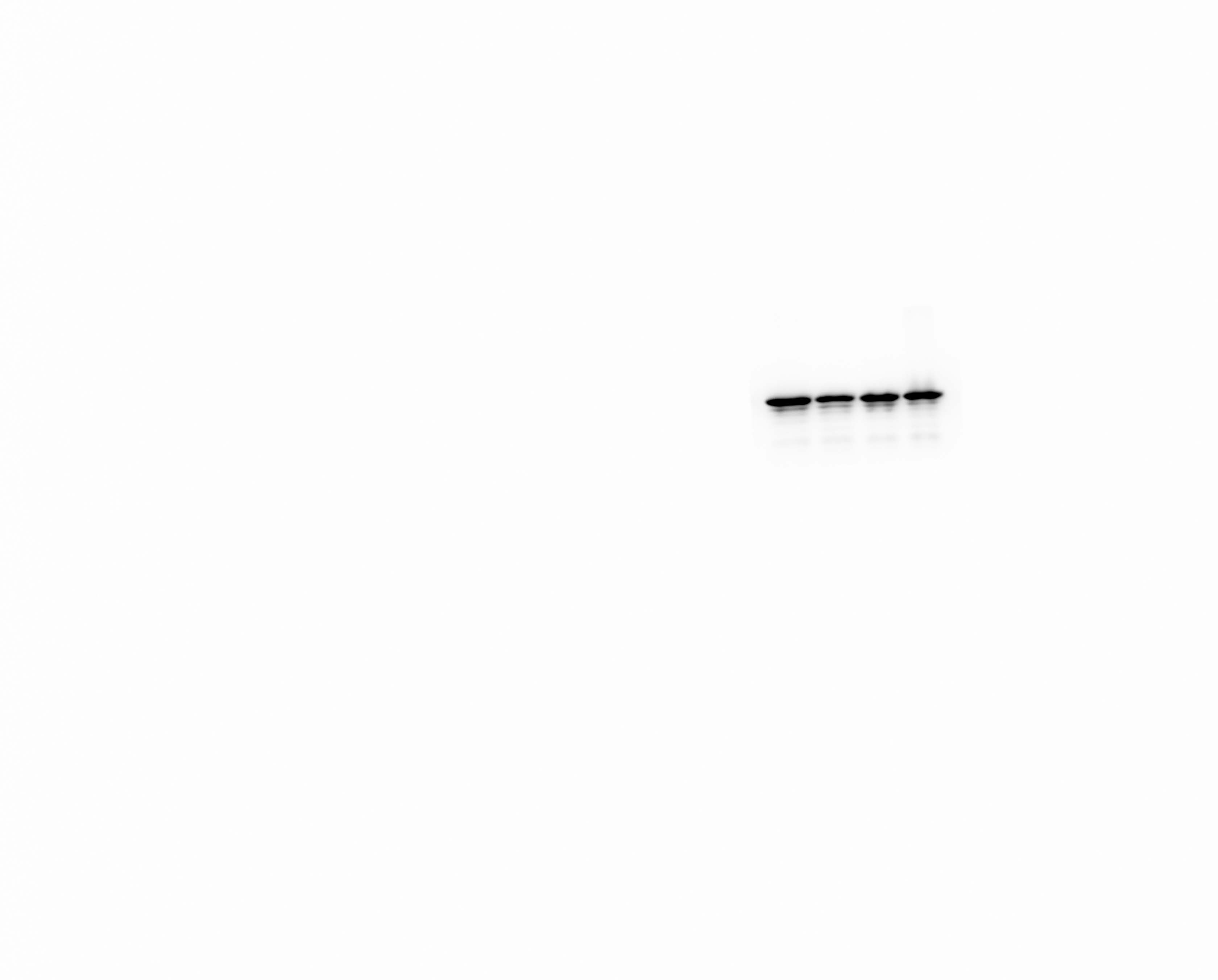

Supplement: Supplementary file 2 — Supporting File 2: advs73976‐sup‐0002‐SuppMat.zip. [file ADVS-13-e11217-s002.zip › WB#U4ee3#U8868#U56fe/xiap#U539f#U59cb#U6570#U636ewb1-JPEG/actin_7 #U4ee3#U8868 sheif#U6548#U7387.jpg]

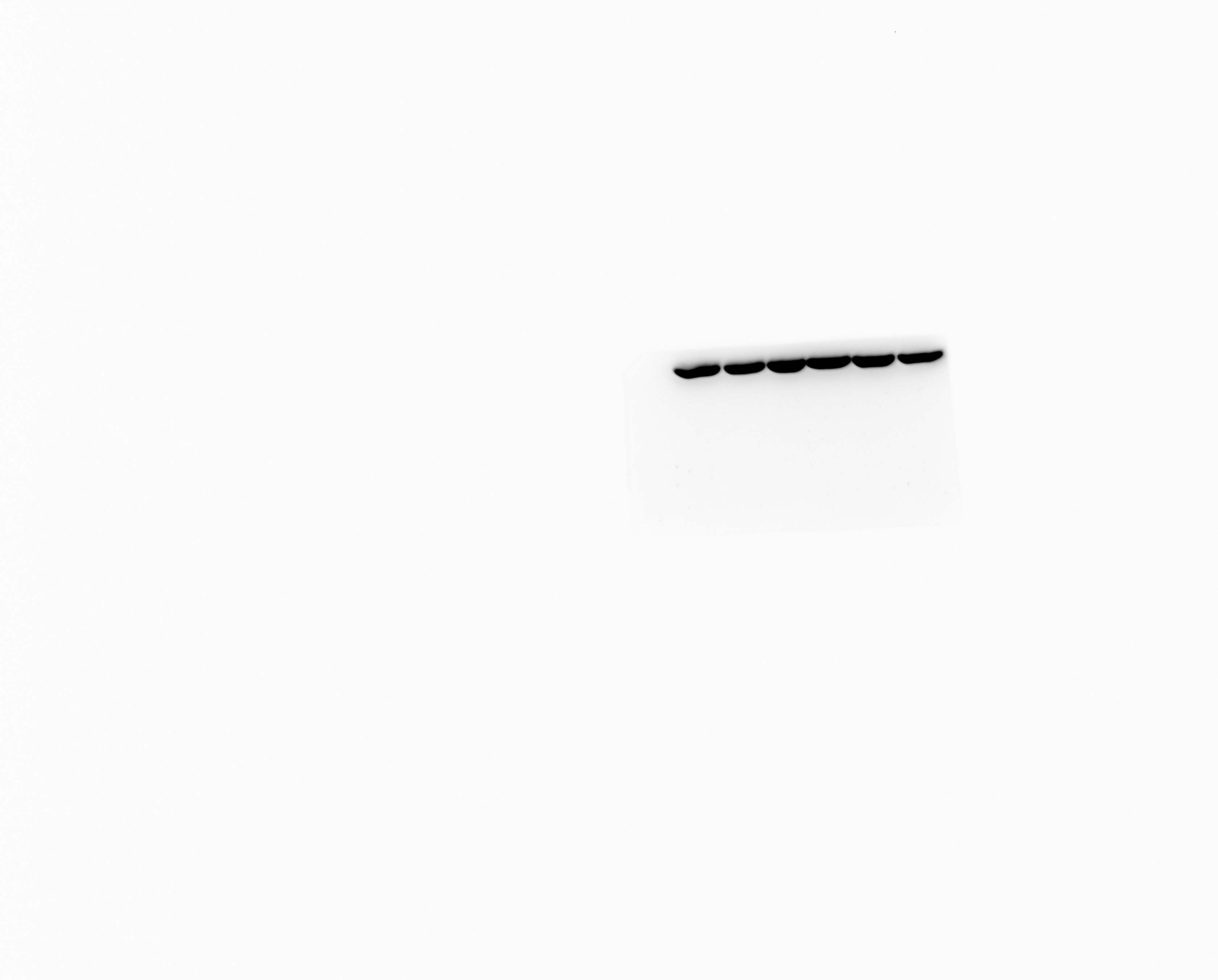

Supplement: Supplementary file 2 — Supporting File 2: advs73976‐sup‐0002‐SuppMat.zip. [file ADVS-13-e11217-s002.zip › WB#U4ee3#U8868#U56fe/xiap#U539f#U59cb#U6570#U636ewb1-JPEG/ACTIN_8 -canx.jpg]

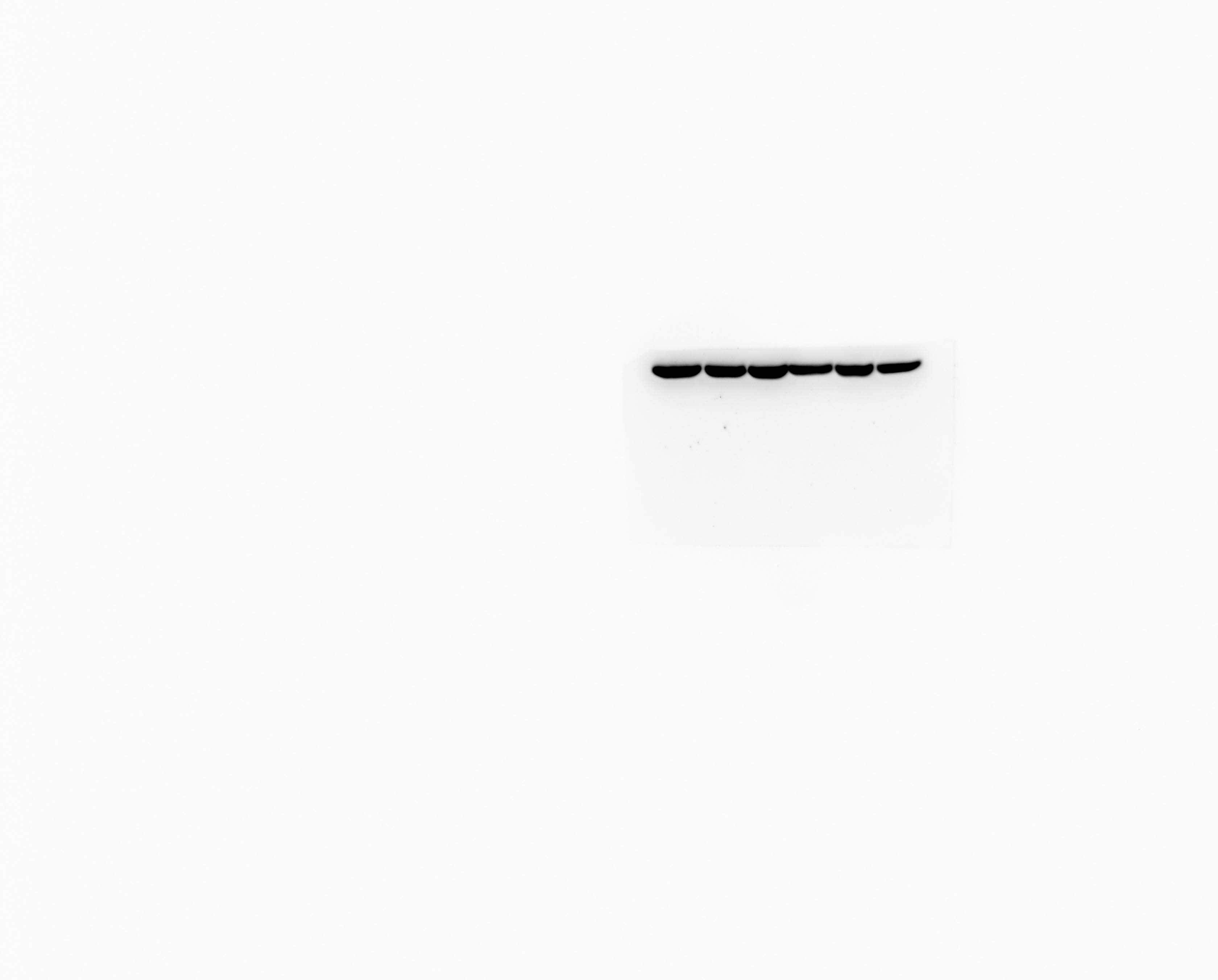

Supplement: Supplementary file 2 — Supporting File 2: advs73976‐sup‐0002‐SuppMat.zip. [file ADVS-13-e11217-s002.zip › WB#U4ee3#U8868#U56fe/xiap#U539f#U59cb#U6570#U636ewb1-JPEG/ACTIN_8 atl3.jpg]
